# Iridium-catalyzed enantioconvergent hydrogenation of trisubstituted olefins

**DOI:** 10.1038/s41467-022-28003-6

**Published:** 2022-01-18

**Authors:** Bram B. C. Peters, Jia Zheng, Norman Birke, Thishana Singh, Pher G. Andersson

**Affiliations:** 1 Department of Organic Chemistry, Stockholm University, Svante Arrhenius väg 16C, SE-10691 Stockholm, Sweden; 2 School of Chemistry and Physics, University of Kwazulu-Natal, Private Bag X54001, Durban, 4000 South Africa

**Keywords:** Synthetic chemistry methodology, Asymmetric catalysis, Green chemistry

## Abstract

Asymmetric hydrogenation of olefins constitutes a practical and efficient method to introduce chirality into prochiral substrates. However, the absolute majority of the developed methodologies is enantiodivergent and thus require isomerically pure olefins which is a considerable drawback since most olefination strategies produce (*E*/*Z*)-mixtures. Although some advances have been reported, a general enantioconvergent hydrogenation featuring a broad functional group tolerance remains elusive. Here, we report the development of a general iridium-catalyzed enantioconvergent hydrogenation of a broad range of functionalized trisubstituted olefins. The substitution pattern around the olefin is critical; whereas α-prochiral olefins can undergo an enantioconvergent hydrogenation, β-prochiral olefins react in an enantiodivergent manner. The presented methodology hydrogenates α-prochiral substrates with excellent control of enantioselection and high isolated yields. Most importantly, both isomerically pure alkenes as well as isomeric mixtures can be hydrogenated to yield the same major enantiomer in excellent enantiomeric excesses which is unusual in transition-metal catalyzed asymmetric hydrogenations.

## Abstract (web summary)

Usually asymmetric hydrogenation of olefins is enantiodivergent and thus requires isomerically pure olefins, which is a considerable drawback. Here, the authors show a general iridium-catalyzed enantioconvergent hydrogenation of a broad range of functionalized trisubstituted olefins.

## Introduction

**Fig. 1:**
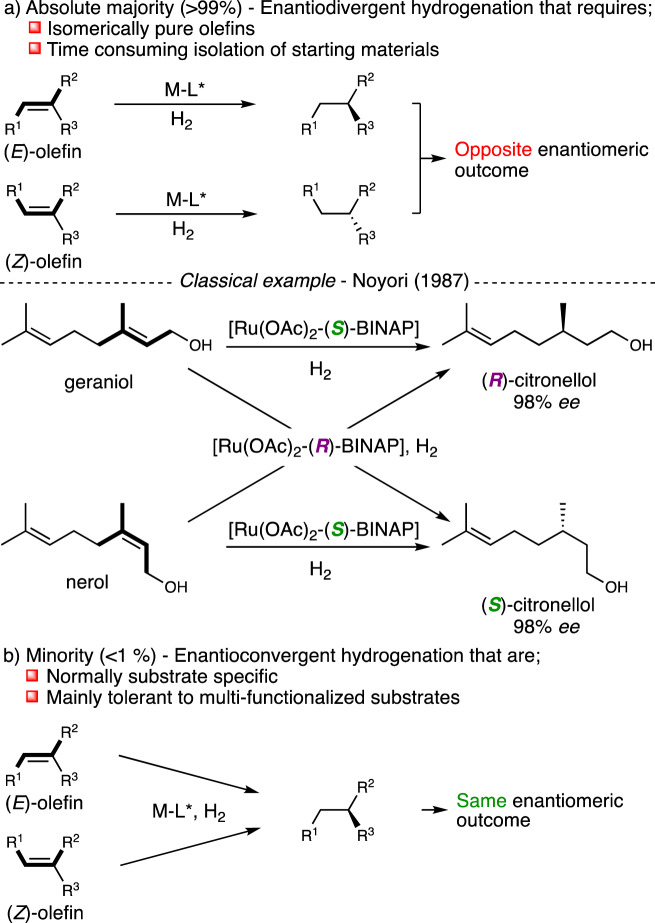
Reported asymmetric hydrogenation of olefins and limitations. **a** Enantiodivergent hydrogenation. **b** Enantioconvergent hydrogenation. BINAP, 2,2′-bis(diphenylphosphino)−1,1′-binaphthalene.

Over the past decades, asymmetric catalysis has received increasing attention by exploring novel reactivity or improving selectivity issues^1–5^. Among the methods to introduce chirality, transition-metal catalyzed asymmetric hydrogenation using hydrogen gas has evolved as one of the most practical methods due to high reactivity, selectivity, atom-economy, and operational simplicity^6–11^. Considering the hydrogenation of carbon-carbon double bonds, nearly perfect chemo- and enantioselectivities have been obtained for a wide range of olefins by developing dedicated ligand libraries to target specific substitution patterns. These findings are important since enantiomers often exhibit different biological and pharmacological properties and as a consequence the pharma industry faces strict rules for the evaluation of potent drug candidates in enantiomerically pure form^12,13^. Despite the maturity of the field of asymmetric hydrogenation, the absolute majority of the hitherto developed systems are enantiodivergent (>99%, Fig. 1a)^14–18^. As a consequence, the geometrical purity around the olefin plays a critical role for both the sense and level of enantioinduction and thus the usefulness of the methodology. Therefore, difficult and time-consuming purification of olefinic substrates is often required prior to hydrogenation since classical olefination methodologies, in most cases, produce mixtures of (*E*/*Z*)-olefins. A classic example that demonstrates the necessity to use pure olefins is the synthesis of citronellol by Noyori in which opposite enantiomers were prepared by starting from either geraniol or nerol^19^. Most methodologies that are reported in more recent years still follow an enantiodivergent hydrogenation that was already observed over three decades ago.

On the other hand, enantioconvergent hydrogenation of olefins is considered the pinnacle in state-of-the-art asymmetric hydrogenation and remains underdeveloped (<1% of all reported methodologies on the asymmetric hydrogenation of olefins, Fig. 1b)^20^. This is mainly attributed to the development of stereodefined catalytic pockets that is fine-tuned to maximize interactions in the hydrogenation of a single stereoisomer of the olefin. This often leads to lower synergy in the hydrogenation of the opposite stereoisomer and in addition, catalysts often show opposite enantioselection between (*E*)- and (*Z*)-olefins. This complicates the hydrogenation of isomeric mixtures since, in a useful enantioconvergent methodology, the enantioselectivity in the hydrogenation of both geometries of the olefin should yield the same enantiomer preferably in high selectivity. Although few enantioconvergent hydrogenations have been reported, these systems showed to be highly substrate-specific and require mostly multi-functionalized olefins and fine-tuned (transition-)metal catalysts^20^. Therefore, the development of a general enantioconvergent hydrogenation methodology that tolerates a broad range of olefinic starting materials remains an unsolved task.

**Fig. 2:**
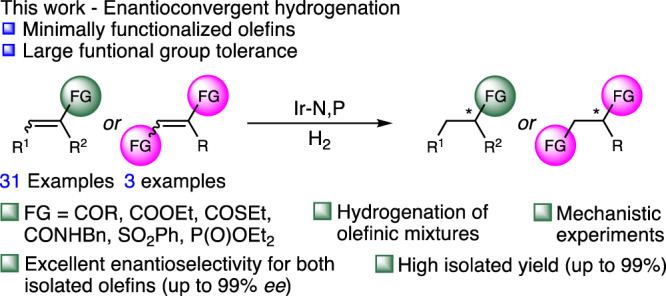
This work. FG functional group.

In this report, we utilize a bidentate N,P-ligated iridium complex to hydrogenate various olefins in an enantioconvergent fashion (Fig. 2). To demonstrate the efficiency of the methodology, high levels of enantiopurity of the hydrogenated products were accessed by subjecting either pure isomeric olefins or a mixture of both.

## Results and discussion

### Initial experiments and observations

**Fig. 3:**
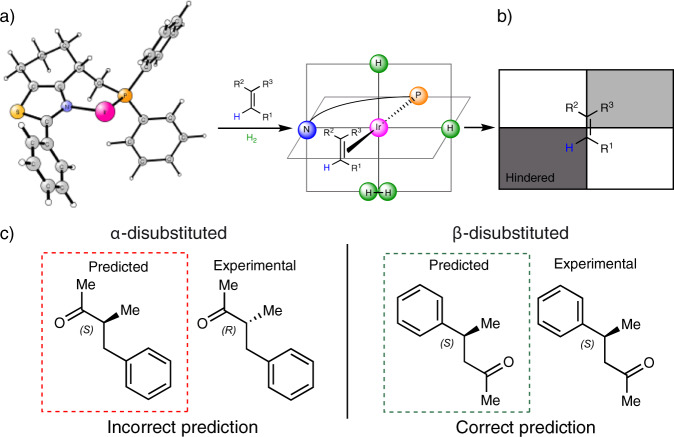
Origin and analysis of the stereoselectivity model. **a** Coordination of the ligand and the substrate to the iridium center. **b** Quadrant model based on the steric environment of the ligand around the coordinated olefin. **c** Predicted and experimentally observed stereochemical outcome for the hydrogenation of enones.

The Andersson group has earlier developed a selectivity model to rationalize the stereochemical outcome of hydrogenations of trisubstituted olefins and iridium-N,P complexes^21–23^. Calculations have suggested that the coordination of an unfunctionalized olefin towards an [IrH_2_(N,P)(Solvent)_2_]^+^ complex takes place in the same equatorial plane as the chiral bidentate N,P-ligand and *trans* to phosphorus (Fig. 3a). The resultant quadrant model rationalizes whether the steric bulk of the ligand is above or below the N-Ir-P plane (Fig. 3b). To minimize steric interactions, the substrate then preferentially coordinates with the smallest olefin substituent, in this case the vinylic proton, to the hindered environment and thus coordinates by one of the enantiotopic faces in favor. This mode of coordination is supported by computational findings of other research groups using different iridium complexes^24–27^. Most important, experimental support for this hypothetical coordination was reported by an elegant low-temperature NMR study of Pfaltz in which a fundamental intermediate, an Ir^III^ dihydride alkene complex akin to the above-described, was identified^28^. Intriguingly, although the model correctly predicts the stereochemical outcome for most olefins, it cannot be used for the hydrogenation of olefins having a conjugated carbonyl group^29,30^. Whereas the correct absolute configuration can be predicted for the formation of β-disubstituted carbonyl compounds, hydrogenation of α-disubstituted unsaturated carbonyl compounds yield the opposite enantiomer compared to the prediction (Fig. 3c).

**Fig. 4:**
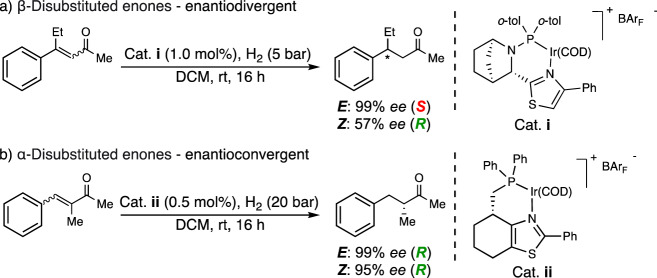
Enantiodivergent/enantioconvergent hydrogenation of trisubstituted enones. **a** β-Disubstituted enones. **b** α-Disubstituted enones. DCM dichloromethane, rt room temperature, COD 1,5-cyclooctadiene, BAr_F_ tetrakis[3,5-bis(trifluoromethyl)phenyl]borate.

The failure of the model to predict the correct stereochemical outcome for the α-disubstituted enones indicates that this class of substrates might undergo hydrogenation via a different mechanism. Previous work already demonstrated that the hydrogenation of β-prochiral unsaturated carbonyl compounds follows the selectivity model and that it is also enantiodivergent which again is in accordance with the selectivity model (Fig. 4a)^29,30^. However, during the evaluation of the two isomeric forms of the α-disubstituted enone **1a** it was found that both (*E*)-**1a** and (*Z*)-**1a** surprisingly gave rise to the (*R*)-enantiomer, and in high selectivity (99% and 95% *ee* respectively, Fig. 4b). That both the (*E*)- and (*Z*)-isomers of a given olefin produce the same enantiomer in high selectivity is an unusual phenomenon in asymmetric hydrogenations and we were therefore encouraged to investigate the generality of the observed enantioconvergent behavior.

### Scope of enantioconvergent hydrogenation

**Fig. 5:**
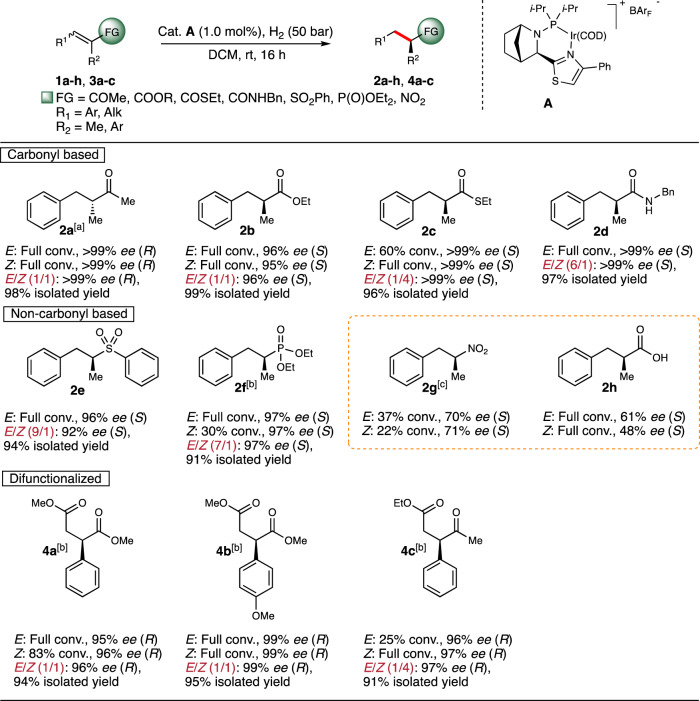
Enantioconvergent hydrogenation of trisubstituted olefins. Reaction conditions: 0.1 mmol substrate, 1.0 mol% catalyst, 2 mL DCM, 50 bar H_2_, 16 h, rt, unless otherwise stated. Isomerically pure starting materials were evaluated on 0.05 mmol scale in 1 mL DCM and conversion was determined by ^1^H NMR spectroscopy. Enantiomeric excess was determined by SFC or GC analysis, using chiral stationary phases. [a] Catalyst **B** was used. [b] 100 bar H_2_. [c] 2.0 mol% catalyst. FG functional group, DCM dichloromethane, rt room temperature, COD 1,5-cyclooctadiene, BAr_F_ tetrakis[3,5-bis(trifluoromethyl)phenyl]borate, conv. conversion.

First, we wanted to explore to what extent the convergent hydrogenation could be applied to a broader range of substrates. Various *N*-heterocyclic-phosphine ligated complexes were initially evaluated in the hydrogenation of (*Z*)-**1b** (see Supplementary Information for optimization details) and the aza-bicyclo thiazole backbone was found superior in terms of enantioselection. Modification of the phosphine substituents demonstrated that complex **A** hydrogenated both geometries of **1b** in high enantioselectivity (96% *ee* from (*E*)-**1b** and 95% *ee* from (*Z*)-**1b**). Although catalyst **A** is also highly selective in the specific case of enones (99% *ee* from (*E*)-**1a** and 96% *ee* from (*Z*)-**1a**), a slightly improved selectivity was found when catalyst **B** was used. With the optimized reaction conditions in hand, (employing 1 mol% of the catalyst under 50 bar of hydrogen gas in DCM,) a large number of functional groups were found to be compatible and result in convergent reactions (Fig. 5). Chiral ketone **2a** was obtained in excellent enantioselectivity (>99% *ee*), regardless of the geometry around the olefin of the starting α,β-unsaturated enone **1a**. (Thio)ester and secondary amide derivatives **1b**, **1c**, and **1d** were all well tolerated giving 96% *ee*, >99% *ee,* and >99% *ee*, respectively, also when isomeric mixtures of (*E*)- and (*Z*)-olefins were employed. Interestingly, evaluation of the complex ligated with the commercial PHOX ligand in the hydrogenation of **1a** (*E*/*Z*, 87:13) provided **2b** in only 22% *ee* (See Supplementary Information). To our delight, the enantioconvergent hydrogenation was not limited to carbonyl functional groups and phenyl sulfone **1e** was hydrogenated in 92% *ee*. Although (*Z*)-ethyl phosphonate (*Z*)-**1f** underwent hydrogenation at a slower rate compared to (*E*)-**1f**, both isomers were hydrogenated in 97% *ee*. Despite vinyl nitro compound **1h** and α-methylcinnamic acid **1g** were hydrogenated in lower reactivity and/or selectivity, both substrates were found to follow an enantioconvergent mechanism. Additionally, γ-diesters **3a** and **3b** were also tolerated resulting in 96% *ee* and 99% *ee* of the corresponding hydrogenated products **4a** and **4b**, respectively, also when mixtures of (*E*/*Z*) were subjected to the reaction. The γ-ketoester **3c** resulted in a larger rate difference between isomeric starting materials, however, a 1:4 (*E*/*Z*) mixture was hydrogenated in excellent results of 97% *ee* and 91% isolated yield.

**Fig. 6:**
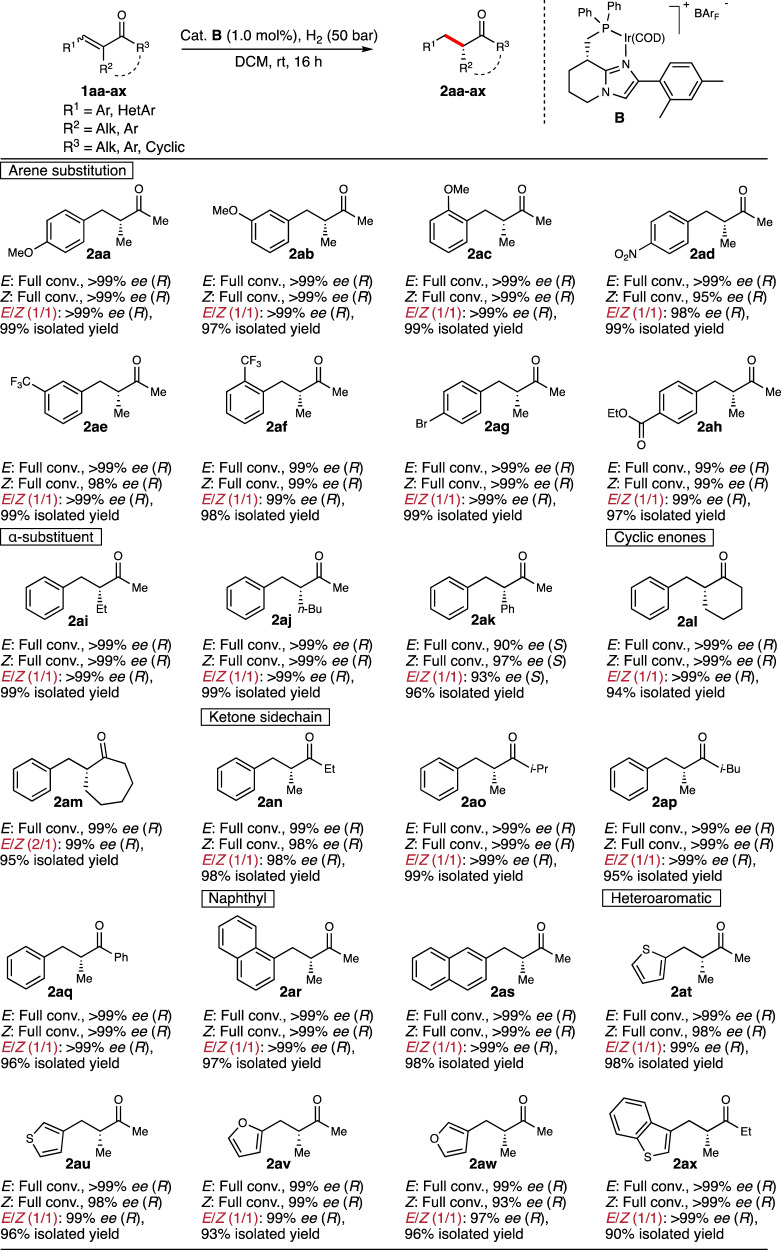
Enantioconvergent hydrogenation of trisubstituted unsaturated enones. Reaction conditions: 0.1 mmol substrate, 1.0 mol% catalyst, 2 mL DCM, 50 bar H_2_, 16 h, rt. Isomerically pure starting materials were evaluated on 0.05 mmol scale in 1 mL DCM and conversion was determined by ^1^H NMR spectroscopy. Enantiomeric excess was determined by SFC or GC analysis, using chiral stationary phases. DCM dichloromethane, rt room temperature, COD 1,5-cyclooctadiene, BAr_F_ tetrakis[3,5-bis(trifluoromethyl)phenyl]borate, conv. conversion.

Next, a range of substituted α,β-unsaturated ketones were evaluated using catalyst **B** (Fig. 6). The reaction showed to be insensitive to the electronic properties of the phenyl ring as both strong electron-donating and electron-withdrawing substituents in all positions of the aromatic ring were hydrogenated in excellent results (**2aa–2af**, 98->99% *ee*, also when starting from 1:1 mixtures of (*E*/*Z*)-isomers). In addition, *para*-bromo and *para*-ethyl ester substrates were both hydrogenated in enantioselectivity of 99% *ee* (**2ag** and **2ah**). Changing the methyl group on the α-position to ethyl and *n*-butyl both afforded the chiral ketones in >99% *ee* (**2ai** and **2aj**) whereas the α-phenyl enone **1ak** was hydrogenated with a slight decrease in enantioselectivity (93% *ee*). Cyclohexanone and cycloheptanone-derived enones **1al** and **1am** were both hydrogenated in 99% *ee* as mixtures of starting geometries. Different substituents on the ketone side-chain were also evaluated and hydrogenation of ethyl, *i*-propyl, *i*-butyl, and phenyl substituted ketone all yielded the desired products in 98->99% *ee* (**2an–2aq**). Finally, both naphthyl, as well as various heteroaromatic substrates, were all hydrogenated in excellent enantioselectivities and isolated yields (**1ar–1ax**, 97->99% *ee*, >90% yield), also when 1:1 isomeric mixtures were employed (no conversion was obtained when the hydrogenations of R^1^ = 3-pyridyl, 4-pyridyl or 2-thiazolyl with R^2^ = R^3^ = Me were attempted. The heteroaromatic moiety most likely forms a ligand on the iridium complex, alternatively, it is too basic and deprotonates the iridium dihydrogen complex).

### Scale-up enantioconvergent hydrogenation, sequential olefination/hydrogenation, and mechanistic experiment

**Fig. 7:**
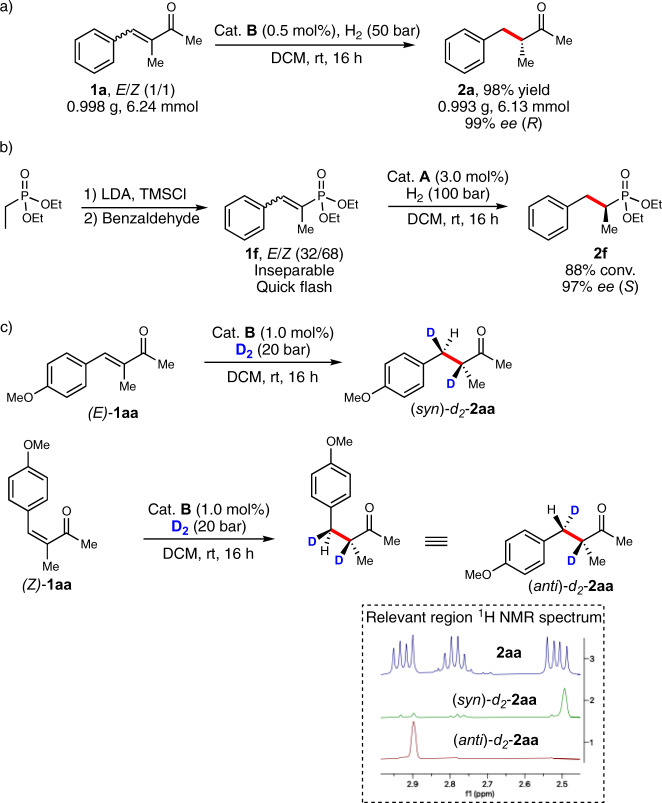
Practical applications and deuterium gas study. **a** Gram-scale enantioconvergent hydrogenation. **b** Olefination/hydrogenation sequence. **c** D_2_-gas hydrogenation. DCM dichloromethane, rt room temperature, LDA lithium diisopropylamide, TMSCl trimethylsilyl chloride, conv. conversion.

Then, the developed protocol was evaluated on a gram-scale and **1a** was smoothly hydrogenated as a mixture of geometries to provide **2a** in optically pure form (99% *ee*) and 98% yield (Fig. 7a). In addition, the usefulness of this methodology was demonstrated in the hydrogenation of **1f** which geometrical isomers cannot be separated by ordinary column chromatography (silica). The synthesis of **1f** by a Peterson olefination provided a 32:68 (*E*/*Z*) mixture which was isolated by a quick flash column chromatography and subsequently hydrogenated to give **2f** in 97% *ee* (Fig. 7b).

As last, our group recently reviewed^20^ the proposed mechanisms for enantioconvergent hydrogenations and concluded that they can either be due to; (1) a dynamic isomerization between the (*E*)- and (*Z*)-geometry of the starting olefin, accompanied with a large difference in hydrogenation rate for the two isomeric forms. Or, (2) by a chelation-controlled hydrogenation for substrates possessing a coordinating group on the prochiral carbon of the olefin. In order to exclude one of these two mechanistic possibilities, deuterium-labeling experiments were performed. Individual hydrogenation of both (*E*)-**1aa** and (*Z*)-**1aa** under D_2_ atmosphere resulted in the formation of two different diastereomeric products (Fig. 7c). This indicates that the enantioconvergent hydrogenation is not a result of isomerization between starting geometries prior to hydrogenation.

In summary, we have developed a general and highly efficient strategy for the enantioconvergent hydrogenation of trisubstituted olefins using a bidentate N,P-ligated iridium complex. The reaction tolerates a broad substrate scope of α-prochiral functionalized olefins in terms of chelating functional group as well as a wide range of (heteroaromatic) substitution patterns. The olefins can be hydrogenated either in isomerically pure forms or as isomeric mixtures yielding the same absolute configuration in excellent enantiomeric excess and isolated yield. We anticipate that our findings will be applicable to a large number of asymmetric hydrogenations of mixtures of (*E*)- and (*Z*)-olefins.

## Methods

### General procedure for the enantioconvergent hydrogenation

An oven-dried vial was charged with olefin (0.1 mmol, 1.0 equiv.) and Ir-N,P-catalyst (1.0 mol%). DCM (2 mL) and a magnetic stirring bar were added and the vial was placed in a high-pressure hydrogenation apparatus. The reactor was purged three times with Ar, purged three times with H_2_ and then pressurized with H_2_ (50–100 bar). The reaction was stirred at room temperature for 16 h before the H_2_ pressure was released and the solvent was removed under reduced pressure. The residue was purified by flash chromatography (pentane/Et_2_O, 50/50) on silica gel to give the alkane. The *ee* value was determined by GC analysis or SFC analysis on the chiral stationary phase. The corresponding racemic product was used for comparison and it was prepared on a 0.05-mmol scale using Pd/C (or racemic Ir-catalyst) as the catalyst, following the same hydrogenation procedure. The absolute configuration was determined by comparing the sign of optical rotation with reported values.

## Supplementary information

Supplementary Information

## Data Availability

Experimental procedures, catalyst screening, characterization data of new compounds, separation of chiral products, and NMR spectra of new compounds are available in the Supplementary Information. All data is available from the authors upon request.
