# Supplementary material for: Iridium-catalyzed enantioconvergent hydrogenation of trisubstituted olefins
Source: Nat Commun. 2022 Jan 18;13:361. doi: 10.1038/s41467-022-28003-6 (PMC8766446; doi:10.1038/s41467-022-28003-6)
Supplement: Supplementary file 1 — Supplementary Information [file 41467_2022_28003_MOESM1_ESM.pdf]

SUPPLEMENTARY INFORMATION

Iridium-Catalyzed Enantioconvergent Hydrogenation of Trisubstituted Olefins

Bram B. C. Peters,[a‡] Jia Zheng,[a‡] Norman Birke,[a] Thishana Singh[b] and Pher G. Andersson[*ab]

* E-mail: Pher.Andersson@su.se

[a] Department of Organic Chemistry, Stockholm University, Svante Arrhenius väg 16C, SE-10691 Stockholm, Sweden.

[b] School of Chemistry and Physics, University of Kwazulu-Natal, Private Bag X54001, Durban, 4000, South Africa.

Table of contents

Supplementary Methods……………………………………………………………………2

General information…………………………………………………………………...……2

Experimental details and characterization data of new compounds……………………3

General procedure for the *E* → *Z* isomerization………………………………..3

General procedures for the synthesis of α,β-unsaturated ketones………………3

General procedure for the asymmetric hydrogenation…………………………5

Characterization of hydrogenated products…………………………………..25

Catalyst optimization……………………………………………………………………29

Procedure for the gram-scale hydrogenation…………………………..……….…30

Deuterium gas study……………………………………………………………………..31

NMR spectra………………………………………………………….…………….....33

Separation of chiral products……………………………………..……………..81

Chromatograms………………………………………….……………..………84

Supplementary References…………………………………………………………101

Supplementary Methods

General information

All reaction vessels were dried in a vacuum oven (160 °C) and cooled down to room temperature under a flow of nitrogen prior to use. Dichloromethane was dried over calcium hydride and freshly distilled under nitrogen. THF was distilled from sodium-benzophenone under nitrogen. The commercially available chemicals were used directly or purified by either distillation or column chromatography. Chromatographic separations were performed on Kiesel gel 60 H silica gel (particle size: 0.063-0.100 mm). Thin-layer chromatography (TLC) was performed on aluminum plates coated with Kieselgel 60 (0.20 mm, UV 254 nm) and visualized under ultraviolet light followed by staining with potassium permanganate or phosphomolybdic acid. ^{1}H NMR spectra were recorded at 400 MHz in $CDCl_3$ and referenced internally to the residual $CHCl_3$ signal (7.26 ppm). ^{13}C NMR spectra were recorded at 100 MHz in $CDCl_3$ and referenced to the central peak of $CHCl_3$ (77.16 ppm). ^{19}F NMR spectra were recorded at 377 MHz in $CDCl_3$. ^{31}P NMR spectra were recorded at 162 MHz in $CDCl_3$. Chemical shifts were reported in ppm (δ scale), and coupling constants (*J*) were reported in Hertz (Hz). High resolution mass spectrometric (HRMS) data were obtained from Bruker microTOF-Q II instrument operated at ambient temperatures. Optical rotation was recorded on a thermostated polarimeter using sodium lamp (589 nm) and a 10 cm cell. Enantiomeric excesses were determined using either GC-MS (30 m column, helium gas carrier at 1 mL/min, constant pressure) or SFC-UV (250 mm Chiralcel columns, CO_2/MeOH) using chiral stationary phases. Racemic compounds were in all cases used for comparison.

Experimental details and characterization data of new compounds

General procedure for the *E* → *Z* isomerization of α,β-unsaturated carbonyl compounds and the *Z* → *E* isomerization of γ-diesters.

An 8 mL screw-cap vial was charged with suitable starting material (0.5 mmol, 1 equiv.) and a stirring bar followed by a combination of DCM (4 mL) and 9-fluorenone (4.5 mg, 5 mol%) or MeCN (4 mL) and (-)-riboflavin (9.4 mg, 5 mol%). The screw-cap vial was tightly capped, inserted to the photoisomerization reactor, constantly irradiated using a stripe of LEDs (white light, 3 m, 18 W, 1.5 A) and allowed to stir over-night. Then, the solvent was evaporated under reduced pressure and the crude mixture containing (*E*)-olefin, (*Z*)-olefin and photosensitizer was purified by column chromatography (pentane/Et_2O, 95:5) to yield the product.

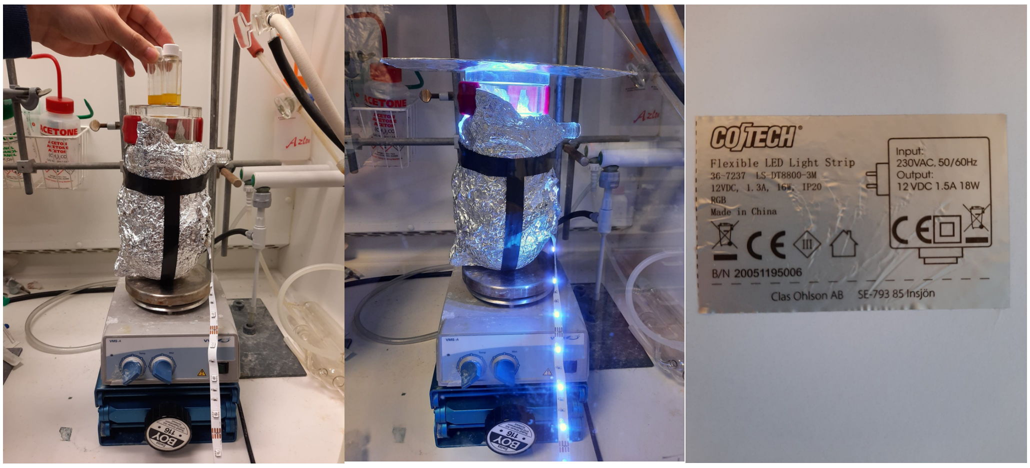

Supplementary Figure 1. Photographs of the reaction setup for the isomerization reaction and the details of the LED light source.

General procedure A for the synthesis of α,β-unsaturated ketones

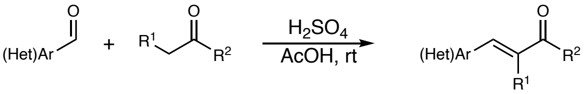

Supplementary Figure 2. General reaction scheme for the acid-catalyzed aldol condensation.

According to reported procedure.[1] Towards a stirring solution of suitable aldehyde (10 mmol, 1.0 equiv.) in a solution of H_2SO_4 (0.5 mL) in acetic acid (10 mL) was added suitable ketone (11 mmol, 1.1 equiv.) and the mixture was allowed to stir at room temperature over-night. Then, Et_2O (20 mL) was added and the reaction mixture was carefully neutralized with aqueous NaOH (4M). The aqueous phase was extracted with Et_2O (3x, 20 mL) and the combined organic phases were washed with $NaHCO_3$ (20 mL), washed with brine (20 mL), dried over Na_2SO_4 and evaporated *in vacuo* to dryness. The crude was purified by column chromatography (pentane/Et_2O, 95:5) to yield the desired α,β-unsaturated ketone.

General procedure B for the synthesis of α,β-unsaturated ketones

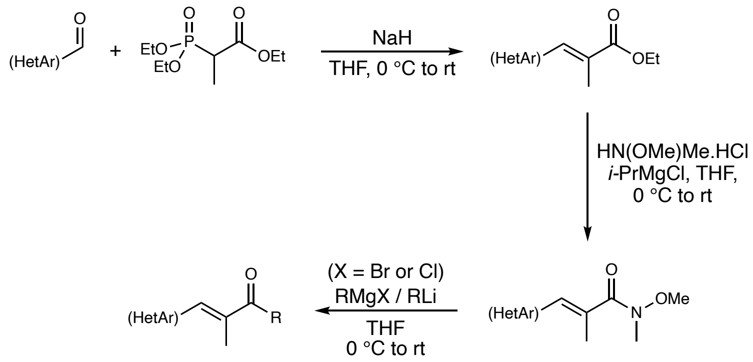

Supplementary Figure 3. General reaction scheme for the synthesis of enones.

According to reported procedure.[1] Towards a stirring suspension of NaH (60% in mineral oil, 0.48 g, 12 mmol, 1.2 equiv.) in THF (50 mL) was added triethyl-2-phosphonopropionate (2.86 g, 12 mmol, 1.2 equiv.) at 0 °C. suitable aldehyde (10 mmol, 1.0 equiv.) was added after stirring for 30 min and the mixture was allowed to stir at room temperature over-night. Then, saturated aqueous NH_4Cl solution (20 mL) was added to quench the reactants and the mixture was extracted with Et_2O (3x, 20 mL). The combined organic phases were washed with brine (20 mL), dried over Na_2SO_4 and evaporated *in vacuo* to dryness to give the crude. The crude was purified by column chromatography (pentane/Et_2O, 95:5) to yield the desired α,β-unsaturated ester.

The isolated α,β-unsaturated ester (10 mmol, 1.0 equiv.) was charged in a flask containing HN(OMe)Me.HCl (1.94 g, 20 mmol, 2.0 equiv.) in THF (50 mL) followed by the dropwise addition of ^{i}PrMgCl (3.0M in THF, 13.3 mL, 40 mmol, 4.0 equiv.) at 0 °C over a time period of 30 min. After stirring at room temperature for 1 h, saturated aqueous NH_4Cl solution (20 mL) was added to quench the reactants and the mixture was extracted with Et_2O (3x, 20 mL). The combined organic phases were washed with brine (20 mL), dried over Na_2SO_4 and evaporated *in vacuo* to dryness to give the crude. The crude was purified by column chromatography (pentane/Et_2O, 70:30) to yield the desired α,β-unsaturated Weinreb amide.

The isolated α,β-unsaturated Weinreb amide (10 mmol, 1.0 equiv.) was dissolved in THF (50 mL) followed by the dropwise addition of Suitable Grignard reagent (RMgBr, RMgCl or RLi, 11 mmol, 1.1 equiv.) at 0 °C. After stirring at room temperature for 1 h, saturated aqueous NH_4Cl solution (20 mL) was added to quench the reactants and the mixture was extracted with Et_2O (3x, 20 mL). The combined organic phases were washed with brine (20 mL), dried over Na_2SO_4 and evaporated *in vacuo* to dryness to give the crude. The crude was purified by column chromatography (pentane/Et_2O, 95:5) to yield the desired α,β-unsaturated ketone.

General procedure C for the synthesis of α,β-unsaturated ketones

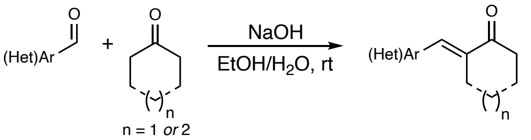

Supplementary Figure 4. General reaction scheme for the base-catalyzed aldol condensation.

According to reported procedure.[1] Towards a stirring solution of suitable aldehyde (10 mmol, 1.0 equiv.) and suitable ketone (20 mmol, 2.0 equiv.) in EtOH (30 mL) at room temperature was added a solution of NaOH (0.48 g, 12 mmol, 1.2 equiv.) in EtOH/H_2O (1:1, 30 mL) and the mixture was allowed to stir over-night. Then, the reaction mixture was carefully neutralized with aqueous HCl (4M), concentrated and extracted with DCM (3x 20 mL). The organic phase was washed with $NaHCO_3$ (20 mL), washed with brine (20 mL), dried over Na_2SO_4, and evaporated *in vacuo* to dryness to give the crude. The crude was purified by column chromatography (pentane/Et_2O, 95:5) to yield the desired α,β-unsaturated ketone.

General procedure for the asymmetric hydrogenation

An oven-dried vial was charged with olefin (0.1 mmol, 1.0 equiv.) and Ir-N,P-catalyst (1.0 mol%). DCM (2 mL) and a magnetic stirring bar were added and the vial was placed in a high-pressure hydrogenation apparatus. The reactor was purged three times with Ar, purged three times with H_2 and then pressurized with H_2 (50-100 bar). The reaction was stirred at room temperature for 16 h before the H_2 pressure was released and the solvent removed under reduced pressure. The residue was purified by flash chromatography (pentane/Et_2O, 50/50) on silica gel to give the alkane. The *ee* value was determined by GC analysis or SFC analysis on chiral stationary phase. The corresponding racemic product was used for comparison and it was prepared on a 0.05 mmol scale using Pd/C (or racemic Ir-catalyst) as catalyst, following the same asymmetric hydrogenation procedure. The absolute configuration was determined by comparing the sign of optical rotation with reported values.

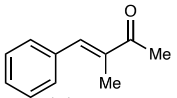

(*E*)-**1a**

(*E*)-3-methyl-4-phenylbut-3-en-2-one (1.52 g, 9.5 mmol, 95% yield) Prepared according to the general procedure A for the synthesis of α,β-unsaturated ketones on a 10 mmol scale. Spectroscopic data was in accordance with reported data.[1]

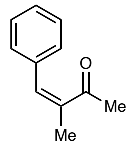

(Z)-**1a** **(*Z*)-3-methyl-4-phenylbut-3-en-2-one** (68.8 mg, 0.43 mmol, 86% yield over 2 steps) Prepared from ethyl (*Z*)-2-methyl-3-phenylacrylate ((*Z*)-**12a**) according to general procedure B for the synthesis of α,β-unsaturated ketones on a 0.5 mmol scale. Spectroscopic data was in accordance with reported data.[2]

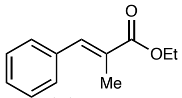

(E)-**1b** **ethyl (*E*)-2-methyl-3-phenylacrylate** (0.35 g, 1.84 mmol, 92% yield) Prepared according to the general procedure B for the synthesis of α,β-unsaturated ketones (step 1) on a 2 mmol scale. Spectroscopic data was in accordance with reported data.[2]

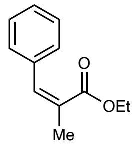

(Z)-**1b** **ethyl (*Z*)-2-methyl-3-phenylacrylate** Prepared according to reported procedure.[3] Towards a stirring solution of KO*t*Bu (2.51 g, 22.4 mmol, 1.2 equiv.) in THF (100 mL) was added ethyl 2-(bis(2-(*tert*-butyl)phenoxy)phosphoryl)propanoate (10.0 g, 22.4 mmol, 1.2 equiv.) in THF (125 mL) at 0 °C. Benzaldehyde (1.9 mL, 18.7 mmol, 1.0 equiv.) was added after stirring for 30 min and the mixture was allowed to stir at room temperature over-night. Then, saturated NH_4Cl solution (70 mL) was added to quench the reactants and the mixture was extracted with Et_2O (3x, 50 mL). The combined organic phases were washed with brine (100 mL), dried over Na_2SO_4 and evaporated *in vacuo* to dryness to give the crude. The crude was purified by column chromatography (pentane/Et_2O, 95:5) to yield the title compound (0.53 g, 2.8 mmol, 15% yield). Spectroscopic data was in accordance with reported data.[2]

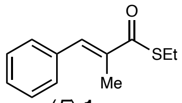

(E)-**1c** ***S*-ethyl (*E*)-2-methyl-3-phenylprop-2-enethioate** Prepared from (*E*)-2-methyl-3-phenylacrylic acid ((*E*)-**12g**) according to reported procedure.[9] Towards a stirring solution of carboxylic acid (0.49 g, 3.0 mmol, 1.0 equiv.) and DMAP (0.37 g, 0.3 mmol, 0.1 equiv.) in DCM (30 mL) was added EDCI.HCl (0.86 g, 4.5 mmol, 1.5 equiv.) and ethanethiol (0.28 mL, 3.9 mmol, 1.3 equiv.) at 0 °C after which the mixture was allowed to stir at room temperature for 3 h. Then, water (20 mL) was added and the organic phase was washed with brine (20 mL), dried over Na_2SO_4 and evaporated *in vacuo* to dryness to give the crude. The crude was purified by column chromatography (pentane/Et_2O, 97:3) to yield the title compound (0.54 g, 2.61 mmol, 87% yield). Spectroscopic data was in accordance with reported data.[4]

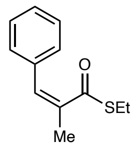

(*Z*)-**1c** ***S*-ethyl (*Z*)-2-methyl-3-phenylprop-2-enethioate** Prepared from (*Z*)-2-methyl-3-phenylacrylic acid ((*E*)-**12g**) according to reported procedure.[4] Towards a stirring solution of carboxylic acid (81.7 mg, 0.5 mmol, 1.0 equiv.) and DMAP (61.7 mg, 0.05 mmol, 0.1 equiv.) in DCM (5 mL) was added EDCI.HCl (0.14g, 0.75 mmol, 1.5 equiv.) and ethanethiol (0.05 mL, 0.65 mmol, 1.3 equiv.) at 0 °C after which the mixture was allowed to stir at room temperature for 3 h. Then, water (5 mL) was added and the organic phase was washed with brine (5 mL), dried over Na_2SO_4 and evaporated *in vacuo* to dryness to give the crude. The crude was purified by column chromatography (pentane/Et_2O, 97:3) to yield the title compound (63.6 mg, 0.31 mmol, 62% yield). **Appearance:** Colorless oil. **^{1}H NMR** (400 MHz, $CDCl_3$) δ7.33 – 7.20 (m, 5H), 6.54 (d, *J* = 1.5 Hz, 1H), 2.88 (q, *J* = 7.4 Hz, 2H), 2.10 (d, *J* = 1.6 Hz, 3H), 1.22 (t, *J* = 7.4 Hz, 3H) ppm. **^{13}C NMR** (100 MHz, $CDCl_3$) δ 198.2, 137.8, 135.8, 131.1, 128.8, 128.3, 128.0, 23.6, 22.3, 14.5 ppm. **HRMS-ESI**: Found $[M+Na]^+$ = 229.0659; $C_{12}H_{14}OSNa$ requires 229.0658.

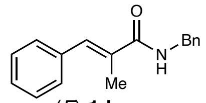

(*E*)-**1d** **(*E*)-*N*-benzyl-2-methyl-3-phenylacrylamide** Prepared from (*E*)-2-methyl-3-phenylacrylic acid ((*E*)-**12g**) according to reported procedure.[5] The carboxylic acid (0.49 g, 3.0 mmol, 1.0 equiv.) was dissolved in SO_2Cl (5 mL) and refluxed for 1 h after which the solvent was evaporated *in vacuo* and the remaining acyl chloride was dissolved in DCM (20 mL). Then, freshly distilled benzylamine (0.63 mL, 6.0 mmol, 2.0 equiv.) and Et_3N (4.2 mL, 30.0 mmol, 10.0 equiv.) were added at 0 °C and the mixture was allowed to stir at room temperature over-night. The mixture was washed with brine (3x, 30 mL) and the organic phase was evaporated *in vacuo* to dryness to give the crude. The crude was purified by column chromatography (pentane/EtOAc, 80:20) to yield the title compound (0.72 g, 2.7 mmol, 90% yield). Spectroscopic data was in accordance with reported data.[5]

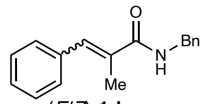

(*E/Z*)-**1d** **(*E*/*Z*)-*N*-benzyl-2-methyl-3-phenylacrylamide** Prepared from (*E*/*Z*)-2-methyl-3-phenylacrylic acid ((*E/Z*)-**12g**) according to reported procedure.[5] The carboxylic acid (0.16 g, 0.5 mmol, 1.0 equiv.) was dissolved in SO_2Cl (5 mL) and refluxed for 1 h after which the solvent was evaporated *in vacuo* and the remaining acyl chloride was dissolved in DCM (5 mL). Then, freshly distilled benzylamine (0.11 mL, 1.0 mmol, 2.0 equiv.) and Et_3N (0.7 mL, 5.0 mmol, 10.0 equiv.) were added at 0 °C and the mixture was allowed to stir at room temperature over-night. The mixture was washed with brine (3x 20 mL) and the organic phase was evaporated *in vacuo* to dryness to give the crude. The crude was purified by column chromatography (pentane/EtOAc, 80:20) and isolated as a mixture of isomeric olefins to yield the title compound (0.11 g, 0.4 mmol, 80% yield).

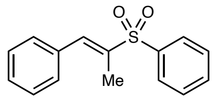

(E)-1e **(*E*)-((1-phenylprop-1-en-2-yl)sulfonyl)benzene** Prepared according to reported procedure.[6] Towards a stirring solution of ethylphenyl sulfone (0.51 g, 3.0 mmol, 1.0 equiv.) in THF (20 mL) was added dropwise *n*-BuLi (2.0M in hexanes, 3.0 mL, 6.0 mmol, 2.0 equiv.) at 0 °C. After 30 min, a solution of was diethyl chlorophosphate (0.43 Ml, 3.0 mmol, 1.0 equiv.) in THF (10 mL) was added dropwise and the mixture was stirred for an additional 30 min at 0 °C. Then, benzaldehyde (0.32 mL, 3.0 mmol, 1.0 equiv.) was added and the mixture was stirred for 1 h at 0 °C before it was quenched with water (10 mL) and extracted with EtOAc (3x, 20 mL). The combined organic phases were washed with brine (20 mL), dried over Na_2SO_4 and evaporated *in vacuo* to dryness to give the crude. The crude was purified by column chromatography (pentane/EtOAc, 90:10) to yield the title compound (0.56 g, 2.2 mmol, 72% yield). Spectroscopic data was in accordance with reported data.[6]

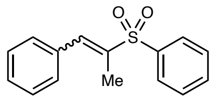

(E/Z)-1e **(*E*/*Z*)-((1-phenylprop-1-en-2-yl)sulfonyl)benzene** Towards a stirring solution of ethylphenyl sulfone (0.17 g, 1.0 mmol, 1.0 equiv.) in THF (20 mL) was added dropwise *n*-BuLi (2.0M in hexanes, 0.5 mL, 1.0 mmol, 1.0 equiv.) at -78 °C and the mixture was allowed to stir at room temperature for 2 h. Then, TMSCl (0.13 mL, 1.0 mmol, 1.0 equiv.) was added and the mixture was stirred for an additional 3 h. The reactants were quenched with water (5 mL) and extracted with Et_2O (3x, 10 mL). The combined organic phases were washed with brine (10 mL), dried over Na_2SO_4 and evaporated *in vacuo* to dryness to give the crude. The crude was purified by column chromatography (pentane/EtOAc, 90:10) to yield the α-trimethylsilyl sulfone (0.22 g, 0.9 mmol, 90% yield).

Towards a stirring solution of the α-trimethylsilyl sulfone (0.22 g, 0.9 mmol, 1.0 equiv.) in THF (10 mL) was added dropwise *n*-BuLi (2.0M in hexanes, 0.55 mL, 1.1 mmol, 1.2 equiv.) at -78 °C and the mixture was allowed to stir at room temperature for 2 h. Then, benzaldehyde (0.11 mL, 1.1 mmol, 1.2 equiv.) was added and the mixture was stirred at room temperature over-night. The mixture was quenched with water (5 mL) and extracted with Et_2O (3x, 5 mL). The combined organic phases were washed with brine (10 mL), dried over Na_2SO_4 and evaporated *in vacuo* to dryness to give the crude. The crude was purified by column chromatography (pentane/Et_2O, 90:10) and isolated as a mixture of isomeric olefins to yield the title compound (0.21 g, 0.81 mmol, 90% yield).

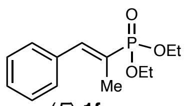

(E)-1f **diethyl (*E*)-(1-phenylprop-1-en-2-yl)phosphonate** Prepared according to reported procedure.[7] Freshly distilled DIPA (1.20 mL, 6.9 mmol, 2.3 equiv.) in THF (10 mL) was slowly added to *n*-BuLi (2.0M in hexanes, 3.1 mL, 6.2 mmol, 2.05 equiv.) in THF (20 mL) at -20 °C and the solution was further cooled to -78 °C. Then, diethyl ethylphosphonate (0.49

mL, 3.0 mmol, 1.0 equiv.) in THF (10 mL) was dropwise added and stirred for 10 min followed by the dropwise addition of TMSCl (0.38 mL, 3.0 mmol, 1.0 equiv.) in THF (10 mL) and the mixture was stirred for an additional 15 min. Thereafter, benzaldehyde (0.33 mL, 3.2 mmol, 1.05 equiv.) in THF (10 mL) was added at -20 °C and the reaction mixture was stirred for 2 h before it was quenched by HCl (5M) to slightly acidic pH and extracted with Et_2O (3x, 40 mL). The combined organic phases were washed with brine (40 mL), dried over Na_2SO_4 and evaporated *in vacuo* to dryness to give the crude. The crude was purified by column chromatography (pentane/Et_2O, 60:40) and isolated as a mixture of isomeric olefins (0.64 g, 2.5 mmol, 84% yield). Isomerically pure vinyl phosphonates were obtained by separation using preparative HPLC using OJ stationary phase (20x250 mm, hexane/*i*-PrOH, 90:10, 3 mL/min, 10 mg loading, 55 min). Spectroscopic data was in accordance with reported data.[8]

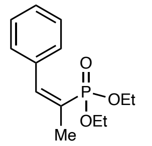

(*Z*)-**1f** **diethyl (*Z*)-(1-phenylprop-1-en-2-yl)phosphonate** Prepared according to reported procedure.[7] Freshly distilled DIPA (1.20 mL, 6.9 mmol, 2.3 equiv.) in THF (10 mL) was slowly added to *n*-BuLi (2.0M in hexanes, 3.1 mL, 6.2 mmol, 2.05 equiv.) in THF (20 mL) at -20 °C and the solution was further cooled to -78 °C. Then, diethyl ethylphosphonate (0.49 mL, 3.0 mmol, 1.0 equiv.) in THF (10 mL) was dropwise added and stirred for 10 min followed by the dropwise addition of TMSCl (0.38 mL, 3.0 mmol, 1.0 equiv.) in THF (10 mL) and the mixture was stirred for an additional 15 min. Thereafter, benzaldehyde (0.33 mL, 3.2 mmol, 1.05 equiv.) in THF (10 mL) was added at -20 °C and the reaction mixture was stirred for 2 h before it was quenched by HCl (5M) to slightly acidic pH and extracted with Et_2O (3x, 40 mL). The combined organic phases were washed with brine (40 mL), dried over Na_2SO_4 and evaporated *in vacuo* to dryness to give the crude. The crude was purified by column chromatography (pentane/Et_2O, 60:40) and isolated as a mixture of isomeric olefins (0.64 g, 2.5 mmol, 84% yield). Isomerically pure vinyl phosphonates were obtained by separation using preparative HPLC using OJ stationary phase (20x250 mm, hexane/*i*-PrOH, 90:10, 3 mL/min, 10 mg loading, 55 min). **Appearance:** Colorless oil. **^{1}H NMR** (400 MHz, $CDCl_3$) δ 7.50 – 7.41 (m, 2H), 7.36 – 7.22 (m, 3H), 7.24 – 7.08 (m, 1H), 4.00 – 3.79 (m, 4H), 2.17 – 2.10 (m, 3H), 1.11 (tt, J = 7.1, 0.7 Hz, 6H) ppm. **^{13}C NMR** (100 MHz, $CDCl_3$) δ 143.8 (d, $^2J_{C\text{-}P}$ = 9.6 Hz), 136.5 (d, $^3J_{C\text{-}P}$ = 7.4 Hz), 129.1 (d, $^4J_{C\text{-}P}$ = 1.9 Hz), 128.1, 127.8, 126.6 (d, $^1J_{C\text{-}P}$ = 175.1 Hz), 61.7 (d, $^3J_{C\text{-}P}$ = 6.2 Hz), 23.2 (d, $^2J_{C\text{-}P}$ = 12.1 Hz), 16.2 (d, $^2J_{C\text{-}P}$ = 6.7 Hz) ppm. **^{31}P NMR** (162 MHz, $CDCl_3$) δ 17.8 ppm. **HRMS-ESI**: Found $[M+Na]^+$ = 277.0972; $C_{13}H_{19}O_3PNa$ requires 277.0964.

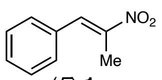

(*E*)-1g **(*E*)-(2-nitroprop-1-en-1-yl)benzene** Prepared according to reported procedure.[9] A mixture of NH_4OAc (0.30 g, 3.9 mmol, 1.3 equiv.) and benzaldehyde (0.31 mL, 3.0 mmol, 1.0 equiv.) in nitroethane (20 mL) was stirred at reflux for 2 h after which the solvent was evaporate *in vacuo* and the residue was dissolved in DCM (10 mL). The organic phase was washed with brine (10 mL), washed with water (10 mL), dried over Na_2SO_4 and evaporated *in vacuo* to dryness to give the crude. The crude was purified by column chromatography (pentane/Et_2O, 95:5) to yield the title compound (0.29 g, 1.8 mmol, 60% yield). Spectroscopic data was in accordance with reported data.[10]

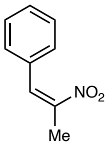

(*Z*)-1g **(*Z*)-(2-nitroprop-1-en-1-yl)benzene** Prepared by isomerization of (*E*)-(2-nitroprop-1-en-1-yl)benzene ((*E*)-**12f**) according to reported procedure.[11] $NaBH_4$ (38.0 mg, 1.0 mmol, 1.2 equiv.) was added towards a stirring solution of diphenyl diselenide (0.19 g, 0.6 mmol, 0.6 equiv.) in EtOH (10 mL) to give a colorless solution. Then, (*E*)-(2-nitroprop-1-en-1-yl)benzene) ((*E*)-**12f**) (0.13 g, 0.83 mmol, 1.0 equiv.) was added at -78 °C, stirred for 1 h, AcOH (0.10 mL, 1.7 mmol, 2.0 equiv.) was added and stirring was continued for an additional 1 h. at room temperature. To the reaction mixture was added water (10 mL), EtOH was evaporated and the aqueous phase was extracted with Et_2O (3x, 10 mL). The combined organic phases were washed with brine (10 mL), dried *in vacuo* to dryness and the residue in DCM (10 mL) was treated with H_2O_2 (30%, 0.15 mL, 1.5 mmol, 1.8 equiv.) at 0 °C for 30 min. The organic phase was washed with saturated Na_2CO_3 (10 mL), dried over Na_2SO_4 and evaporated *in vacuo* to dryness to give the crude. The crude was purified by column chromatography (pentane/Et_2O, 95:5) and isolated as a mixture of isomeric olefins (53.8 mg, 0.33 mmol, 40% yield). Isomerically pure vinyl nitro compounds were obtained by separation using preparative HPLC using OD stationary phase (20x250 mm, hexane/*i*-PrOH, 99:1, 3 mL/min, 30 mg loading, 90 min). Spectroscopic data was in accordance with reported data.[11]

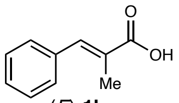

(*E*)-1h **(*E*)-2-methyl-3-phenylacrylic acid** Substrate is commercially available.

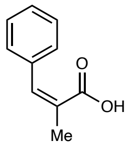

(*Z*)-**1h** **(*Z*)-2-methyl-3-phenylacrylic acid** Prepared from ethyl (*Z*)-2-methyl-3-phenylacrylate ((*Z*)-**12a**) according to reported procedure.[12] LiOH (24.0 mg, 1.0 mmol, 2.0 equiv.) was added towards a solution of (*Z*)-2-methyl-3-phenylacrylate ((*Z*)-**1b**) (95.0 mg, 0.5 mmol, 1.0 equiv.) in a mixture of H_2O/THF/MeOH (3/3/1, 7 mL) and the reaction mixtures was allowed to stir at room temperature over-night. Then, aqueous HCl (1M) was added until neutral pH and the mixture was extracted with Et_2O (3x, 5 mL). The combined organic phases were washed with brine (10 mL), dried over Na_2SO_4 and evaporated *in vacuo* to dryness to give the crude. The crude was purified by column chromatography (pentane/Et_2O, 60:40) to yield the title compound (90.3 mg, 0.47 mmol, 95% yield). Spectroscopic data was in accordance with reported data.[12]

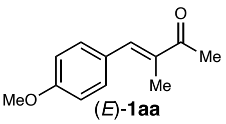

(*E*)-**1aa** **(*E*)-4-(4-methoxyphenyl)-3-methylbut-3-en-2-one** (1.69 g, 8.9 mmol, 89% yield) Prepared according to the general procedure A for the synthesis of α,β-unsaturated ketones on a 10 mmol scale. Spectroscopic data was in accordance with reported data.[1]

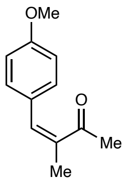

(*Z*)-**1aa** **(*Z*)-4-(4-methoxyphenyl)-3-methylbut-3-en-2-one** (45.6 mg, 0.24 mmol, 48% yield) Prepared by isomerization of (*E*)-4-(4-methoxyphenyl)-3-methylbut-3-en-2-one ((*E*)-**8b**) according to general procedure for the *E* → *Z* isomerization of α,β-unsaturated carbonyl compounds on a 0.5 mmol scale. **Appearance:** Colorless oil. **^{1}H NMR** (400 MHz, $CDCl_3$) δ7.18 – 7.07 (m, 2H), 6.88 – 6.81 (m, 2H), 6.70 (s, 1H), 3.81 (s, 3H), 2.03 (s, 1H), 2.00 (d, J = 1.6 Hz, 1H) ppm. **^{13}C NMR** (100 MHz, $CDCl_3$) δ 207.6, 159.7, 138.6, 132.2, 130.0, 129.2, 114.1, 55.5, 30.1, 21.4 ppm. **HRMS-ESI**: Found $[M+Na]^+$ = 213.0887; $C_{12}H_{14}O_2Na$ requires 213.0886.

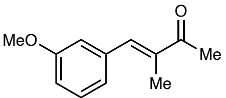

(*E*)-**1ab** **(*E*)-4-(3-methoxyphenyl)-3-methylbut-3-en-2-one** (1.81 g, 9.5 mmol, 95% yield) Prepared according to the general procedure A for the synthesis of α,β-unsaturated ketones on a 10 mmol scale. Spectroscopic data was in accordance with reported data.[1]

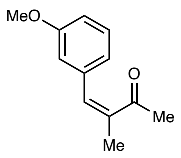

(*Z*)-**1ab** **(*Z*)-4-(3-methoxyphenyl)-3-methylbut-3-en-2-one** (41.8 mg, 0.22 mmol, 44% yield) Prepared by isomerization of (*E*)-4-(3-methoxyphenyl)-3-methylbut-3-en-2-one ((*E*)-**8g**) according to general procedure for the *E* → *Z* isomerization of α,β-unsaturated carbonyl compounds on a 0.5 mmol scale. **Appearance:** Yellow oil. **^{1}H NMR** (400 MHz, $CDCl_3$) δ 7.22 (t, *J* = 7.9 Hz, 1H), 6.83 (dd, *J* = 8.3, 2.6 Hz, 1H), 6.77 (d, *J* = 7.6 Hz, 1H), 6.75 – 6.69 (m, 2H), 3.79 (s, 3H), 2.03 (s, 3H), 2.01 (d, *J* = 1.7 Hz, 3H) ppm. **^{13}C NMR** (100 MHz, $CDCl_3$) δ 207.4, 159.8, 140.3, 138.1, 131.8, 129.7, 121.1, 113.9, 113.8, 55.4, 30.2, 21.30 ppm. **HRMS-ESI**: Found $[M+Na]^+$ = 213.0886; $C_{12}H_{14}O_2Na$ requires 213.0886.

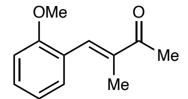

(*E*)-**1ac** **(*E*)-4-(2-methoxyphenyl)-3-methylbut-3-en-2-one** (1.62 g, 8.5 mmol, 85% yield) Prepared according to the general procedure A for the synthesis of α,β-unsaturated ketones on a 10 mmol scale. Spectroscopic data was in accordance with reported data.[13]

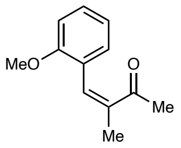

(*Z*)-**1ac** **(*Z*)-4-(2-methoxyphenyl)-3-methylbut-3-en-2-one** (46.6 mg, 0.25 mmol, 49% yield) Prepared by isomerization of (*E*)-4-(2-methoxyphenyl)-3-methylbut-3-en-2-one ((*E*)-**8u**) according to general procedure for the *E* → *Z* isomerization of α,β-unsaturated carbonyl compounds on a 0.5 mmol scale. **Appearance:** Yellow oil. **^{1}H NMR** (400 MHz, $CDCl_3$) δ 7.33 – 7.23 (m, 1H), 7.10 – 7.02 (m, 1H), 6.93 – 6.84 (m, 3H), 3.85 (s, 3H), 2.04 (d, *J* = 1.6 Hz, 3H), 1.98 (s, 3H) ppm. **^{13}C NMR** (100 MHz, $CDCl_3$) δ 206.6, 156.8, 139.7, 130.7, 129.8, 129.4, 125.9, 120.8, 110.7, 55.5, 29.9, 21.4 ppm. **HRMS-ESI**: Found $[M+Na]^+$ = 213.0894; $C_{12}H_{14}O_2Na$ requires 213.0886.

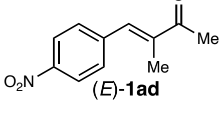

(*E*)-**1ad** **(*E*)-3-methyl-4-(4-nitrophenyl)but-3-en-2-one** (1.85 g, 9.0 mmol, 90% yield) Prepared according to the general procedure A for the synthesis of α,β-unsaturated ketones on a 10 mmol scale. Spectroscopic data was in accordance with reported data.[1]

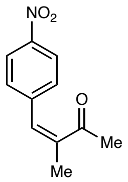
(Z)-**1ad**

(*Z*)-3-methyl-4-(4-nitrophenyl)but-3-en-2-one (41.0 mg, 0.20 mmol, 40% yield) Prepared by isomerization of (*E*)-3-methyl-4-(4-nitrophenyl)but-3-en-2-one ((*E*)-**8f**) according to general procedure for the *E* → *Z* isomerization of α,β-unsaturated carbonyl compounds on a 0.5 mmol scale. **Appearance:** Yellow oil. **^{1}H NMR** (400 MHz, $CDCl_3$) δ 8.17 – 8.03 (m, 1H), 7.57 – 7.43 (m, 1H), 7.26 (s, 2H), 6.65 (s, 1H), 2.09 (s, 3H), 2.09 (d, *J* = 1.6 Hz, 3H) ppm. **^{13}C NMR** (100 MHz, $CDCl_3$) δ 205.9, 142.8, 138.2, 134.3, 129.7, 128.7, 123.3, 122.8, 30.0, 21.5 ppm. **HRMS-ESI**: Found $[M+Na]^+$ = 228.0633; $C_{11}H_{11}NO_3Na$ requires 228.0631.

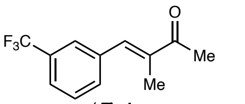
(E)-**1ae**

(*E*)-3-methyl-4-(3-(trifluoromethyl)phenyl)but-3-en-2-one (1.64 g, 7.2 mmol, 72% yield) Prepared according to the general procedure A for the synthesis of α,β-unsaturated ketones on a 10 mmol scale. **Appearance:** Yellow oil. **^{1}H NMR** (400 MHz, $CDCl_3$) δ 7.69 – 7.47 (m, 5H), 2.47 (s, 3H), 2.04 (d, *J* = 1.4 Hz, 3H) ppm. **^{13}C NMR** (100 MHz, $CDCl_3$) δ 200.1, 139.5, 137.8, 136.9, 132.8 (q, $^4J_{C\text{-}F}$ = 2.1 Hz, 1H), 131.2 (q, $^2J_{C\text{-}F}$ = 32.4 Hz), 129.2, 126.5 (q, $^3J_{C\text{-}F}$ = 3.8 Hz), 125.3 (q, $^3J_{C\text{-}F}$ = 3.8 Hz), 124.1 (q, $^1J_{C\text{-}F}$ = 270.6 Hz), 122.8, 26.1, 13.2 ppm. **^{19}F NMR** (377 MHz, $CDCl_3$) δ -62.8 ppm. **HRMS-ESI**: Found $[M+Na]^+$ = 251.0664; $C_{12}H_{11}F_3ONa$ requires 251.0654.

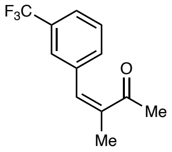
(Z)-**1ae**

(*Z*)-3-methyl-4-(3-(trifluoromethyl)phenyl)but-3-en-2-one (43,3 mg, 0.19 mmol, 38% yield) Prepared by isomerization of (*E*)-3-methyl-4-(3-(trifluoromethyl)phenyl)but-3-en-2-one ((*E*)-**8v**) according to general procedure for the *E* → *Z* isomerization of α,β-unsaturated carbonyl compounds on a 0.5 mmol scale. **Appearance:** Yellow oil. **^{1}H NMR** (400 MHz, $CDCl_3$) δ7.53 (d, *J* = 8.0 Hz, 1H), 7.47 – 7.36 (m, 3H), 6.69 (s, 1H), 2.06 (d, *J* = 1.7 Hz, 3H), 2.04 (s, 3H) ppm. **^{13}C NMR** (100 MHz, $CDCl_3$) δ 206.5, 141.9, 137.4, 131.7 (q, $^4J_{C\text{-}F}$ = 1.4 Hz), 131.2 (q, $^2J_{C\text{-}F}$ = 22.1 Hz), 129.9, 129.2, 125.3 (q, $^3J_{C\text{-}F}$ = 3.7 Hz), 124.8 (q, $^1J_{C\text{-}F}$ = 273.0 Hz), 124.7 (q, $^3J_{C\text{-}F}$ = 3.8 Hz), 30.1, 21.5 ppm. **^{19}F NMR** (377 MHz, $CDCl_3$) δ -62.83 ppm. **HRMS-ESI**: Found $[M+Na]^+$ = 251.0656; $C_{12}H_{11}F_3ONa$ requires 251.0654.

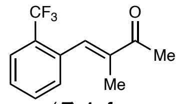

(*E*)-**1af** **(*E*)-3-methyl-4-(2-(trifluoromethyl)phenyl)but-3-en-2-one** (2.01 g, 8.8 mmol, 88% yield) Prepared according to the general procedure A for the synthesis of α,β-unsaturated ketones on a 10 mmol scale. Spectroscopic data was in accordance with reported data.[14]

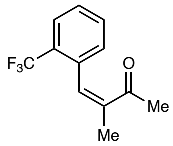

(*Z*)-**1af** **(*Z*)-3-methyl-4-(2-(trifluoromethyl)phenyl)but-3-en-2-one** (39.9 mg, 0.18 mmol, 35% yield) Prepared by isomerization of (*E*)-3-methyl-4-(2-(trifluoromethyl)phenyl)but-3-en-2-one ((*E*)-**8w**) according to general procedure for the *E* → *Z* isomerization of α,β-unsaturated carbonyl compounds on a 0.5 mmol scale. **Appearance:** Yellow oil. **^{1}H NMR** (400 MHz, $CDCl_3$) δ 7.69 (d, J = 7.6 Hz, 1H), 7.50 – 7.37 (m, 2H), 7.23 – 7.19 (m, 1H), 7.00 (s, 1H), 2.06 (d, J = 1.6 Hz, 3H), 1.83 (s, 3H) ppm. **^{13}C NMR** (100 MHz, $CDCl_3$) δ 205.5, 142.1, 135.9 (q, $^4J_{C\text{-}F}$ = 1.9 Hz) 132.0 (q, $^4J_{C\text{-}F}$ =0.7 Hz), 131.5, 129.5 (q, $^4J_{C\text{-}F}$ = 0.7 Hz), 128.3, 127.9 (q, $^2J_{C\text{-}F}$ = 30.0 Hz), 126.2 (q, ^3J = 5.4 Hz), 124.3 (q, $^1J_{C\text{-}F}$ 273.7 Hz), 30.1, 21.1 ppm. **^{19}F NMR** (377 MHz, $CDCl_3$) δ -61.13 ppm. **HRMS-ESI**: Found $[M+Na]^+$ = 251.0651; $C_{12}H_{11}F_3ONa$ requires 251.0654.

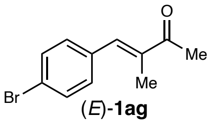

(*E*)-**1ag** **(*E*)-4-(4-bromophenyl)-3-methylbut-3-en-2-one** (2.20 g, 9.3 mmol, 93% yield) Prepared according to the general procedure A for the synthesis of α,β-unsaturated ketones on a 10 mmol scale. Spectroscopic data was in accordance with reported data.[15]

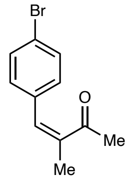

(*Z*)-**1ag** **(*Z*)-4-(4-bromophenyl)-3-methylbut-3-en-2-one** (48.6 mg, 0.21 mmol, 41% yield) Prepared by isomerization of (*E*)-4-(4-bromophenyl)-3-methylbut-3-en-2-one ((*E*)-**8x**) according to general procedure for the *E* → *Z* isomerization of α,β-unsaturated carbonyl compounds on a 0.5 mmol scale. **Appearance:** Colorless oil. **^{1}H NMR** (400 MHz, $CDCl_3$) δ 7.51 – 7.35 (m, 2H), 7.10 – 7.03 (m, 2H), 6.61 (s1H), 2.04 (s, 2H), 2.02 (d, J = 1.7 Hz, 3H) ppm. **^{13}C NMR** (100 MHz, $CDCl_3$) δ 207.0, 140.9, 135.5, 131.9, 130.3, 130.1, 122.2, 30.2, 21.5 ppm. **HRMS-ESI**: Found $[M+Na]^+$ = 260.9885; $C_{11}H_{11}BrONa$ requires 260.9885.

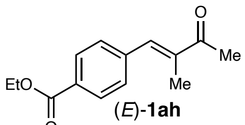

(*E*)-**1ah** **ethyl (*E*)-4-(2-methyl-3-oxobut-1-en-1-yl)benzoate** (1.86 g, 8.0 mmol, 80% yield) Prepared according to the general procedure A for the synthesis of α,β-unsaturated ketones on a 10 mmol scale. **Appearance:** White solid. **^{1}H NMR** (400 MHz, $CDCl_3$) δ 8.10 - 8.05 (m, 2H), 7.53 - 7.50 (m, 1H), 7.48 - 7.44 (m, 2H), 4.39 (q, *J* = 7.1 Hz, 2H), 2.47 (s, 3H), 2.04 (d, *J* = 1.4 Hz, 3H), 1.40 (t, *J* = 7.1 Hz, 3H) ppm. **^{13}C NMR** (100 MHz, $CDCl_3$) δ 200.1, 166.2, 140.5, 139.5, 138.4, 130.3, 129.7, 129.6, 61.3, 26.1, 14.5, 13.2 ppm. **HRMS-ESI**: Found $[M+Na]^+$ = 255.0994; $C_{14}H_{16}O_3Na$ requires 255.0992.

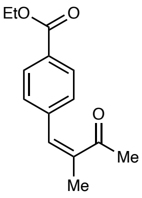

(*Z*)-**1ah** **ethyl (*Z*)-4-(2-methyl-3-oxobut-1-en-1-yl)benzoate** (52.2 mg, 0.23 mmol, 45% yield) Prepared by isomerization of ethyl (*E*)-4-(2-methyl-3-oxobut-1-en-1-yl)benzoate ((*E*)-**8y**) according to general procedure for the *E* → *Z* isomerization of α,β-unsaturated carbonyl compounds on a 0.5 mmol scale. **Appearance:** Yellow oil. **^{1}H NMR** (400 MHz, $CDCl_3$) δ 7.99 (d, *J* = 6.7 Hz, 2H), 7.25 (d, *J* = 7.3 Hz, 2H), 6.70 (s, 1H), 4.37 (q, *J* = 7.1 Hz, 2H), 2.05 (d, *J* = 1.6 Hz, 3H), 2.03 (s, 3H), 1.39 (t, *J* = 7.1 Hz, 3H) ppm. **^{13}C NMR** (100 MHz, $CDCl_3$) δ 206.9, 166.4, 142.0, 141.1, 130.4, 123.0, 129.9, 128.4, 61.3, 30.2, 21.6, 14.5 ppm. **HRMS-ESI**: Found $[M+Na]^+$ = 255.0989; $C_{14}H_{16}O_3Na$ requires 255.0992.

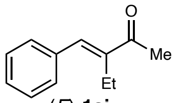

(*E*)-**1ai** **(*E*)-3-benzylidenepentan-2-one** (0.97 g, 5.6 mmol, 56% yield) Prepared according to the general procedure A for the synthesis of α,β-unsaturated ketones on a 10 mmol scale. Spectroscopic data was in accordance with reported data.[1]

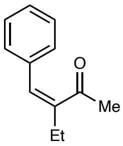

(*Z*)-**1ai** **(*Z*)-3-benzylidenepentan-2-one** (41.8 mg, 0.24 mmol, 48% yield) Prepared by isomerization of (*E*)-3-benzylidenepentan-2-one ((*E*)-**8l**) according to general procedure for the *E* → *Z* isomerization of α,β-unsaturated carbonyl compounds on a 0.5 mmol scale. **Appearance:** Yellow oil. **^{1}H NMR** (400 MHz, $CDCl_3$) δ 7.35 – 7.24 (m, 3H), 7.21 – 7.17 (m, 2H), 6.64 (s, 1H), 2.40 (qd, *J* = 7.4, 1.5 Hz, 2H), 2.02 (s, 3H), 1.12 (t, *J* = 7.4 Hz, 3H) ppm. **^{13}C NMR** (100 MHz, $CDCl_3$) δ 208.3, 146.4, 136.7, 129.4, 128.7, 128.6, 31.1, 28.4, 12.9 ppm. **HRMS-ESI**: Found $[M+Na]^+$ = 197.0943; $C_{12}H_{14}ONa$ requires 197.0937.

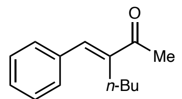

(*E*)-**1aj** **(*E*)-3-benzylideneheptan-2-one** (1.56 g, 7.7 mmol, 77% yield) Prepared according to the general procedure A for the synthesis of α,β-unsaturated ketones on a 10 mmol scale. Spectroscopic data was in accordance with reported data.[16]

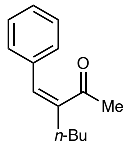

(*Z*)-**1aj** **(*Z*)-3-benzylideneheptan-2-one** (49.5 mg, 0.25 mmol, 49% yield) Prepared by isomerization of (*E*)-3-benzylideneheptan-2-one ((*E*)-**8z**) according to general procedure for the *E* → *Z* isomerization of α,β-unsaturated carbonyl compounds on a 0.5 mmol scale. Spectroscopic data was in accordance with reported data.[17]

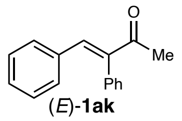

(*E*)-**1ak** **(*E*)-3,4-diphenylbut-3-en-2-one** (1.11 g, 5.0 mmol, 50% yield) Prepared according to the general procedure A for the synthesis of α,β-unsaturated ketones on a 10 mmol scale. Spectroscopic data was in accordance with reported data.[13]

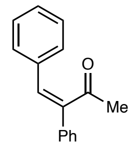

(*Z*)-**1ak** **(*Z*)-3,4-diphenylbut-3-en-2-one** (43.3 mg, 0.20 mmol, 39% yield) Prepared by isomerization of (*E*)-3,4-diphenylbut-3-en-2-one **((*E*)-8aa**) according to general procedure for the *E* → *Z* isomerization of α,β-unsaturated carbonyl compounds on a 0.5 mmol scale. Spectroscopic data was in accordance with reported data.[18]

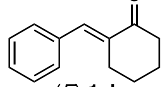

(*E*)-**1al** **(*E*)-2-benzylidenecyclohexan-1-one** (1.60 g, 8.6 mmol, 86% yield) Prepared according to the general procedure C for the synthesis of α,β-unsaturated ketones on a 10 mmol scale. Spectroscopic data was in accordance with reported data.[1]

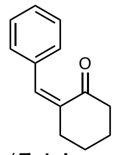

(*Z*)-**1al** **(*Z*)-2-benzylidenecyclohexan-1-one** (38.1 mg, 0.21 mmol, 41% yield) Prepared by isomerization of (*E*)-2-benzylidenecyclohexan-1-one ((*E*)-**8n**) according to general procedure for the *E* → *Z* isomerization of α,β-unsaturated carbonyl compounds on a 0.5 mmol scale.

Appearance: Colorless oil. **^{1}H NMR** (400 MHz, $CDCl_3$) δ 7.39 – 7.10 (m, 5H), 6.40 (s, 1H), 2.60 (ddd, J = 25.4, 10.4, 6.5 Hz, 4H), 2.08 – 1.82 (m, 4H) ppm. **^{13}C NMR** (100 MHz, $CDCl_3$) δ 206.0, 141.0, 135.8, 130.8, 128.9, 128.3, 127.9, 44.6, 38.7, 26.9 ppm. **HRMS-ESI**: Found $[M+Na]^+$ = 209.0937; $C_{13}H_{14}ONa$ requires 209.0937.

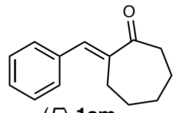

(*E*)-**1am** **(*E*)-2-benzylidenecycloheptan-1-one** (1.62 g, 8.1 mmol, 81% yield) Prepared according to the general procedure C for the synthesis of α,β-unsaturated ketones on a 10 mmol scale. Spectroscopic data was in accordance with reported data.[1]

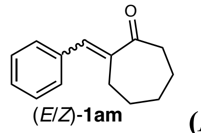

(*E*/*Z*)-**1am** **(*E/Z*)-2-benzylidenecycloheptan-1-one** (96.0 mmol, 96% yield) Prepared by isomerization of (*E*)-2-benzylidenecycloheptan-1-one ((*E*)-**8o**) according to general procedure for the *E* → *Z* isomerization of α,β-unsaturated carbonyl compounds on a 0.5 mmol scale. The crude was purified by column chromatography as a mixture of isomeric olefins to yield the title compound.

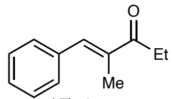

(*E*)-**1an** **(*E*)-2-methyl-1-phenylpent-1-en-3-one** (1.22 g, 7.0 mmol, 70% yield over three steps) Prepared according to the general procedure B for the synthesis of α,β-unsaturated ketones on a 10 mmol scale. Spectroscopic data was in accordance with reported data.[1]

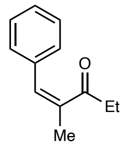

(*Z*)-**1an** **(*Z*)-2-methyl-1-phenylpent-1-en-3-one** (38.3 mg, 0.22 mmol, 44% yield) Prepared by isomerization of (*E*)-2-methyl-1-phenylpent-1-en-3-one ((*E*)-**8ab**) according to general procedure for the *E* → *Z* isomerization of α,β-unsaturated carbonyl compounds on a 0.5 mmol scale. **Appearance:** Colorless oil. **^{1}H NMR** (400 MHz, $CDCl_3$) δ 7.35 – 7.19 (m, 3H), 7.15 (d, J = 7.2 Hz, 2H), 6.64 (s, 1H), 2.29 (q, J = 7.2 Hz, 2H), 2.03 (d, J = 1.4 Hz, 3H), 0.97 (t, J = 7.3 Hz, 3H) ppm. **^{13}C NMR** (100 MHz, $CDCl_3$) δ 211.3, 140.2, 136.7, 130.4, 128.6, 128.4, 127.9, 35.6, 21.8, 8.5 ppm. **HRMS-ESI**: Found $[M+Na]^+$ = 197.0939; $C_{12}H_{14}ONa$ requires 197.0937.

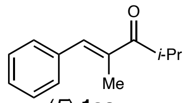

(*E*)-**1ao** **(*E*)-2,4-dimethyl-1-phenylpent-1-en-3-one** (1.67 g, 8.9 mmol, 89% yield) Prepared according to the general procedure A for the synthesis of α,β-unsaturated ketones on a 10 mmol scale. Spectroscopic data was in accordance with reported data.[1]

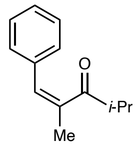

(*Z*)-**1ao** **(*Z*)-2,4-dimethyl-1-phenylpent-1-en-3-one** (37.6 mg, 0.2 mmol, 40% yield) Prepared by isomerization of (*E*)-2,4-dimethyl-1-phenylpent-1-en-3-one ((*E*)-**8j**) according to general procedure for the *E* → *Z* isomerization of α,β-unsaturated carbonyl compounds on a 0.5 mmol scale. **Appearance:** Colorless oil. **^{1}H NMR** (400 MHz, $CDCl_3$) δ 7.35 – 7.20 (m, 3H), 7.18 – 7.09 (m, 2H), 6.67 (s, 1H), 2.49 (dt, *J* = 13.8, 6.9 Hz, 1H), 2.05 (d, *J* = 1.6 Hz, 3H), 0.97 (d, *J* = 6.9 Hz, 6H) ppm. **^{13}C NMR** (100 MHz, $CDCl_3$) δ 214.7, 140.2, 136.8, 130.4, 128.6, 128.5, 127.9, 40.6, 22.7, 18.5 ppm. **HRMS-ESI**: Found $[M+Na]^+$ = 211.1089; $C_{13}H_{16}ONa$ requires 211.1093.

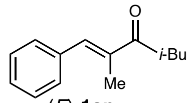

(*E*)-**1ap** **(*E*)-2,5-dimethyl-1-phenylhex-1-en-3-one** (0.85 g, 4.2 mmol, 42% yield over three steps) Prepared according to the general procedure B for the synthesis of α,β-unsaturated ketones on a 10 mmol scale. **Appearance:** Colorless oil. **^{1}H NMR** (400 MHz, $CDCl_3$) δ 7.50 (s, 1H), 7.44 – 7.39 (m, 4H), 7.36 – 7.31 (m, 1H), 2.71 – 2.64 (m, 2H), 2.32 – 2.17 (m, 1H), 2.06 (t, *J* = 2.4 Hz, 3H), 1.01 – 0.95 (m, 6H) ppm. **^{13}C NMR** (100 MHz, $CDCl_3$) δ 202.6, 138.7, 138.1, 136.3, 129.9, 128.6, 128.6, 46.8, 25.9, 23.0, 13.4 ppm. **HRMS-ESI**: Found $[M+Na]^+$ = 225.1246; $C_{14}H_{18}ONa$ requires 225.1250.

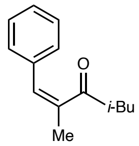

(*Z*)-**1ap** **(*Z*)-2,5-dimethyl-1-phenylhex-1-en-3-one** (47.5 mg, 0.24 mmol, 47% yield) Prepared by isomerization of (*E*)-2,5-dimethyl-1-phenylhex-1-en-3-one ((*E*)-**8ac**) according to general procedure for the *E* → *Z* isomerization of α,β-unsaturated carbonyl compounds on a 0.5 mmol scale. **Appearance:** Colorless oil. **^{1}H NMR** (400 MHz, $CDCl_3$) δ 7.32 – 7.25 (m, 3H), 7.22 – 7.11 (m, 2H), 6.65 (s, 1H), 2.16 (d, *J* = 6.7 Hz, 2H), 2.10 – 1.98 (m, 4H), 0.79 (d, *J* = 6.6 Hz, 6H) ppm. **^{13}C NMR** (100 MHz, $CDCl_3$) δ 210.3, 140.5, 136.6, 130.6, 128.6, 127.9, 51.4, 24.9, 22.7, 21.7 ppm. **HRMS-ESI**: Found $[M+Na]^+$ = 225.1254; $C_{14}H_{18}ONa$ requires 225.1250.

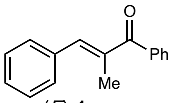

(*E*)-**1aq** **(*E*)-2-methyl-1,3-diphenylprop-2-en-1-one** (1.23 g, 5.5 mmol, 55% yield over three steps) Prepared according to the general procedure B for the synthesis of α,β-unsaturated ketones on a 10 mmol scale. Spectroscopic data was in accordance with reported data.[1]

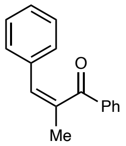

(*Z*)-**1aq** **(*Z*)-2-methyl-1,3-diphenylprop-2-en-1-one** (43.3 mg, 0.2 mmol, 39% yield) Prepared by isomerization of (*E*)-2-methyl-1,3-diphenylprop-2-en-1-one ((*E*)-**8k**) according to general procedure for the *E* → *Z* isomerization of α,β-unsaturated carbonyl compounds on a 0.5 mmol scale. **Appearance:** Colorless oil. **^{1}H NMR** (400 MHz, $CDCl_3$) δ 7.98 – 7.81 (m, 2H), 7.45 (ddt, *J* = 7.3, 6.0, 3.0 Hz, 1H), 7.38 – 7.28 (m, 2H), 7.23 – 7.05 (m, 5H), 6.75 (d, *J* = 1.6 Hz, 1H), 2.18 (d, *J* = 1.6 Hz, 3H) ppm. **^{13}C NMR** (100 MHz, $CDCl_3$) δ 201.2, 137.4, 136.0, 135.4, 133.6, 130.5, 129.6, 128.8, 128.6, 128.4, 127.6, 23.0 ppm. **HRMS-ESI**: Found $[M+Na]^+$ = 245.0934; $C_{16}H_{14}ONa$ requires 245.0937.

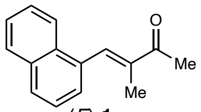

(*E*)-**1ar** **(*E*)-3-methyl-4-(naphthalen-1-yl)but-3-en-2-one** (1.58 g, 7.5 mmol, 75% yield) Prepared according to the general procedure A for the synthesis of α,β-unsaturated ketones on a 10 mmol scale. **Appearance:** Yellow oil. **^{1}H NMR** (400 MHz, $CDCl_3$) δ 8.04 (t, J = 1.4 Hz, 1H), 7.93 – 7.84 (m, 3H), 7.57 – 7.48 (m, 3H), 7.41 (dt, J = 7.1, 1.2 Hz, 1H), 2.58 (s, 3H), 1.93 (d, J = 1.4 Hz, 3H) ppm. **^{13}C NMR** (100 MHz, $CDCl_3$) δ 200.4, 140.1, 138.1, 133.7, 133.3, 131.5, 129.0, 129.0, 126.9, 126.7, 126.4, 125.4, 124.6, 26.3, 13.6 ppm. **HRMS-ESI**: Found $[M+Na]^+$ = 233.0939; $C_{15}H_{14}ONa$ requires 233.0937.

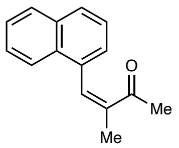

(*Z*)-**1ar** **(*Z*)-3-methyl-4-(naphthalen-1-yl)but-3-en-2-one** (43.1 mg, 0.21 mmol, 41% yield) Prepared by isomerization of (*E*)-3-methyl-4-(naphthalen-1-yl)but-3-en-2-one ((*E*)-**8ad**) according to general procedure for the *E* → *Z* isomerization of α,β-unsaturated carbonyl compounds on a 0.5 mmol scale. **Appearance:** Yellow oil. **^{1}H NMR** (400 MHz, $CDCl_3$) δ 8.00 – 7.96 (m, 1H), 7.91 – 7.86 (m, 1H), 7.83 (dd, J = 8.4, 1.1 Hz, 1H), 7.58 – 7.51 (m, 2H), 7.43 – 7.35 (m, 2H), 7.28 – 7.23 (m, 1H), 2.15 (d, J = 1.6 Hz, 3H), 1.75 (s, 3H) ppm. **^{13}C NMR** (100 MHz, $CDCl_3$) δ 205.8, 134.6, 133.7, 132.4, 131.3 128.9, 128.8, 127.5, 126.7, 162.5, 125.7, 125.0, 30.2, 21.1 ppm. **HRMS-ESI**: Found $[M+Na]^+$ = 233.0928; $C_{15}H_{14}ONa$ requires 233.0937.

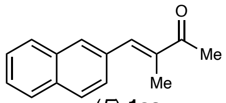

(*E*)-**1as** **(*E*)-3-methyl-4-(naphthalen-2-yl)but-3-en-2-one** (1.68 g, 8.0 mmol, 80% yield) Prepared according to the general procedure A for the synthesis of α,β-unsaturated ketones on a 10 mmol scale. Spectroscopic data was in accordance with reported data.[1]

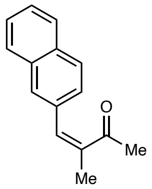

(*Z*)-**1as** **(*Z*)-3-methyl-4-(naphthalen-2-yl)but-3-en-2-one** (44,1 mg, 0.21 mmol, 42% yield) Prepared by isomerization of (*E*)-3-methyl-4-(naphthalen-2-yl)but-3-en-2-one ((*E*)-**8h**) according to general procedure for the *E* → *Z* isomerization of α,β-unsaturated carbonyl compounds on a 0.5 mmol scale. **Appearance:** Yellow oil. **^{1}H-NMR** (400 MHz, $CDCl_3$) δ 7.84 – 7.77 (m, 3H), 7.66 – 7.63 (m, 1H), 7.51 – 7.46 (m, 2H), 7.32 (dd, J = 8.5, 1.8 Hz, 1H), 6.90 (t, J = 1.5 Hz, 1H), 2.09 (d, J = 1.7 Hz, 3H), 2.02 (s, 3H) ppm. **^{13}C NMR** (100 MHz, $CDCl_3$) δ 207.4, 140.4, 134.1, 132.1, 128.3, 128.2, 127.8, 120.8, 126.6, 126.5, 126.2, 30.2, 21.4 ppm. **HRMS-ESI**: Found $[M+Na]^+$ = 233.0929; $C_{15}H_{14}ONa$ requires 233.0937.

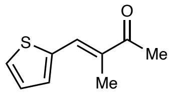

(*E*)-**1at** **(*E*)-3-methyl-4-(thiophen-2-yl)but-3-en-2-one** (1.00 g, 6.0 mmol, 60% yield over three steps) Prepared according to the general procedure B for the synthesis of α,β-unsaturated ketones on a 10 mmol scale. **Appearance:** Colorless oil. **^{1}H NMR** (400 MHz, $CDCl_3$) δ 7.69 (d, J = 1.6 Hz, 1H), 7.54 (dt, J = 5.2, 0.9 Hz, 1H), 7.33 (d, J = 3.7 Hz, 1H), 7.15 (dd, J = 5.1, 3.7 Hz, 1H), 2.45 (s, 3H), 2.16 (d, J = 1.3 Hz, 3H) ppm. **^{13}C NMR** (100 MHz, $CDCl_3$) δ 199.4, 139.6, 132.6, 132.1, 130.0, 127.7, 25.9, 13.1 ppm. **HRMS-ESI**: Found $[M+Na]^+$ = 189.0340; $C_9H_{10}NaOS$ requires 189.0345.

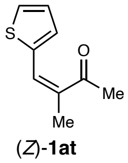

(*Z*)-**1at** **(*Z*)-3-methyl-4-(thiophen-2-yl)but-3-en-2-one** (0.26 mg, 0.16 mmol, 31% yield over two steps) Prepared by isomerization of (*E*)-*N*-methoxy-*N*,2-dimethyl-3-(thiophen-2-yl)acrylamide according to general procedure for the *E* → *Z* isomerization of α,β-unsaturated carbonyl compounds on a 0.5 mmol scale. The isomeric mixture of olefins was separated as α,β-unsaturated ketones. **Appearance:** Colorless oil. **^{1}H NMR** (400 MHz, $CDCl_3$) δ 7.31 (dd, J = 5.0, 1.2 Hz, 1H), 7.08 (dt, J = 3.6, 1.1 Hz, 1H), 6.99 (dd, J = 5.1, 3.6 Hz, 1H), 6.71 (dq, J = 1.8, 0.9 Hz, 1H), 2.26 (s, 3H), 2.10 (d, J = 1.5 Hz, 3H) ppm. **^{13}C NMR** (100 MHz, $CDCl_3$) δ 204.2, 138.8, 134.8, 131.3, 128.2, 127.2, 126.9, 29.5, 22.0 ppm. **HRMS-ESI**: Found $[M+Na]^+$ = 189.0340; $C_9H_{10}NaOS$ requires 189.0345.

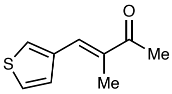

(E)-**1au** **(E)-3-methyl-4-(thiophen-3-yl)but-3-en-2-one** (0.81 g, 4.9 mmol, 49% yield over three steps) Prepared according to the general procedure B for the synthesis of α,β-unsaturated ketones on a 10 mmol scale. **Appearance:** Colorless oil. **^{1}H NMR** (400 MHz, $CDCl_3$) δ 7.52 – 7.49 (m, 2H), 7.38 (dd, J = 5.0, 2.9 Hz, 1H), 7.29 – 7.25 (m, 1H), 2.44 (s, 3H), 2.10 (d, J = 1.3 Hz, 3H) ppm. **^{13}C NMR** (100 MHz, $CDCl_3$) δ 200.2, 137.7, 136.4, 133.6, 129.2, 127.7, 126.1, 26.0, 13.3 ppm. **HRMS-ESI**: Found $[M+Na]^+$ = 189.0352; $C_9H_{10}NaOS$ requires 189.0345.

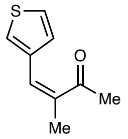

(Z)-**1au** **(Z)-3-methyl-4-(thiophen-3-yl)but-3-en-2-one** (0.27 mg, 0.17 mmol, 33% yield over two steps) Prepared by isomerization of (E)-N-methoxy-N,2-dimethyl-3-(thiophen-3-yl)acrylamide according to general procedure for the E → Z isomerization of α,β-unsaturated carbonyl compounds on a 0.5 mmol scale. The isomeric mixture of olefins was separated as α,β-unsaturated ketones. **Appearance:** Colorless oil. **^{1}H NMR** (400 MHz, $CDCl_3$) δ 7.26 (dd, J = 5.3, 2.6 Hz, 1H), 7.20 (dt, J = 2.8, 1.1 Hz, 1H), 6.99 (dd, J = 5.0, 1.3 Hz, 1H), 6.56 (p, J = 1.5 Hz, 1H), 2.14 (s, 3H), 2.01 (d, J = 1.7 Hz, 3H) ppm. **^{13}C NMR** (100 MHz, $CDCl_3$) δ 207.0, 139.0, 137.5, 128.1, 126.0, 125.7, 124.6, 29.8, 21.2 ppm. **HRMS-ESI**: Found $[M+Na]^+$ = 189.0349; $C_9H_{10}NaOS$ requires 189.0345.

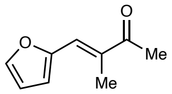

(E)-**1av** **(E)-4-(furan-2-yl)-3-methylbut-3-en-2-one** (0.95 g, 6.3 mmol, 63% yield over three steps) Prepared according to the general procedure B for the synthesis of α,β-unsaturated ketones on a 10 mmol scale. **Appearance:** Colorless oil. **^{1}H NMR** (400 MHz, $CDCl_3$) δ 7.57 (d, J = 1.8 Hz, 1H), 7.28 (t, J = 1.3 Hz, 1H), 6.69 (d, J = 3.5 Hz, 1H), 6.55 – 6.52 (m, 1H), 2.42 (s, 3H), 2.14 (d, J = 1.1 Hz, 3H) ppm. **^{13}C NMR** (100 MHz, CDCl3) δ 199.5, 152.0, 144.6, 134.3, 126.9, 115.5, 112.6, 25.8, 13.0 ppm. **HRMS-ESI**: Found $[M+Na]^+$ = 173.0577; $C_9H_{10}NaO_2$ requires 173.0573.

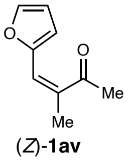

(Z)-**1av** **(Z)-4-(furan-2-yl)-3-methylbut-3-en-2-one** (21.8 mg, 0.15 mmol, 29% yield over two steps) Prepared by isomerization of (E)-3-(furan-2-yl)-N-methoxy-N,2-dimethylacrylamide according to general procedure for the E → Z isomerization of α,β-unsaturated carbonyl compounds on a 0.5 mmol scale. The isomeric mixture of olefins was

separated as α,β-unsaturated ketones. **Appearance:** Yellow oil. **^{1}H NMR** (400 MHz, $CDCl_3$) δ (d, J = 1.8 Hz, 1H), 6.50 (d, J = 3.4 Hz, 1H), 6.38 (dd, J = 3.4, 1.8 Hz, 1H), 6.28 (q, J = 1.7 Hz, 1H), 2.30 (s, 3H), 2.04 (d, J = 1.6 Hz, 3H) ppm. **^{13}C NMR** (100 MHz, $CDCl_3$) δ 206.1, 151.0, 143.1, 136.5, 118.4, 111.8, 111.1, 53.6, 29.5, 21.4 ppm. **HRMS-ESI**: Found $[M+Na]^+$ = 173.0579; $C_9H_{10}NaO_2$ requires 173.0573.

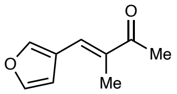

(*E*)-**1aw** **(*E*)-4-(furan-3-yl)-3-methylbut-3-en-2-one** (0.75 g, 5.0 mmol, 50% yield over three steps) Prepared according to the general procedure B for the synthesis of α,β-unsaturated ketones on a 10 mmol scale. **Appearance:** Yellow oil. **^{1}H NMR** (400 MHz, $CDCl_3$) δ 7.69 (s, 1H), 7.48 (t, *J* = 1.5 Hz, 1H), 7.32 (s, 1H), 6.66 – 6.60 (m, 1H), 2.41 (s, 3H), 2.02 (d, *J* = 1.1 Hz, 3H) ppm. **^{13}C NMR** (100 MHz, $CDCl_3$) δ 199.8, 144.4, 143.9, 136.4, 130.5, 122.5, 111.2, 25.8, 13.2 ppm. **HRMS-ESI**: Found $[M+Na]^+$ = 173.0573; $C_9H_{10}NaO_2$ requires 173.0573.

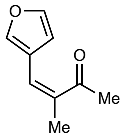

(*Z*)-**1aw** **(*Z*)-4-(furan-3-yl)-3-methylbut-3-en-2-one** (22.5 mg, 0.15 mmol, 30% yield over two steps) Prepared by isomerization of ethyl (*E*)-3-(furan-3-yl)-2-methylacrylate according to general procedure for the *E* → *Z* isomerization of α,β-unsaturated carbonyl compounds on a 0.5 mmol scale. The isomeric mixture of olefins was separated as α,β-unsaturated esters. **Appearance:** Yellow oil. **^{1}H NMR** (400 MHz, $CDCl_3$) δ 7.62 (s, 1H), 7.35 (d, *J* = 1.8 Hz, 1H), 6.45 - 6.38 (m, 1H), 6.31 (d, *J* = 1.7 Hz, 1H), 2.25 (d, *J* = 0.6 Hz, 3H), 2.05 (d, *J* = 1.6 Hz, 3H) ppm. **^{13}C NMR** (100 MHz, $CDCl_3$) δ 205.2, 143.1, 143.0, 136.9, 122.5, 121.6, 111.0, 29.6, 21.54 ppm. **HRMS-ESI**: Found $[M+Na]^+$ = 173.0575; $C_9H_{10}NaO_2$ requires 173.0573.

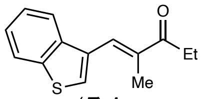

(*E*)-**1ax** **(*E*)-1-(benzo[*b*]thiophen-3-yl)-2-methylpent-1-en-3-one** (0.98 g, 4.3 mmol, 85% yield) Prepared according to the general procedure C for the synthesis of α,β-unsaturated ketones on a 5 mmol scale. Spectroscopic data was in accordance with reported data.[1]

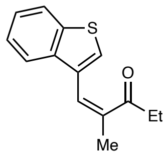

(*Z*)-**1ax** **(*Z*)-1-(benzo[*b*]thiophen-3-yl)-2-methylpent-1-en-3-one** (55.2 mg, 0.24 mmol, 48% yield) Prepared by isomerization of (*E*)-1-(benzo[*b*]thiophen-3-yl)-2-methylpent-1-en-3-one ((*E*)-**8t**) according to general procedure for the *E* → *Z* isomerization of α,β-unsaturated carbonyl compounds on a 0.5 mmol scale. **Appearance:** Colorless oil. **^{1}H NMR** (400 MHz, $CDCl_3$) δ 7.87 (d, *J* = 7.3 Hz, 1H), 7.82 – 7.72 (m, 1H), 7.49 – 7.31 (m, 2H), 7.26 (d, *J* = 1.5 Hz, 1H), 6.77 (s, 1H), 2.30 (q, *J* = 7.3 Hz, 2H), 2.12 (d, *J* = 1.5 Hz, 3H), 0.93 (t, *J* = 7.3 Hz, 3H) ppm. **^{13}C NMR** (100 MHz, $CDCl_3$) δ 210.6, 142.2, 140.0, 138.3, 132.3, 125.3, 125.0, 124.6, 123.1, 122.7, 122.2, 35.3, 21.5, 8.5 ppm. **HRMS-ESI**: Found $[M+Na]^+$ = 253.0658; $C_{14}H_{14}NaOS$ requires 253.0658.

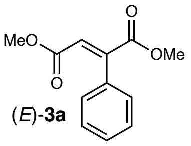

(*E*)-**3a** **dimethyl 2-phenylfurmarate** (79.2 mg, 0.36 mmol, 72% yield) Prepared by isomerization of dimethyl 2-phenylmaleate ((Z)-**14a**) according to general procedure for the *E* → *Z* isomerization of α,β-unsaturated carbonyl compounds on a 0.5 mmol scale. Spectroscopic data was in accordance with reported data.[19]

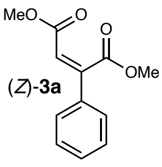

(*Z*)-**3a** **dimethyl 2-phenylmaleate** Prepared according to reported procedure.[19] Towards a stirring solution of cuprous bromide-dimethylsulfide complex (1.03 g, 5.0 mmol, 1.0 equiv.) in THF (30 mL) was added freshly prepared phenylmagnesium bromide (1.0M, 6.5 mL, 6.5 mmol, 1.3 equiv.) at -40 °C and the mixture was allowed to stir for 2 h. Then dimethyl acetylenedicarboxylate (0.61 mL, 5.0 mmol, 1.0 equiv.) was added at -78 °C and stirring was continued for an additional 2 h. Saturated aqueous NH_4Cl solution (20 mL) was added to quench the reactants and the mixture was extracted with Et_2O (3x, 20 mL). The combined organic phases were washed with brine (20 mL), dried over Na_2SO_4 and evaporated *in vacuo* to dryness to give the crude. The crude was purified by column chromatography (pentane/Et_2O, 90:10) to yield the title compound (0.99g, 4.5 mmol, 90% yield). Spectroscopic data was in accordance with reported data.[19]

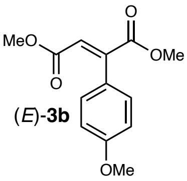

(*E*)-**3b**

dimethyl 2-(4-methoxyphenyl)fumarate (82.5 mg, 0.33 mmol, 66% yield) Prepared by isomerization of dimethyl 2-(4-methoxyphenyl)maleate ((Z)-**14b**) according to general procedure for the *E* → *Z* isomerization of α,β-unsaturated carbonyl compounds on a 0.5 mmol scale. Spectroscopic data was in accordance with reported data.[19]

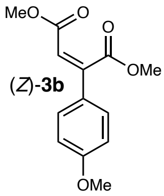

(*Z*)-**3b**

dimethyl 2-(4-methoxyphenyl)maleate Prepared according to reported procedure.[19] Towards a stirring solution of cuprous bromide-dimethylsulfide complex (1.03 g, 5.0 mmol, 1.0 equiv.) in THF (30 mL) was added freshly prepared 4-methoxy phenylmagnesium bromide (1.0M, 6.5 mL, 6.5 mmol, 1.3 equiv.) at -40 °C and the mixture was allowed to stir for 2 h. Then dimethyl acetylenedicarboxylate (0.61 mL, 5.0 mmol, 1.0 equiv.) was added at -78 °C and stirring was continued for an additional 2 h. Saturated aqueous NH_4Cl solution (20 mL) was added to quench the reactants and the mixture was extracted with Et_2O (3x, 20 mL). The combined organic phases were washed with brine (20 mL), dried over Na_2SO_4 and evaporated *in vacuo* to dryness to give the crude. The crude was purified by column chromatography (pentane/Et_2O, 90:10) to yield the title compound (0.99g, 4.0 mmol, 79% yield). Spectroscopic data was in accordance with reported data.[19]

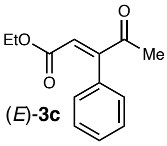

(*E*)-**3c**

ethyl (*E*)-4-oxo-3-phenylpent-2-enoate Prepared according to reported procedure.[20] Towards a stirring solution of 1-phenylpropane-1,2-dione (1.35 mL, 10 mmol, 1.0 equiv.) in EtOAc (100 mL) was added ethyl (triphenylphosphoranylidene)acetate (6.96 g, 20 mmol, 2.0 equiv.) and the mixture was allowed to stir at 100 °C for 3 h after which the mixture was filtered through a silica funnel, once cooled down. The solid compounds remaining on the silica were washed with Et_2O (50 mL) and evaporated *in vacuo* to dryness to give the crude. The complex crude mixture was purified by column chromatography (pentane/EtOAc, 90:10) to yield the title compound (0.17 g, 0.8 mmol, 8% yield). **Appearance:** Colorless oil. **^{1}H NMR** (400 MHz, $CDCl_3$) δ 7.49 – 7.36 (m, 5H), 6.15 (s, 1H), 4.23 (q, *J* = 7.1 Hz, 2H), 2.44 (s, 3H), 1.31 (t, *J* = 7.1 Hz, 3H) ppm. **^{13}C NMR** (100 MHz, $CDCl_3$) δ 204.7, 165.7, 158.4, 133.0, 130.7, 129.4, 127.0, 115.6, 61.2, 30.6, 14.3 ppm. **HRMS-ESI**: Found $[M+Na]^+$ = 241.0828; $C_{13}H_{14}NaO_3$ requires 241.0835.

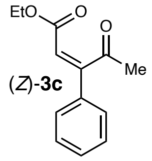

ethyl (*Z*)-4-oxo-3-phenylpent-2-enoate Prepared according to reported procedure.[20] Towards a stirring solution of 1-phenylpropane-1,2-dione (1.35 mL, 10 mmol, 1.0 equiv.) in EtOAc (100 mL) was added ethyl (triphenylphosphoranylidene)acetate (6.96 g, 20 mmol, 2.0 equiv.) and the mixture was allowed to stir at 100 °C for 3 h after which the mixture was filtered through a silica funnel, once cooled down. The solid compounds remaining on the silica were washed with Et_2O (50 mL) and evaporated *in vacuo* to dryness to give the crude. The complex crude mixture was purified by column chromatography (pentane/EtOAc, 90:10) to yield the title compound (0.15 g, 0.7 mmol, 7% yield). **Appearance:** Colorless oil. **^{1}H NMR** (400 MHz, $CDCl_3$) δ 7.44 – 7.34 (m, 3H), 7.17 (dd, *J* = 6.5, 3.2 Hz, 2H), 6.75 (s, 1H), 4.03 (q, *J* = 7.1 Hz, 2H), 2.30 (s, 3H), 1.05 (t, *J* = 7.1 Hz, 3H) ppm. **^{13}C NMR** (100 MHz, $CDCl_3$) δ 199.1, 165.7, 151.0, 134.9, 128.7, 128.7, 128.3, 127.1, 61.1, 28.2, 14.0 ppm. **HRMS-ESI**: Found $[M+Na]^+$ = 241.0836; $C_{13}H_{14}NaO_3$ requires 241.0835.

Characterization of hydrogenated products

Spectroscopic data of compounds **2a**[1] (15.9 mg, 98% yield), **2b**[21] (19.0 mg, 99% yield), **2d**[22] (24.5 mg, 97% yield), **2e**[6] (24.4 mg, 94% yield), **2f**[23] (23.3 mg, 91% yield), **2aa**[1] (19.0 mg, 99% yield), **2ab**[1] (18.6 mg, 97% yield), **2ac**[13] (19.0 mg, 99% yield), **2ad**[1] (20.5 mg, 99% yield), **2ag**[15] (23.9 mg, 99% yield), **2ai**[1] (17.4 mg, 99% yield), **2aj**[1] (20.2 mg, 99% yield), **2ak**[13] (21.5 mg, 96% yield), **2al**[1] (17.7 mg, 94% yield), **2am**[1] (19.2 mg, 95% yield), **2an**[1] (17.3 mg, 98% yield), **2ao**[1] (18.8 mg, 99% yield), **2ap**[24] (19.4 mg, 95% yield), **2aq**[1] (21.5 mg, 96% yield), **2ar**[25] (20.6 mg, 97% yield), **2as**[26] (20.8 mg, 98% yield), **2ax**[1] (20.9 mg, 90% yield), **4a**[19] (20.9 mg, 94% yield), **4b**[19] (23.9 mg, 95% yield) was in accordance with reported data and were all prepared according to the general procedure for the asymmetric hydrogenation on a 0.1 mmol scale

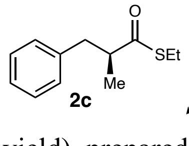

***S*-ethyl (*S*)-2-methyl-3-phenylpropanethioate** Colorless oil (20.0 mg, 96% yield), prepared according to the general procedure for the asymmetric hydrogenation on a 0.1 mmol scale. **^{1}H NMR** (400 MHz, $CDCl_3$) δ 7.31 – 7.24 (m, 3H), 7.23 – 7.13 (m, 2H), 3.07 (dd, *J* = 13.5, 6.6 Hz, 1H), 2.96 – 2.79 (m, 3H), 2.65 (dd, *J* = 13.5, 8.0 Hz, 1H), 1.22 (t, *J* = 7.4 Hz, 3H), 1.16 (d, *J* = 6.9 Hz, 3H) ppm. **^{13}C NMR** (100 MHz, $CDCl_3$) δ 203.5, 139.3, 129.3, 128.6, 126.6, 50.5, 40.1, 23.3, 17.4, 14.9 ppm. **HRMS-ESI**: Found $[M+Na]^+$ = 231.0809; $C_{12}H_{16}NaOS$ requires 231.0814. **$[\alpha]_D^{26}$** = -98.0 (c = 0.1, $CHCl_3$).

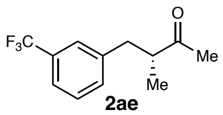

2ae **(*R*)-3-methyl-4-(3-(trifluoromethyl)phenyl)butan-2-one** Colorless oil (22.8 mg, 99% yield), prepared according to the general procedure for the asymmetric hydrogenation on a 0.1 mmol scale. **^{1}H NMR** (400 MHz, $CDCl_3$) δ 7.54 – 7.30 (m, 5H), 3.07 (dd, J = 13.7, 6.9 Hz, 1H), 2.84 (d, J = 7.2 Hz, 1H), 2.61 (dd, J = 13.7, 7.6 Hz, 1H), 2.11 (s, 3H), 1.11 (d, J = 7.0 Hz, 3H) ppm. **^{13}C NMR** (100 MHz, $CDCl_3$) δ 211.5, 140.9, 132.6 (q, $^{4}J_{C\text{-}F}$ = 1.5 Hz), 130.9 (q, $^{2}J_{C\text{-}F}$ = 32.1 Hz), 129.0, 125.7 (q, $^{3}J_{C\text{-}F}$ = 3.8 Hz), 123.3 (q, $^{3}J_{C\text{-}F}$ = 3.8 Hz), 124.3 (q, $^{1}J_{C\text{-}F}$ = 271.0 Hz), 48.7, 38.5, 29.0, 16.5 ppm. **^{19}F NMR** (377 MHz, $CDCl_3$) δ -62.6 ppm. **HRMS-ESI**: Found $[M+Na]^+$ = 253.0803; $C_{12}H_{13}F_3NaO$ requires 253.08011. **$[\alpha]_D^{26}$** = -10.0 (c = 0.1, $CHCl_3$).

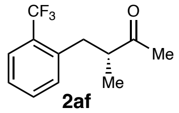

2af **(*R*)-3-methyl-4-(2-(trifluoromethyl)phenyl)butan-2-one** Colorless oil (22.5 mg, 98% yield), prepared according to the general procedure for the asymmetric hydrogenation on a 0.1 mmol scale. **^{1}H NMR** (400 MHz, $CDCl_3$) δ 7.64 (d, J = 7.8 Hz, 1H), 7.45 (t, J = 7.3 Hz, 1H), 7.36 – 7.21 (m, 2H), 3.22 (dd, J = 14.1, 6.7 Hz, 1H), 2.95 – 2.82 (m, 1H), 2.74 (ddd, J = 14.0, 7.4, 1.1 Hz, 1H), 2.11 (s, 3H), 1.11 (d, J = 7.0 Hz, 3H) ppm. **^{13}C NMR** (100 MHz, $CDCl_3$) δ 211.9, 138.7 (q, $^{4}J_{C\text{-}F}$ = 1.8 Hz), 132.1, 131.9 (q, $^{4}J_{C\text{-}F}$ = 1.9 Hz), 128.9 (q, $^{2}J_{C\text{-}F}$ = 29.7 Hz), 126.5 (q, $^{3}J_{C\text{-}F}$ = 5.8 Hz), 124.8 (q, $^{1}J_{C\text{-}F}$ = 271.0 Hz), 48.3, 35.4 (q, $^{4}J_{C\text{-}F}$ = 1.7 Hz), 29.2, 16.7 ppm. **^{19}F NMR** (377 MHz, $CDCl_3$) δ -59.3 ppm. **HRMS-ESI**: Found $[M+Na]^+$ = 253.0820; $C_{12}H_{13}F_3NaO$ requires 253.08011. **$[\alpha]_D^{26}$** = -26.0 (c = 0.1, $CHCl_3$).

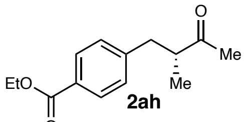

2ah **ethyl (*R*)-4-(2-methyl-3-oxobutyl)benzoate** Colorless oil (22.7 mg, 97% yield), prepared according to the general procedure for the asymmetric hydrogenation on a 0.1 mmol scale. **^{1}H NMR** (400 MHz, $CDCl_3$) δ 8.02 – 7.92 (m, 2H), 7.21 (d, J = 8.3 Hz, 2H), 4.36 (q, J = 7.1 Hz, 2H), 3.05 (dd, J = 13.6, 6.9 Hz, 1H), 2.84 (dd, J = 14.3, 7.1 Hz, 1H), 2.61 (dd, J = 13.6, 7.6 Hz, 1H), 2.09 (s, 3H), 1.38 (t, J = 7.1 Hz, 3H), 1.09 (d, J = 7.0 Hz, 3H) ppm. **^{13}C NMR** (100 MHz, $CDCl_3$) δ 211.7, 166.7, 145.3, 129.9, 129.2, 128.9, 61.1, 48.7, 38.9, 29.1, 16.6, 14.6 ppm. **HRMS-ESI**: Found $[M+Na]^+$ = 257.1158; $C_{14}H_{18}NaO_3$ requires 257.1148. **$[\alpha]_D^{26}$** = +17.0 (c = 0.1, $CHCl_3$).

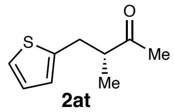

2at **(*R*)-3-methyl-4-(thiophen-2-yl)butan-2-one** Colorless oil (16.5 mg, 98% yield), prepared according to the general procedure for the asymmetric hydrogenation on a 0.1 mmol scale. **^{1}H NMR** (400 MHz, $CDCl_3$) δ 7.12 (dd, J = 5.1, 1.1 Hz, 1H), 6.90 (dd, J = 5.1,

3.4 Hz, 1H), 6.82 – 6.74 (m, 1H), 3.19 (qd, *J* = 5.9, 1.8 Hz, 1H), 2.94 – 2.72 (m, 2H), 2.13 (s, 3H), 1.15 (d, *J* = 6.9 Hz, 3H) ppm. **^{13}C NMR** (100 MHz, $CDCl_3$) δ 211.7, 142.3, 127.0, 125.7, 123.8, 49.3, 32.9, 29.0, 16.6 ppm. **HRMS-ESI**: Found $[M+Na]^+$ = 191.0502; $C_9H_{12}NaOS$ requires 191.0501. **[α]$_D$**^{26} = +15.0 (c = 0.1, $CHCl_3$).

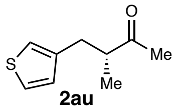

2au

(*R*)-3-methyl-4-(thiophen-3-yl(butan-2-one Colorless oil (16.1 mg, 96% yield), prepared according to the general procedure for the asymmetric hydrogenation on a 0.1 mmol scale. **^{1}H NMR** (400 MHz, $CDCl_3$) δ 7.27 – 7.20 (m, 1H), 6.94 – 6.86 (m, 2H), 3.00 (dd, *J* = 14.1, 7.1 Hz, 1H), 2.90 – 2.76 (m, 1H), 2.64 (dd, *J* = 14.1, 7.2 Hz, 1H), 2.10 (s, 3H), 1.11 (d, *J* = 7.0 Hz, 3H) ppm. **^{13}C NMR** (100 MHz, $CDCl_3$) δ 212.3, 140.1, 128.5, 125.8, 121.7, 48.4, 33.5, 29.0, 16.7 ppm. **HRMS-ESI**: Found $[M+Na]^+$ = 191.0507; $C_9H_{12}NaOS$ requires 191.0501. **[α]$_D$**^{26} = +21.0 (c = 0.1, $CHCl_3$).

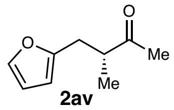

2av

(*R*)-4-(furan-2-yl)-3-methylbutan-2-one Colorless oil (14.1 mg, 93% yield), prepared according to the general procedure for the asymmetric hydrogenation on a 0.1 mmol scale. **^{1}H NMR** (400 MHz, $CDCl_3$) δ 7.29 (dd, *J* = 1.9, 0.8 Hz, 1H), 6.26 (dd, *J* = 3.1, 1.9 Hz, 1H), 6.00 (dd, *J* = 3.2, 0.8 Hz, 1H), 2.94 (ddd, *J* = 20.5, 14.2, 6.9 Hz, 2H), 2.65 (dd, *J* = 14.5, 6.7 Hz, 1H), 2.13 (s, 3H), 1.11 (d, *J* = 7.0 Hz, 3H) ppm. **^{13}C NMR** (100 MHz, $CDCl_3$) δ 211.7, 153.6, 141.5, 110.4, 106.6, 46.2, 31.2, 28.7, 16.5 ppm. **HRMS-ESI**: Found $[M+Na]^+$ = 175.0728; $C_9H_{12}NaO_2$ requires 175.0730. **[α]$_D$**^{26} = +25.0 (c = 0.1, $CHCl_3$).

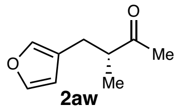

2aw

(*R*)-4-(furan-3-yl)-3-methylbutan-2-one Colorless oil (14.6 mg, 96% yield), prepared according to the general procedure for the asymmetric hydrogenation on a 0.1 mmol scale. **^{1}H NMR** (400 MHz, $CDCl_3$) δ 7.33 (t, *J* = 1.7 Hz, 1H), 7.20 (dd, *J* = 1.5, 0.8 Hz, 1H), 6.30 – 6.16 (m, 1H), 2.75 (tt, *J* = 20.2, 7.0 Hz, 2H), 2.53 – 2.35 (m, 1H), 2.13 (d, *J* = 9.6 Hz, 3H), 1.11 (d, *J* = 7.0 Hz, 3H) ppm. **^{13}C NMR** (100 MHz, $CDCl_3$) δ 212.2, 143.1, 139.9, 122.6, 111.3, 47.9, 28.9, 28.1, 16.6 ppm. **HRMS-ESI**: Found $[M+Na]^+$ = 175.0736; $C_9H_{12}NaO_2$ requires 175.0730. **[α]$_D$**^{26} = +22 (c = 0.1, $CHCl_3$).

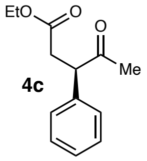
4c

ethyl (*R*)-4-oxo-3-phenylpentanoate Colorless oil (20.0 mg, 91% yield), prepared according to the general procedure for the asymmetric hydrogenation on a 0.1 mmol scale. **^{1}H NMR** (400 MHz, $CDCl_3$) δ 7.37 – 7.25 (m, 3H), 7.24 – 7.18 (m, 2H), 4.18 (dd, J = 9.8, 5.1 Hz, 1H), 4.14 – 4.05 (m, 2H), 3.19 (dd, J = 16.9, 9.8 Hz, 1H), 2.52 (dd, J = 16.9, 5.1 Hz, 1H), 2.11 (s, 3H), 1.21 (t, J = 7.1 Hz, 3H) ppm. **^{13}C NMR** (100 MHz, $CDCl_3$) δ 207.1, 172.3, 137.7, 129.4, 128.5, 127.9, 60.9, 55.1, 37.3, 29.1, 14.3 ppm. **HRMS-ESI**: Found $[M+Na]^+$ = 243.0988; $C_{13}H_{16}O_3Na$ requires 243.0992. **$[\alpha]_D^{26}$** = -331.0 (c = 0.1, $CHCl_3$).

Catalyst optimization

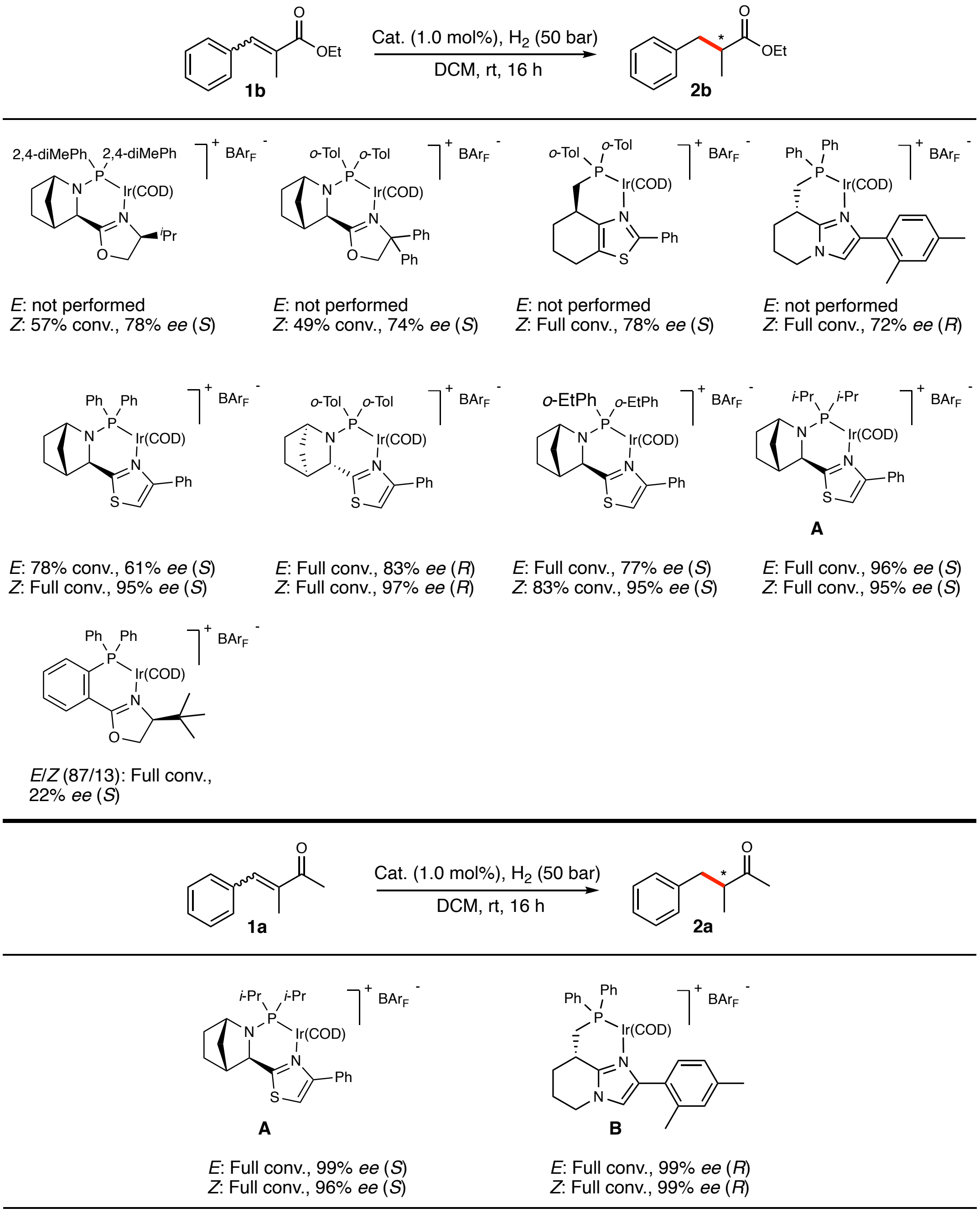

Supplementary Figure 5. Catalyst optimization for the enantioconvergent hydrogenation. Reaction conditions: 0.05 mmol substrate, 1.0 mol% catalyst, 1 mL DCM, 50 bar H_2, 16 h, rt. Conversion was determined by ^{1}H NMR spectroscopy and enantiomeric excess was determined by GC analysis using Hydrodex β-DM chiral stationary phase.

Procedure for the gram-scale hydrogenation

An oven-dried vial was charged with enone **1a** (*E*/*Z* (1/1), 0.998 g, 6.24 mmol, 1.0 equiv.) and Ir-N,P-catalyst **B** (49.5 mg, 0.5 mol%). DCM (10 mL) and a magnetic stirring bar were added and the vial was placed in a high-pressure hydrogenation apparatus. The reactor was purged three times with Ar, purged three times with H_2 and then pressurized with H_2 (50 bar). The reaction was stirred at room temperature for 16 h before the H_2 pressure was released and the solvent removed under reduced pressure. The residue was purified by flash chromatography (pentane/Et_2O, 50/50) on silica gel to give the alkane. The *ee* value was determined by GC analysis using Chiraldex β-DM chiral stationary phase. The corresponding racemic product was used for comparison and it was prepared on a 0.05 mmol scale using Pd/C as catalyst, following the same asymmetric hydrogenation procedure. The absolute configuration was determined by comparing the sign of optical rotation with reported values.

Deuterium gas study

An oven-dried vial was charged with olefin **1aa** (9.5 mg, 0.05 mmol, 1.0 equiv.) and Ir-N,P-catalyst **B** (0.79 mg, 1.0 mol%). DCM (1 mL) and a magnetic stirring bar was added and the vial was placed in a high-pressure hydrogenation apparatus. The reactor was purged three times with Ar, and then filled with D_2 (20 bar). The reaction was stirred at room temperature for overnight before the D_2 pressure was released and the solvent removed under reduced pressure. The residue was purified by flash chromatography (pentane/Et_2O, 50/50) on silica gel to give the deuterated product.

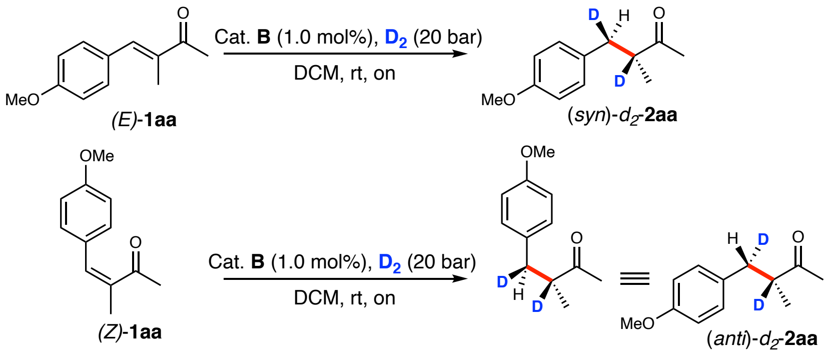

Supplementary Figure 6. D_2-gas hydrogenation of (*E*)-**1aa** and (*Z*)-**1aa**.

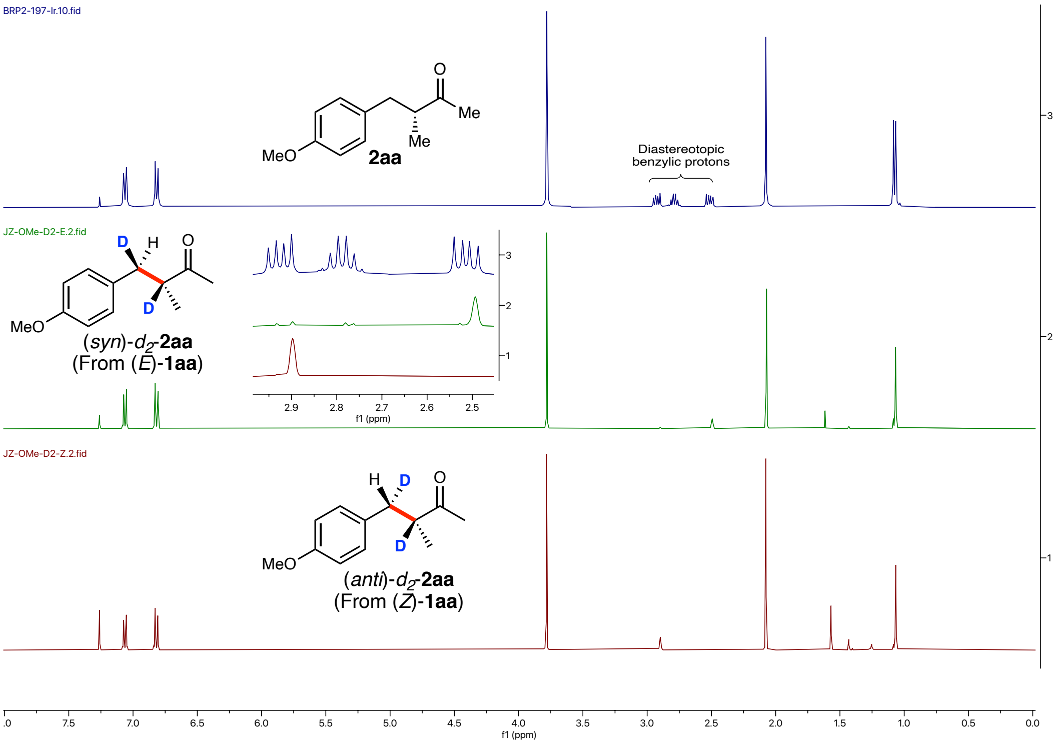

Supplementary Figure 7. ^{1}H NMR (400 MHz, $CDCl_3$) spectra of **2aa** (top), (*syn*)-d_2-**2aa** and (*anti*)-d_2-**2aa** (bottom).

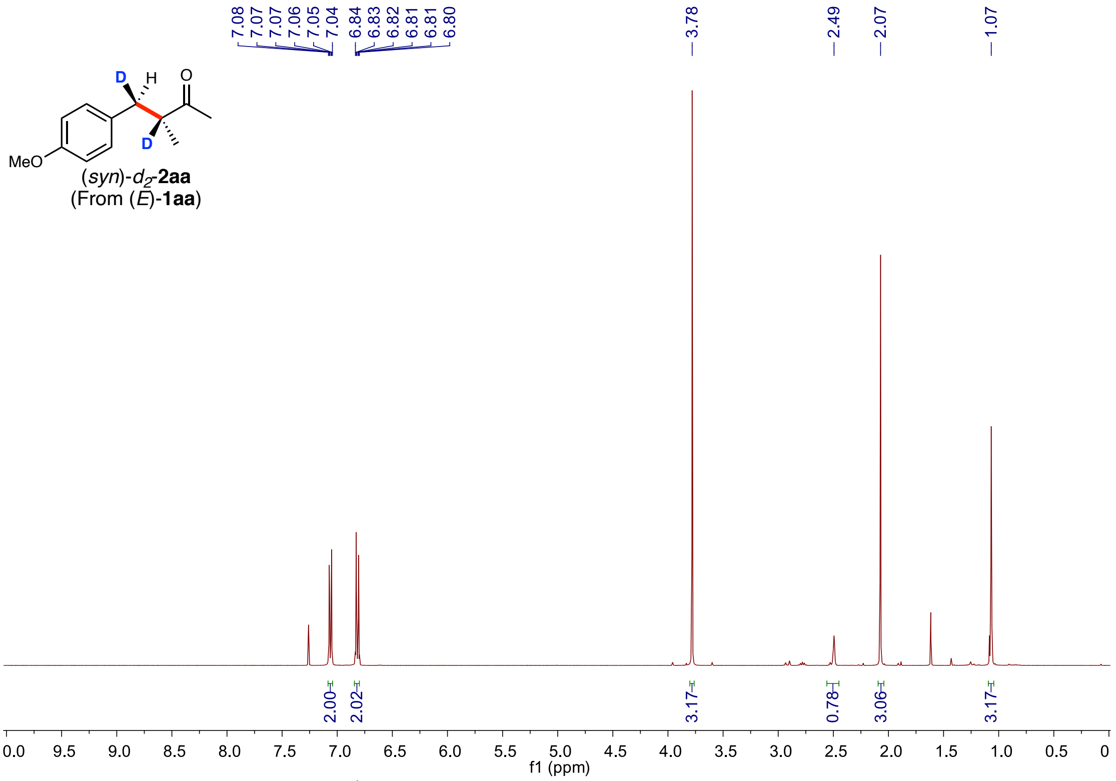

Supplementary Figure 8. ^{1}H NMR (400 MHz, $CDCl_3$) spectrum of (*syn*)-d_2-**2aa**.

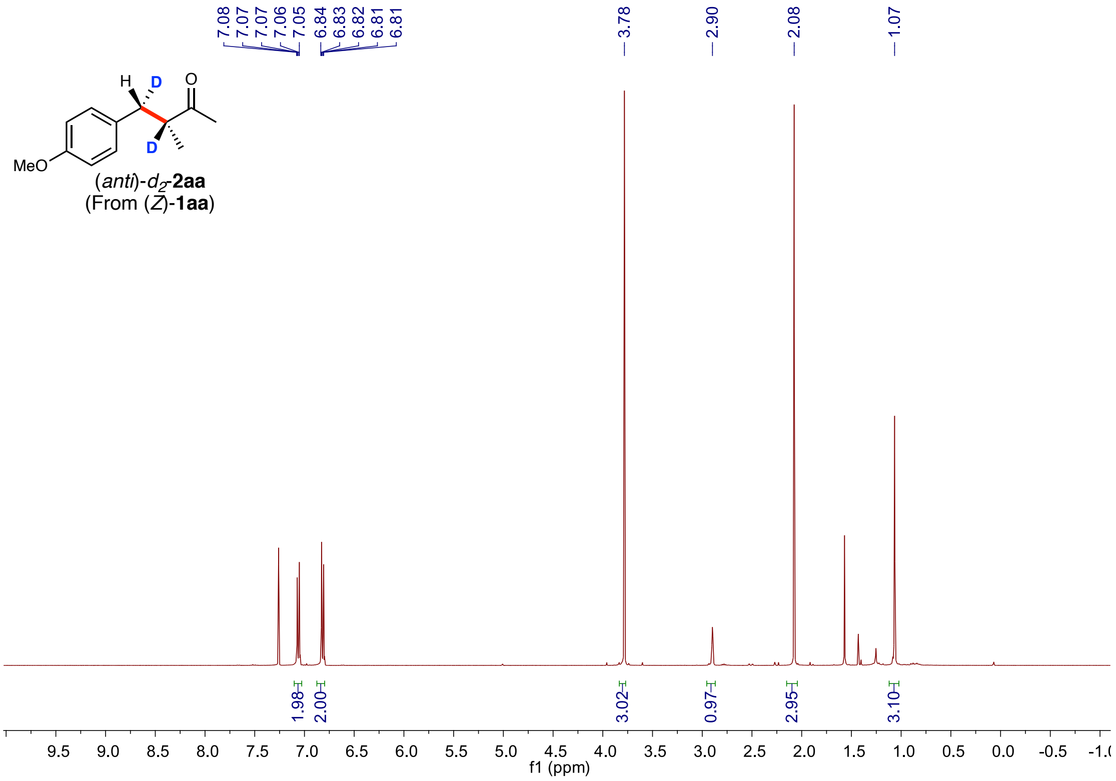

Supplementary Figure 9. ^{1}H NMR (400 MHz, $CDCl_3$) spectrum of (*anti*)-d_2-**2aa**.

NMR Spectra

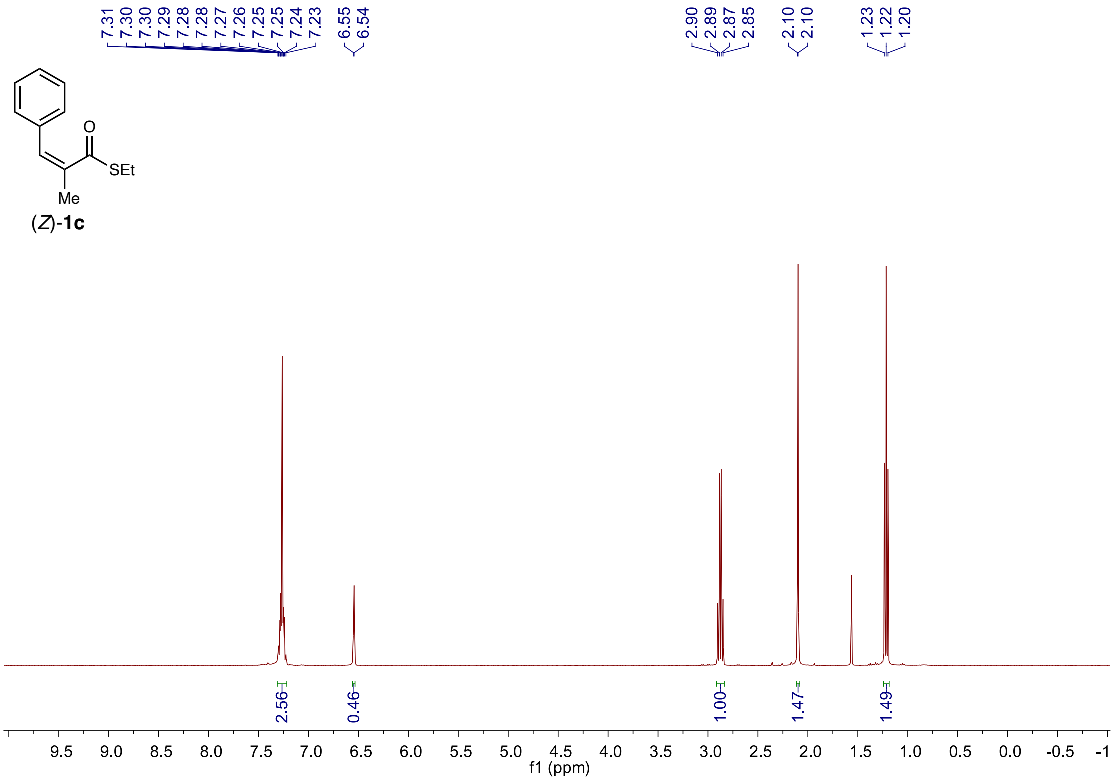

Supplementary Figure 10. ^{1}H NMR (400 MHz, $CDCl_3$) spectrum of (*Z*)-**1c**.

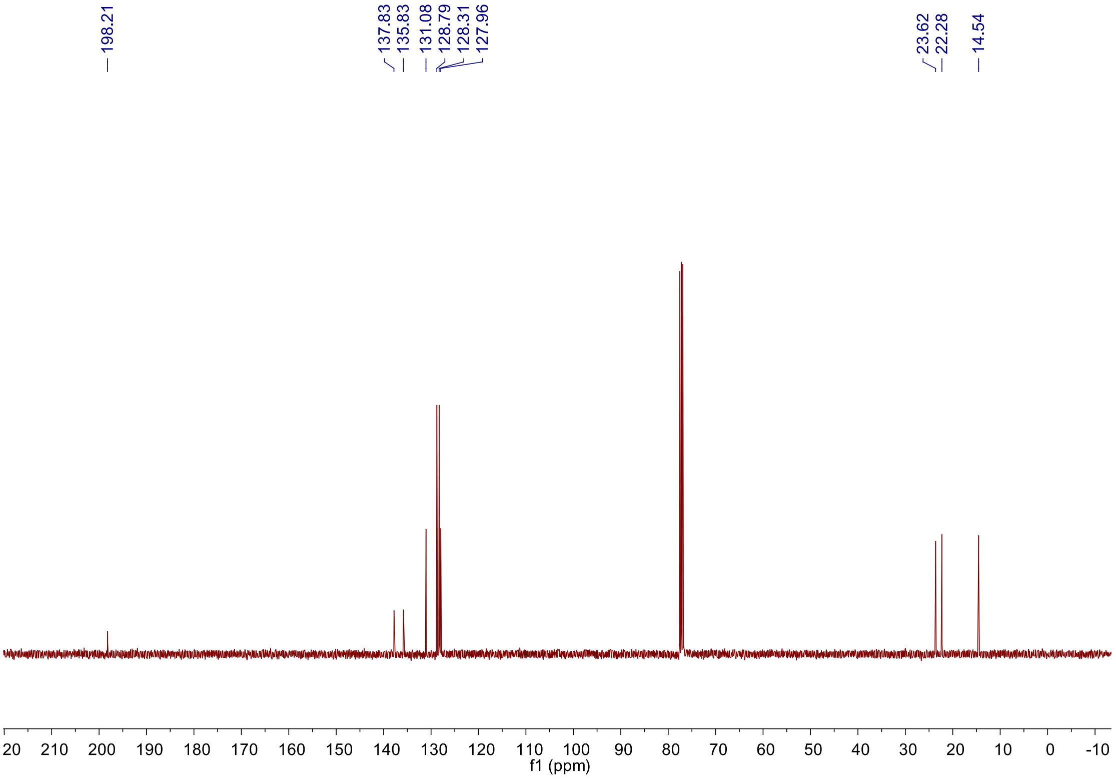

Supplementary Figure 11. ^{13}C NMR (100 MHz, $CDCl_3$) spectrum of (*Z*)-**1c**.

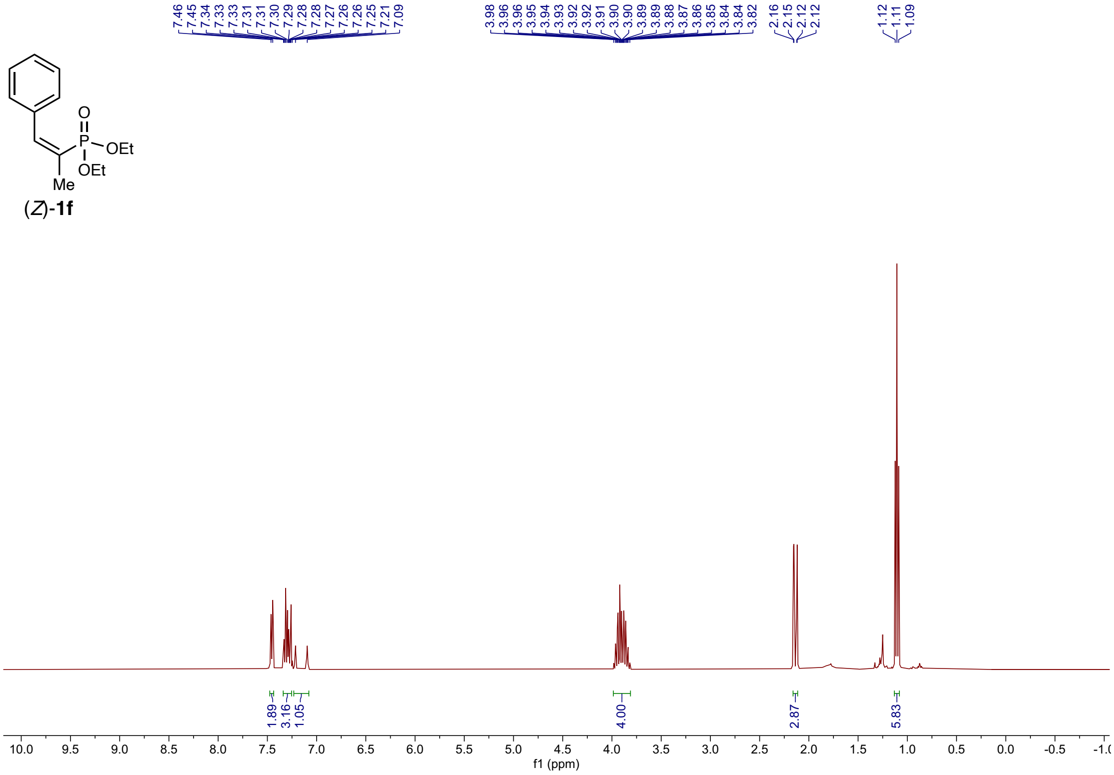

Supplementary Figure 12. ^{1}H NMR (400 MHz, $CDCl_3$) spectrum of (*Z*)-**1f**.

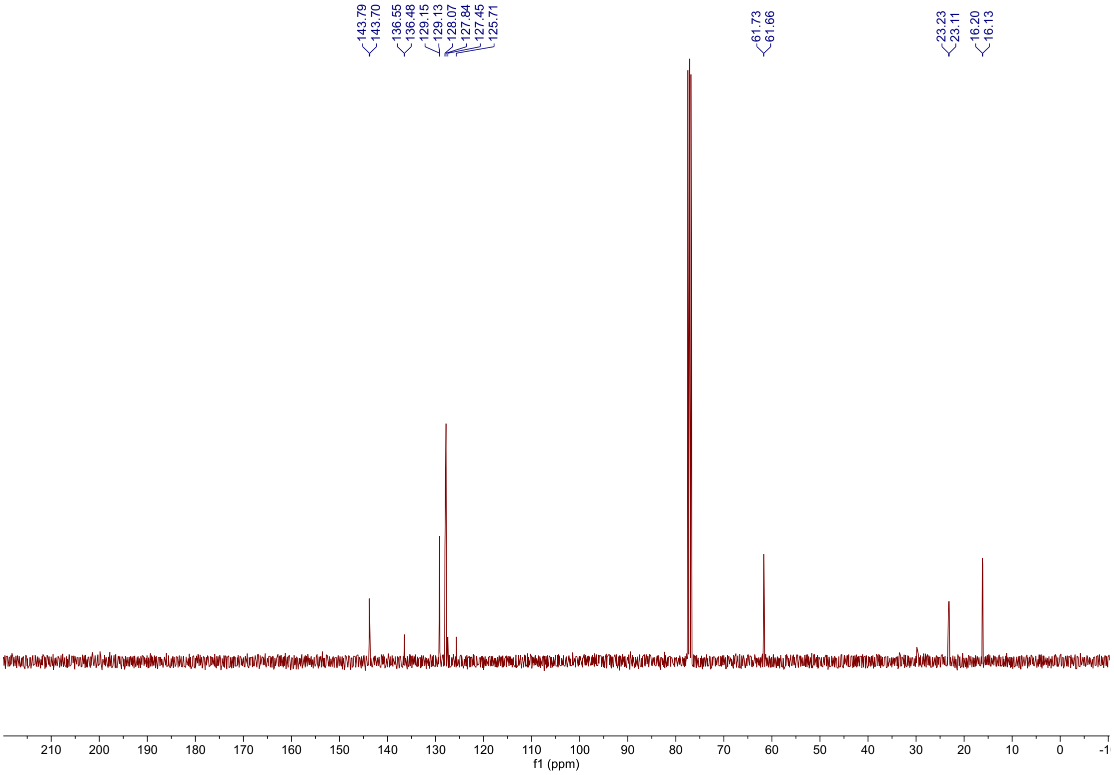

Supplementary Figure 13. ^{13}C NMR (100 MHz, $CDCl_3$) spectrum of (*Z*)-**1f**.

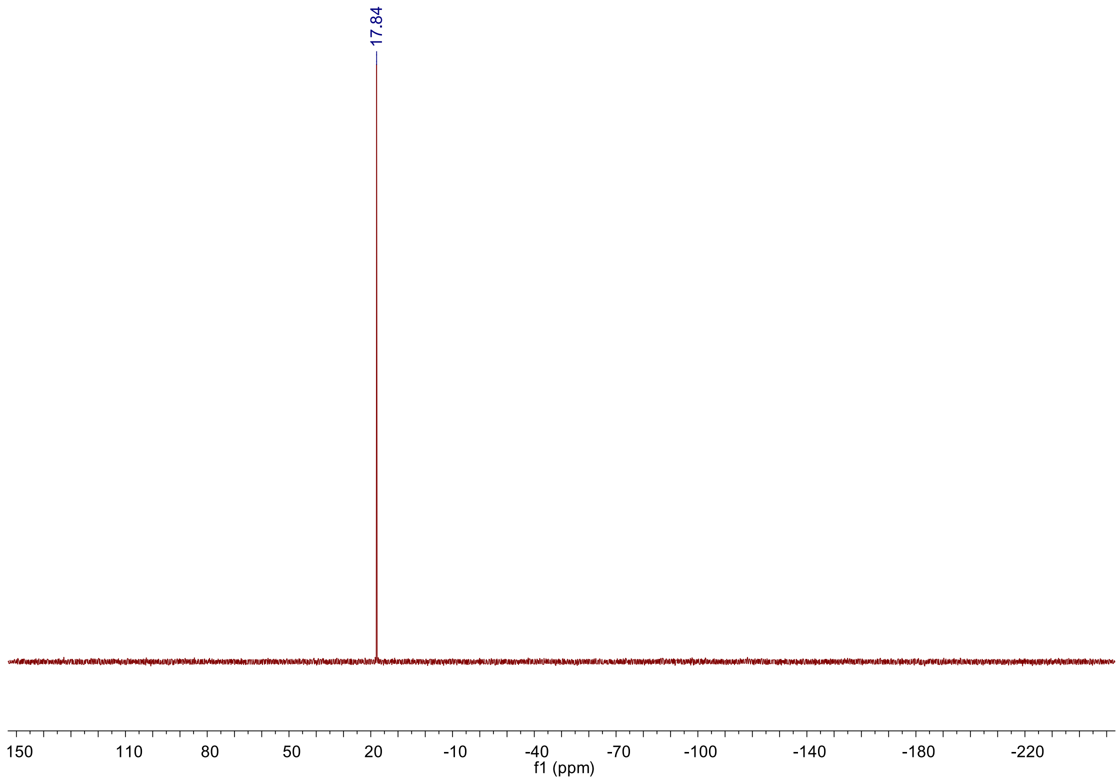

Supplementary Figure 14. ^{31}P NMR (162 MHz, $CDCl_3$) spectrum of (*Z*)-**1f**.

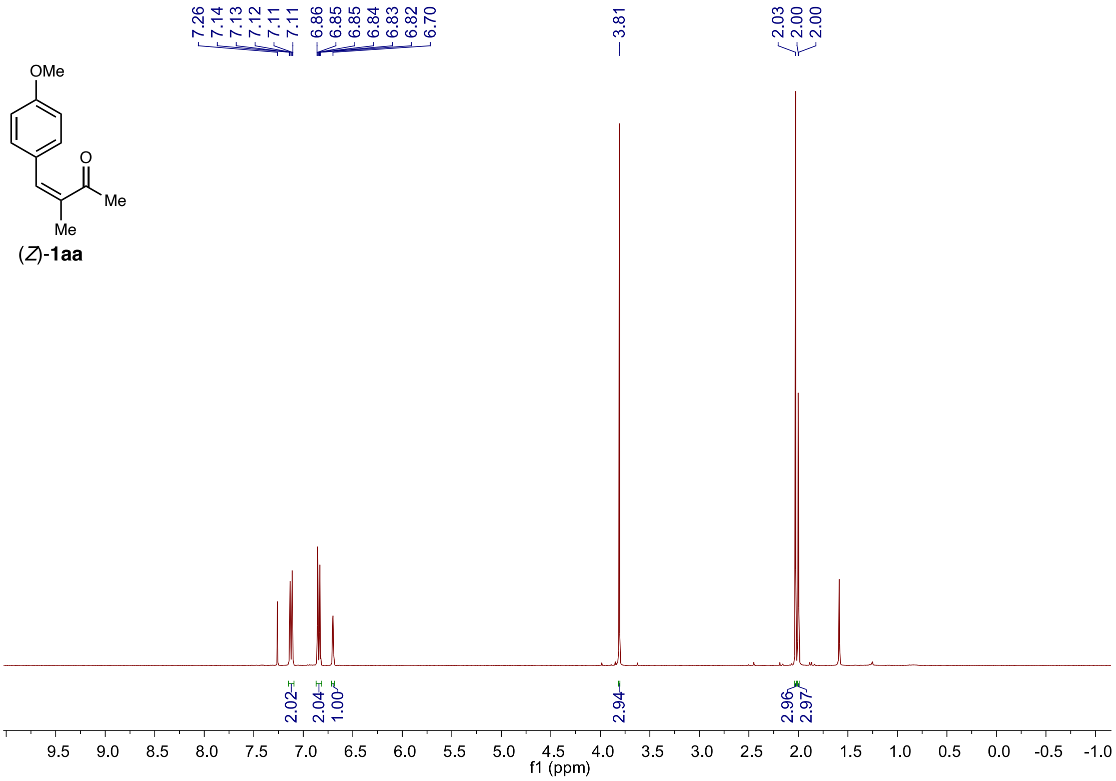

Supplementary Figure 15. ^{1}H NMR (400 MHz, $CDCl_3$) spectrum of (*Z*)-**1aa**.

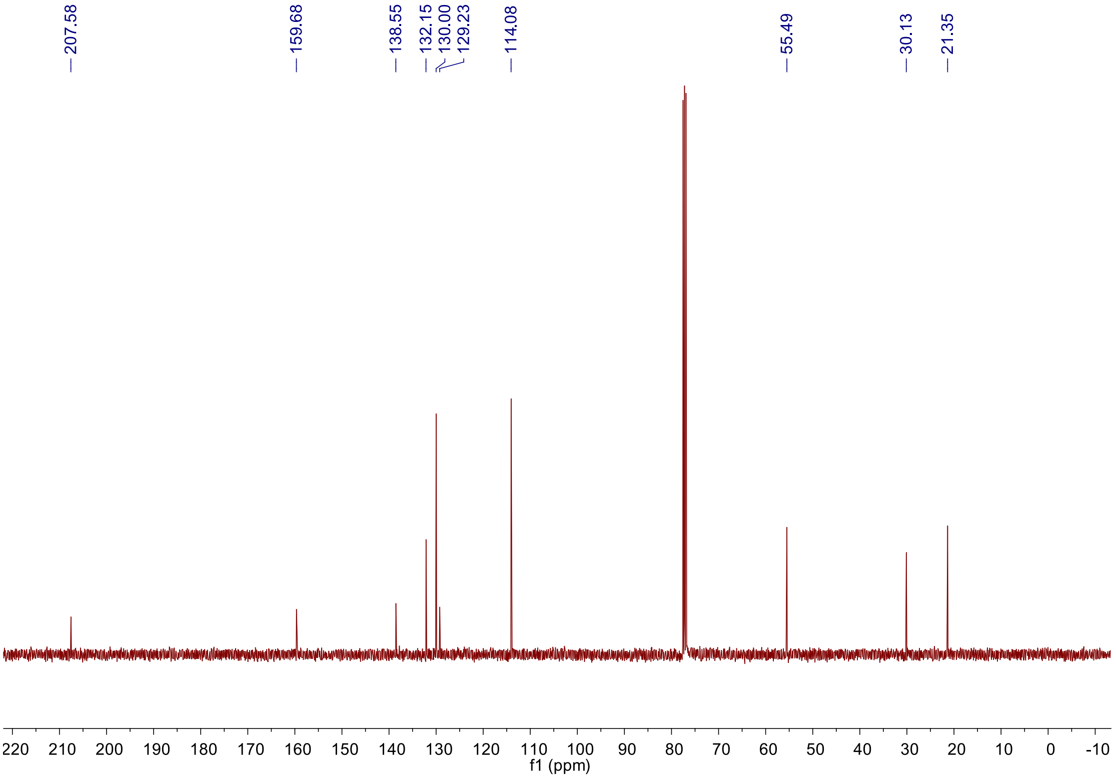

Supplementary Figure 16. ^{13}C NMR (100 MHz, $CDCl_3$) spectrum of (*Z*)-**1aa**.

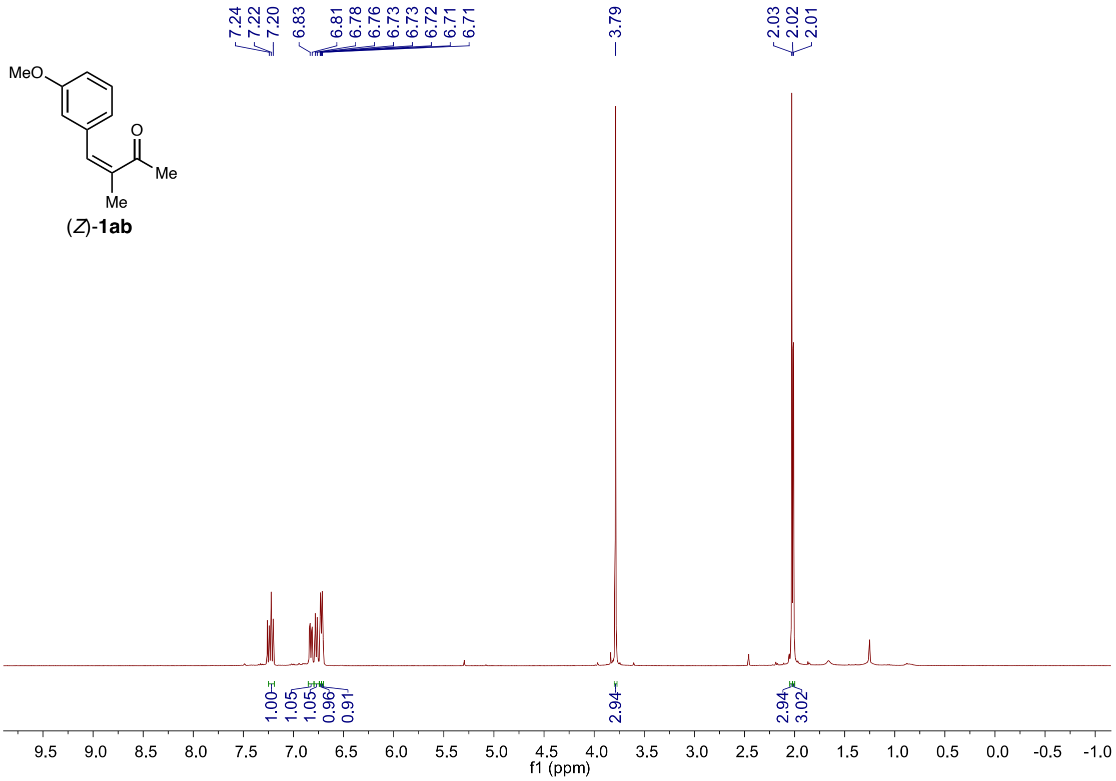

Supplementary Figure 17. ^{1}H NMR (400 MHz, $CDCl_3$) spectrum of (*Z*)-**1ab**.

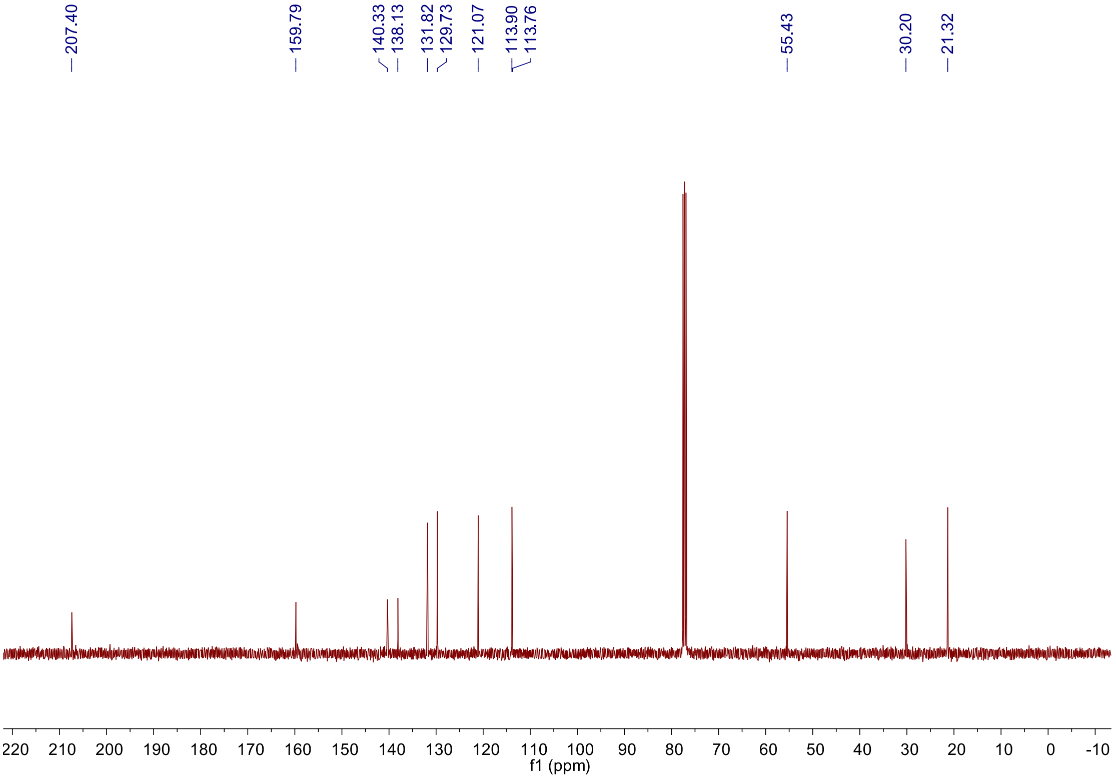

Supplementary Figure 18. ^{13}C NMR (100 MHz, $CDCl_3$) spectrum of (*Z*)-**1ab**.

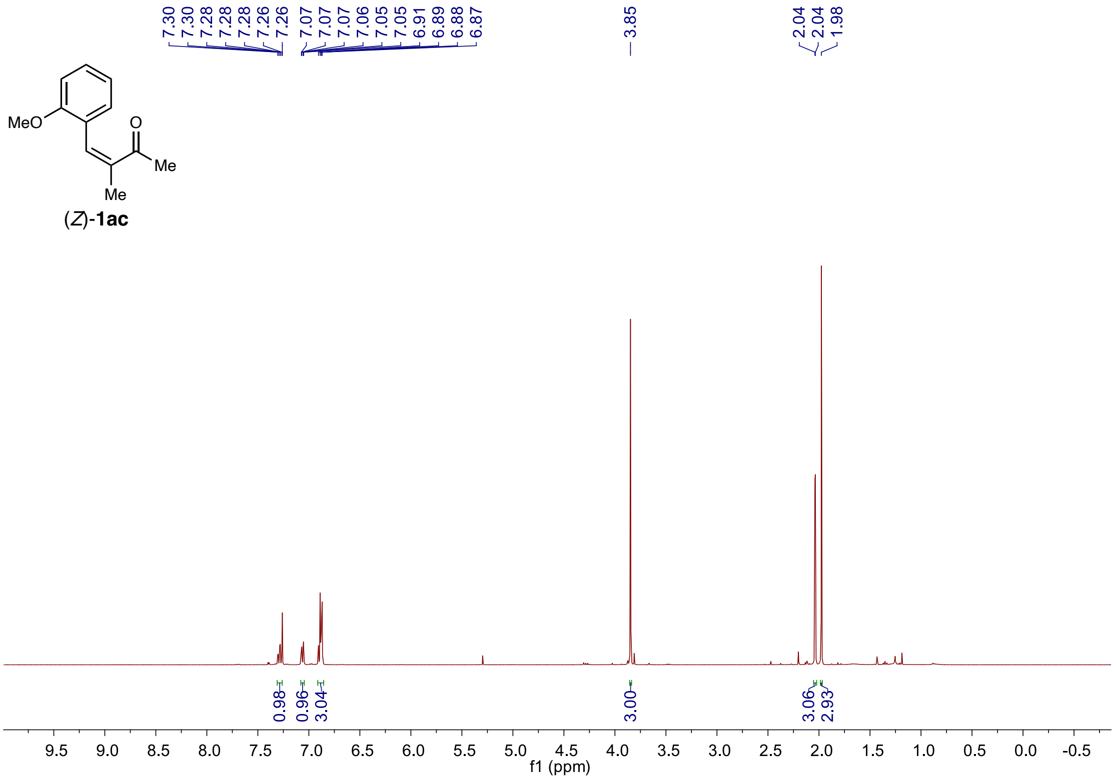

Supplementary Figure 19. ^{1}H NMR (400 MHz, $CDCl_3$) spectrum of (*Z*)-**1ac**.

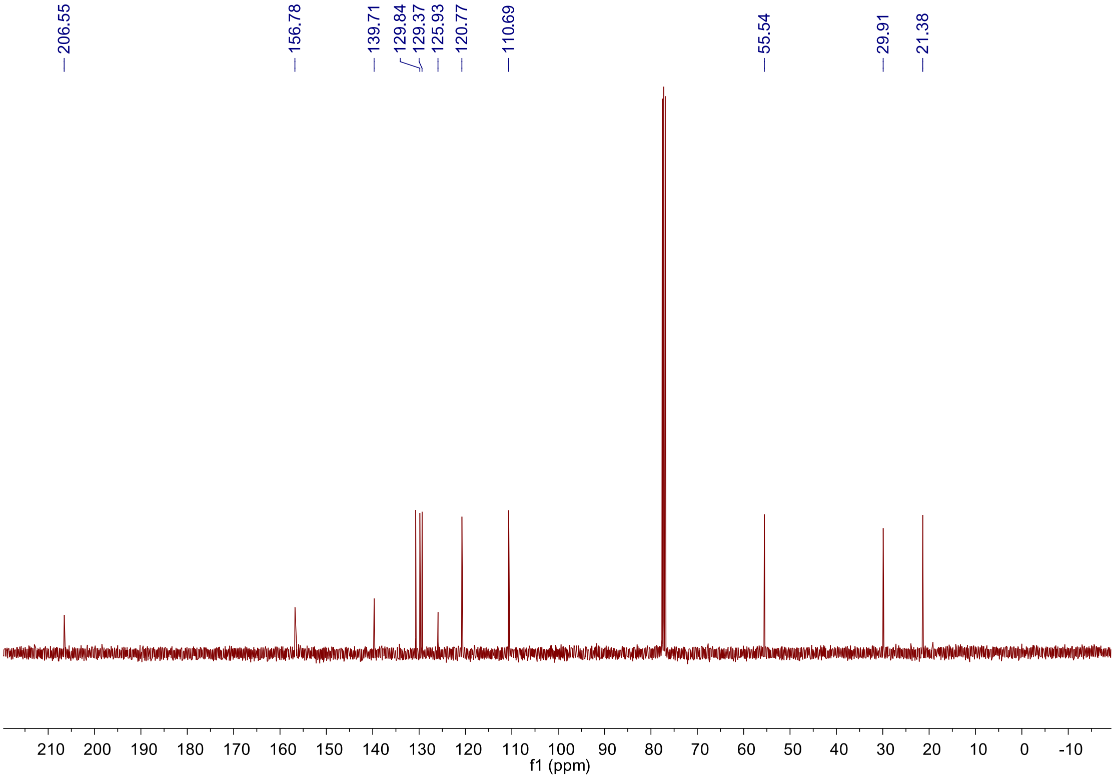

Supplementary Figure 20. ^{13}C NMR (100 MHz, $CDCl_3$) spectrum of (*Z*)-**1ac**.

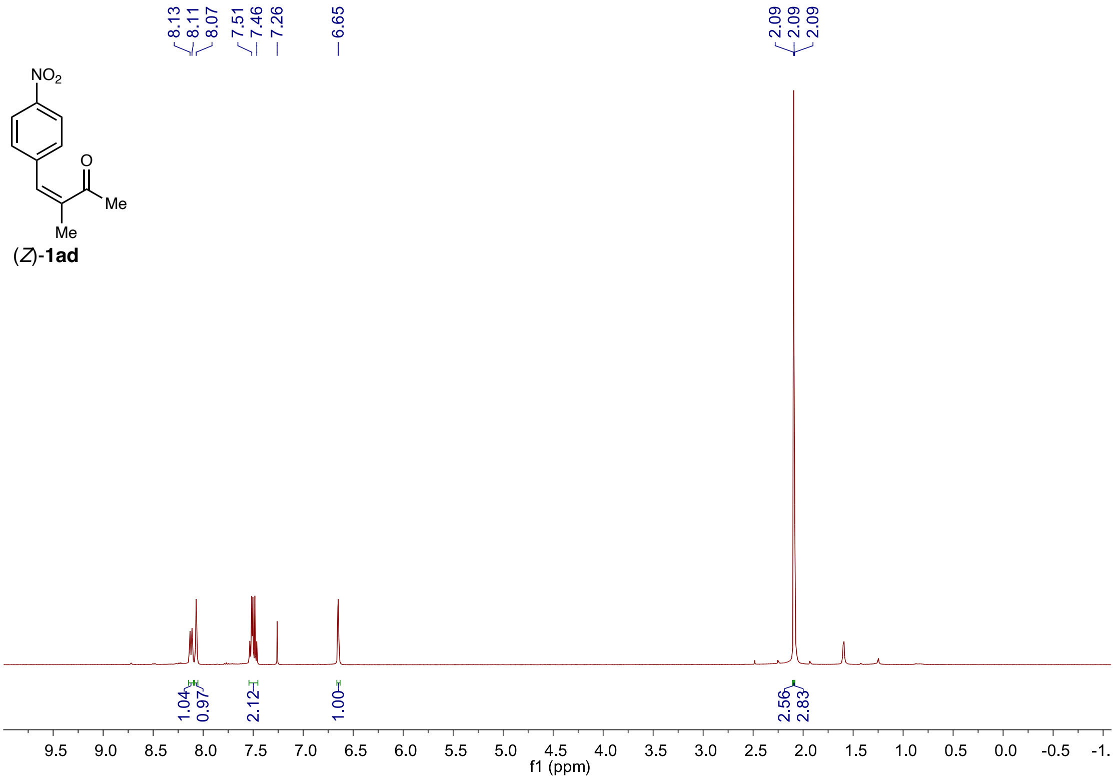

Supplementary Figure 21. ^{1}H NMR (400 MHz, $CDCl_3$) spectrum of (*Z*)-**1ad**.

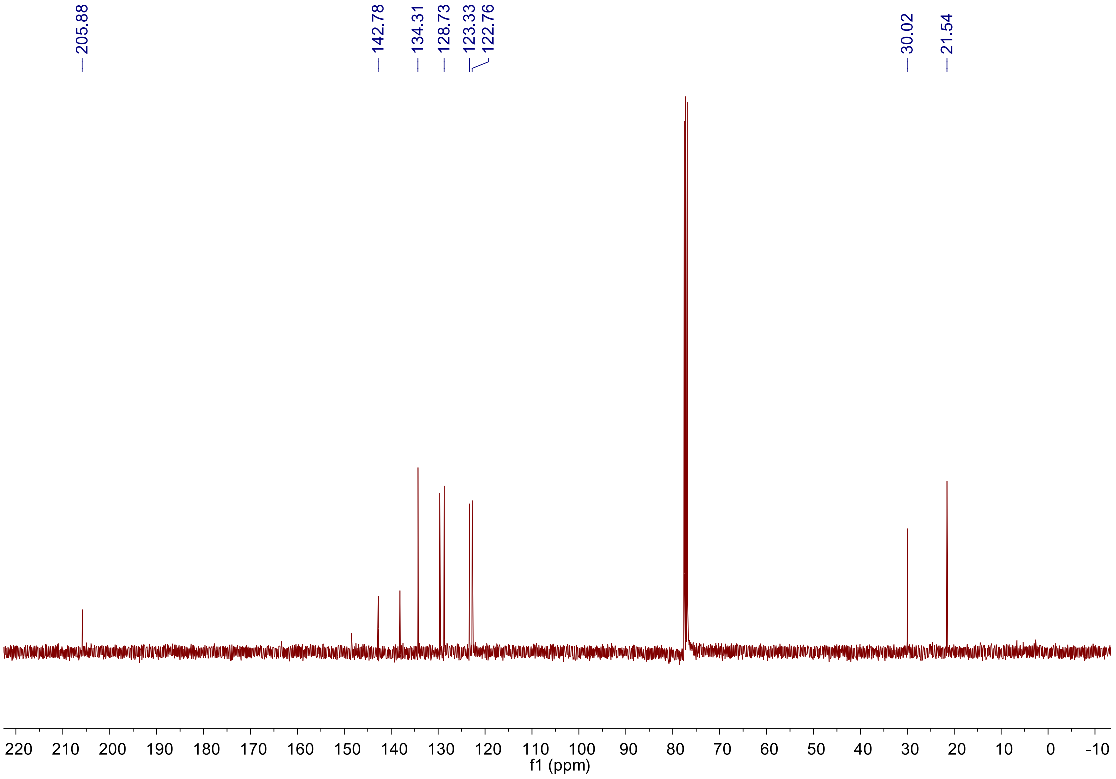

Supplementary Figure 22. ^{13}C NMR (100 MHz, $CDCl_3$) spectrum of (*Z*)-**1ad**.

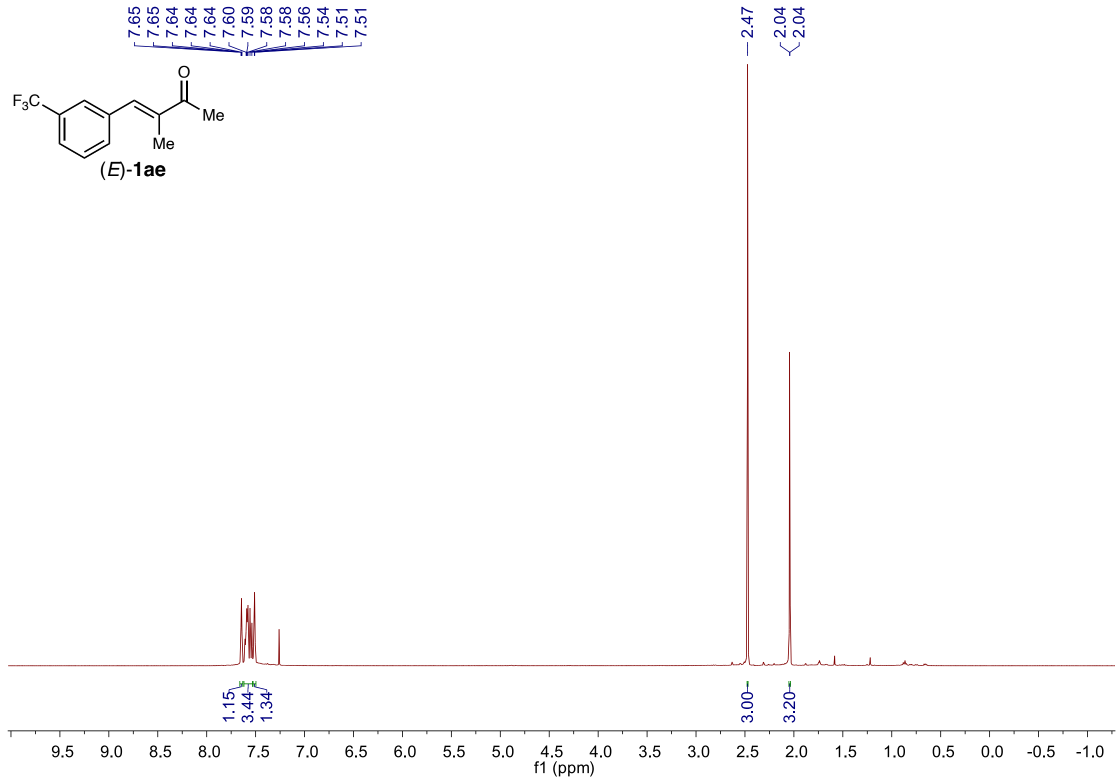

Supplementary Figure 23. ^{1}H NMR (400 MHz, $CDCl_3$) spectrum of (*E*)-**1ae**.

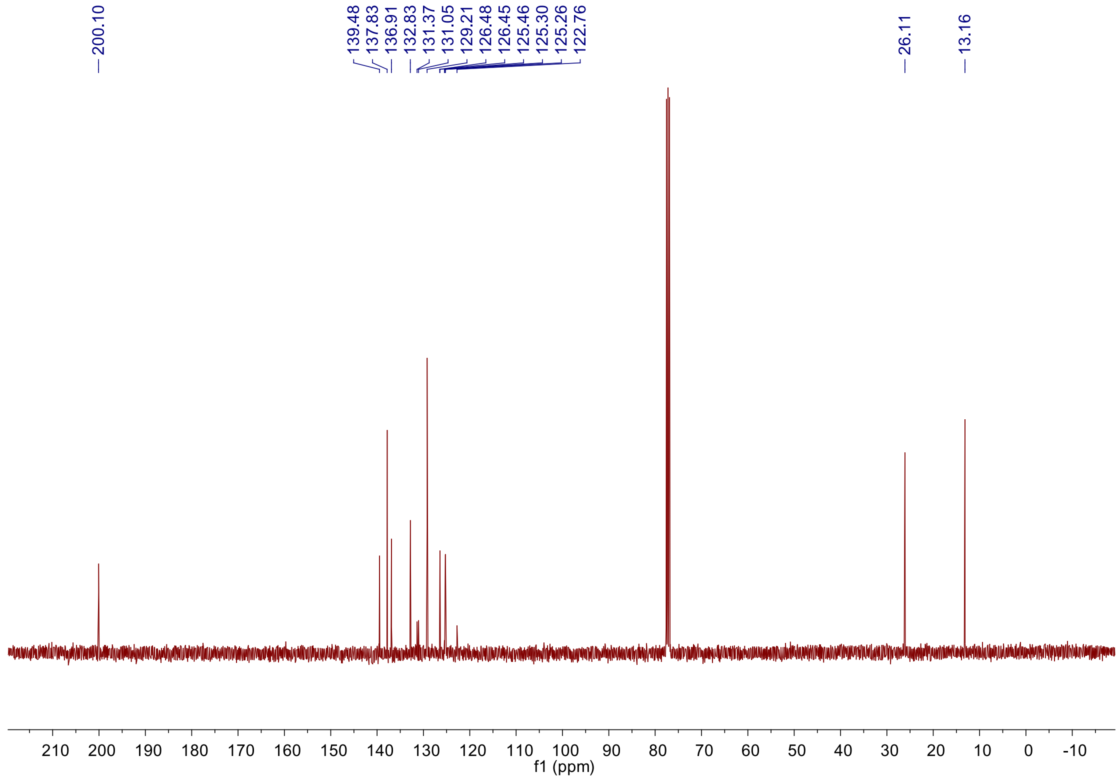

Supplementary Figure 24. ^{13}C NMR (100 MHz, $CDCl_3$) spectrum of (*E*)-**1ae**.

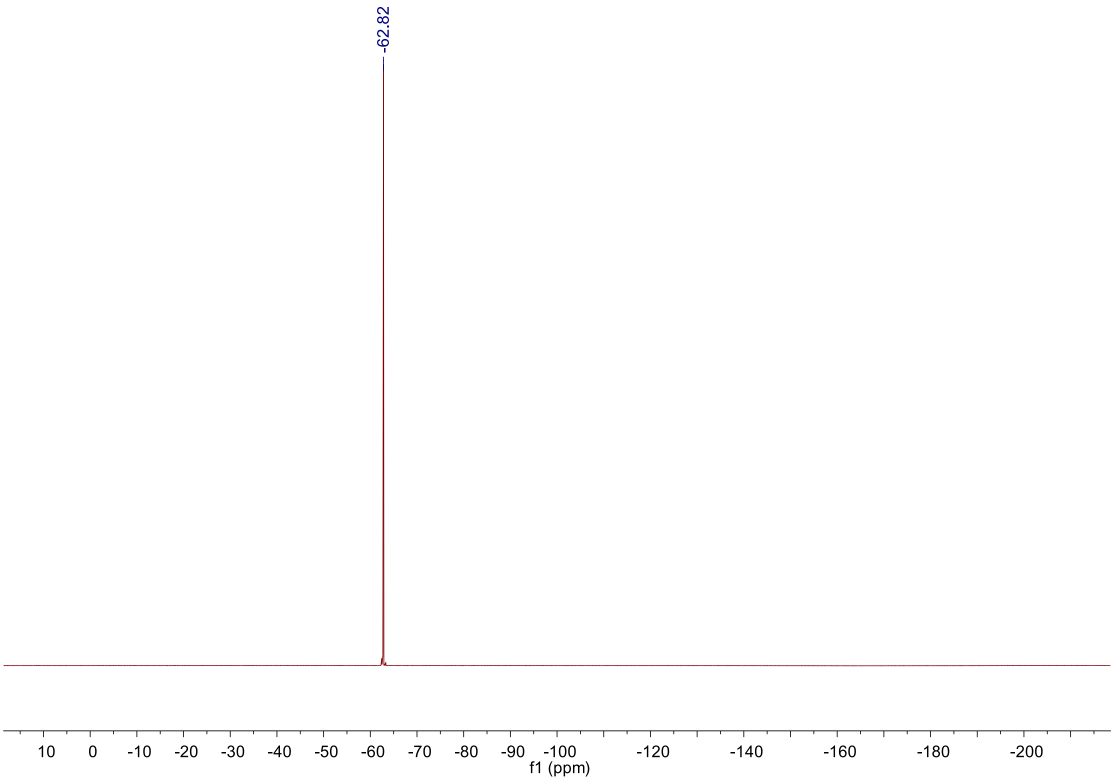

Supplementary Figure 25. ^{19}F NMR (377 MHz, $CDCl_3$) spectrum of (*E*)-**1ae**.

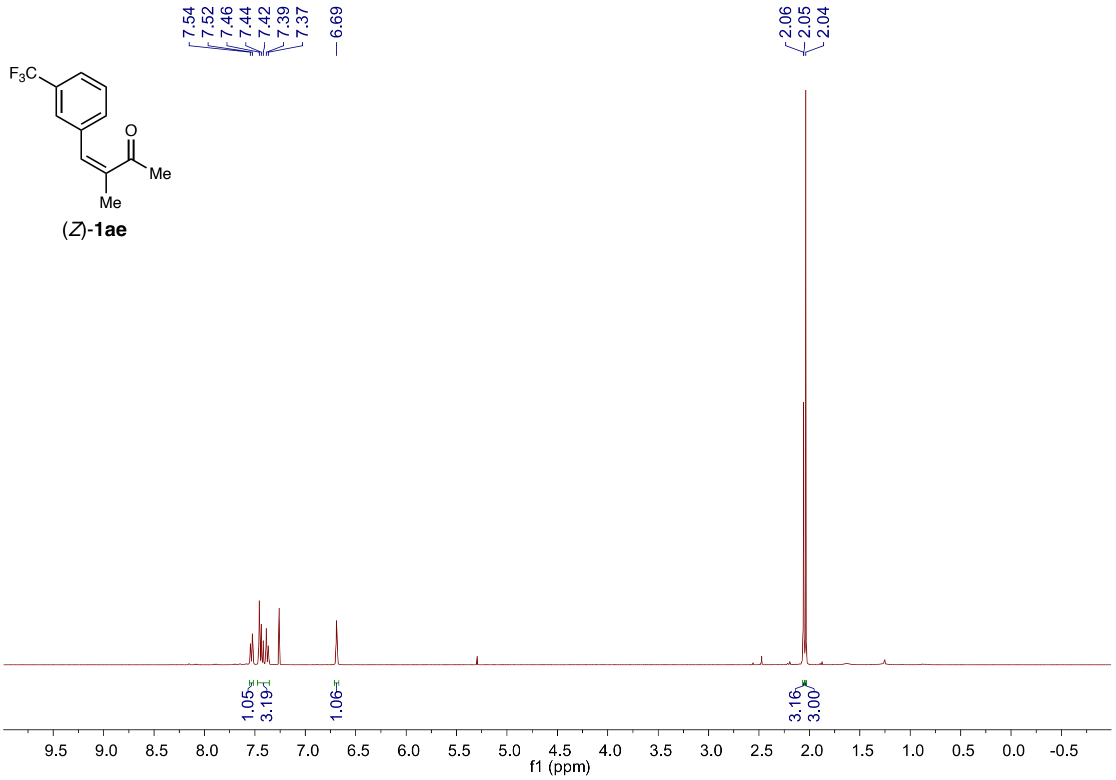

Supplementary Figure 26. ^{1}H NMR (400 MHz, $CDCl_3$) spectrum of (*Z*)-**1ae**.

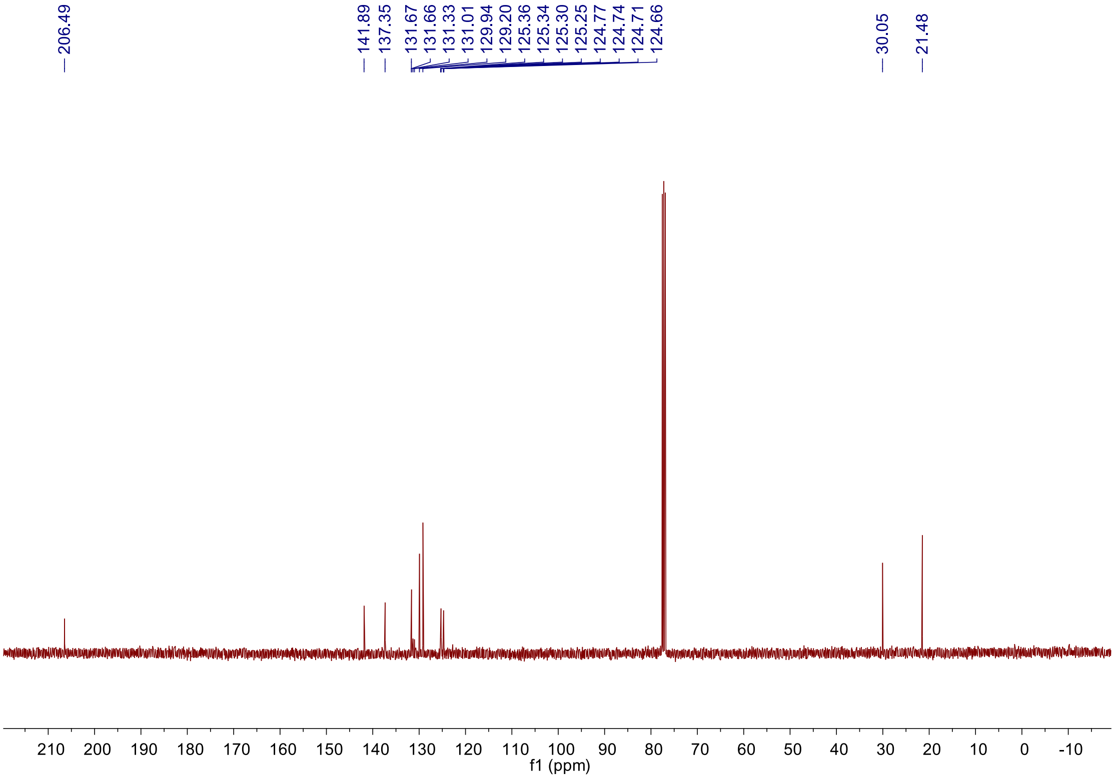

Supplementary Figure 27. ^{13}C NMR (100 MHz, $CDCl_3$) spectrum of (*Z*)-**1ae**.

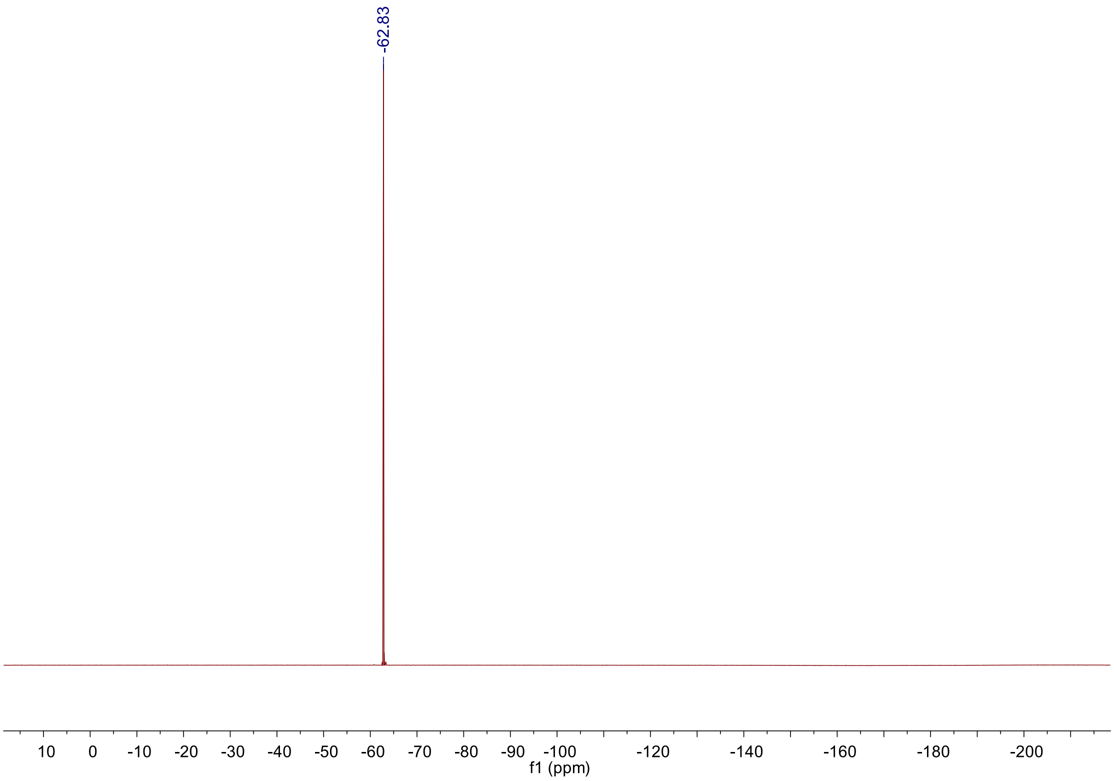

Supplementary Figure 28. ^{19}F NMR (377 MHz, $CDCl_3$) spectrum of (*Z*)-**1ae**.

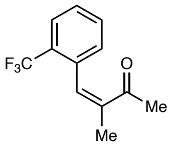

(*Z*)-**1af**

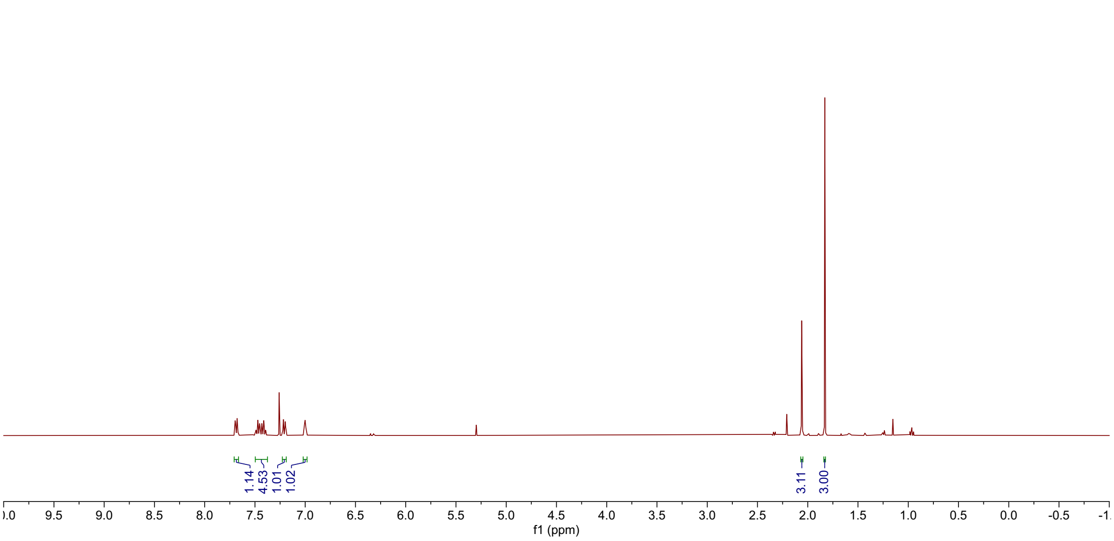

Supplementary Figure 29. ^{1}H NMR (400 MHz, $CDCl_3$) spectrum of (*Z*)-**1af**.

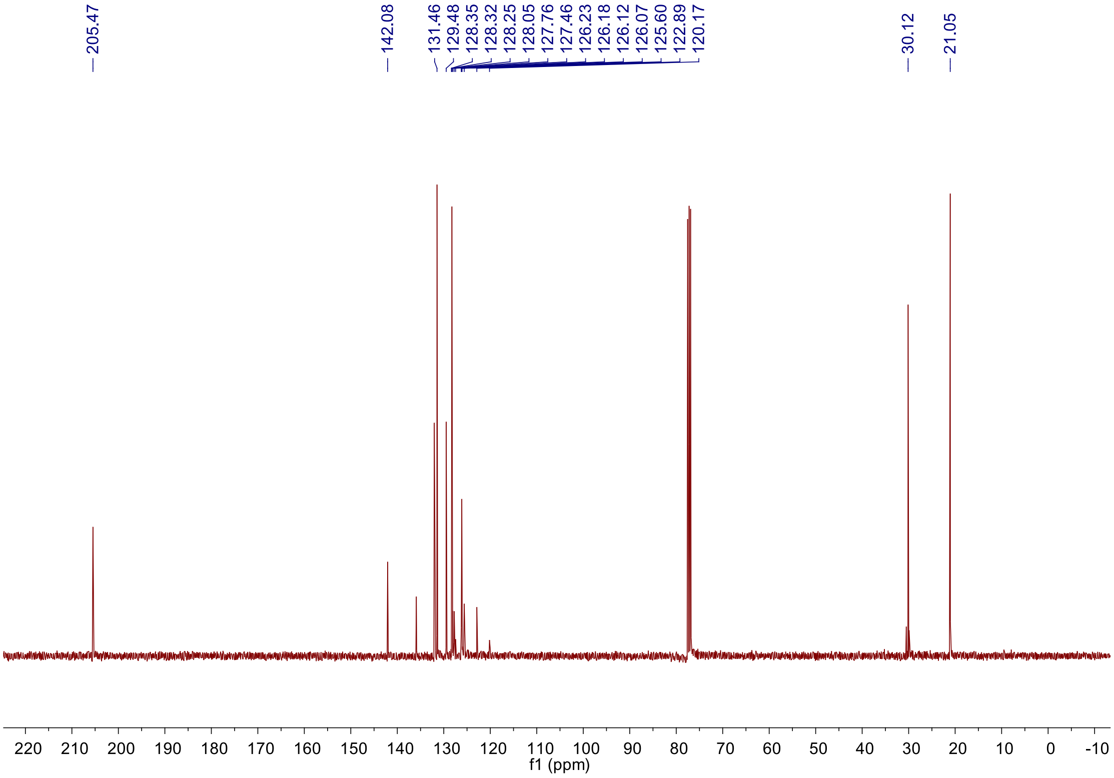

Supplementary Figure 30. ^{13}C NMR (100 MHz, $CDCl_3$) spectrum of (*Z*)-**1af**.

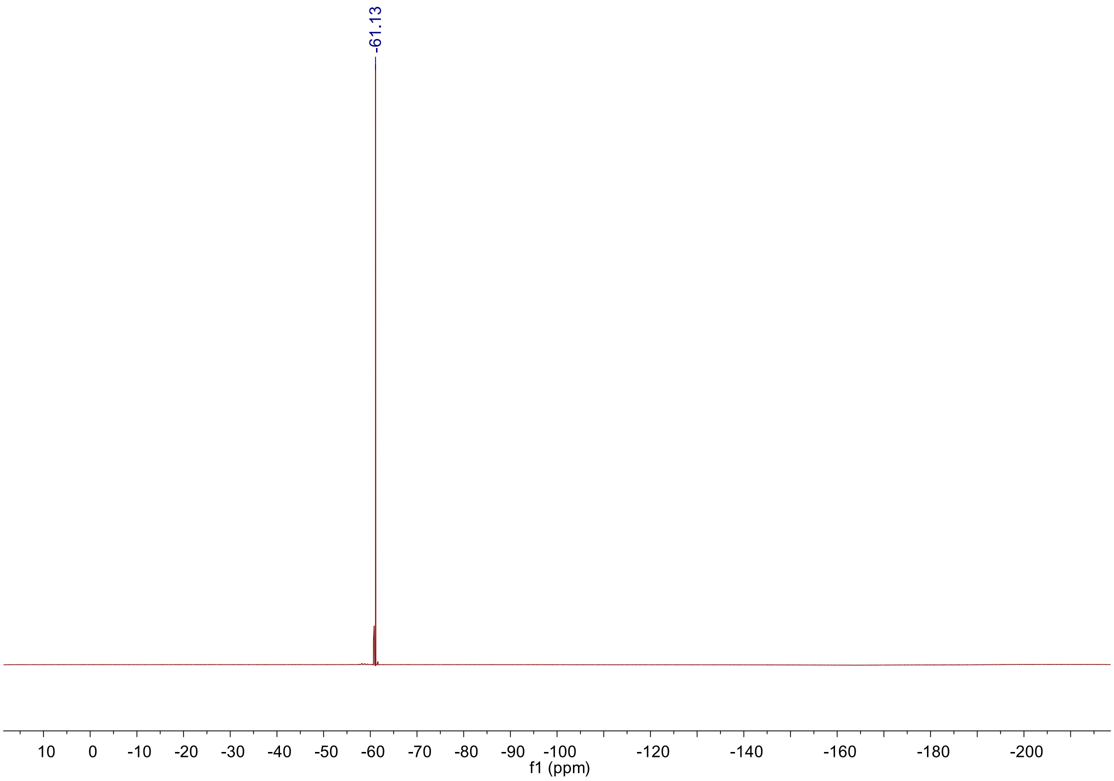

Supplementary Figure 31. ^{19}F NMR (377 MHz, $CDCl_3$) spectrum of (*Z*)-**1af**.

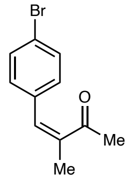

(*Z*)-**1ag**

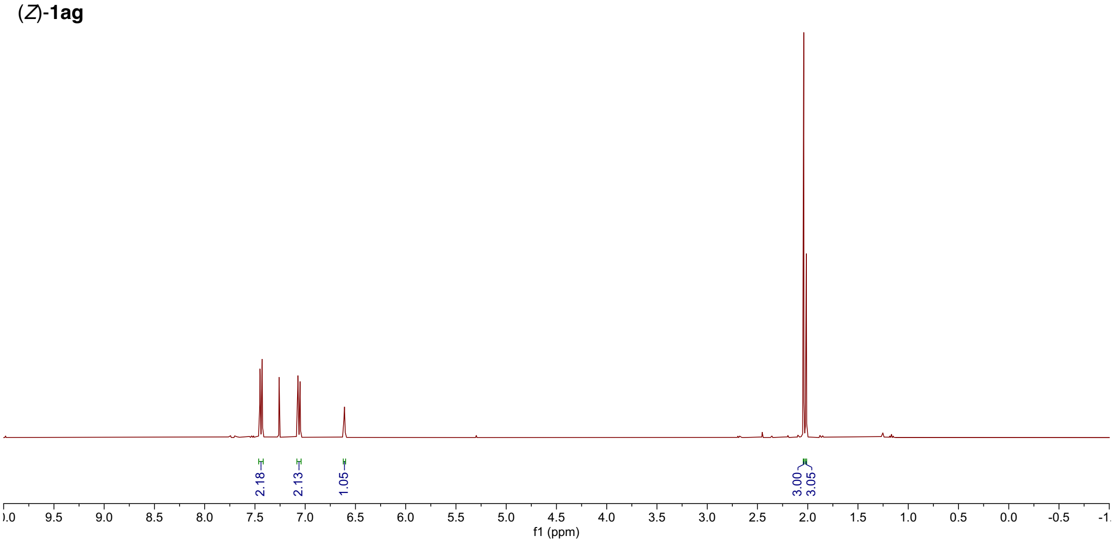

Supplementary Figure 32. ^{1}H NMR (400 MHz, $CDCl_3$) spectrum of (*Z*)-**1ag**.

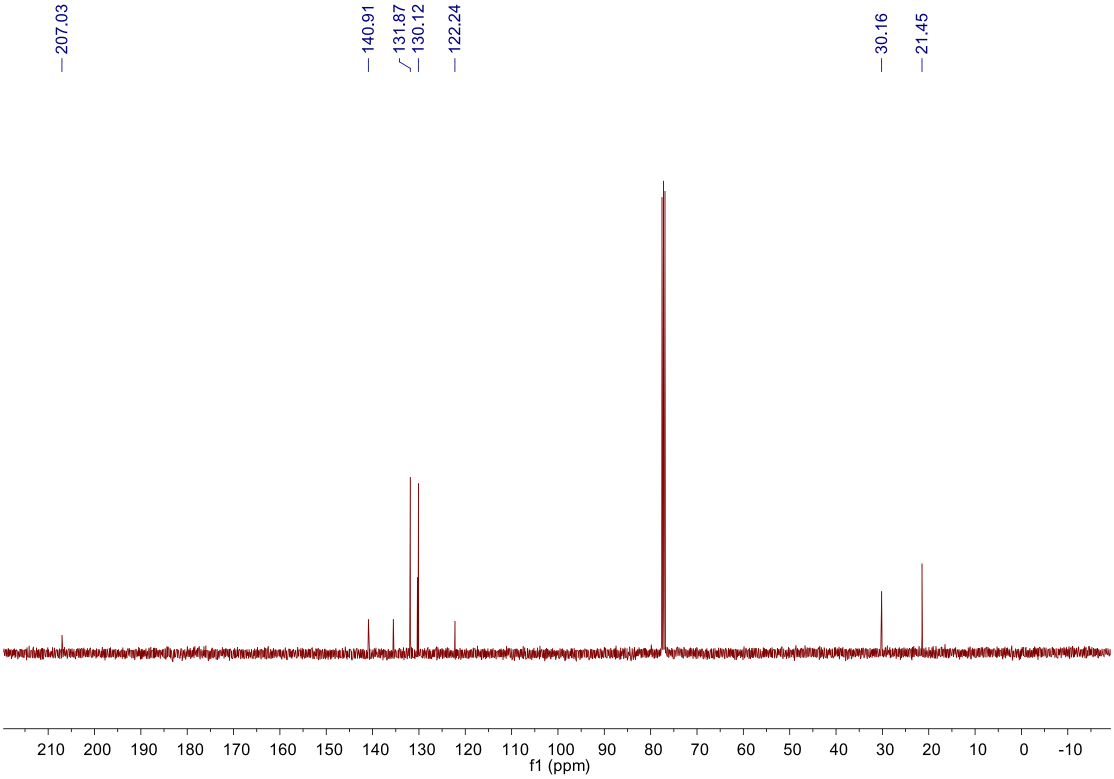

Supplementary Figure 33. ^{13}C NMR (100 MHz, $CDCl_3$) spectrum of (*Z*)-**1ag**.

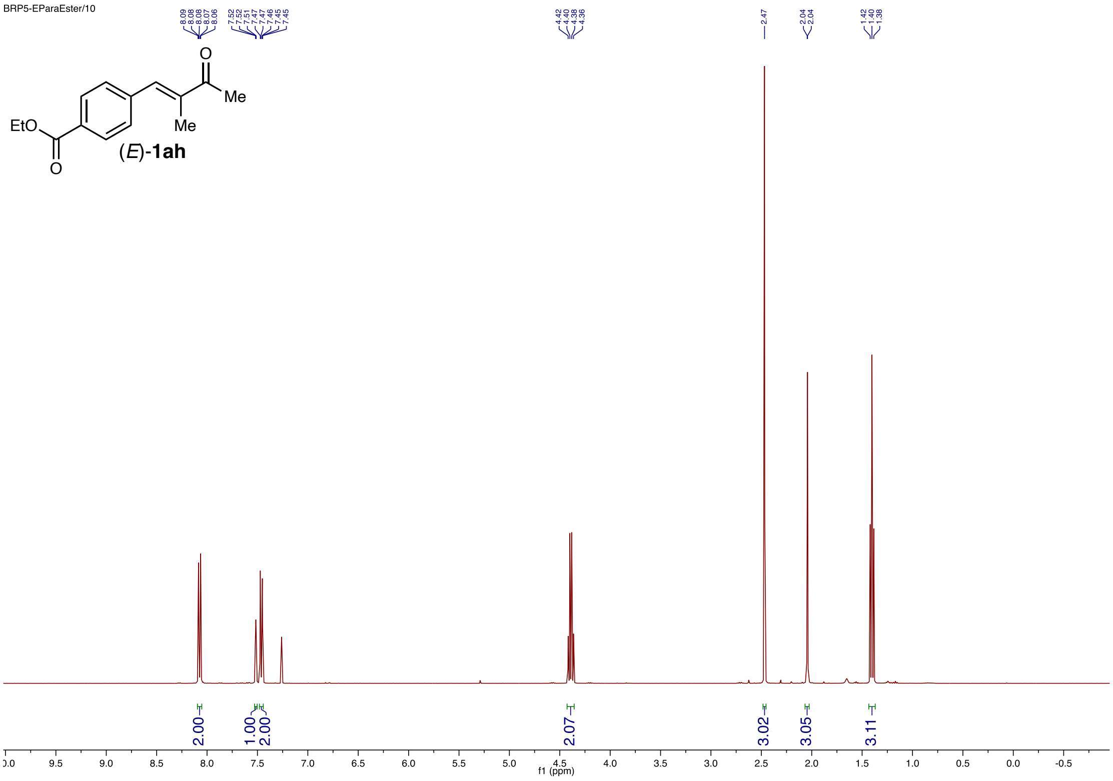

Supplementary Figure 34. ^{1}H NMR (400 MHz, $CDCl_3$) spectrum of (*E*)-**1ah**.

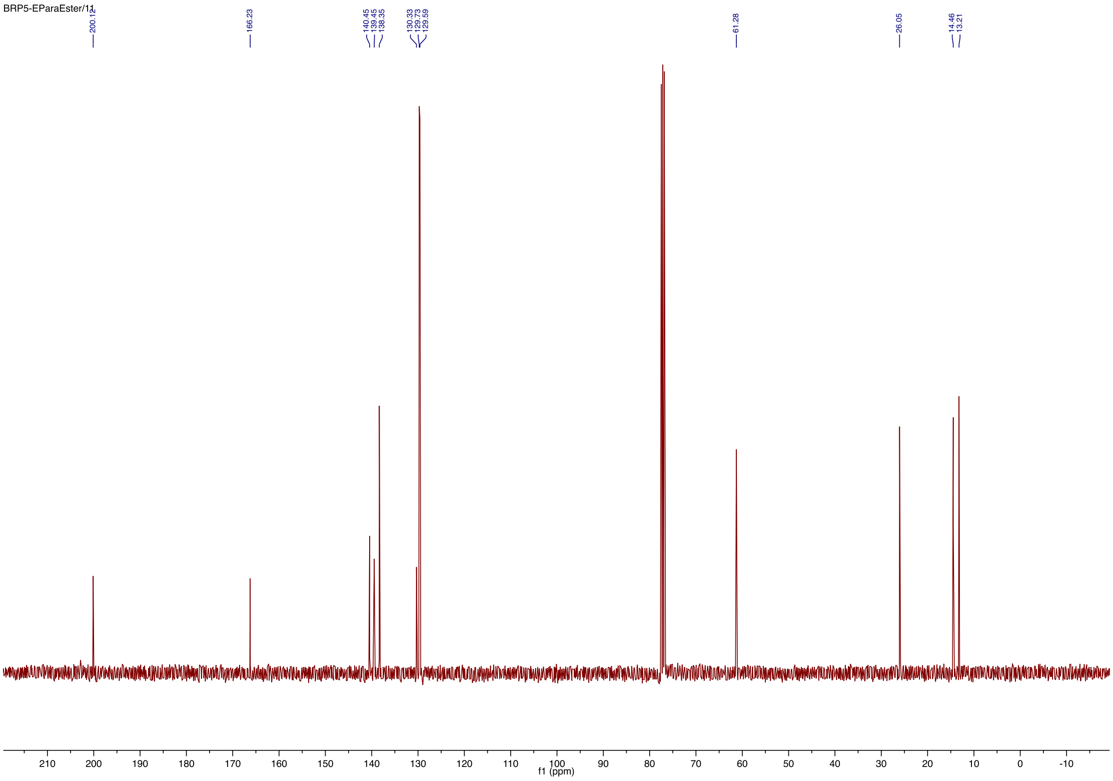

Supplementary Figure 35. ^{13}C NMR (100 MHz, $CDCl_3$) spectrum of (*E*)-**1ah**.

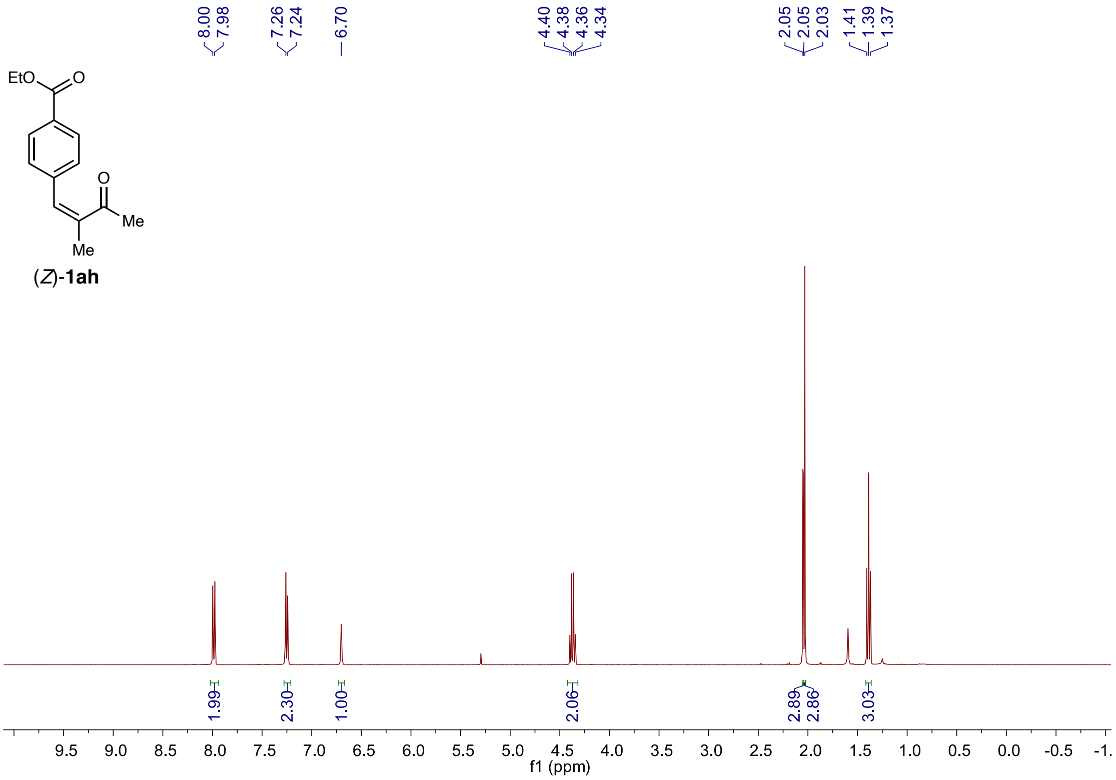

Supplementary Figure 36. ^{1}H NMR (400 MHz, $CDCl_3$) spectrum of (*Z*)-**1ah**.

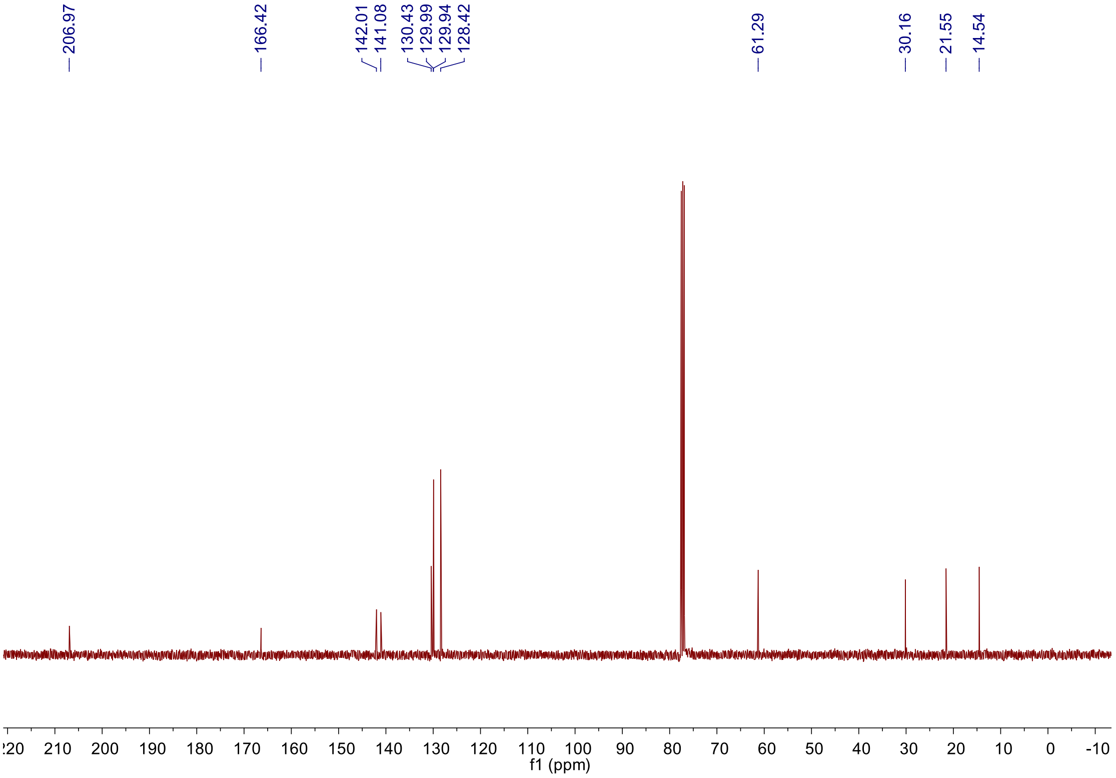

Supplementary Figure 37. ^{13}C NMR (100 MHz, $CDCl_3$) spectrum of (*Z*)-**1ah**.

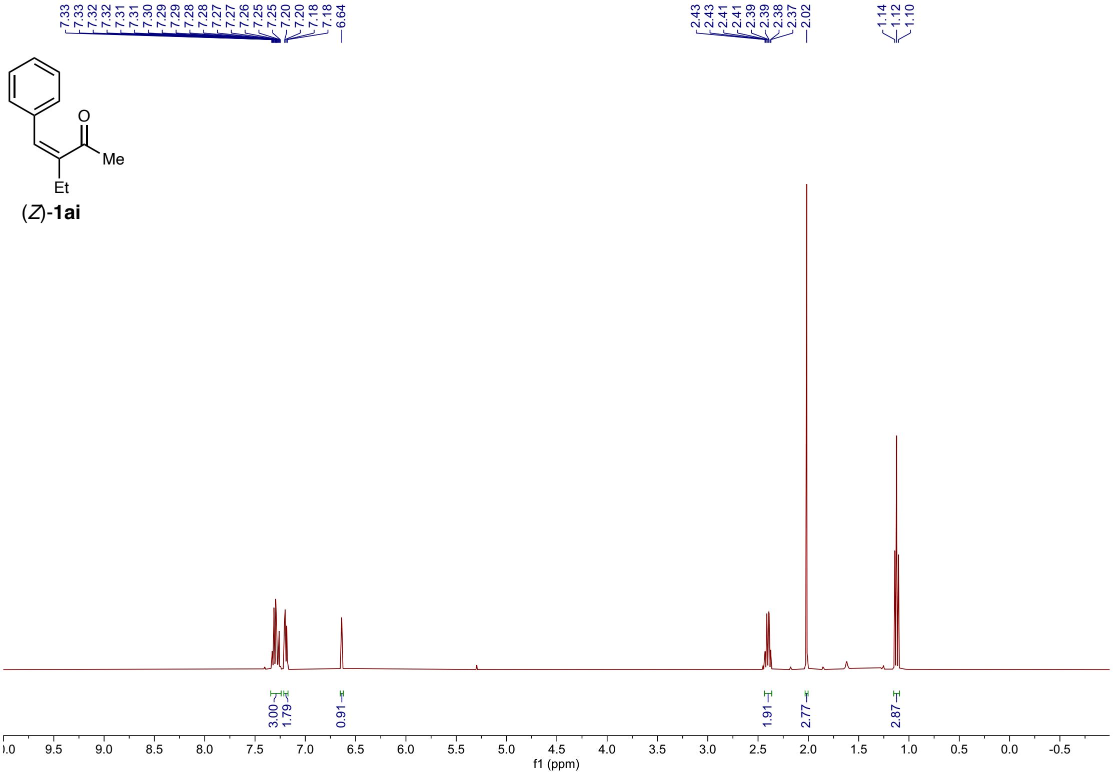

Supplementary Figure 38. ^{1}H NMR (400 MHz, $CDCl_3$) spectrum of (*Z*)-**1ai**.

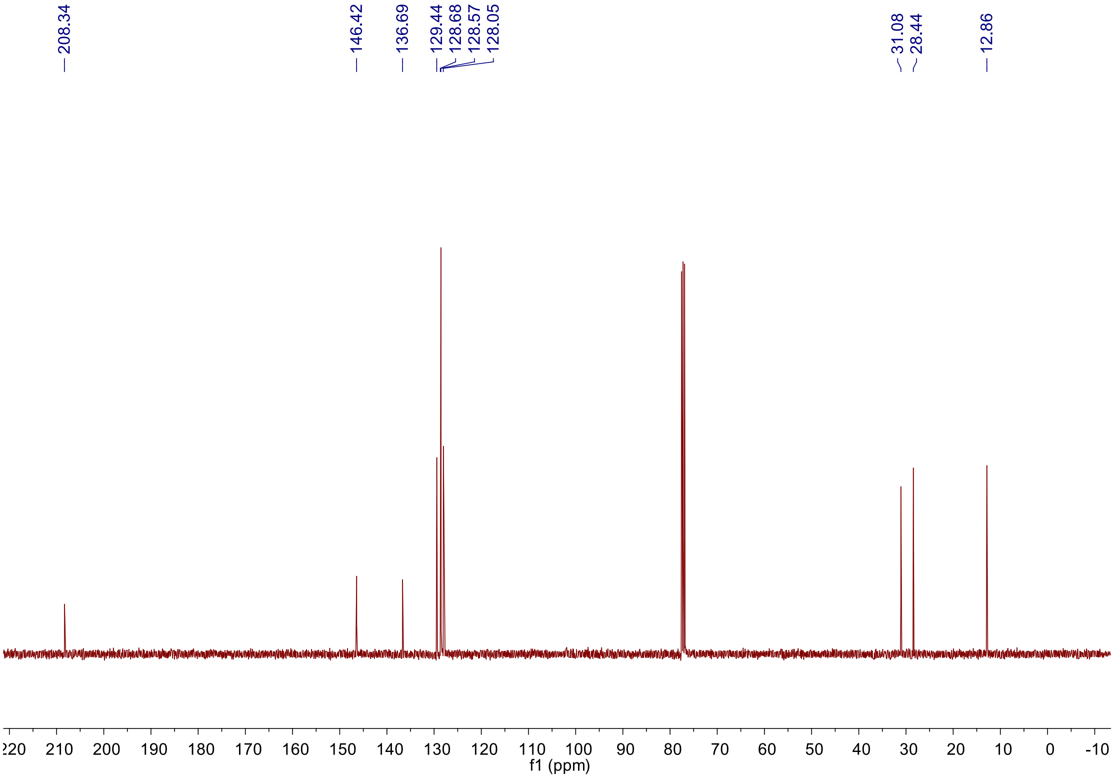

Supplementary Figure 39. ^{13}C NMR (100 MHz, $CDCl_3$) spectrum of (*Z*)-**1ai**.

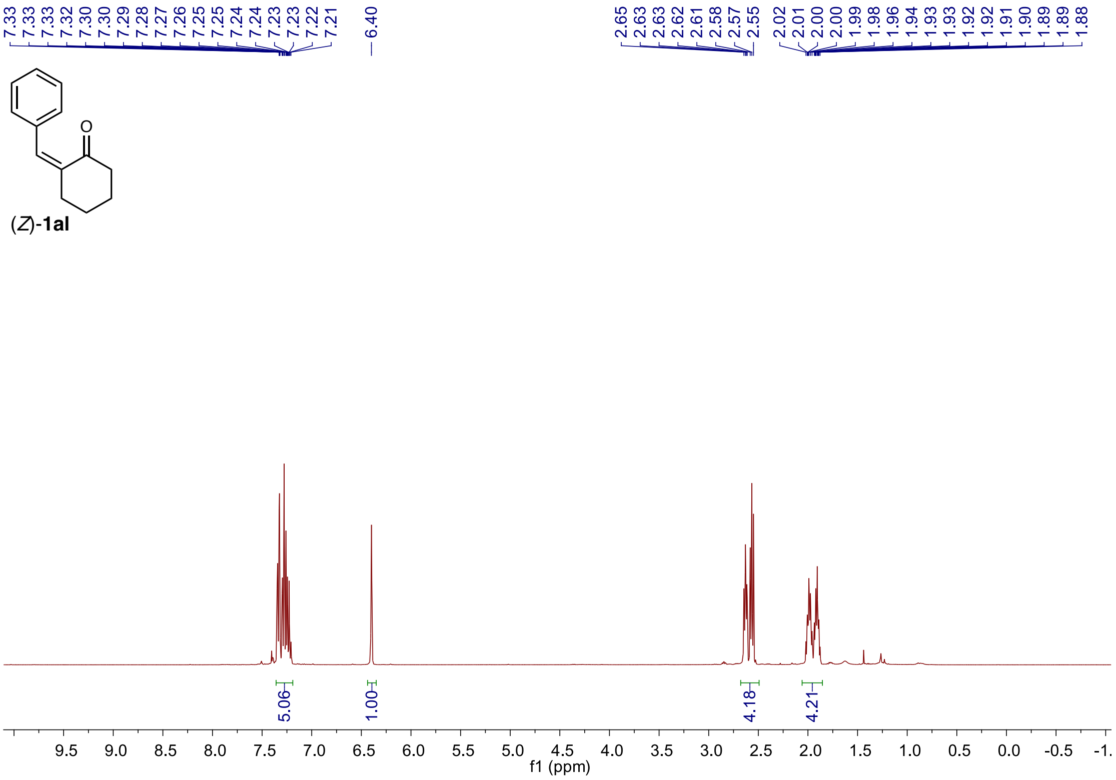

Supplementary Figure 40. ^{1}H NMR (400 MHz, $CDCl_3$) spectrum of (*Z*)-**1al**.

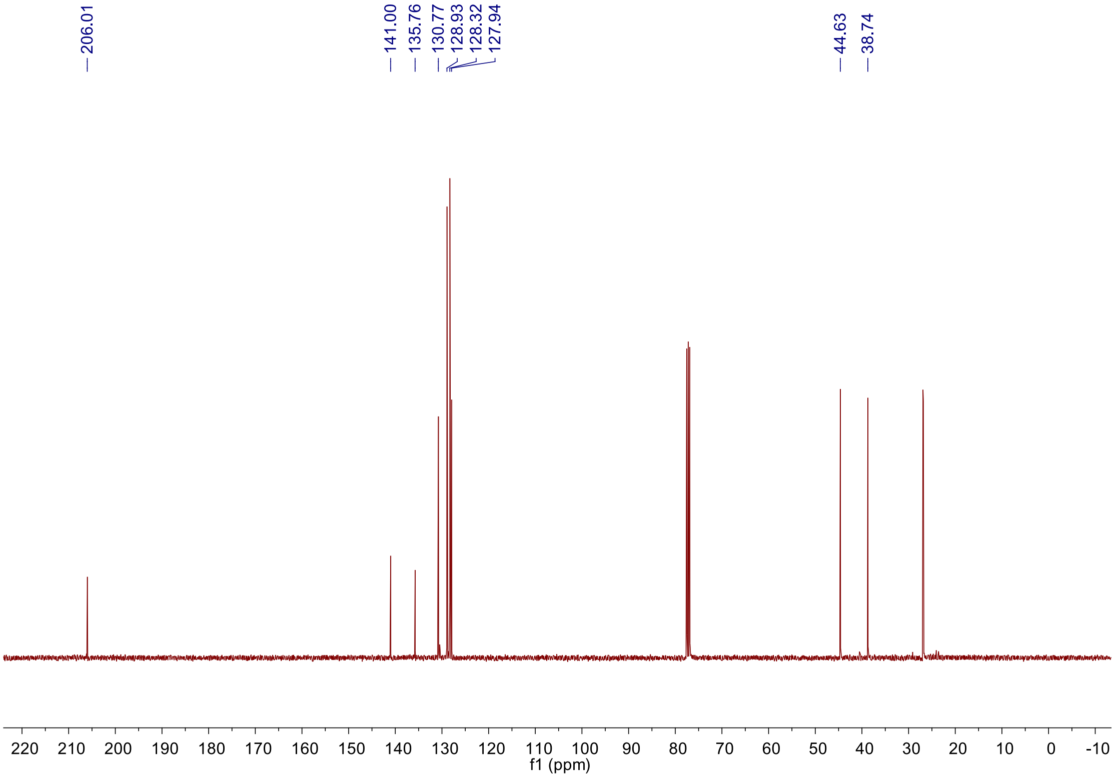

Supplementary Figure 41. ^{13}C NMR (100 MHz, $CDCl_3$) spectrum of (*Z*)-**1al**.

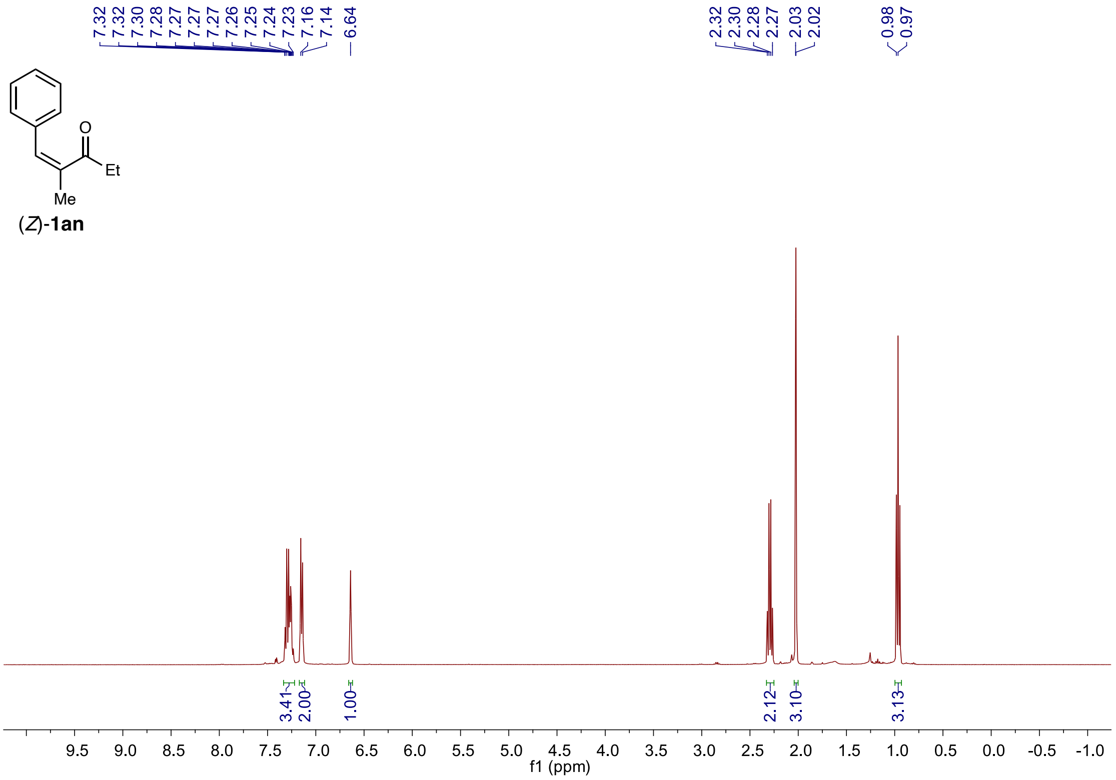

Supplementary Figure 42. ^{1}H NMR (400 MHz, $CDCl_3$) spectrum of (*Z*)-**1an**.

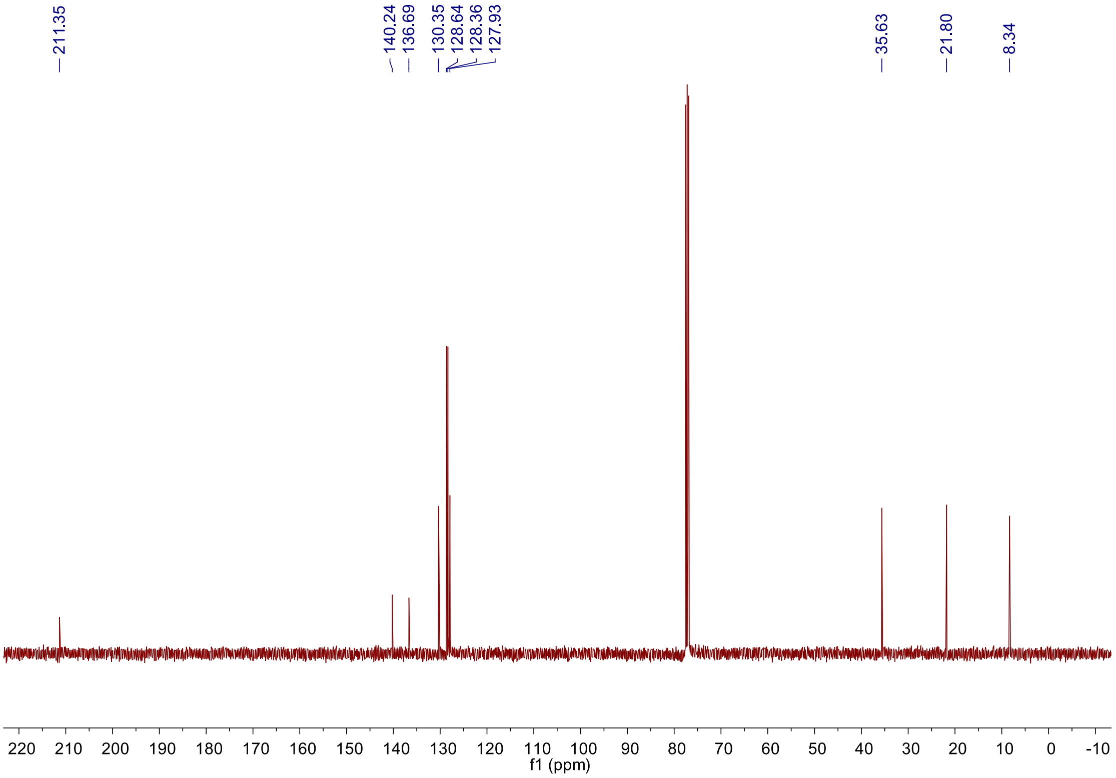

Supplementary Figure 43. ^{13}C NMR (100 MHz, $CDCl_3$) spectrum of (*Z*)-**1an**.

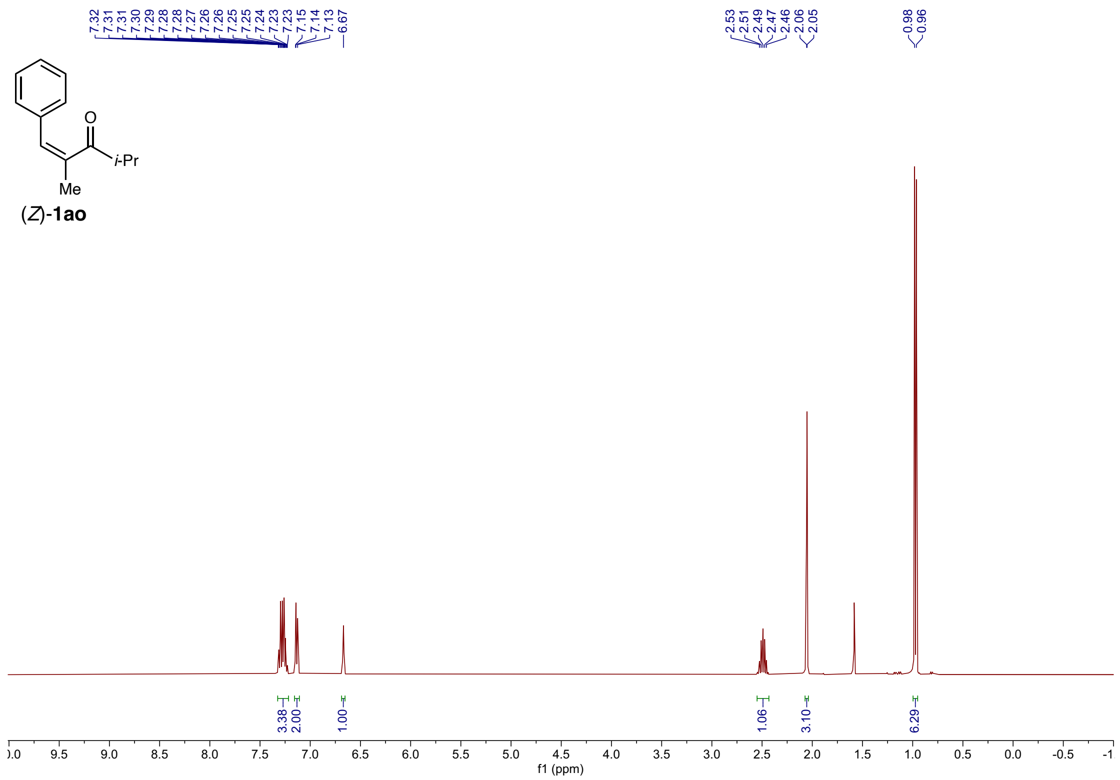

Supplementary Figure 44. ^{1}H NMR (400 MHz, $CDCl_3$) spectrum of (*Z*)-**1ao**.

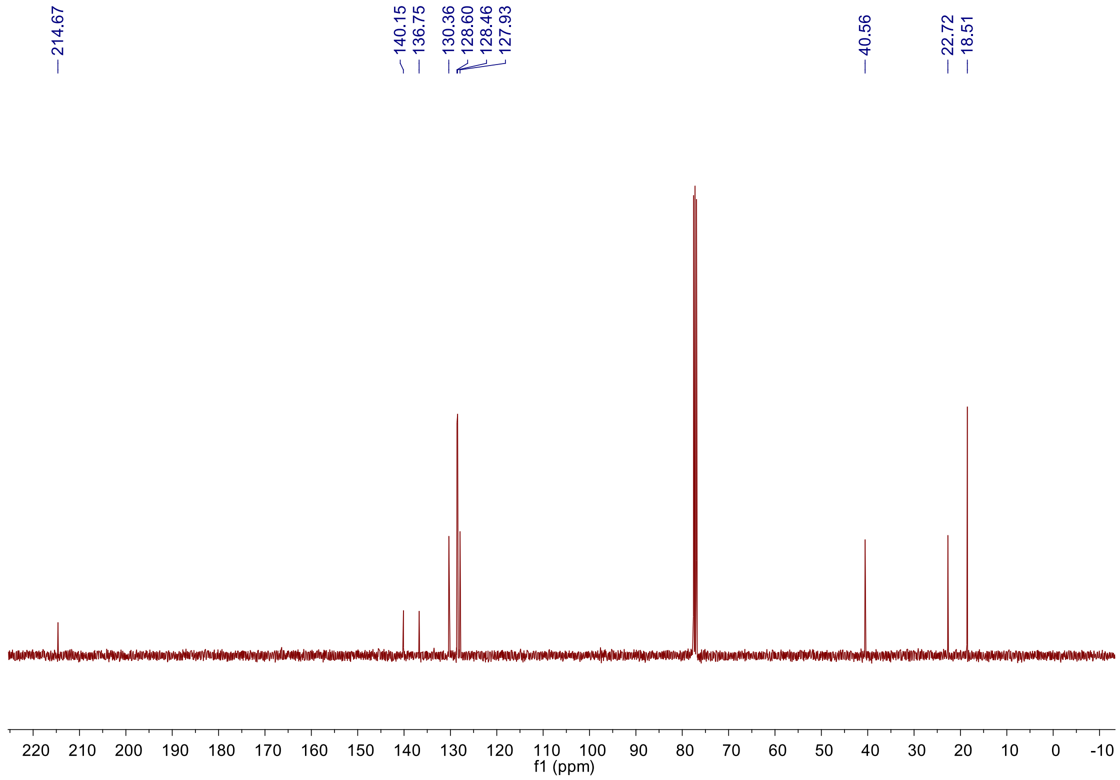

Supplementary Figure 45. ^{13}C NMR (100 MHz, $CDCl_3$) spectrum of (*Z*)-**1ao**.

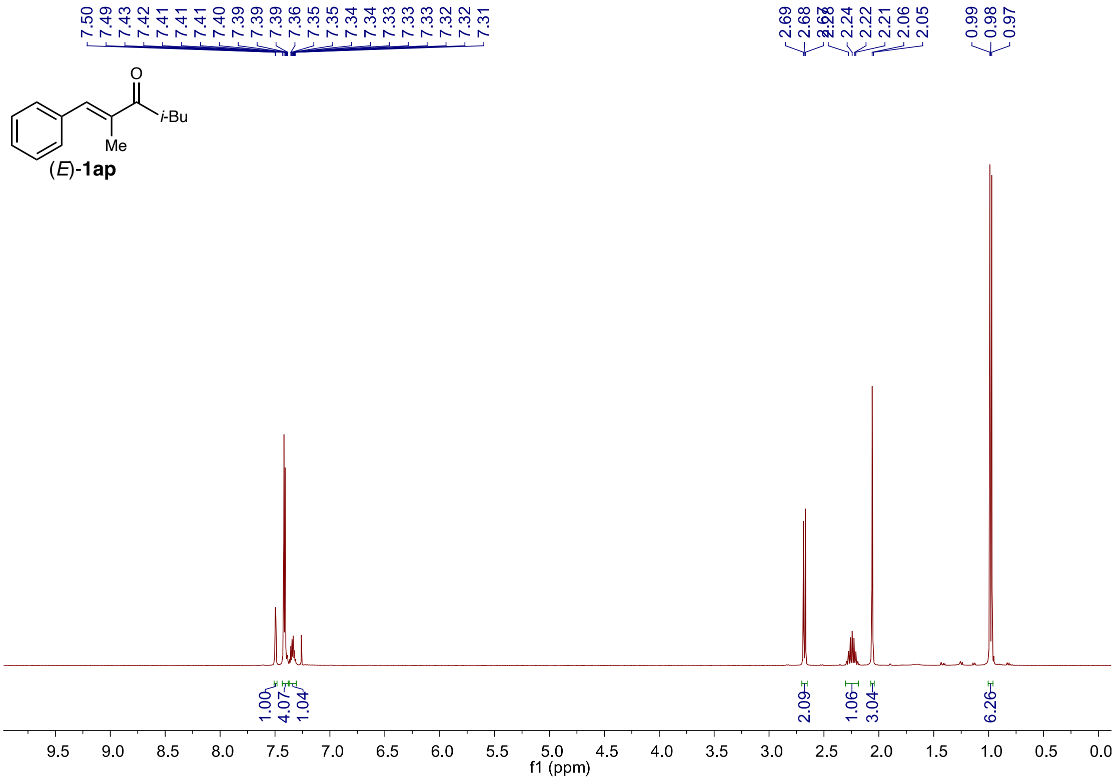

Supplementary Figure 46. ^{1}H NMR (400 MHz, $CDCl_3$) spectrum of (*E*)-**1ap**.

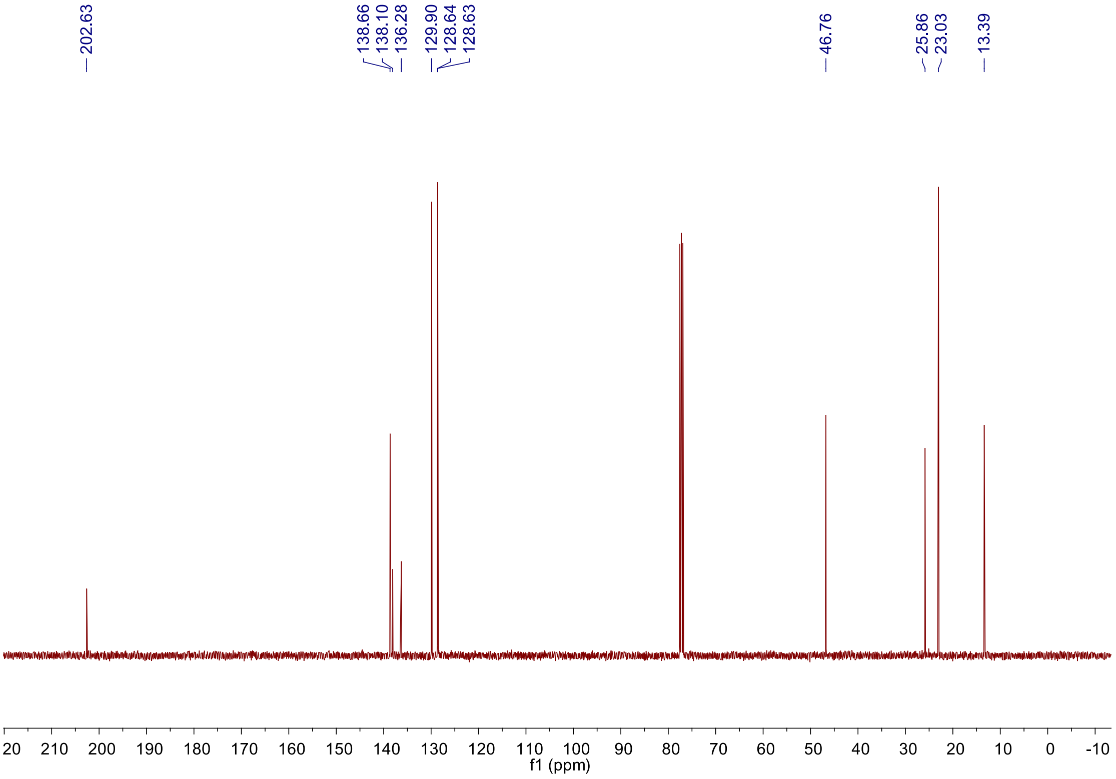

Supplementary Figure 47. ^{13}C NMR (100 MHz, $CDCl_3$) spectrum of (*E*)-**1ap**.

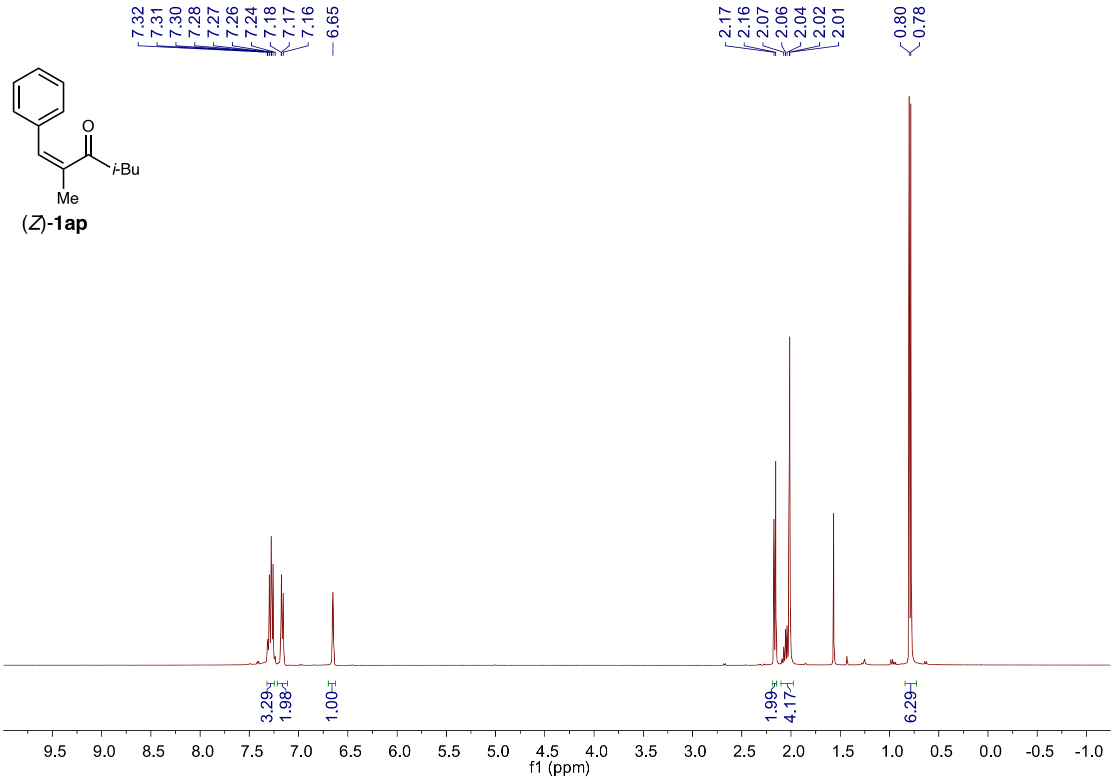

Supplementary Figure 48. ^{1}H NMR (400 MHz, $CDCl_3$) spectrum of (*Z*)-**1ap**.

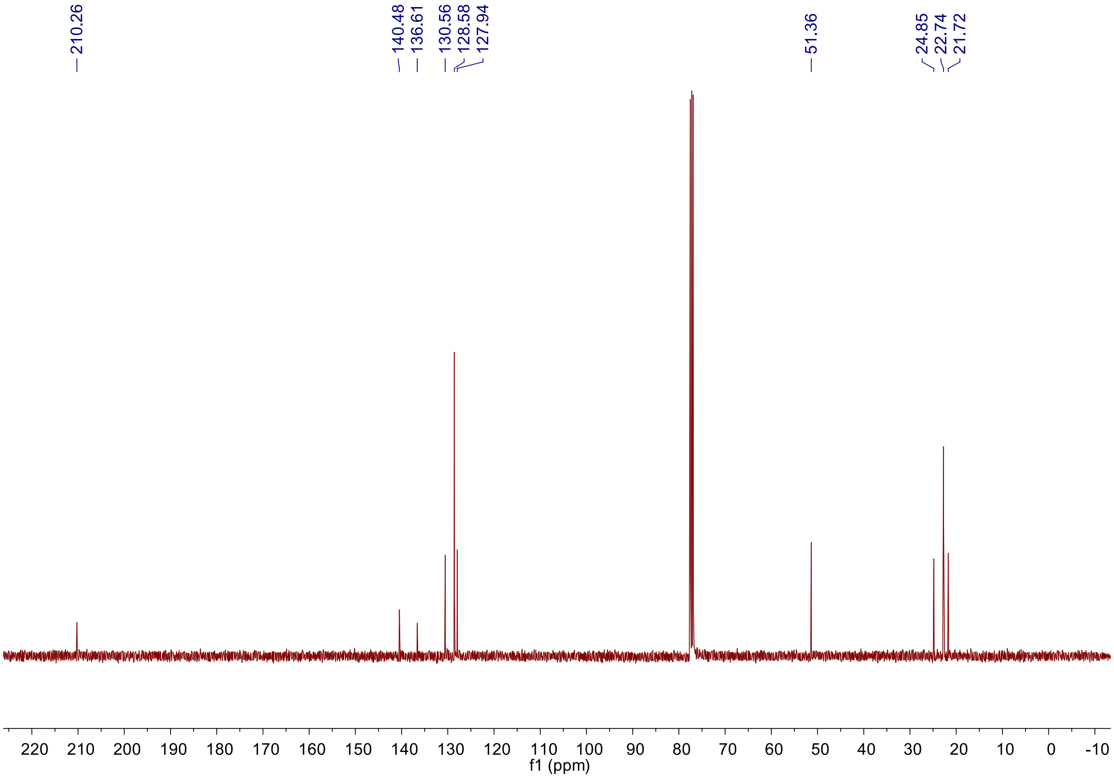

Supplementary Figure 49. ^{13}C NMR (100 MHz, $CDCl_3$) spectrum of (*Z*)-**1ap**.

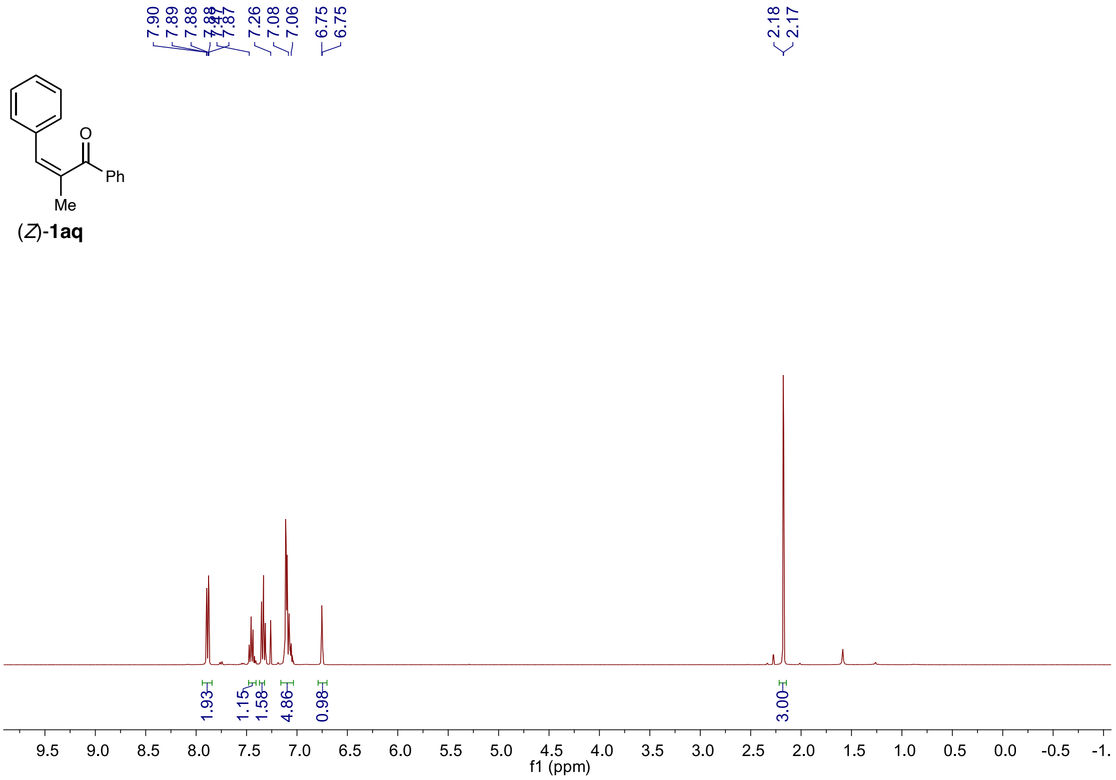

Supplementary Figure 50. ^{1}H NMR (400 MHz, $CDCl_3$) spectrum of (*Z*)-**1aq**.

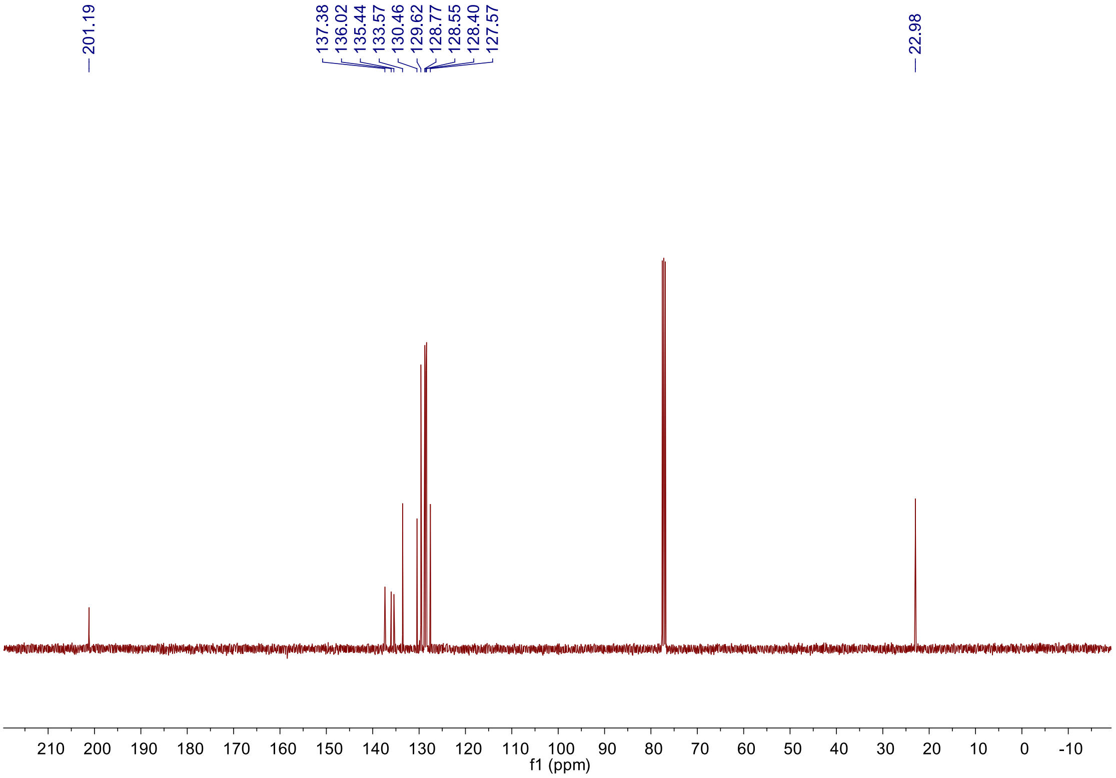

Supplementary Figure 51. ^{13}C NMR (100 MHz, $CDCl_3$) spectrum of (*Z*)-**1aq**.

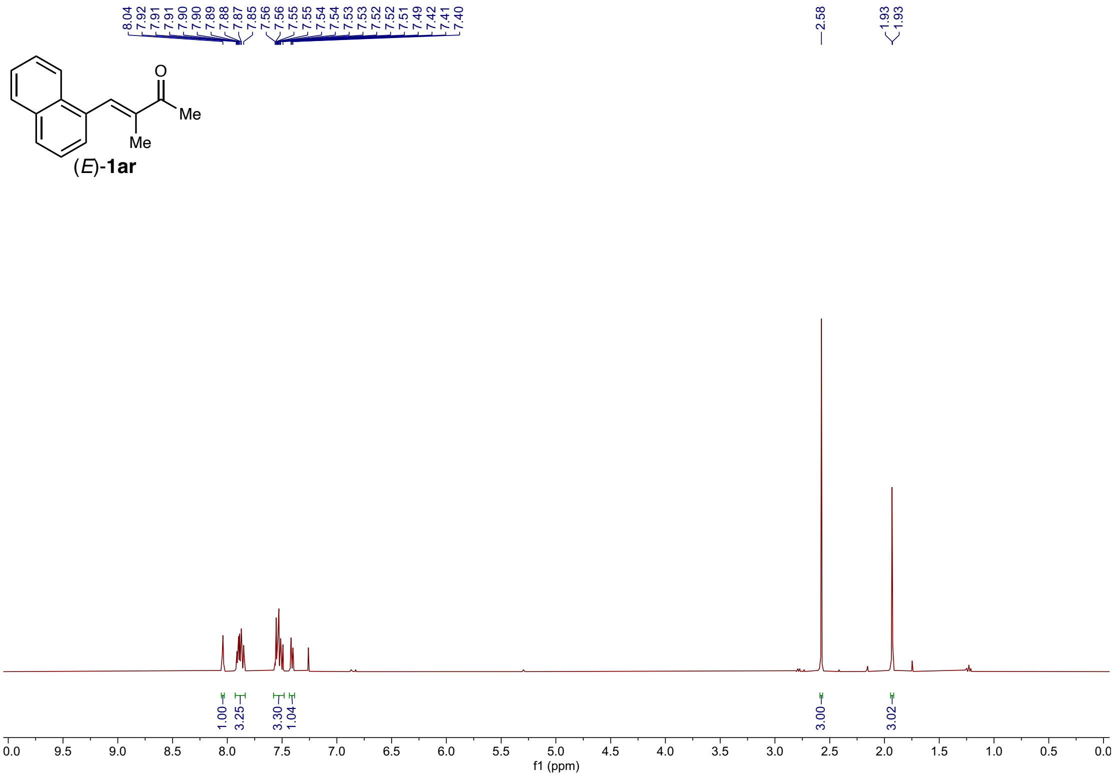

Supplementary Figure 52. ^{1}H NMR (400 MHz, $CDCl_3$) spectrum of (*E*)-**1ar**.

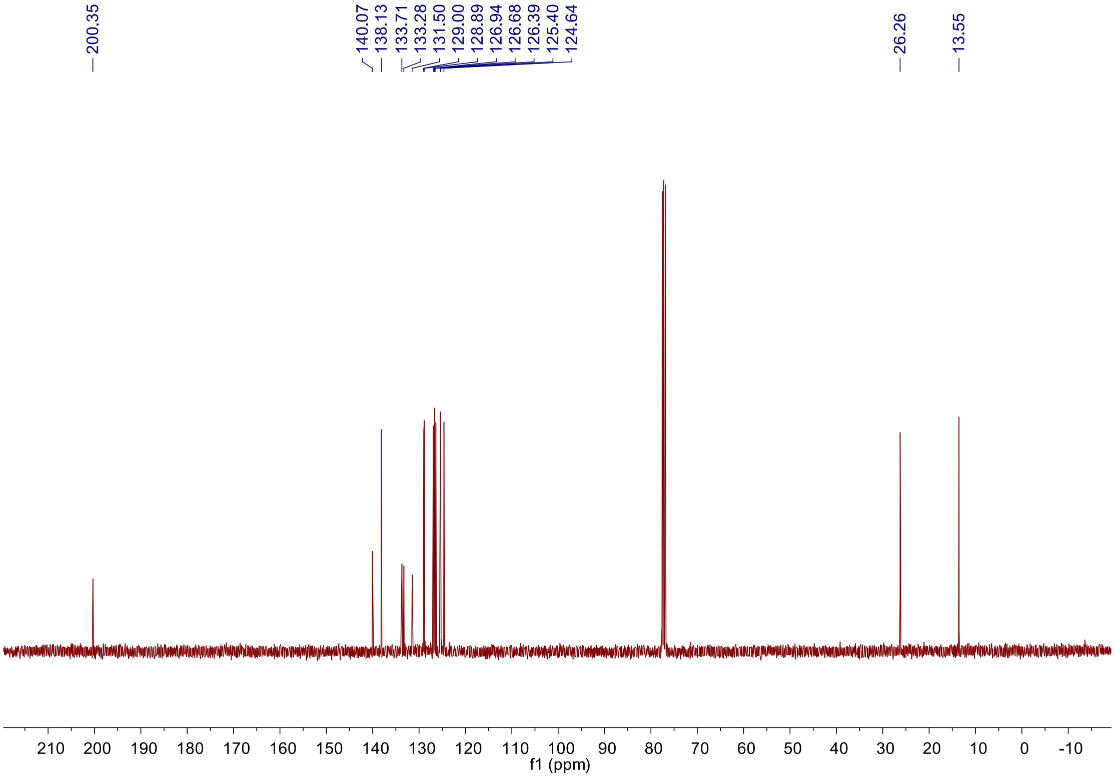

Supplementary Figure 53. ^{13}C NMR (100 MHz, $CDCl_3$) spectrum of (*E*)-**1ar**.

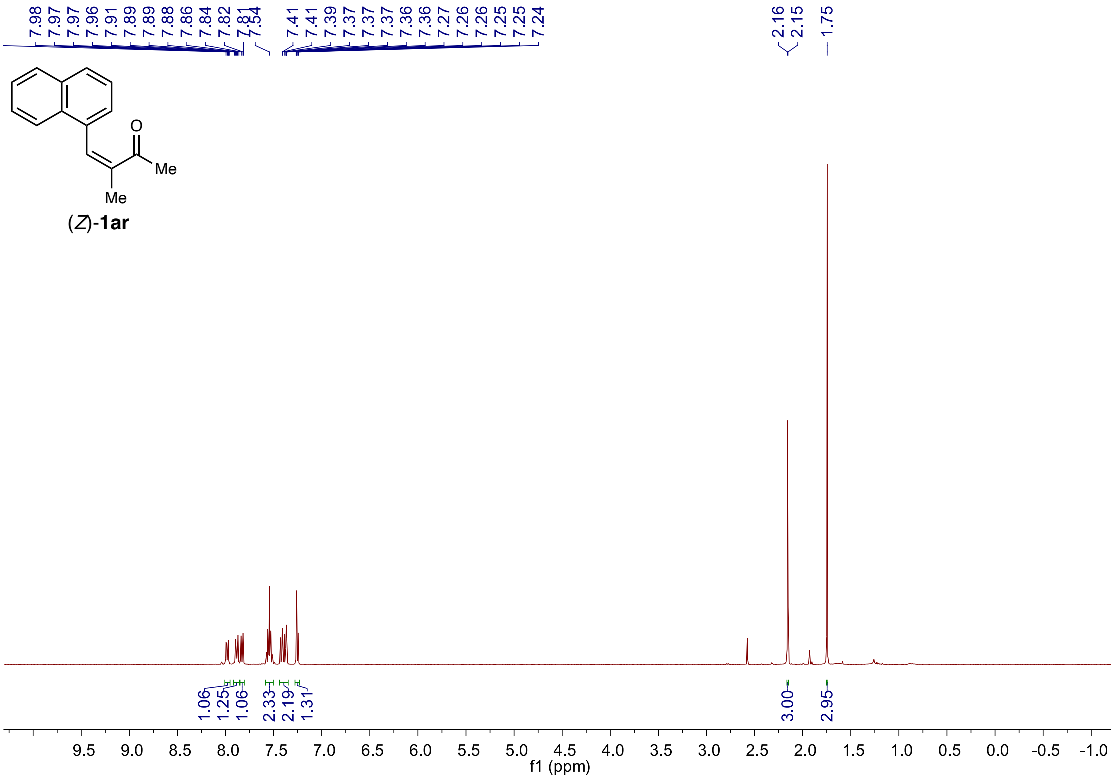

Supplementary Figure 54. ^{1}H NMR (400 MHz, $CDCl_3$) spectrum of (*Z*)-**1ar**.

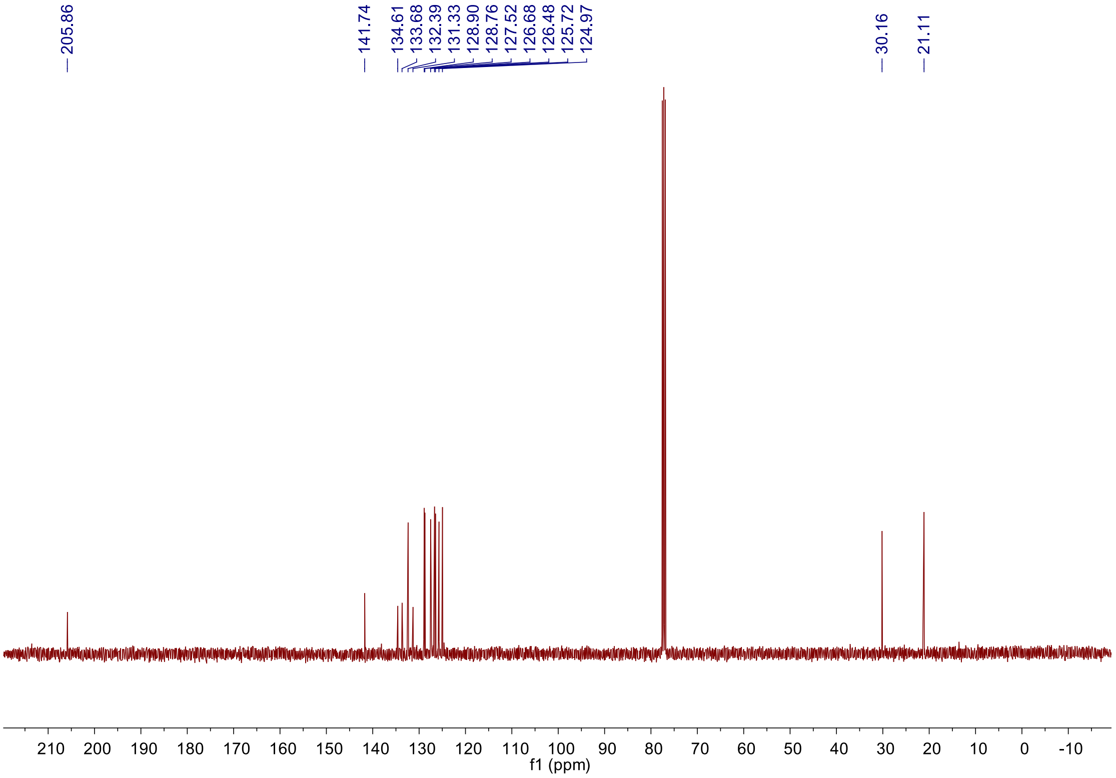

Supplementary Figure 55. ^{13}C NMR (100 MHz, $CDCl_3$) spectrum of (*Z*)-**1ar**.

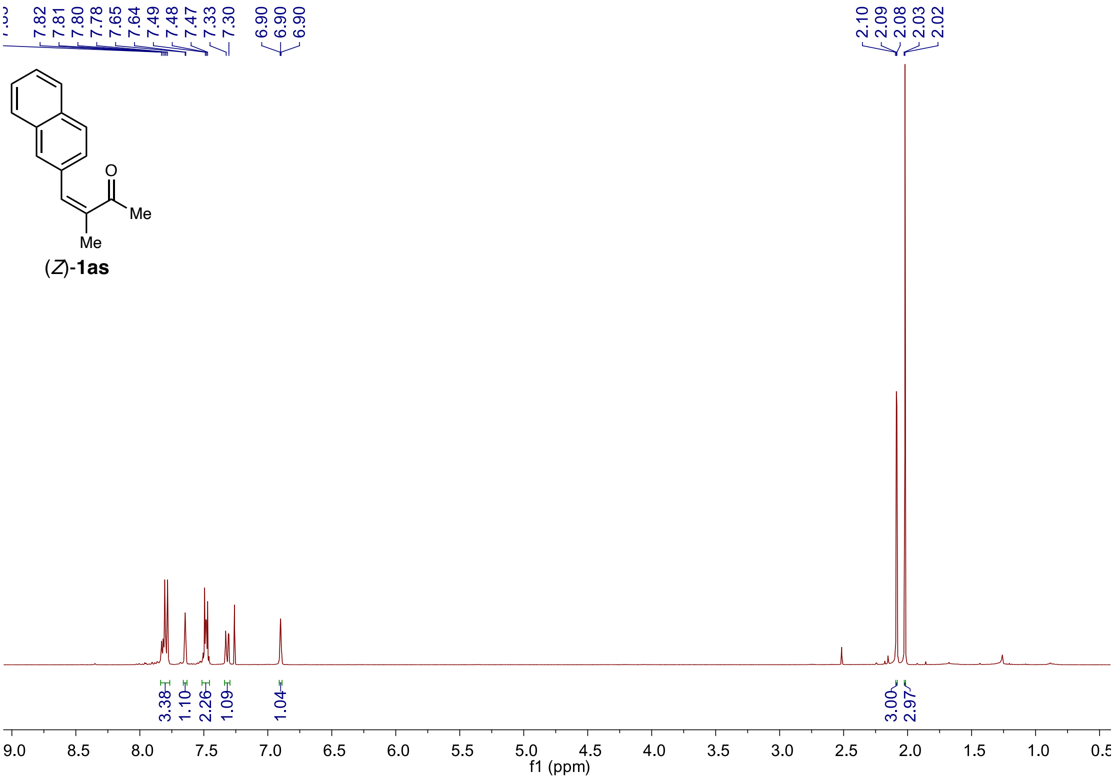

Supplementary Figure 56. ^{1}H NMR (400 MHz, $CDCl_3$) spectrum of (*Z*)-**1as**.

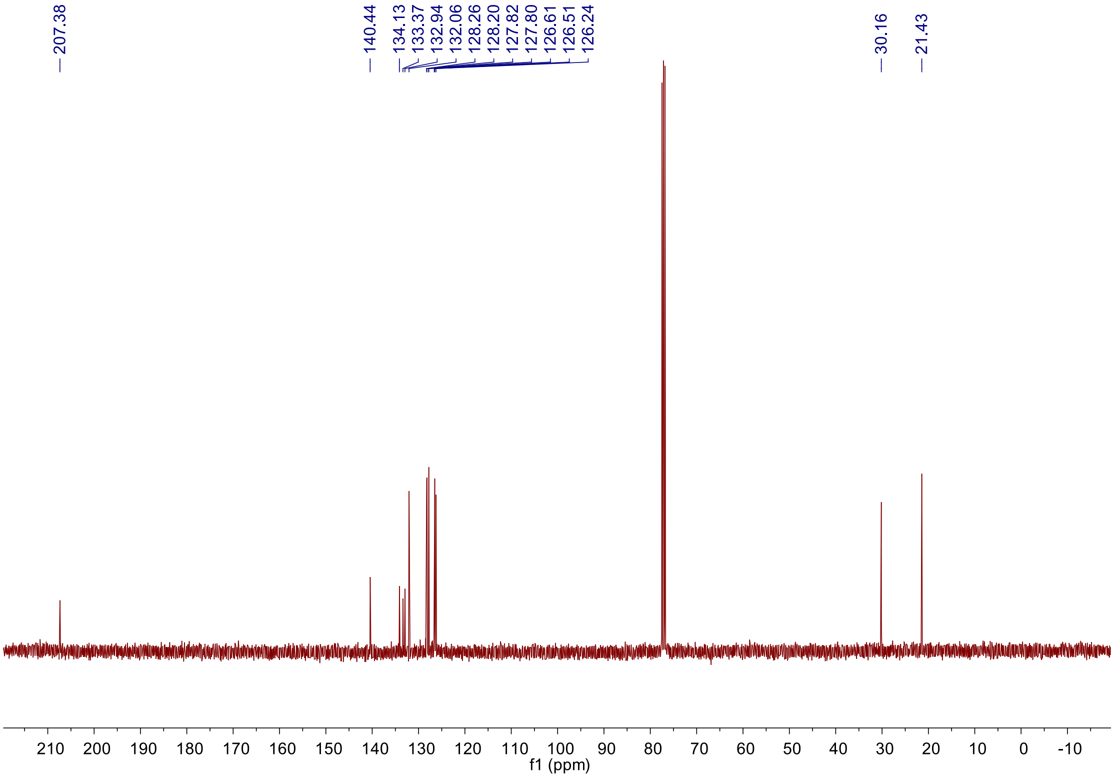

Supplementary Figure 57. ^{13}C NMR (100 MHz, $CDCl_3$) spectrum of (*Z*)-**1as**.

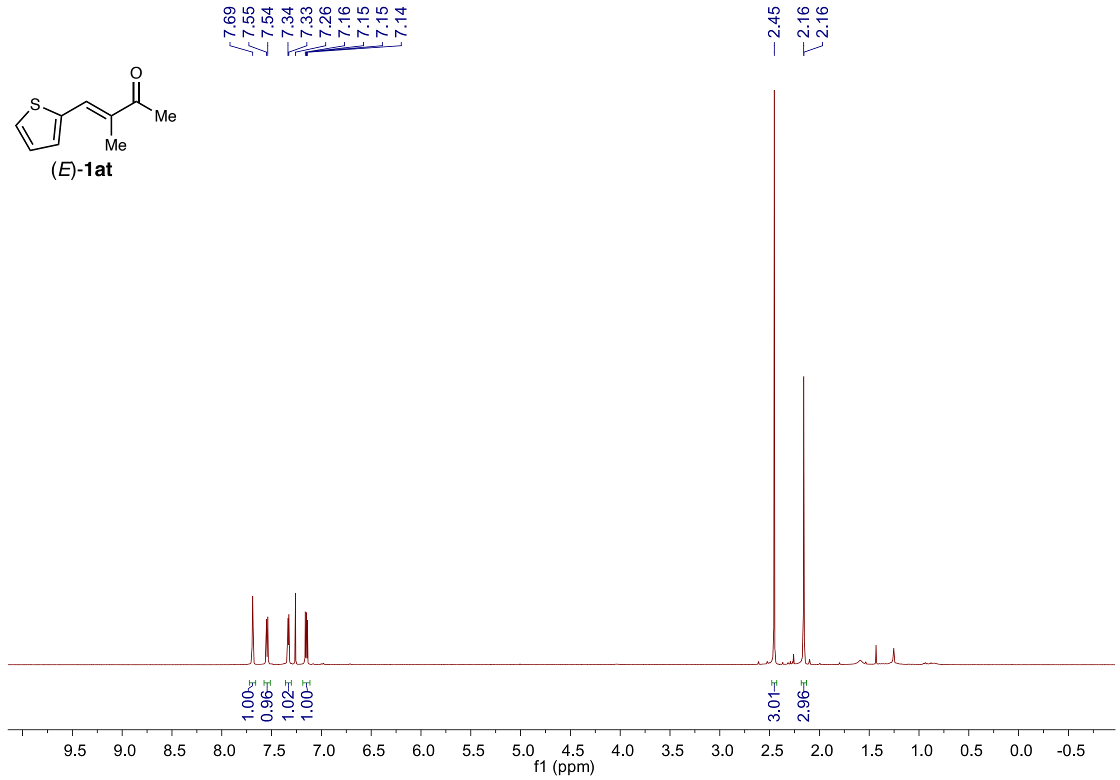

Supplementary Figure 58. ^{1}H NMR (400 MHz, $CDCl_3$) spectrum of (*E*)-**1at**.

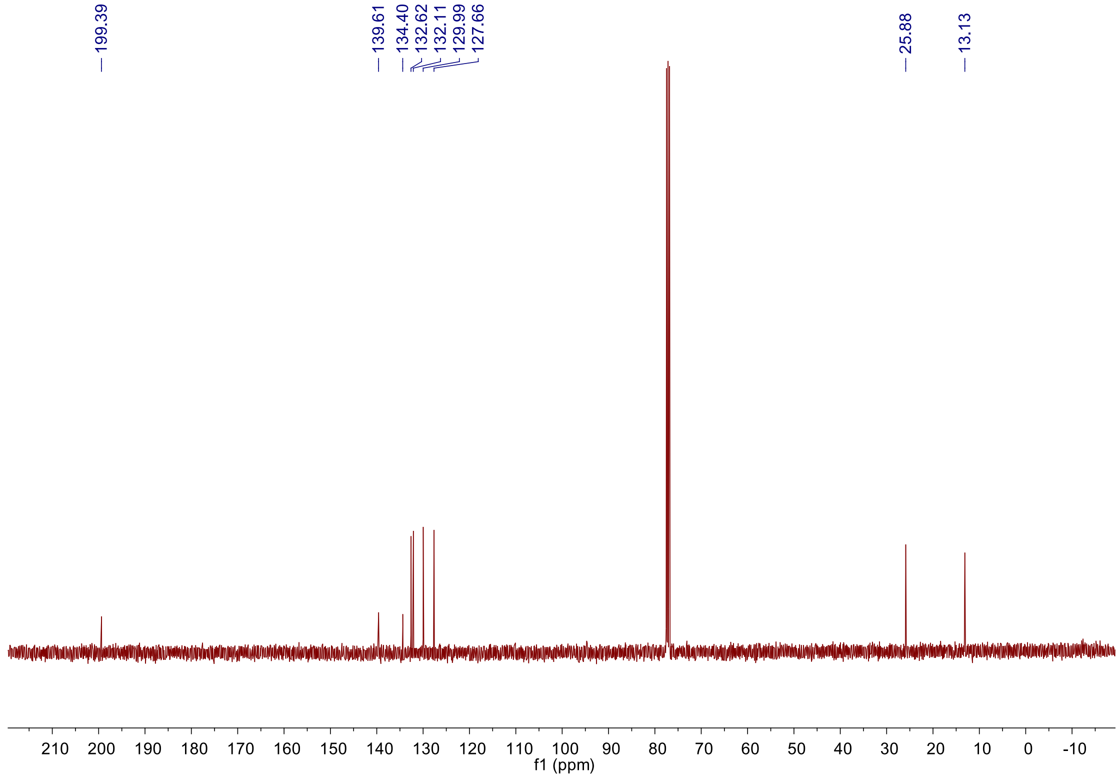

Supplementary Figure 59. ^{13}C NMR (100 MHz, $CDCl_3$) spectrum of (*E*)-**1at**.

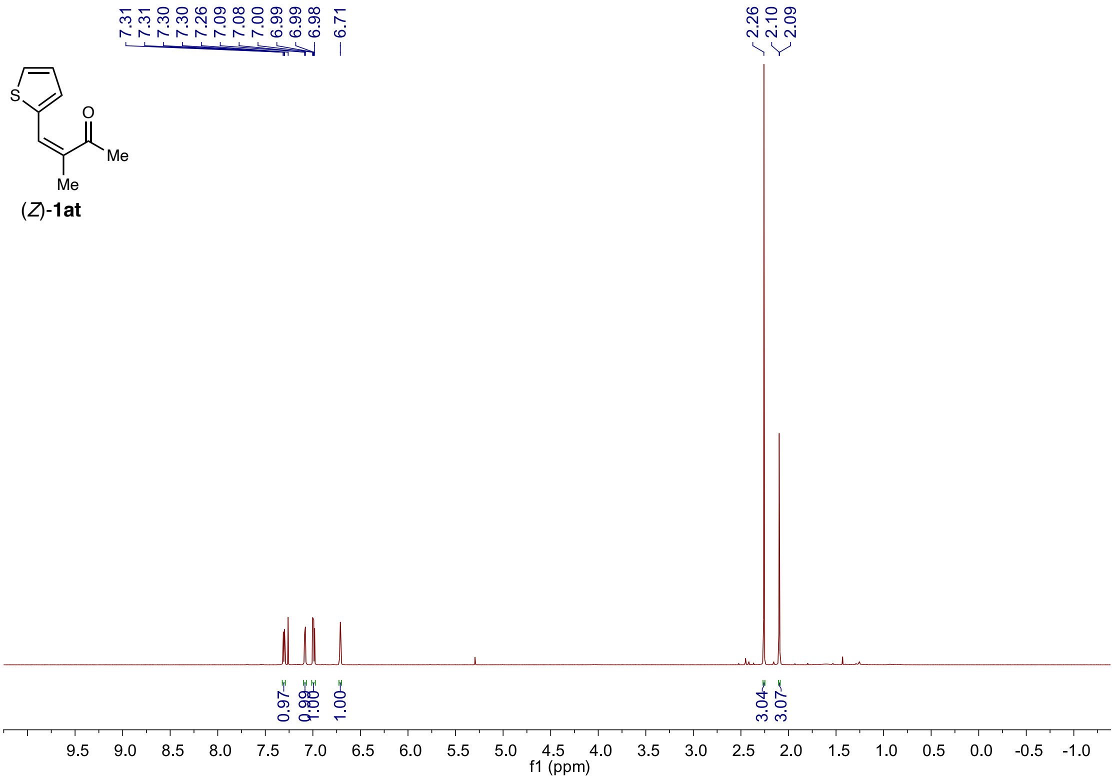

Supplementary Figure 60. ^{1}H NMR (400 MHz, $CDCl_3$) spectrum of (*Z*)-**1at**.

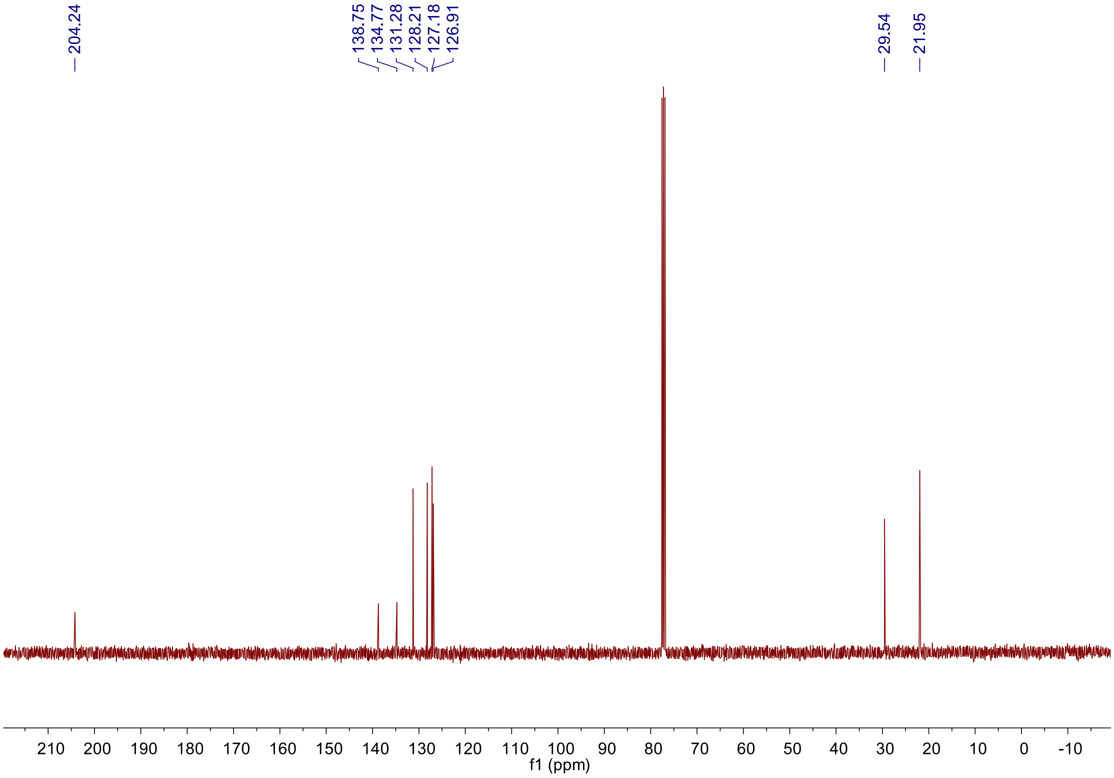

Supplementary Figure 61. ^{13}C NMR (100 MHz, $CDCl_3$) spectrum of (*Z*)-**1at**.

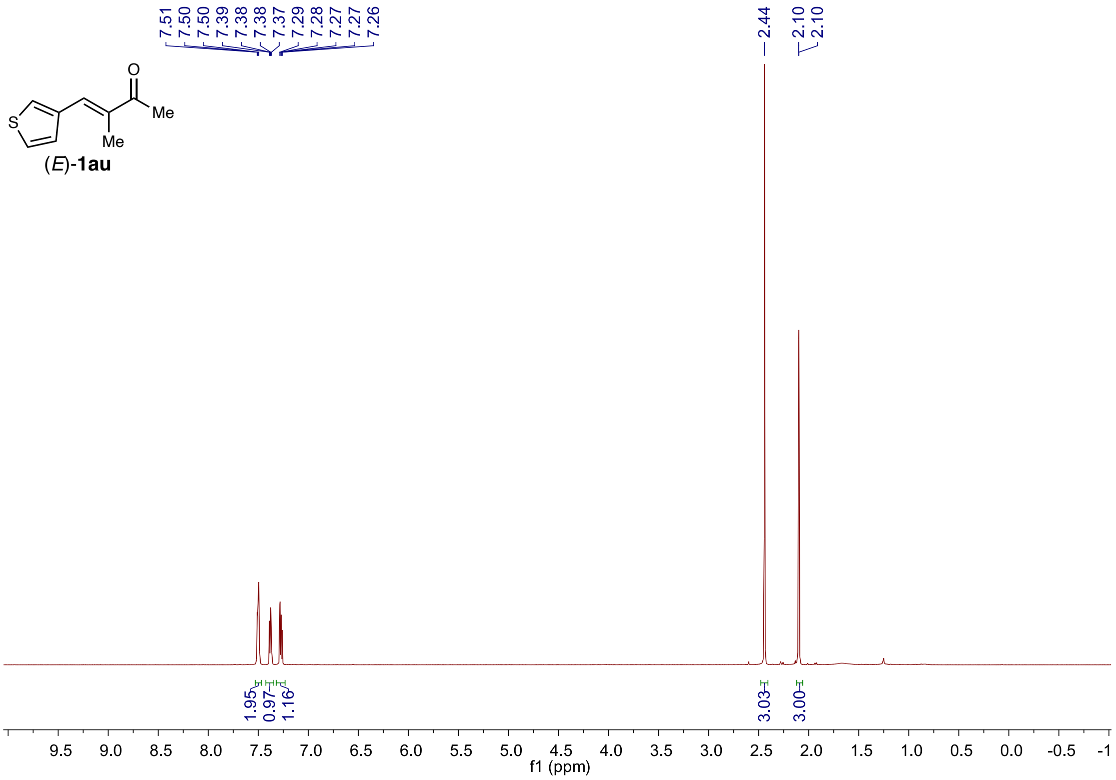

Supplementary Figure 62. ^{1}H NMR (400 MHz, $CDCl_3$) spectrum of (*E*)-**1au**.

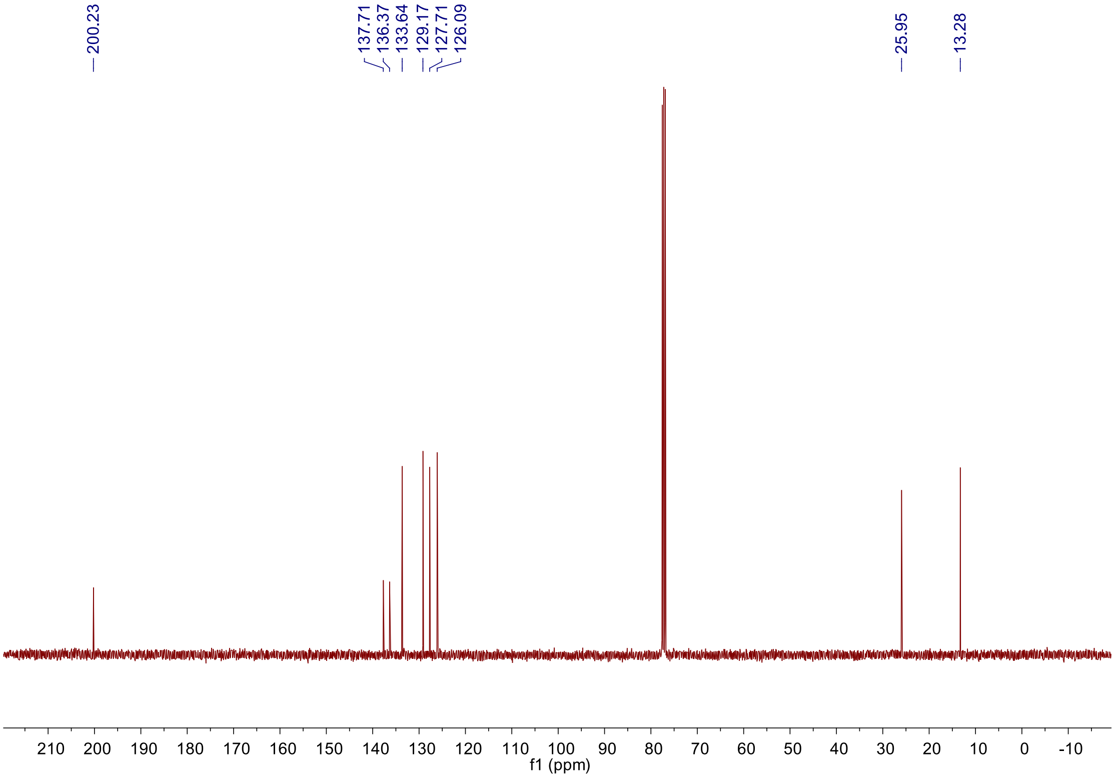

Supplementary Figure 63. ^{13}C NMR (100 MHz, $CDCl_3$) spectrum of (*E*)-**1au**.

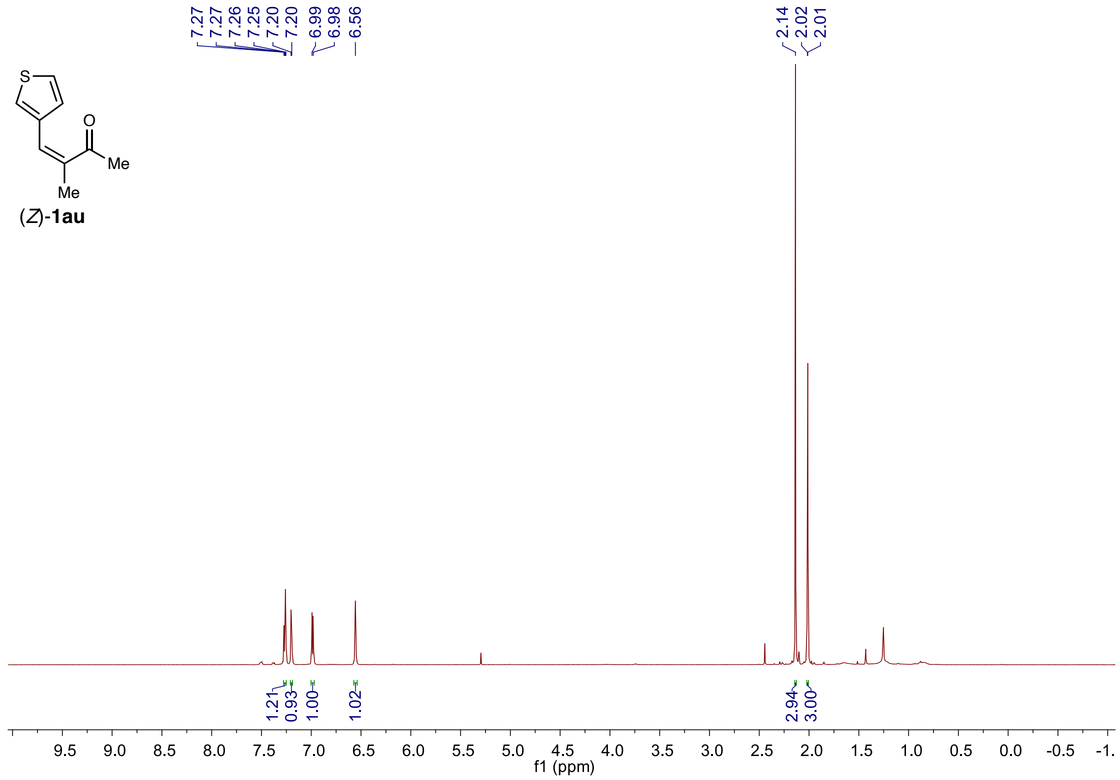

Supplementary Figure 64. ^{1}H NMR (400 MHz, $CDCl_3$) spectrum of (*Z*)-**1au**.

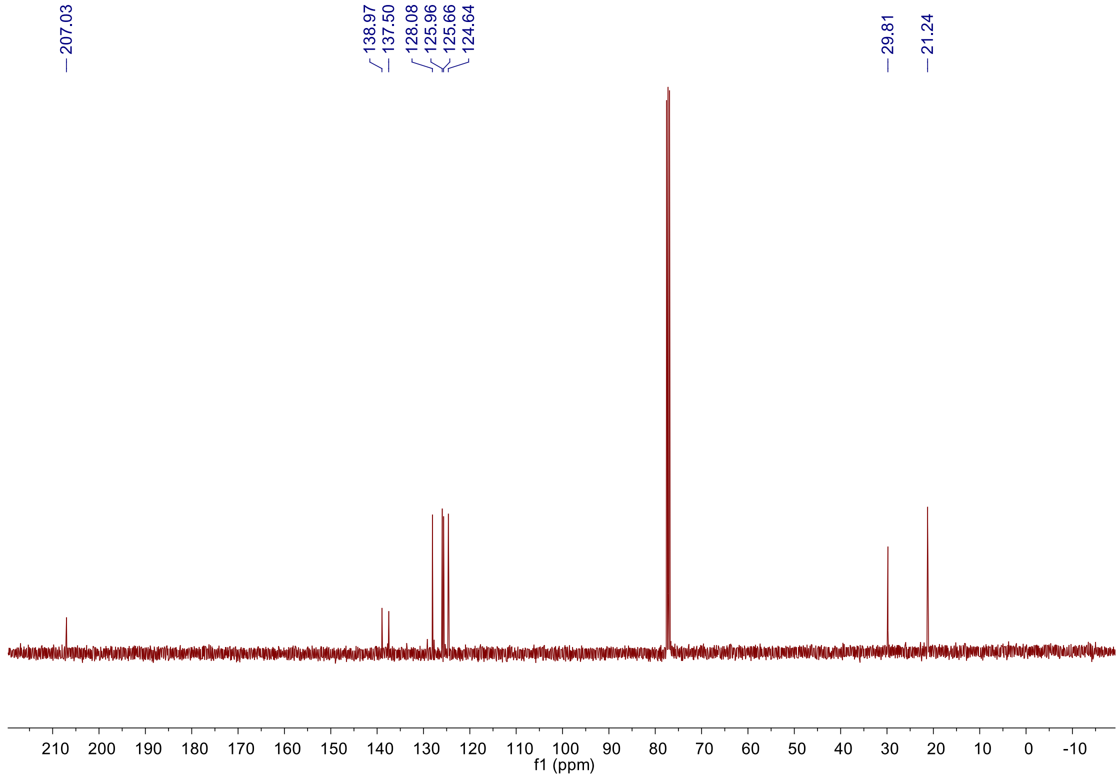

Supplementary Figure 65. ^{13}C NMR (100 MHz, $CDCl_3$) spectrum of (*Z*)-**1au**.

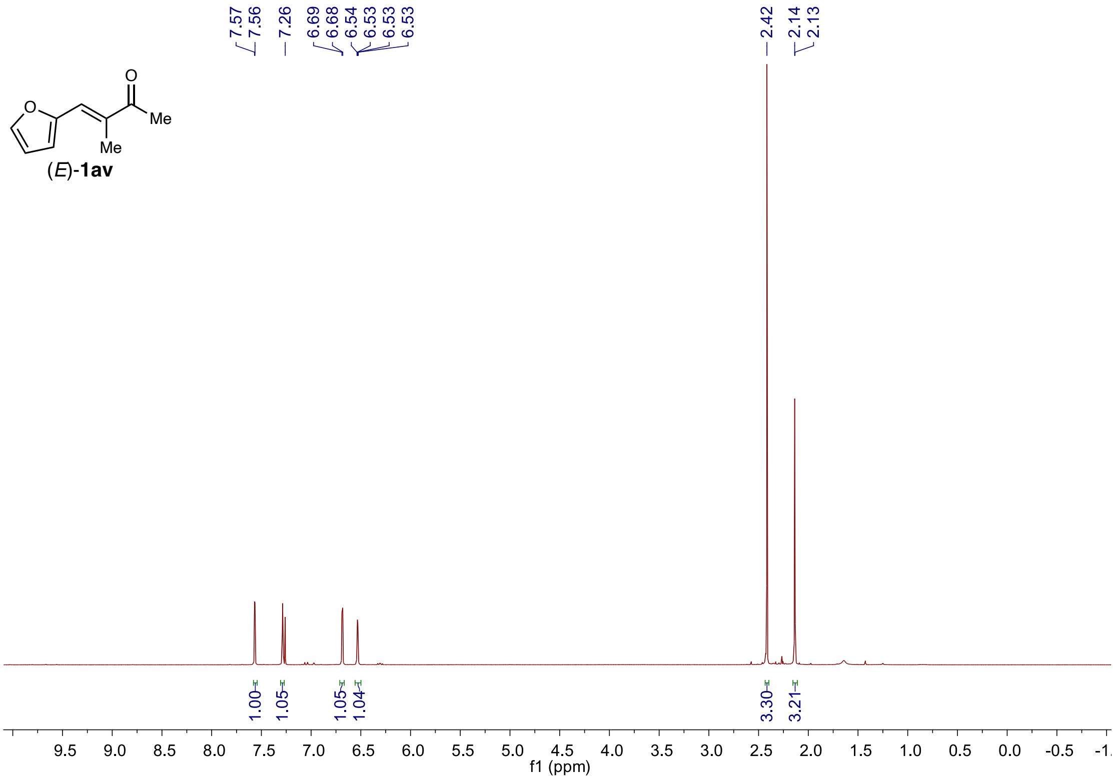

Supplementary Figure 66. ^{1}H NMR (400 MHz, $CDCl_3$) spectrum of (*E*)-**1av**.

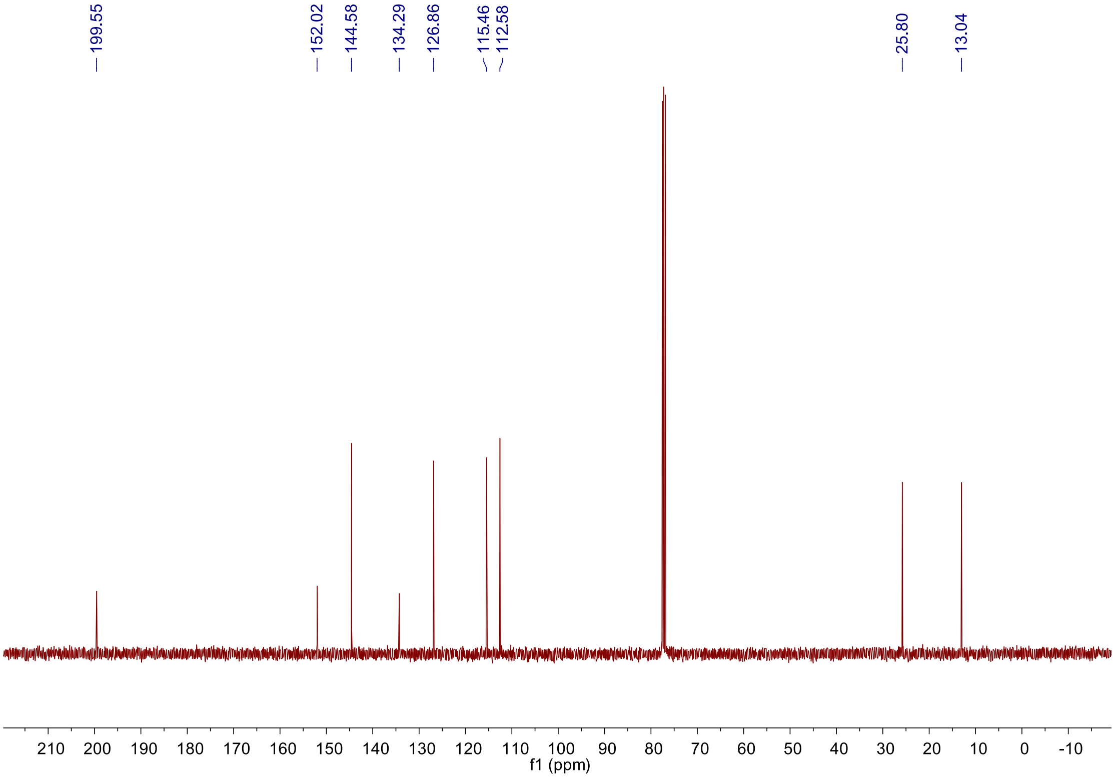

Supplementary Figure 67. ^{13}C NMR (100 MHz, $CDCl_3$) spectrum of (*E*)-**1av**.

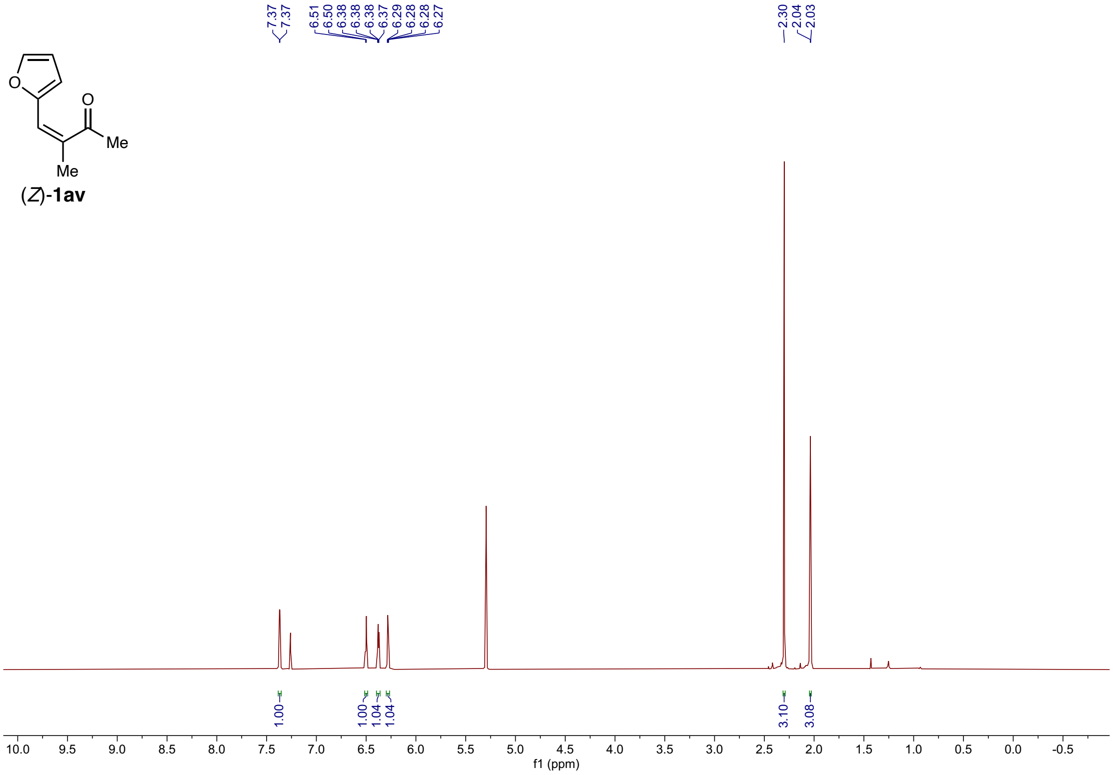

Supplementary Figure 68. ^{1}H NMR (400 MHz, $CDCl_3$) spectrum of (*Z*)-**1av**.

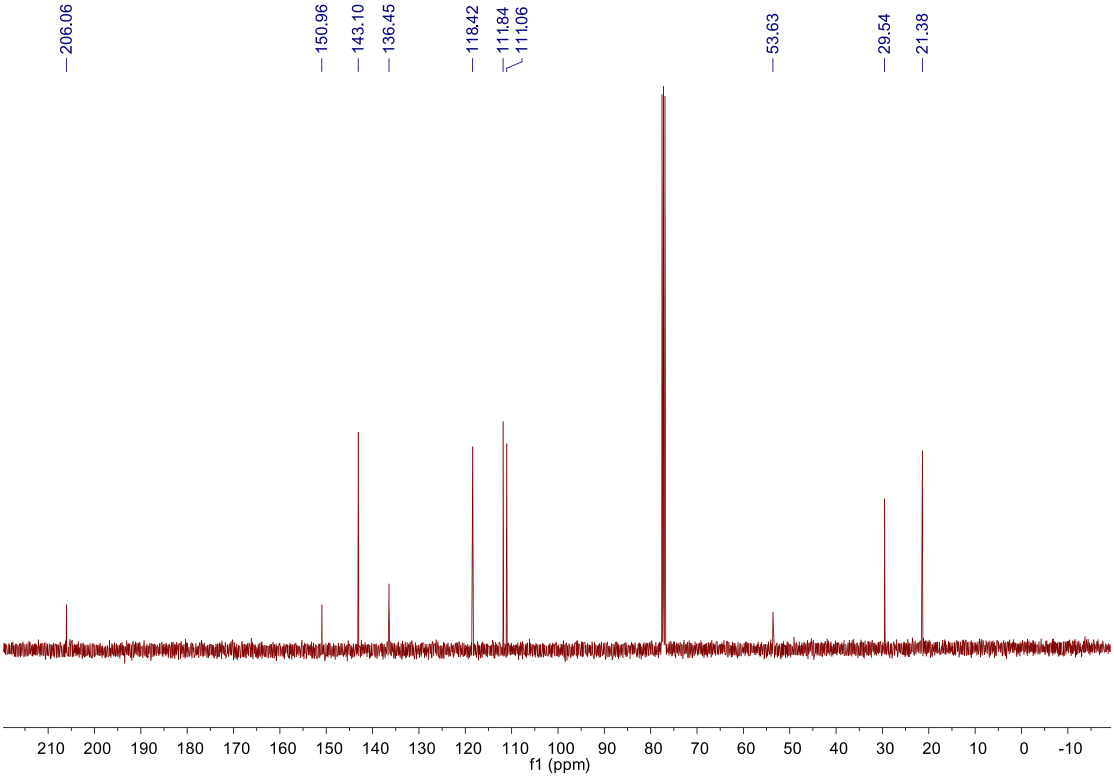

Supplementary Figure 69. ^{13}C NMR (100 MHz, $CDCl_3$) spectrum of (*Z*)-**1av**.

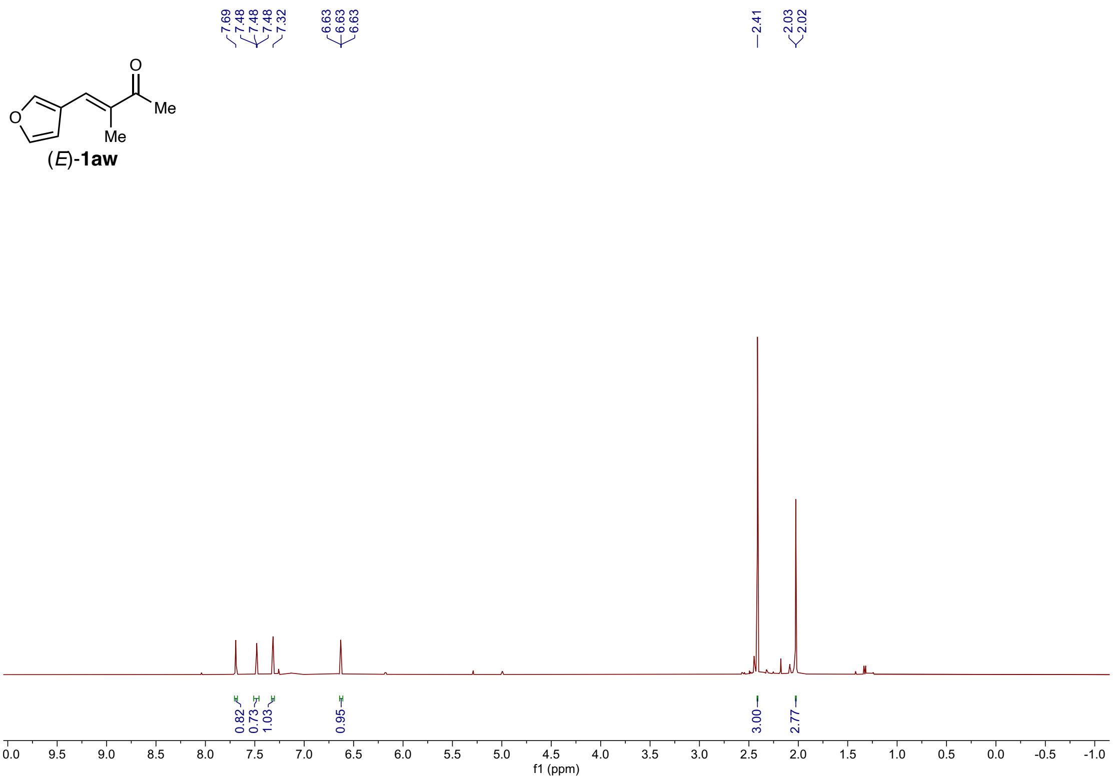

Supplementary Figure 70. ^{1}H NMR (400 MHz, $CDCl_3$) spectrum of (*E*)-**1aw**.

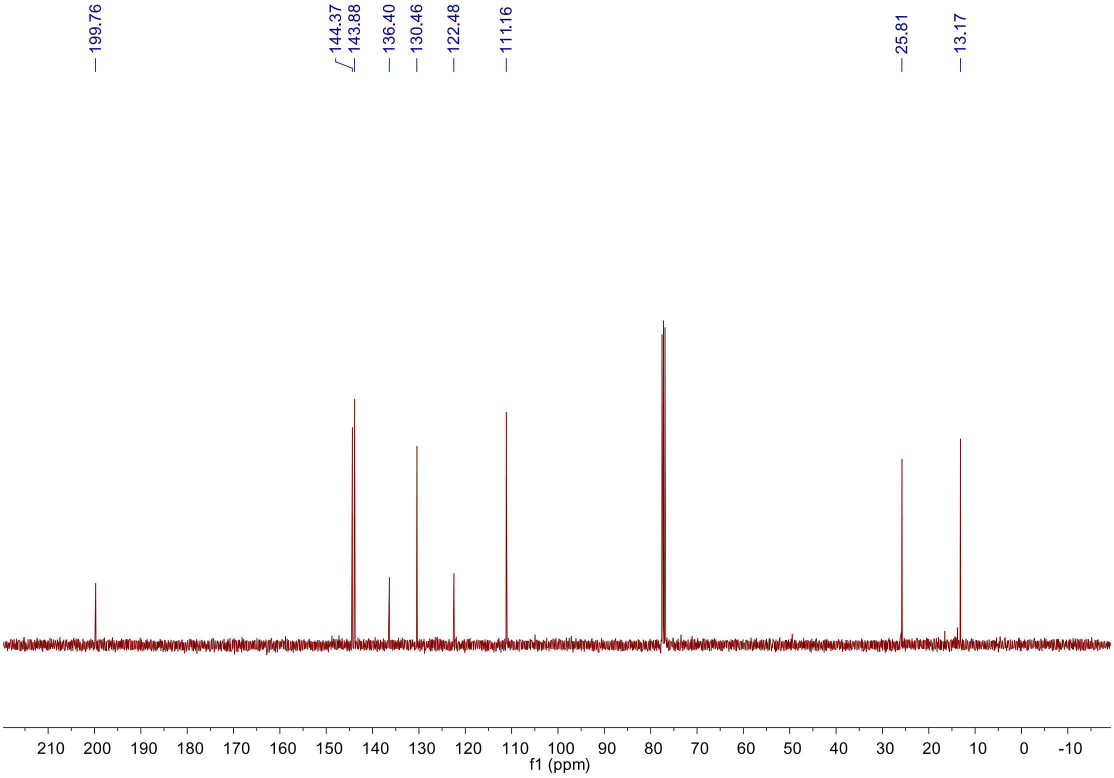

Supplementary Figure 71. ^{13}C NMR (100 MHz, $CDCl_3$) spectrum of (*E*)-**1aw**.

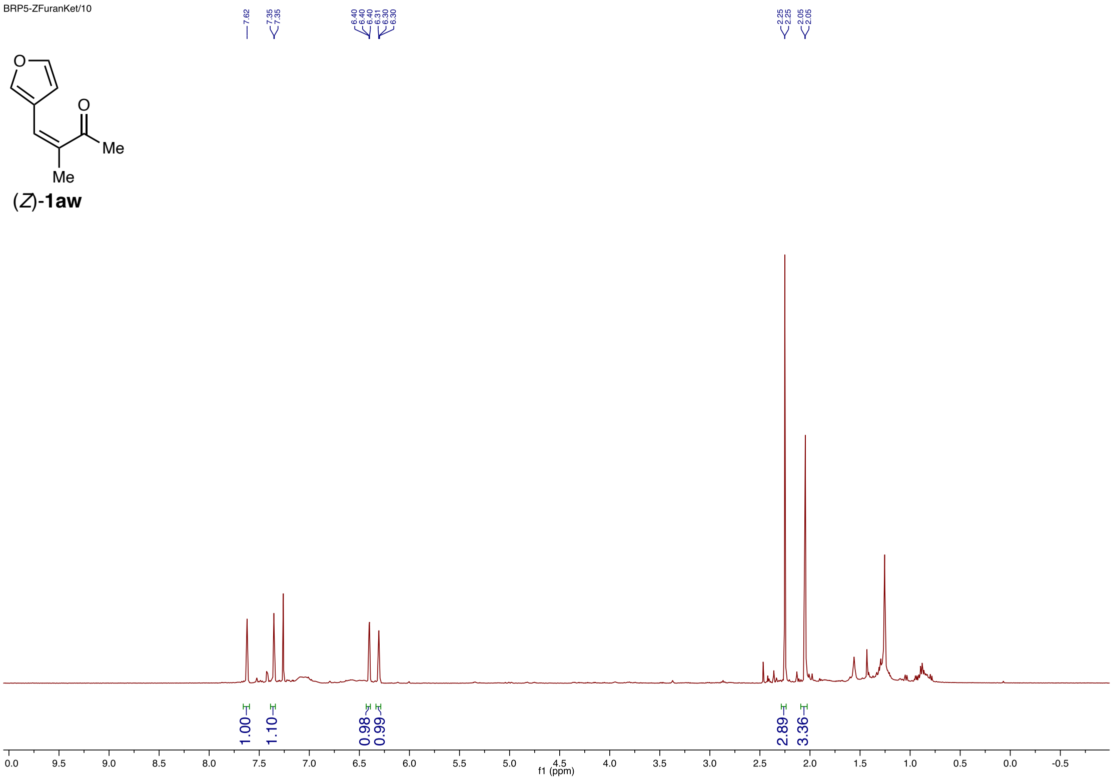

Supplementary Figure 72. ^{1}H NMR (400 MHz, $CDCl_3$) spectrum of (*Z*)-**1aw**.

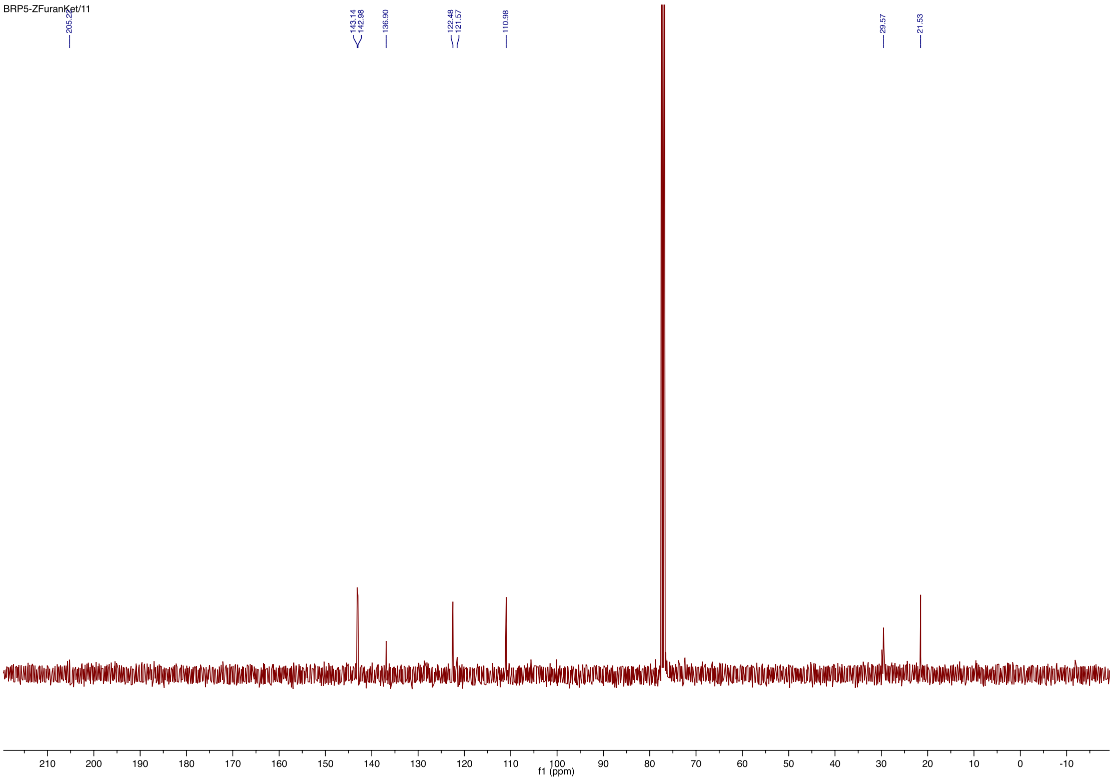

Supplementary Figure 73. ^{13}C NMR (100 MHz, $CDCl_3$) spectrum of (*Z*)-**1aw**.

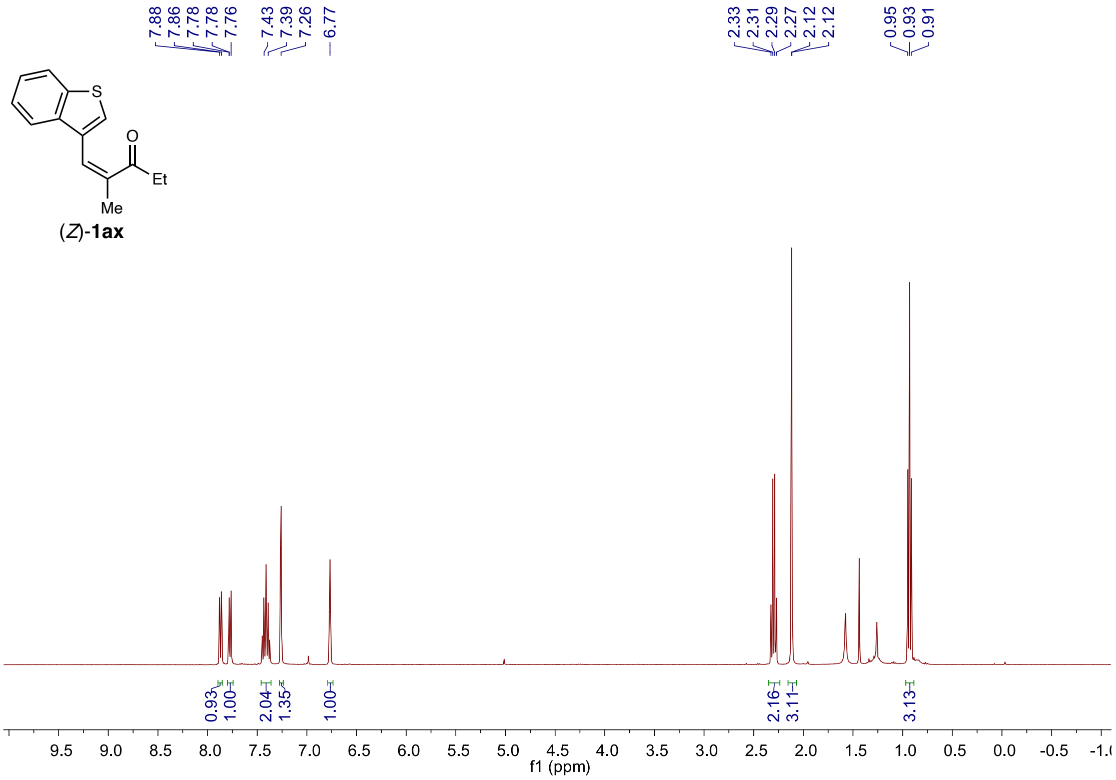

Supplementary Figure 74. ^{1}H NMR (400 MHz, $CDCl_3$) spectrum of (*Z*)-**1ax**.

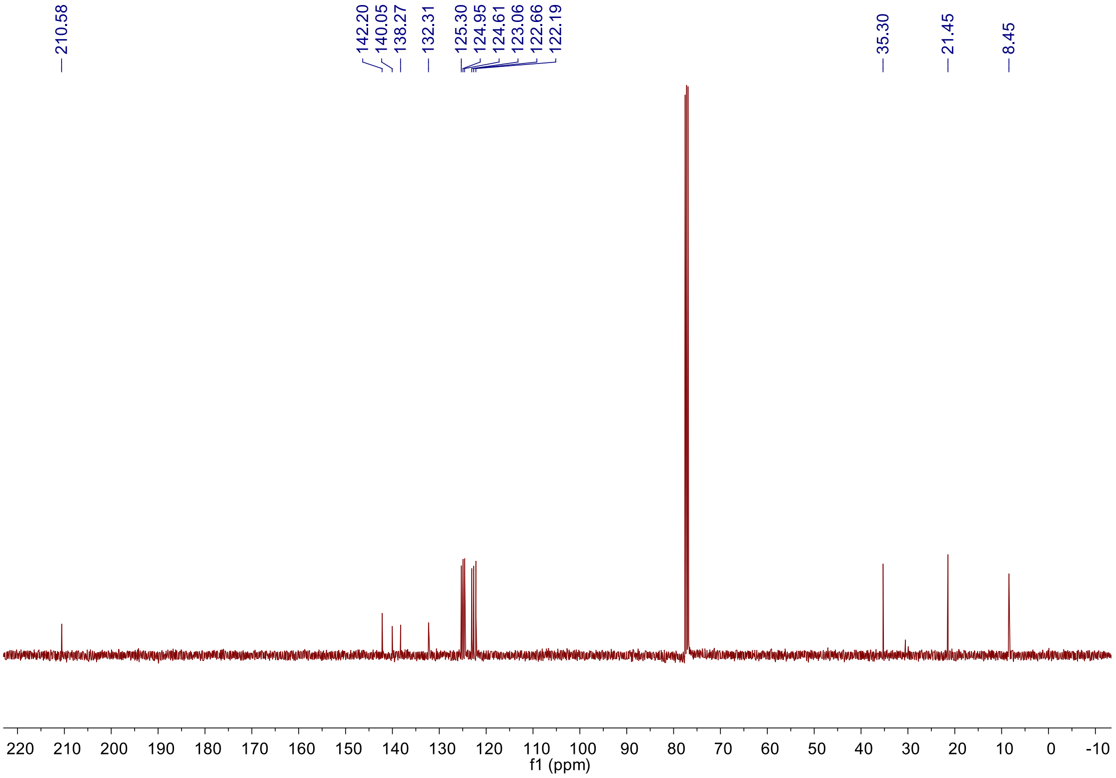

Supplementary Figure 75. ^{13}C NMR (100 MHz, $CDCl_3$) spectrum of (*Z*)-**1ax**.

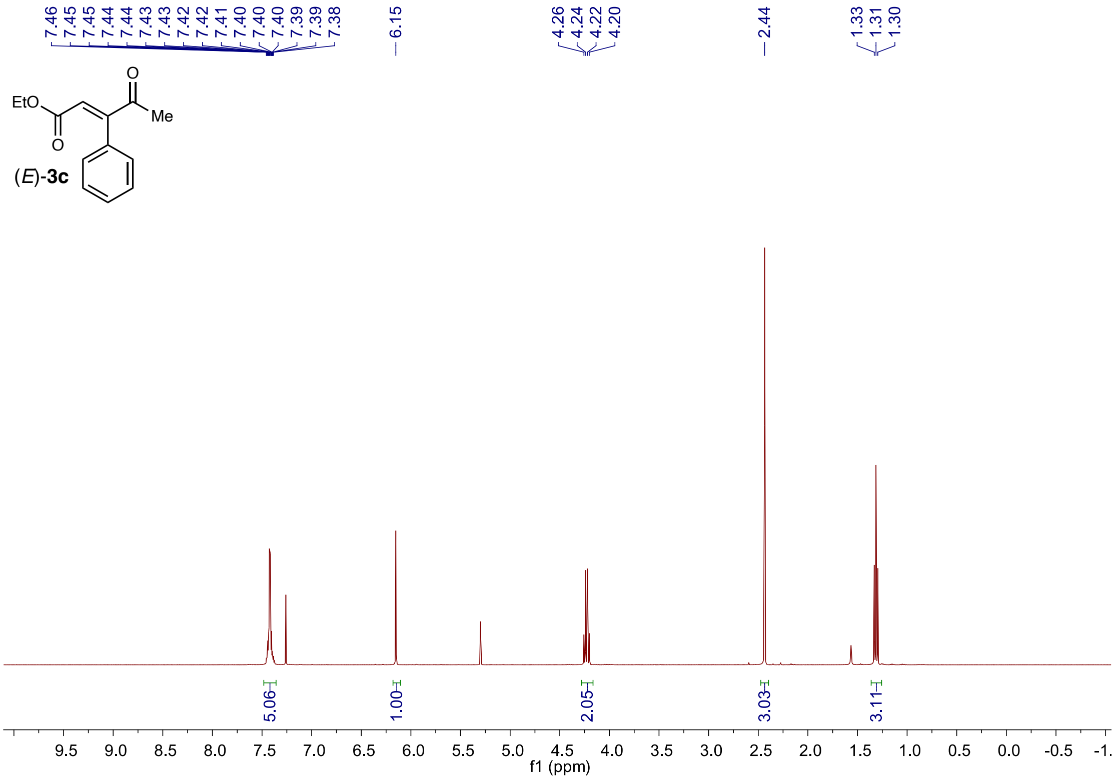

Supplementary Figure 76. ^{1}H NMR (400 MHz, $CDCl_3$) spectrum of (*E*)-**3c**.

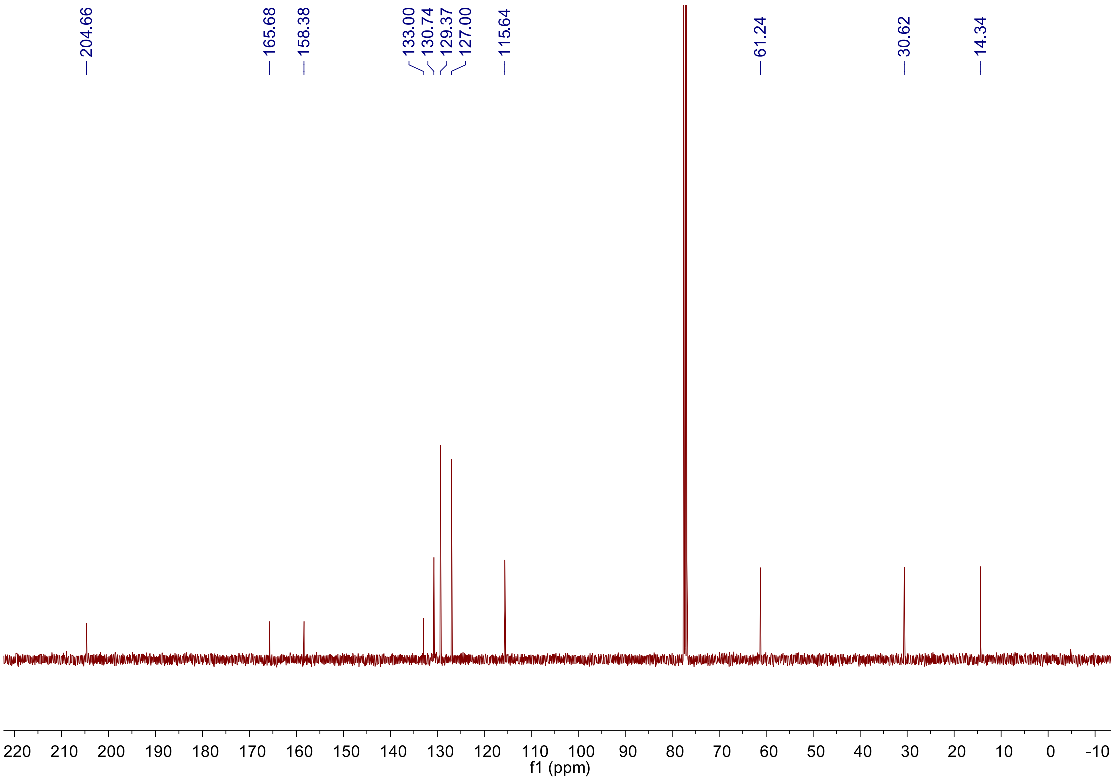

Supplementary Figure 77. ^{13}C NMR (100 MHz, $CDCl_3$) spectrum of (*E*)-**3c**.

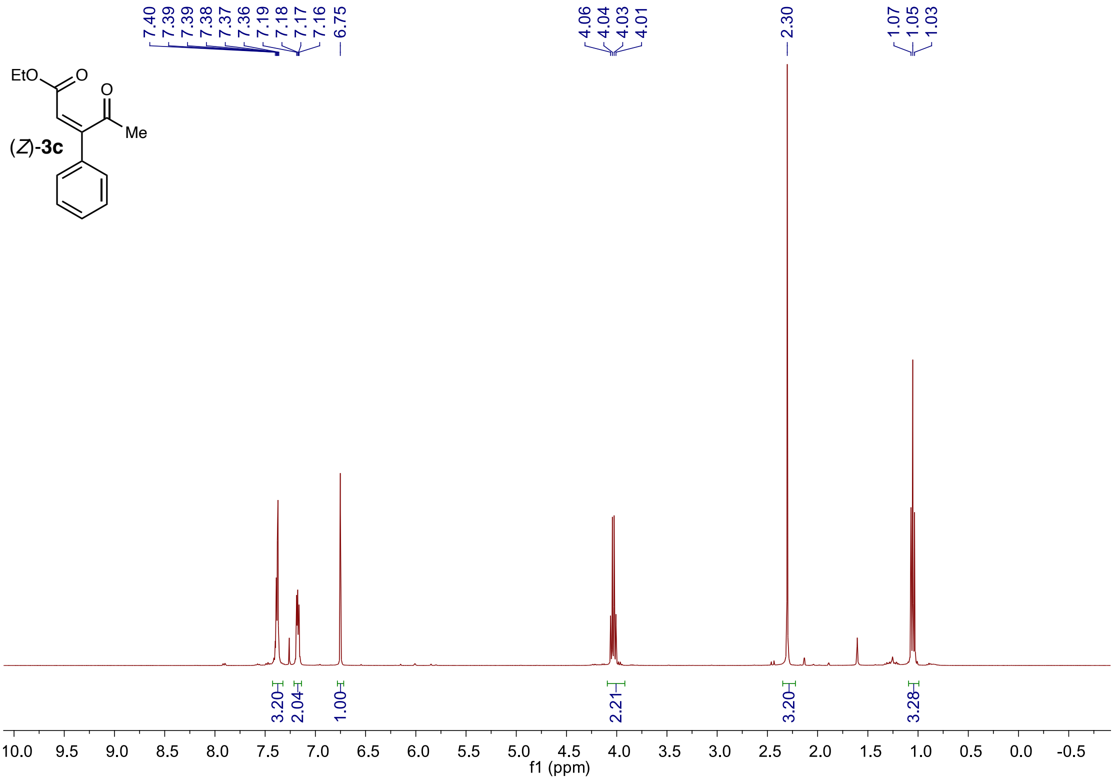

Supplementary Figure 78. ^{1}H NMR (400 MHz, $CDCl_3$) spectrum of (*Z*)-**3c**.

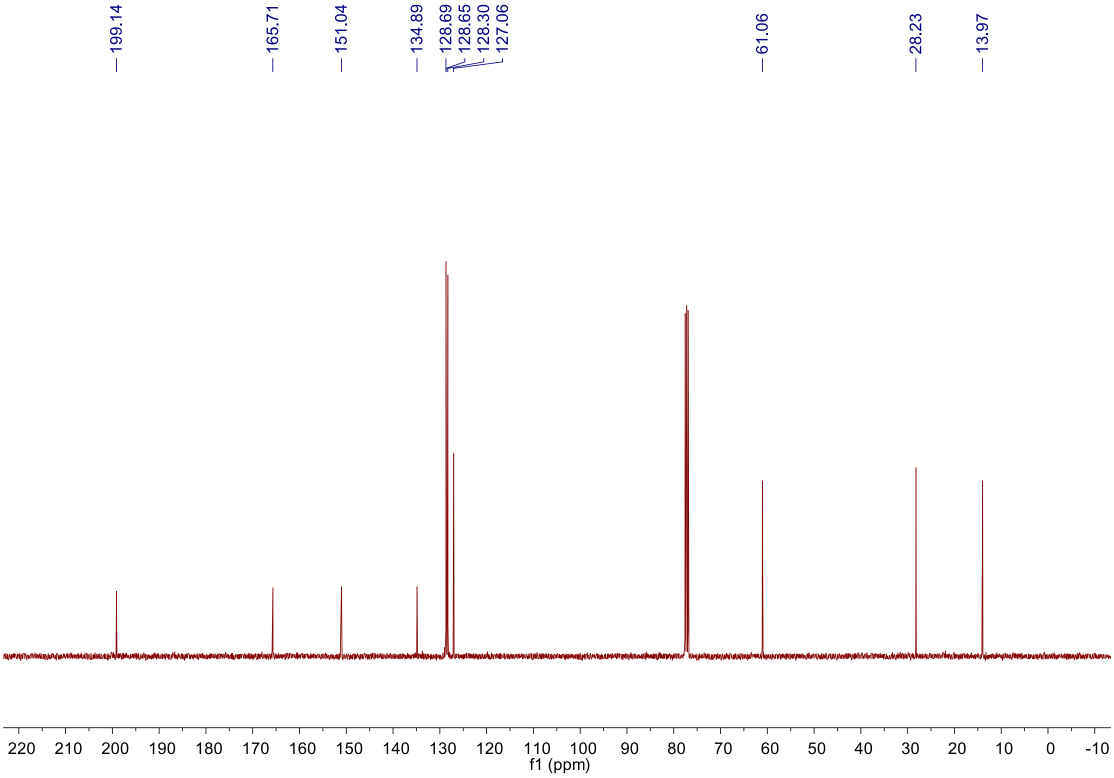

Supplementary Figure 79. ^{13}C NMR (100 MHz, $CDCl_3$) spectrum of (*Z*)-**3c**.

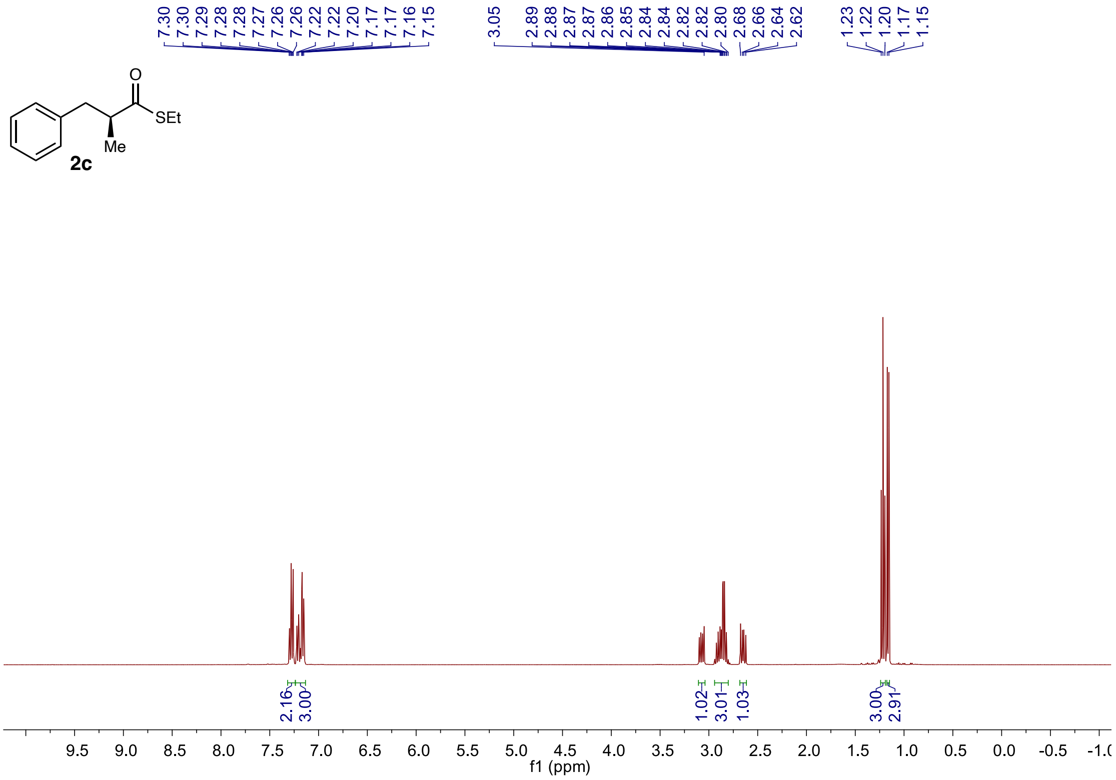

Supplementary Figure 80. ^{1}H NMR (400 MHz, $CDCl_3$) spectrum of **2c**.

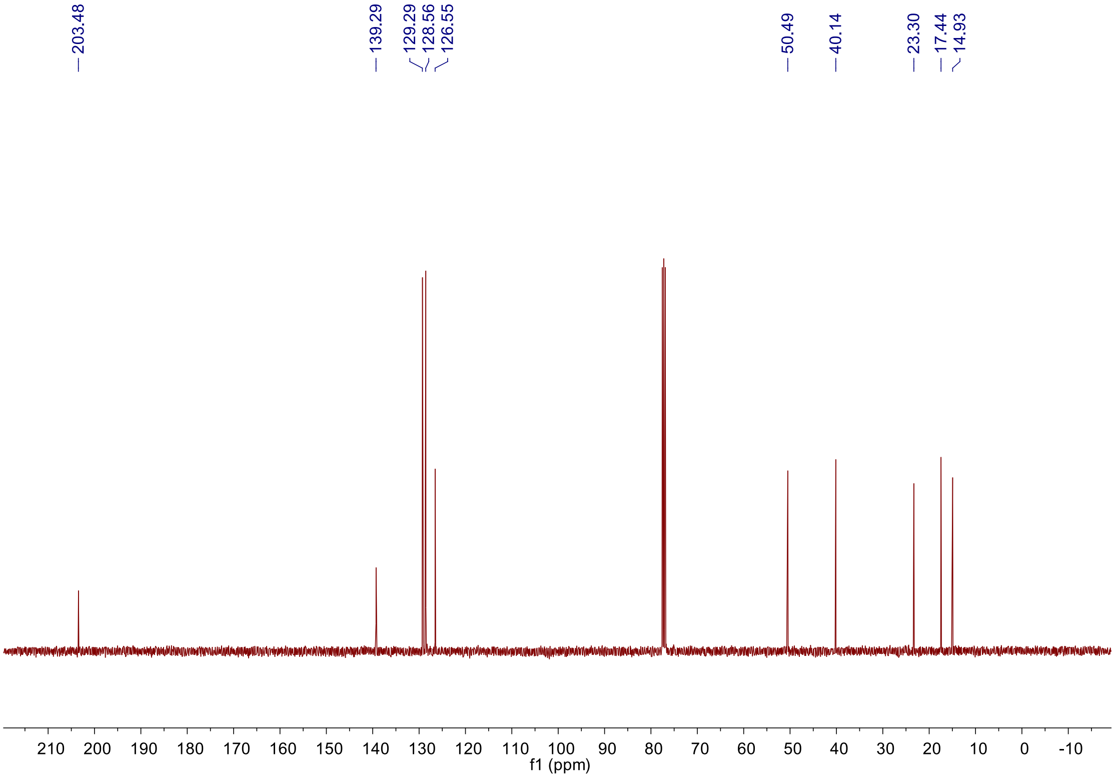

Supplementary Figure 81. ^{13}C NMR (100 MHz, $CDCl_3$) spectrum of **2c**.

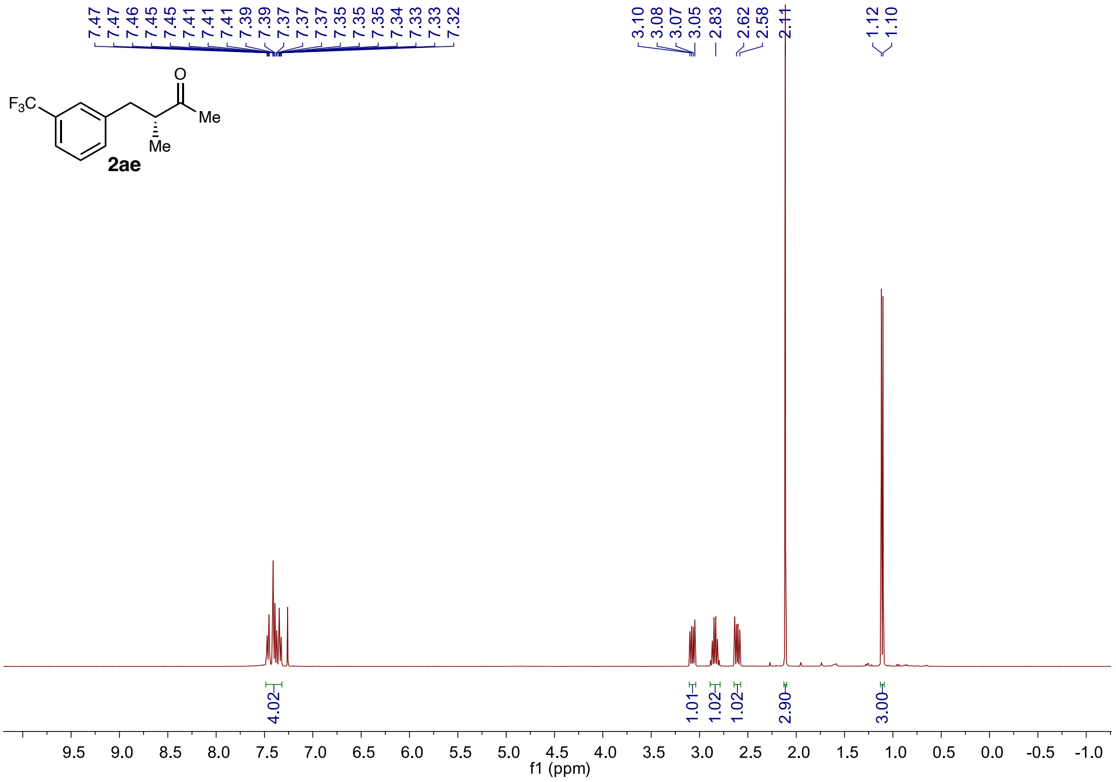

Supplementary Figure 82. ^{1}H NMR (400 MHz, $CDCl_3$) spectrum of **2ae**.

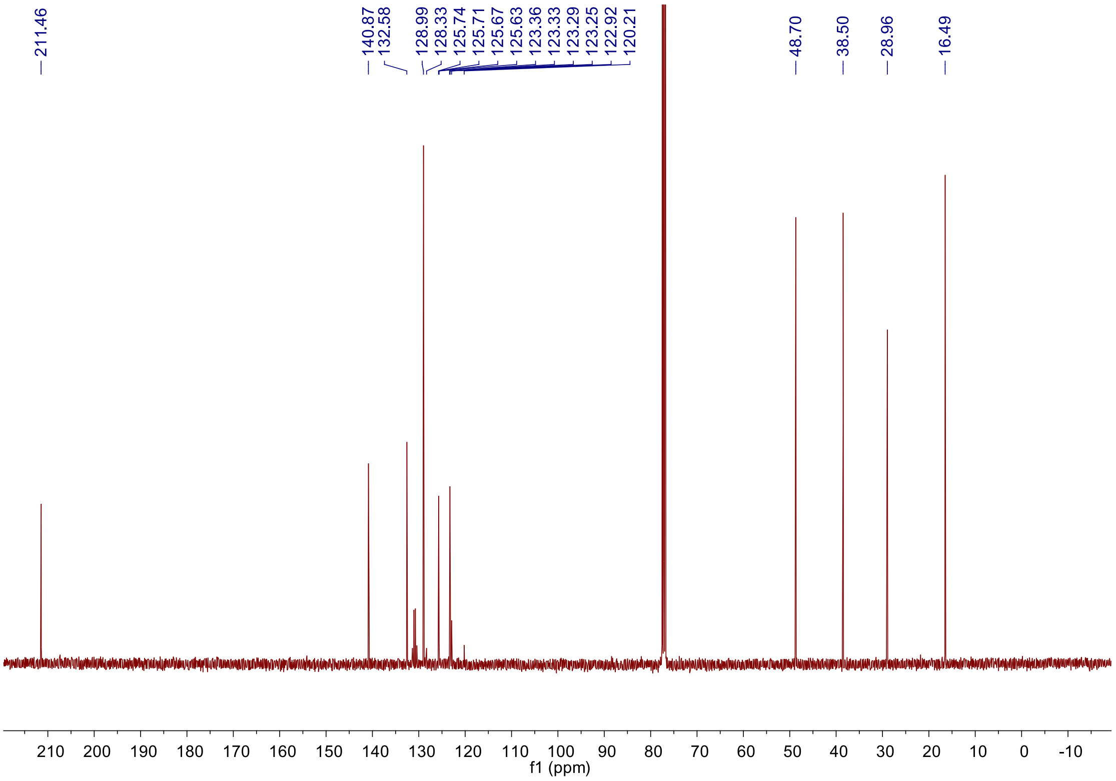

Supplementary Figure 83. ^{13}C NMR (100 MHz, $CDCl_3$) spectrum of **2ae**.

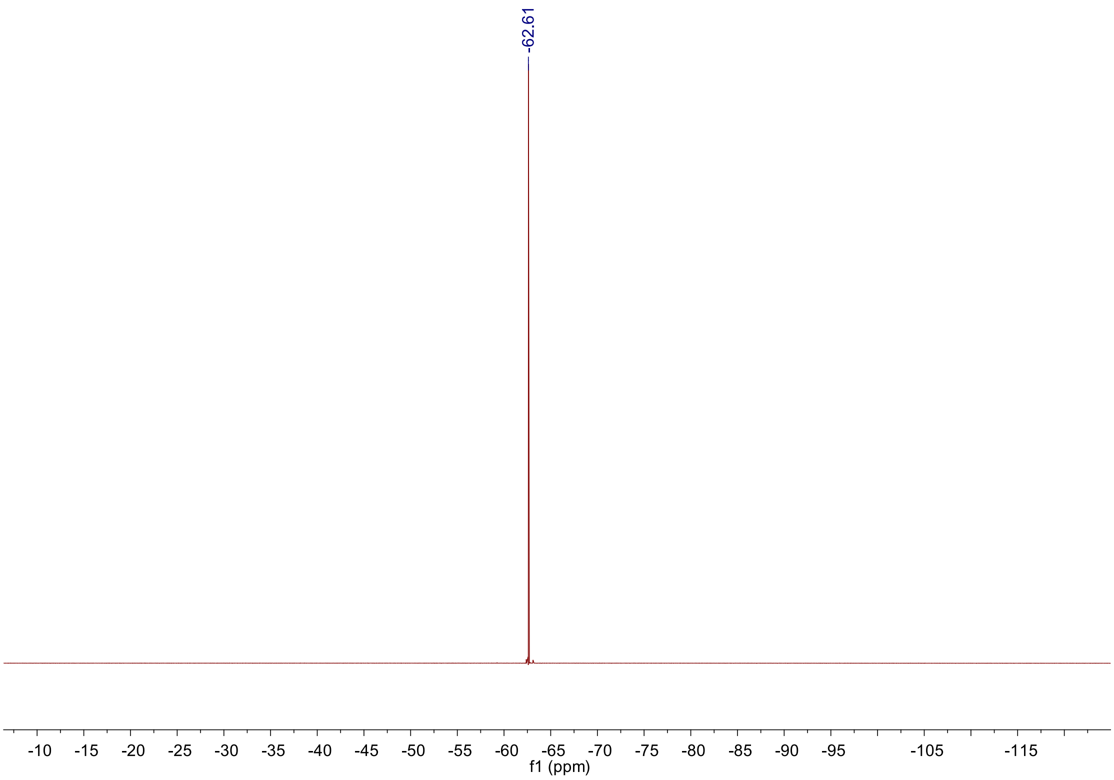

Supplementary Figure 84. ^{19}F NMR (377 MHz, $CDCl_3$) spectrum of **2ae**.

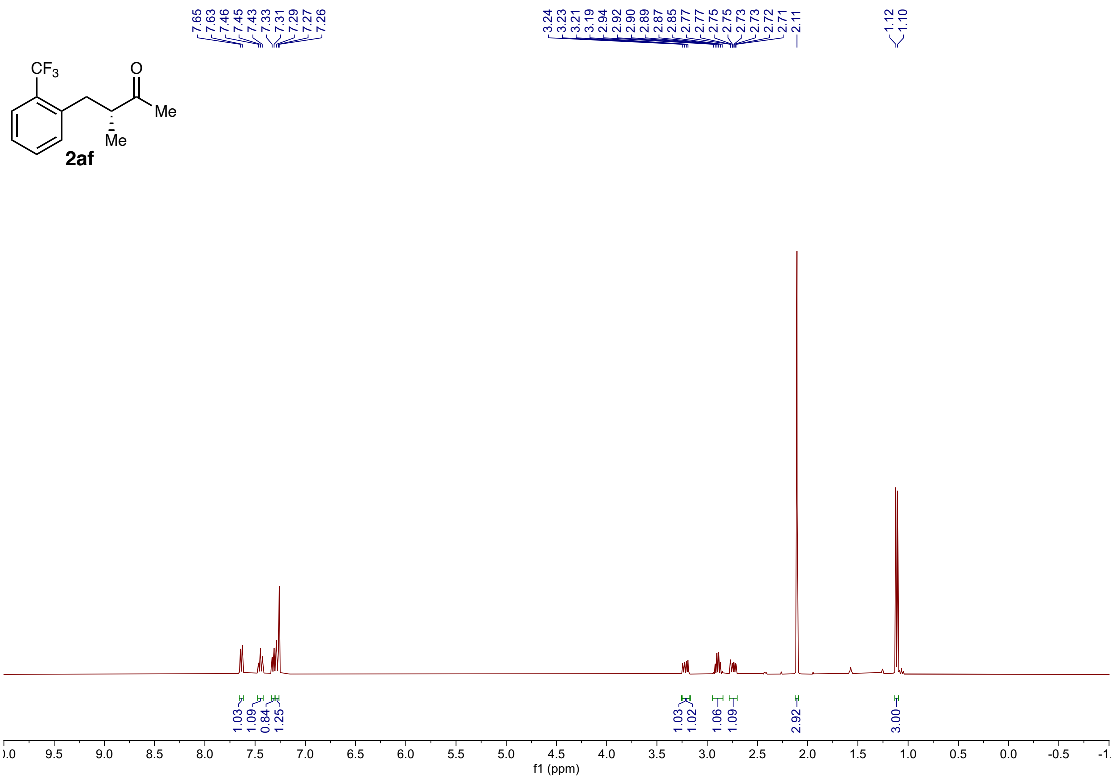

Supplementary Figure 85. ^{1}H NMR (400 MHz, $CDCl_3$) spectrum of **2af**.

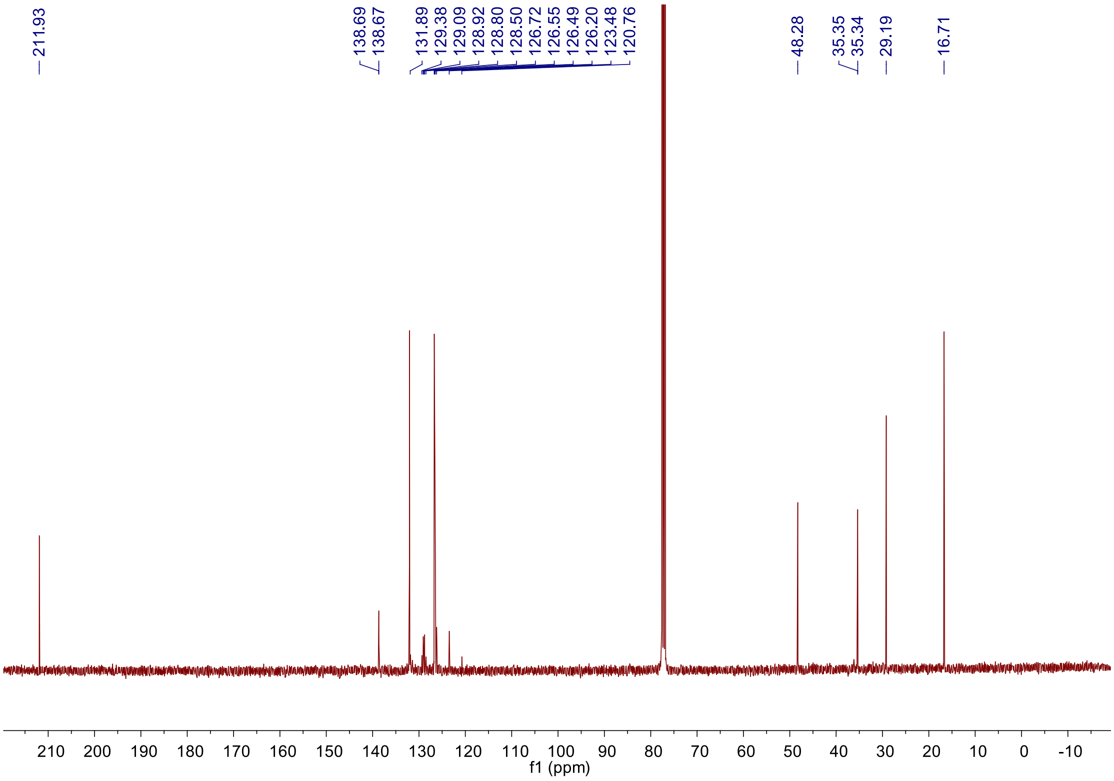

Supplementary Figure 86. ^{13}C NMR (100 MHz, $CDCl_3$) spectrum of **2af**.

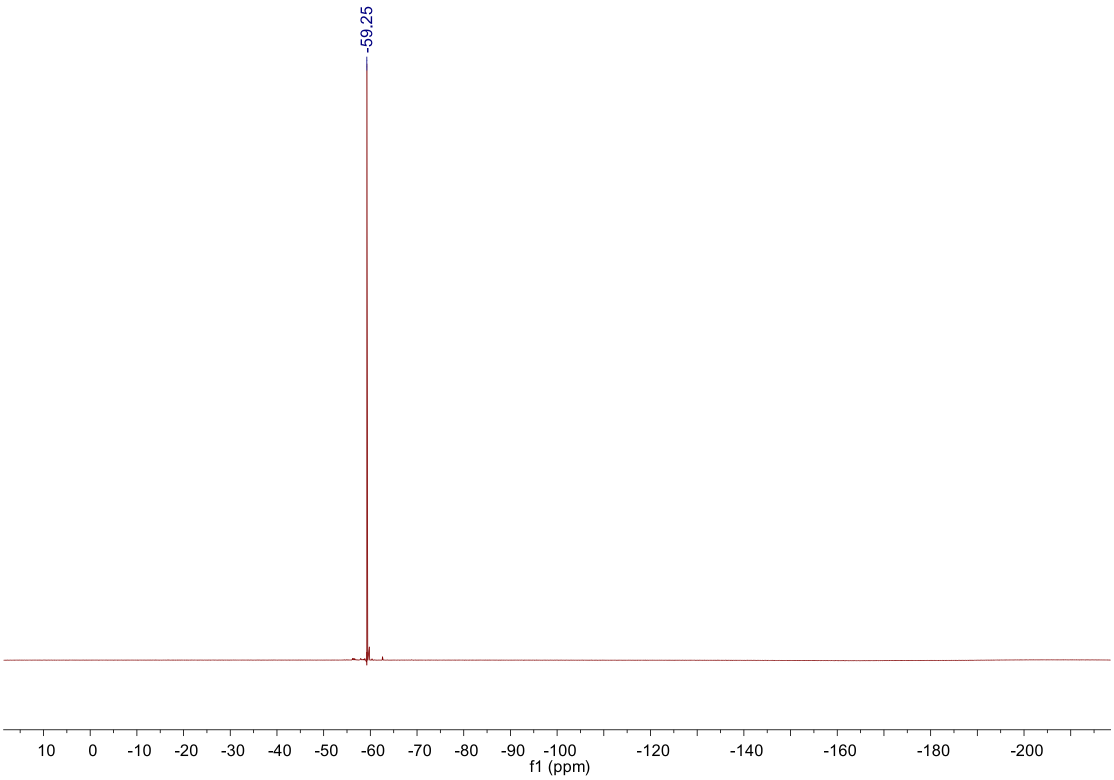

Supplementary Figure 87. ^{19}F NMR (377 MHz, $CDCl_3$) spectrum of **2af**.

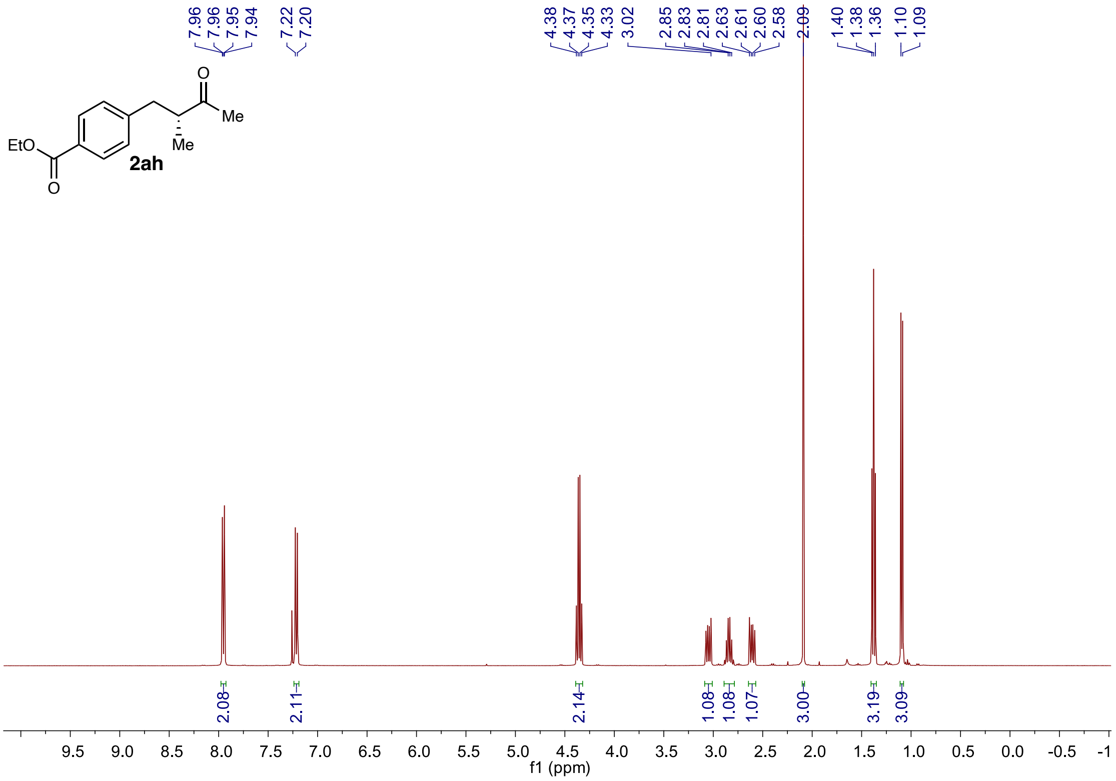

Supplementary Figure 88. ^{1}H NMR (400 MHz, $CDCl_3$) spectrum of **2ah**.

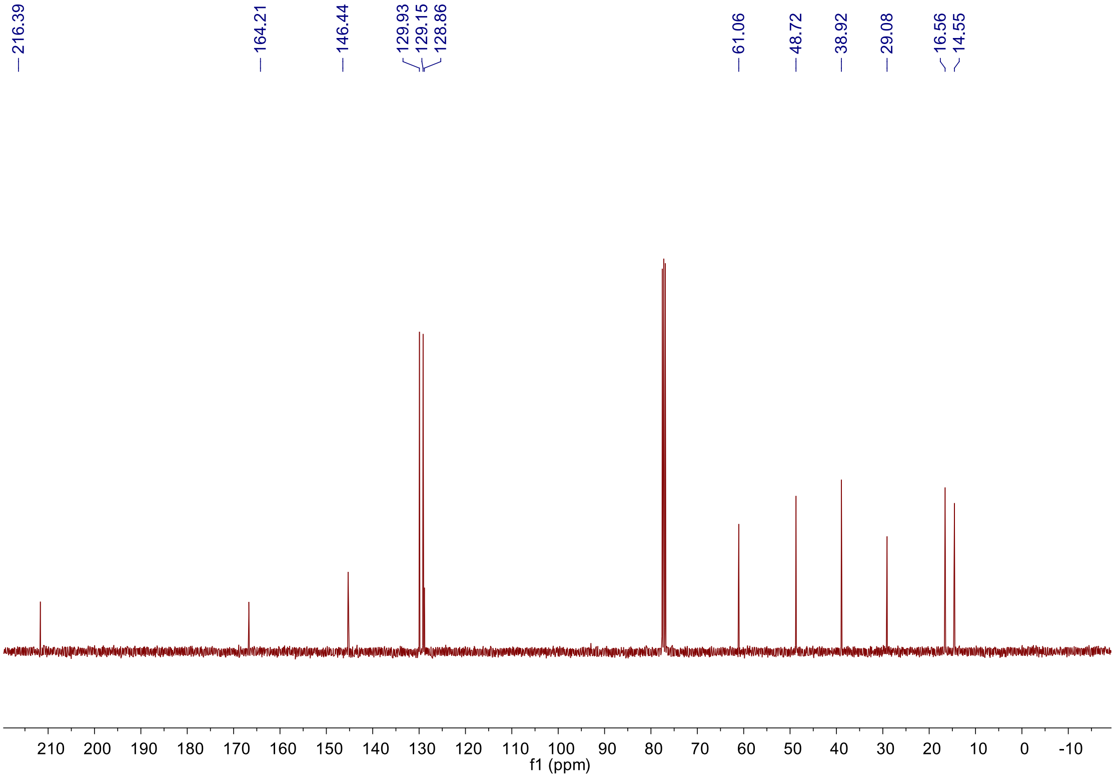

Supplementary Figure 89. ^{13}C NMR (100 MHz, $CDCl_3$) spectrum of **2ah**.

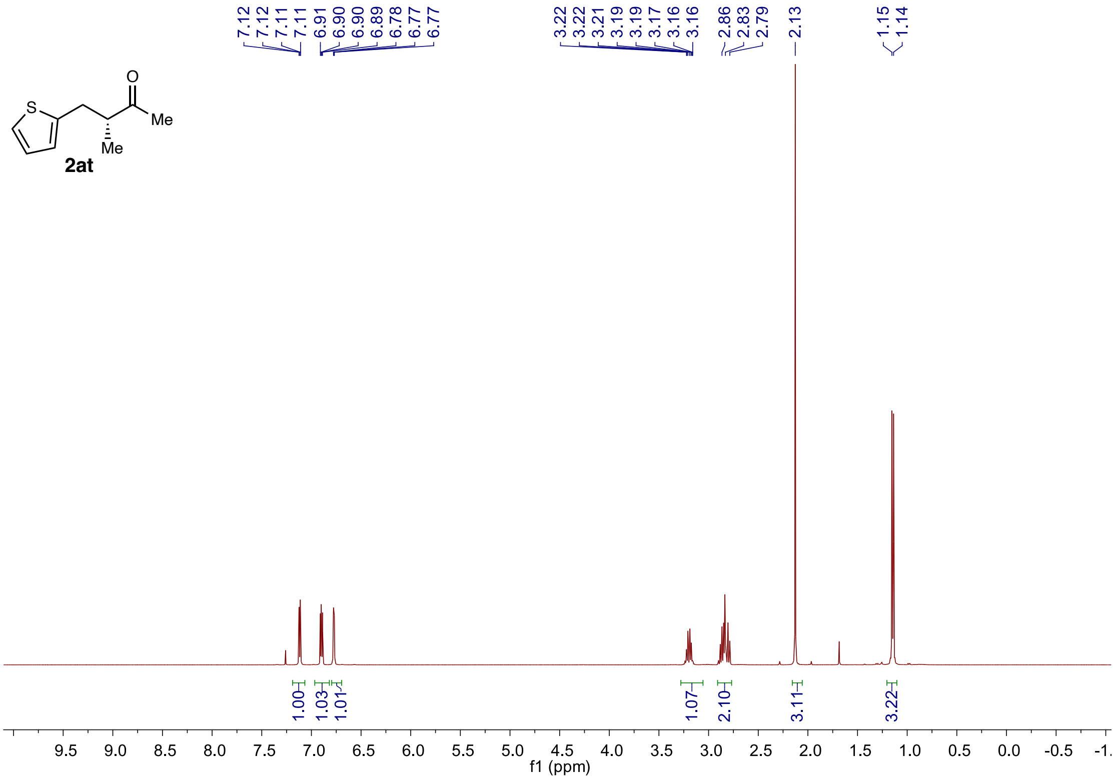

Supplementary Figure 90. ^{1}H NMR (400 MHz, $CDCl_3$) spectrum of **2at**.

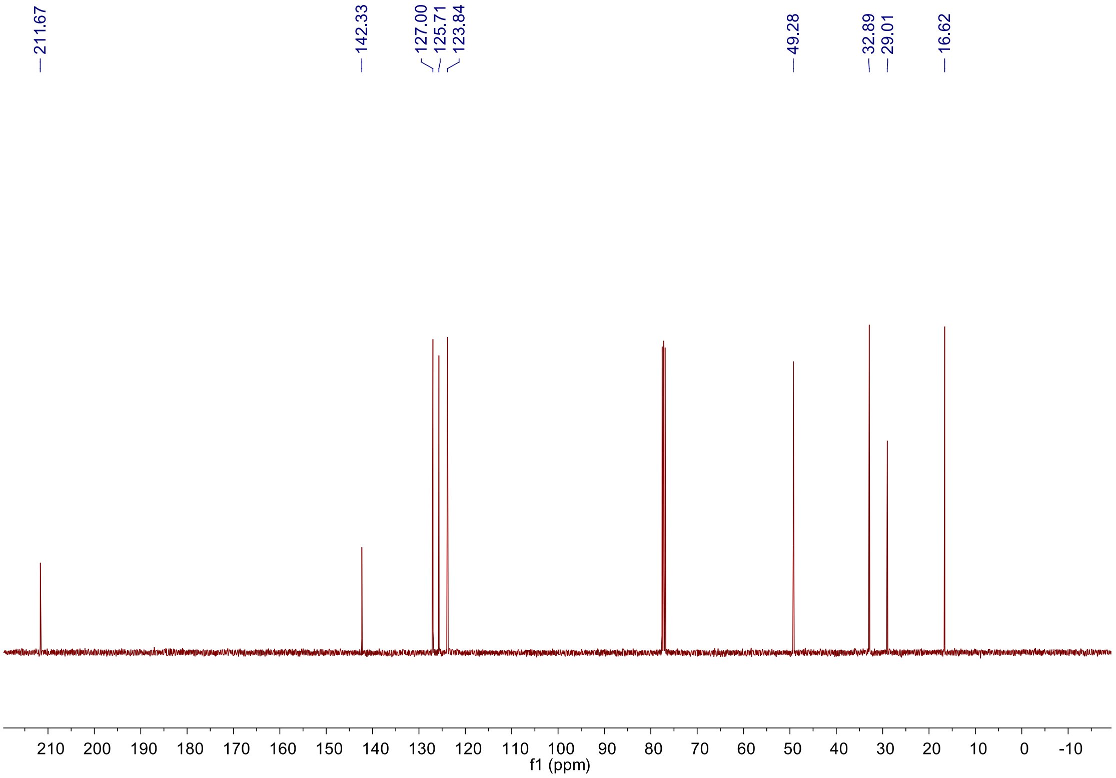

Supplementary Figure 91. ^{13}C NMR (100 MHz, $CDCl_3$) spectrum of **2at**.

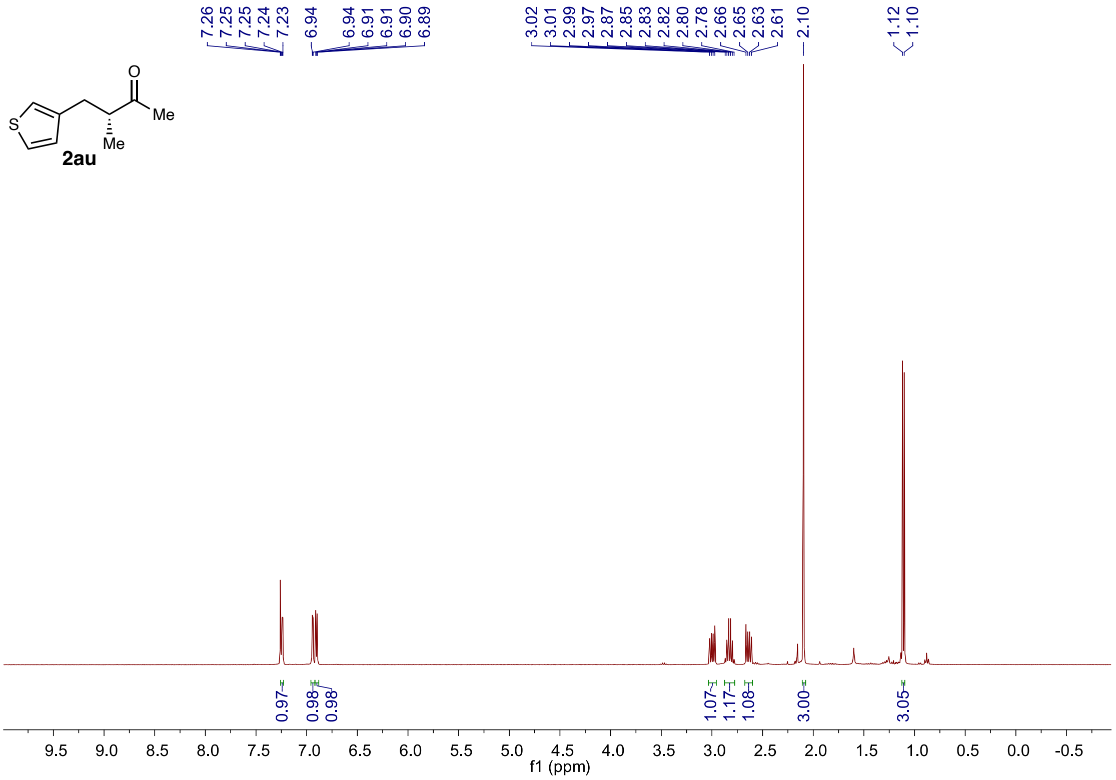

Supplementary Figure 92. ^{1}H NMR (400 MHz, $CDCl_3$) spectrum of **2au**.

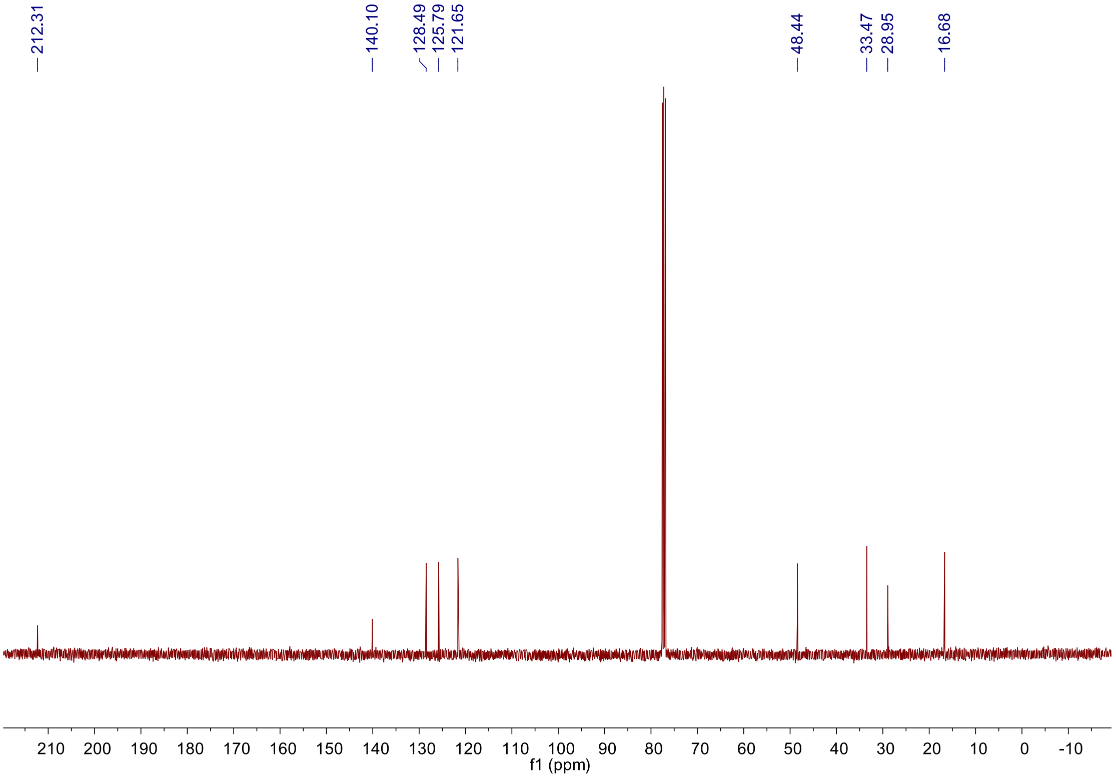

Supplementary Figure 93. ^{13}C NMR (100 MHz, $CDCl_3$) spectrum of **2au**.

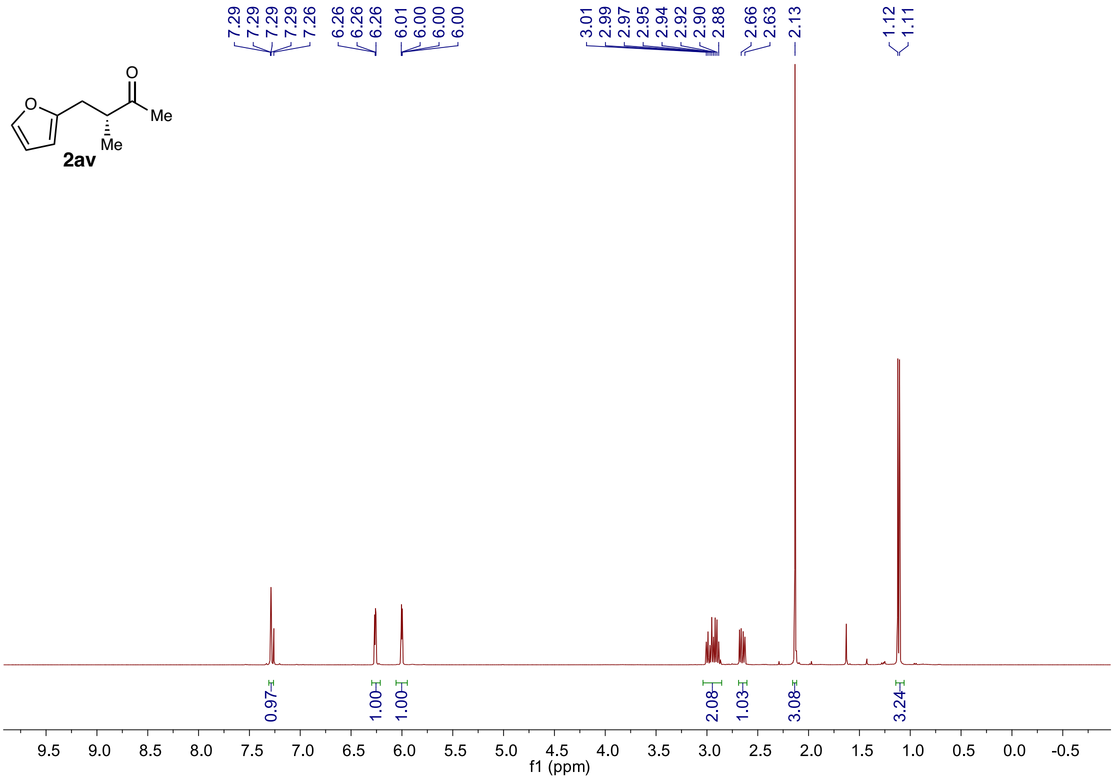

Supplementary Figure 94. ^{1}H NMR (400 MHz, $CDCl_3$) spectrum of **2av**.

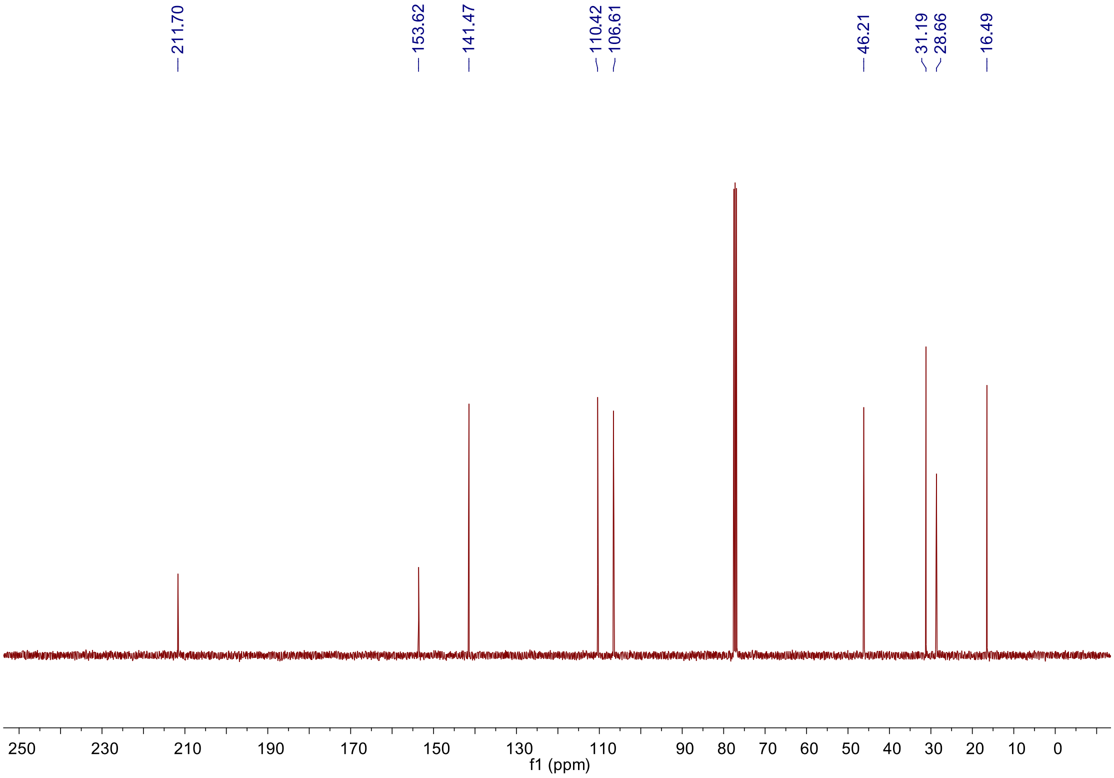

Supplementary Figure 95. ^{13}C NMR (100 MHz, $CDCl_3$) spectrum of **2av**.

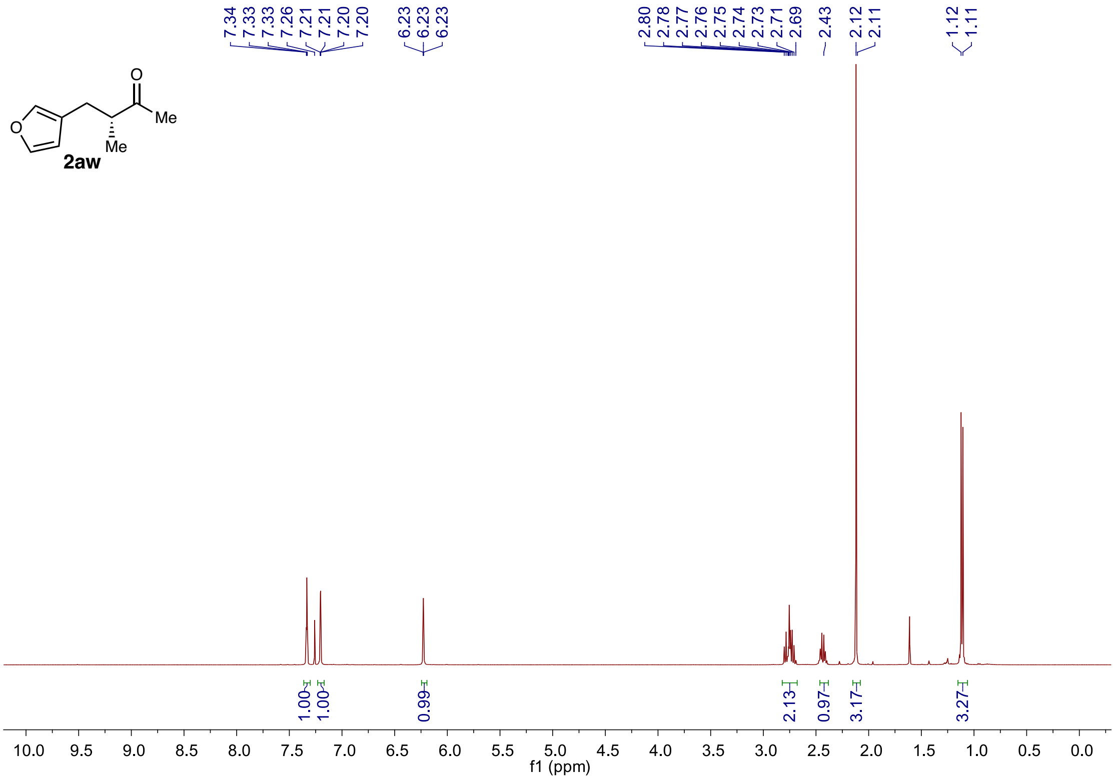

Supplementary Figure 96. ^{1}H NMR (400 MHz, $CDCl_3$) spectrum of **2aw**.

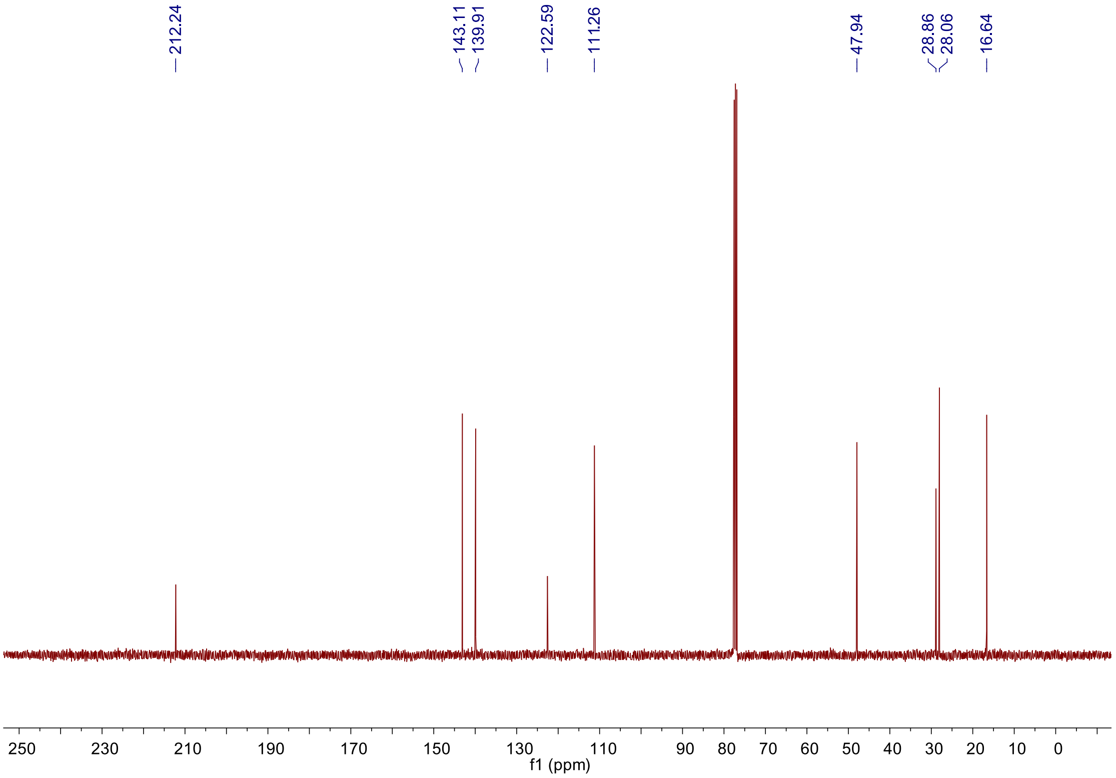

Supplementary Figure 97. ^{13}C NMR (100 MHz, $CDCl_3$) spectrum of **2aw**.

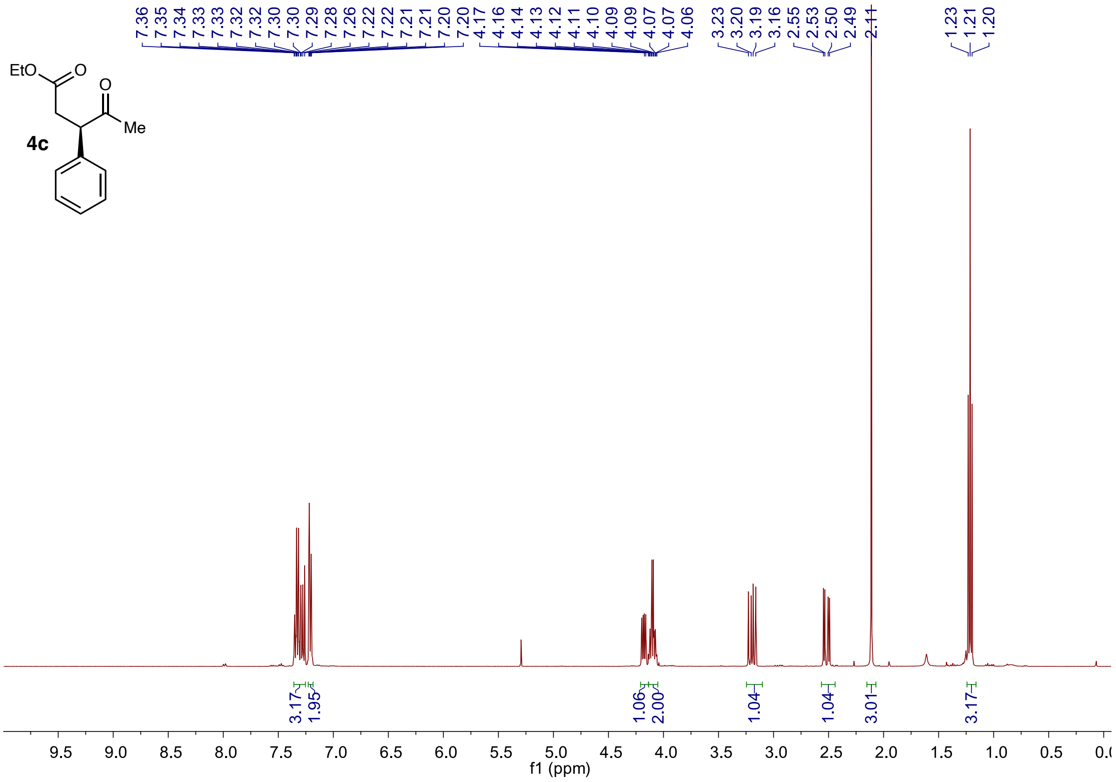

Supplementary Figure 98. ^{1}H NMR (400 MHz, $CDCl_3$) spectrum of **4c**.

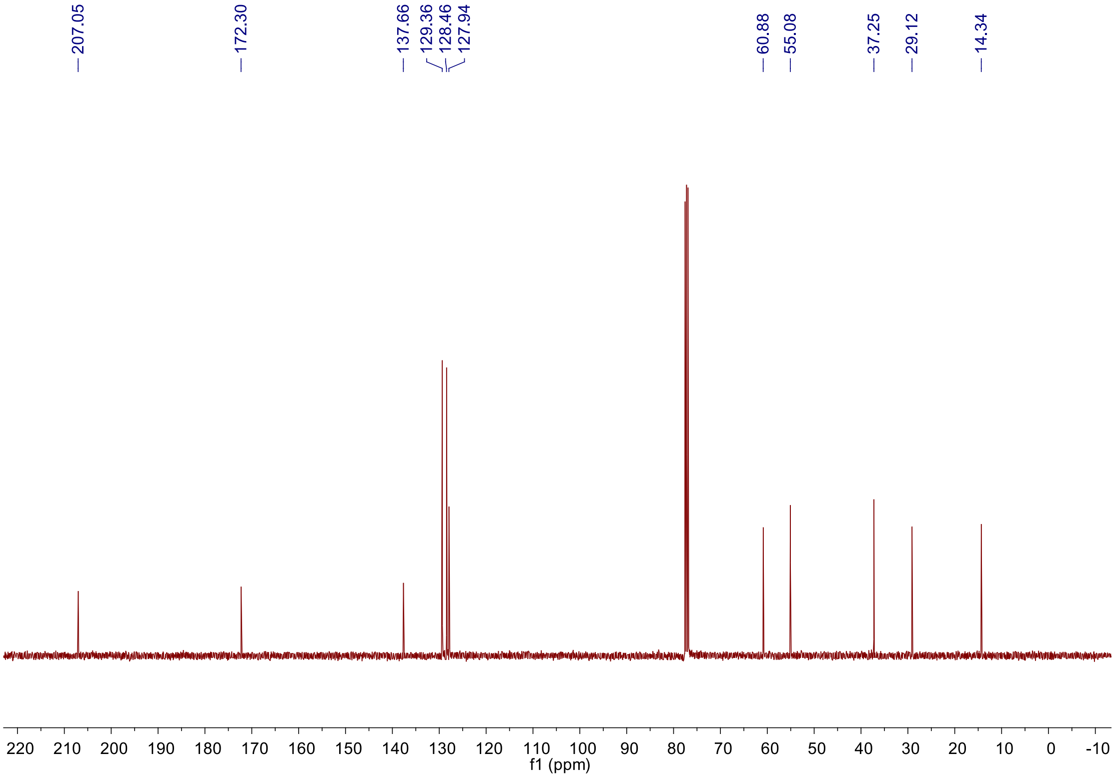

Supplementary Figure 99. ^{13}C NMR (100 MHz, $CDCl_3$) spectrum of **4c**.

Separation of chiral products

Supplementary Table 1. Separation details of chiral products.

Entry	Product	Separation method	Optical rotation	*ee* (%)
1	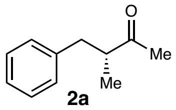 **2a**	GC-MS: Chiraldex β-DM column (50 to 170 °C, 1 °C/min, 1.0 mL/min), t_R = 27.4 min (major) / 30.8 min (minor)	$[\alpha]_D^{26}$ = -37.0 (c = 0.1, $CHCl_3$)	>99
2	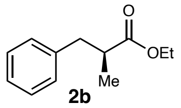 **2b**	GC-MS: Chiraldex β-DM column (50 to 170 °C, 1 °C/min, 1.0 mL/min), t_R = 50.7 min (minor) / 50.3 min (major)	$[\alpha]_D^{26}$ = +25.0 (c = 0.1, $CHCl_3$)	96
3	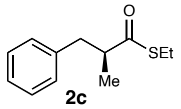 **2c**	SFC: Chiralcel AY-H column (95% CO_2 5% MeOH), 2.0 mL/min, t_R = 3.5 min (minor) / 4.9 min (major)	$[\alpha]_D^{26}$ = -98.0 (c = 0.1, $CHCl_3$)	>99
4	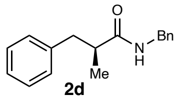 **2d**	SFC: Chiralcel AD-H column (80% CO_2 20% MeOH), 2.0 mL/min, t_R = 7.6 min (major) / 9.5 min (minor)	$[\alpha]_D^{26}$ = +28.0 (c = 0.1, $CHCl_3$)	>99
5	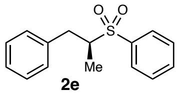 **2e**	SFC: Chiralcel OJ-H column (95% CO_2 5% MeOH), 2.0 mL/min, t_R = 10.4 min (minor) / 11.0 min (major)	$[\alpha]_D^{26}$ = -6.0 (c = 0.1, $CHCl_3$)	92
6	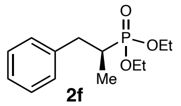 **2f**	SFC: Chiralcel AD-H column (80% CO_2 20% MeOH), 2.0 mL/min, t_R = 3.0 min (major) / 3.3 min (minor)	$[\alpha]_D^{26}$ = +20.0 (c = 0.1, $CHCl_3$)	97
7	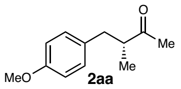 **2aa**	SFC: Chiralcel OJ-H column (95% CO_2 5% MeOH), 2.0 mL/min, t_R = 5.6 min (major) / 6.0 min (minor)	$[\alpha]_D^{26}$ = -34.0 (c = 0.1, $CHCl_3$)	>99
8	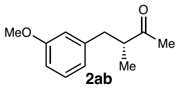 **2ab**	SFC: Chiralpak IC column (95% CO_2 5% MeOH), 2.0 mL/min, t_R = 8.4 min (major) / 9.0 min (minor)	$[\alpha]_D^{26}$ = -25.0 (c = 0.1, $CHCl_3$)	>99
9	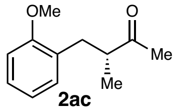 **2ac**	GC-MS: Chiraldex β-DM column (50 to 170 °C, 1 °C/min, 1.0 mL/min), t_R = 63.6 min (major) / 65.0 min (minor)	$[\alpha]_D^{26}$ = -33.0 (c = 0.1, $CHCl_3$)	>99
10	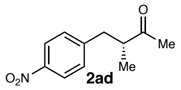 **2ad**	SFC: Chiralpak ID column (95% CO_2 5% MeOH), 2.0 mL/min, t_R = 9.6 min (minor) / 10.1 min (major)	$[\alpha]_D^{26}$ = -6.0 (c = 0.1, $CHCl_3$)	98
11	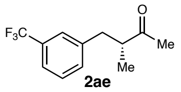 **2ae**	GC-MS: Chiraldex β-DM column (50 to 170 °C, 1 °C/min, 1.0 mL/min), t_R = 44.8 min (major) / 47.0 min (minor)	$[\alpha]_D^{26}$ = -10.0 (c = 0.1, $CHCl_3$)	>99
12	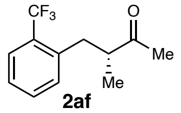 **2af**	GC-MS: Chiraldex β-DM column (50 to 170 °C, 1 °C/min, 1.0 mL/min), t_R = 36.2 min (major) / 37.6 min (minor)	$[\alpha]_D^{26}$ = -26.0 (c = 0.1, $CHCl_3$)	99

13	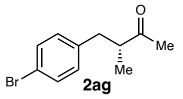 **2ag**	GC-MS: Chiraldex β-DM column (50 to 170 °C, 1 °C/min, 1.0 mL/min), t_R = 78.6 min (major) / 90.0 min (minor)	$[\alpha]_D^{26}$ = -20.0 (c = 0.1, $CHCl_3$)	>99
14	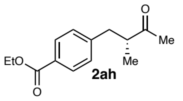 **2ah**	SFC: Chiralcel AD-H column (90% CO_2 10% MeOH), 2.0 mL/min, t_R = 9.0 min (major) / 9.7 min (minor)	$[\alpha]_D^{26}$ = +17.0 (c = 0.1, $CHCl_3$)	99
15	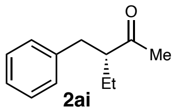 **2ai**	SFC: Chiralpak IC column (95% CO_2 5% MeOH), 2.0 mL/min, t_R = 5.0 min (major) / 5.2 min (minor)	$[\alpha]_D^{26}$ = -37.0 (c = 0.1, $CHCl_3$)	>99
16	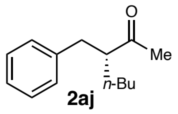 **2aj**	SFC: Chiralpak IC column (95% CO_2 5% MeOH), 2.0 mL/min, t_R = 5.0 min (minor) / 5.3 min (major)	$[\alpha]_D^{26}$ = -17.0 (c = 0.1, $CHCl_3$)	>99
17	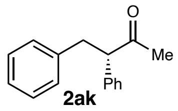 **2ak**	GC-MS: Chiraldex β-DM column (50 to 170 °C, 1 °C/min, 1.0 mL/min), t_R = 47.3 min (major) / 52.7 min (minor)	$[\alpha]_D^{26}$ = -369.0 (c = 0.1, $CHCl_3$)	93
18	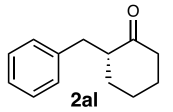 **2al**	GC-MS: Chiraldex β-DM column (50 to 170 °C, 1 °C/min, 1.0 mL/min), t_R = 90.2 min (major) / 91.4 min (minor)	$[\alpha]_D^{26}$ = -38.0 (c = 0.1, $CHCl_3$)	>99
19	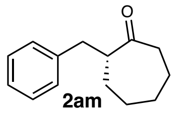 **2am**	SFC: Chiralpak IC column (95% CO_2 5% MeOH), 2.0 mL/min, t_R = 13.4 min (major) / 15.4 min (minor)	$[\alpha]_D^{26}$ = +100.0 (c = 0.1, $CHCl_3$)	99
20	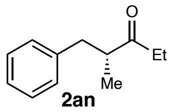 **2an**	SFC: Chiralcel OJ-H column (95% CO_2 5% MeOH), 2.0 mL/min, t_R = 3.9 min (major) / 4.1 min (minor)	$[\alpha]_D^{26}$ = -42.0 (c = 0.1, $CHCl_3$)	98
21	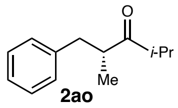 **2ao**	SFC: Chiralpak IF column (95% CO_2 5% MeOH), 2.0 mL/min, t_R = 4.6 min (minor) / 5.0 min (major)	$[\alpha]_D^{26}$ = -82.0 (c = 0.1, $CHCl_3$)	>99
22	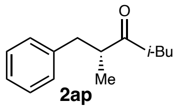 **2ap**	SFC: Chiralcel OJ-H column (95% CO_2 5% MeOH), 2.0 mL/min, t_R = 3.3 min (major) / 3.4 min (minor)	$[\alpha]_D^{26}$ = -47.0 (c = 0.1, $CHCl_3$)	>99
23	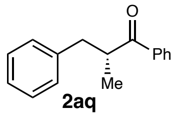 **2aq**	SFC: Chiralcel AS-H column (95% CO_2 5% MeOH), 2.0 mL/min, t_R = 5.2 min (major) / 5.5 min (minor)	$[\alpha]_D^{26}$ = -74.0 (c = 0.1, $CHCl_3$)	>99
24	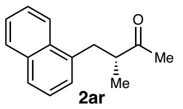 **2ar**	SFC: Chiralpak IC column (95% CO_2 5% MeOH), 2.0 mL/min, t_R = 11.8 min (minor) / 12.8 min (major)	$[\alpha]_D^{26}$ = +49.0 (c = 0.1, $CHCl_3$)	>99
25	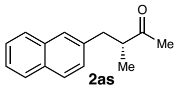 **2as**	GC-MS: Chiraldex β-DM column (50 to 170 °C, 1 °C/min, 1.0 mL/min), t_R = 100.0 min (major) / 100.9 min (minor)	$[\alpha]_D^{26}$ = +38.0 (c = 0.1, $CHCl_3$)	>99
26	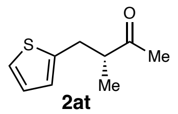 **2at**	GC-MS: Chiraldex β-DM column (50 to 170 °C, 1 °C/min, 1.0 mL/min), t_R = 44.3 min (major) / 46.2 min (minor)	$[\alpha]_D^{26}$ = +15.0 (c = 0.1, $CHCl_3$)	99

27	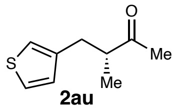 2au	GC-MS: Chiraldex β-DM column (50 to 170 °C, 1 °C/min, 1.0 mL/min), t_R = 50.4 min (major) / 52.7 min (minor)	$[\alpha]_D^{26}$ = +21.0 (c = 0.1, $CHCl_3$)	99
28	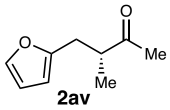 2av	GC-MS: Chiraldex β-DM column (50 to 170 °C, 1 °C/min, 1.0 mL/min), t_R = 25.9 min (major) / 27.5 min (minor)	$[\alpha]_D^{26}$ = +25.0 (c = 0.1, $CHCl_3$)	99
29	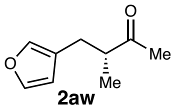 2aw	GC-MS: Chiraldex β-DM column (50 to 170 °C, 1 °C/min, 1.0 mL/min), t_R = 29.3 min (major) / 31.4 min (minor)	$[\alpha]_D^{26}$ = +22 (c = 0.1, $CHCl_3$)	97
30	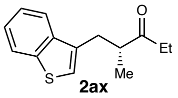 2ax	SFC: Chiralcel AS-H column (95% CO_2 5% MeOH), 2.0 mL/min, t_R = 4.6 min (minor) / 5.2 min (major)	$[\alpha]_D^{26}$ = -34.0 (c = 0.1, $CHCl_3$)	>99
31	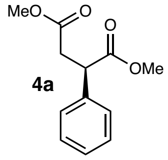 4a	SFC: Chiralcel OZ-H column (90% CO_2 10% MeOH), 2.0 mL/min, t_R = 3.7 min (minor) / 4.1 min (major)	$[\alpha]_D^{26}$ = -74.0 (c = 0.1, $CHCl_3$)	96
32	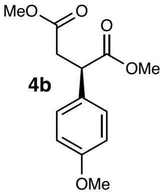 4b	SFC: Chiralcel AY-H column (90% CO_2 10% MeOH), 2.0 mL/min, t_R = 4.6 min (major) / 5.1 min (minor)	$[\alpha]_D^{26}$ = -86.0 (c = 0.1, $CHCl_3$)	99
33	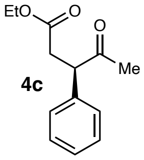 4c	SFC: Chiralcel OZ-H column (95% CO_2 5% MeOH), 2.0 mL/min, t_R = 5.7 min (minor) / 7.3 min (major)	$[\alpha]_D^{26}$ = -331.0 (c = 0.1, $CHCl_3$)	97

Chromatograms

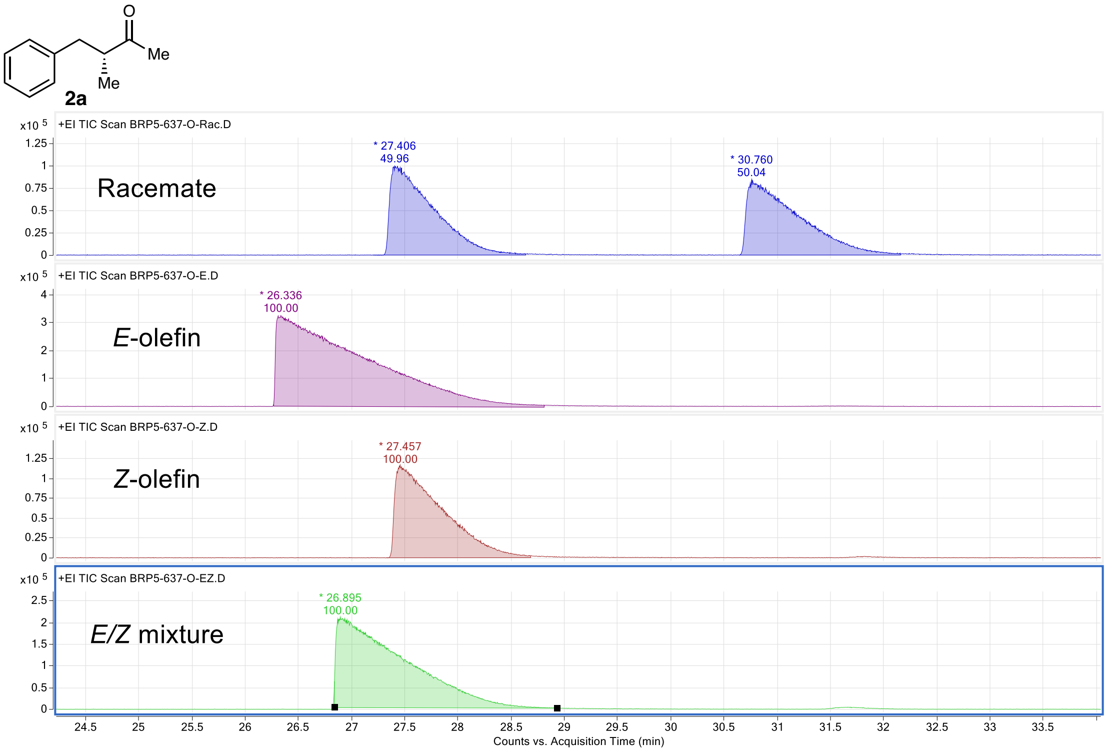

Supplementary Figure 100. GC spectra of **2a**.

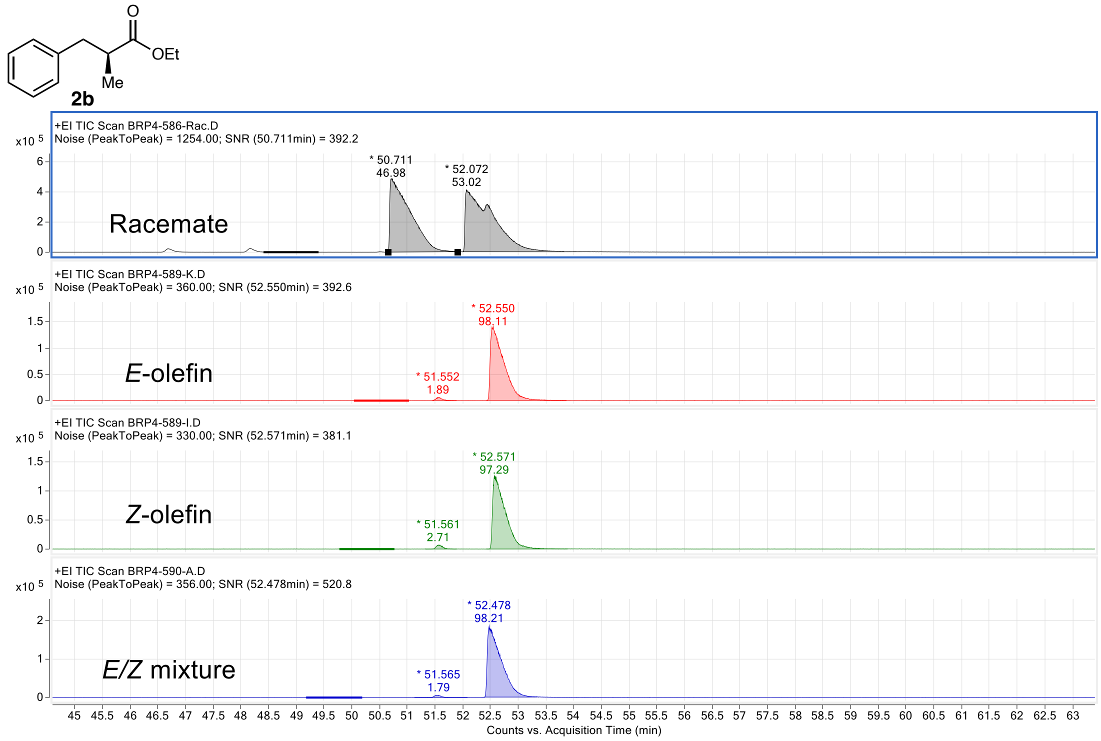

Supplementary Figure 101. GC spectra of **2b**.

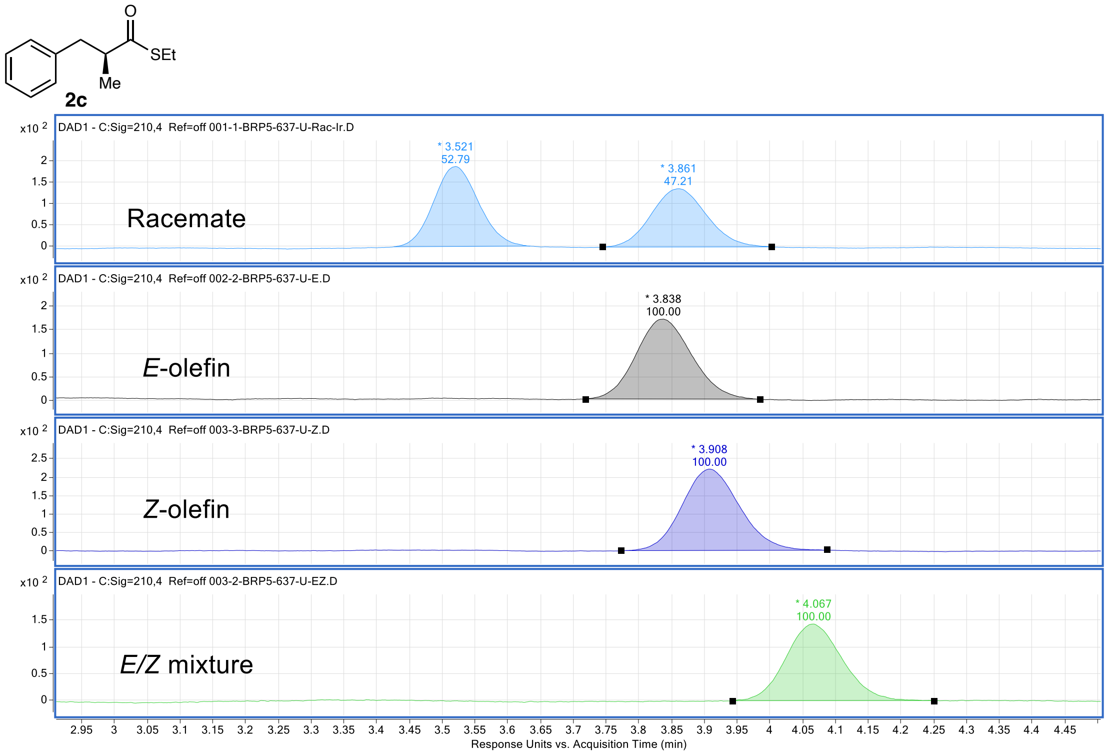

Supplementary Figure 102. SFC spectra of **2c**.

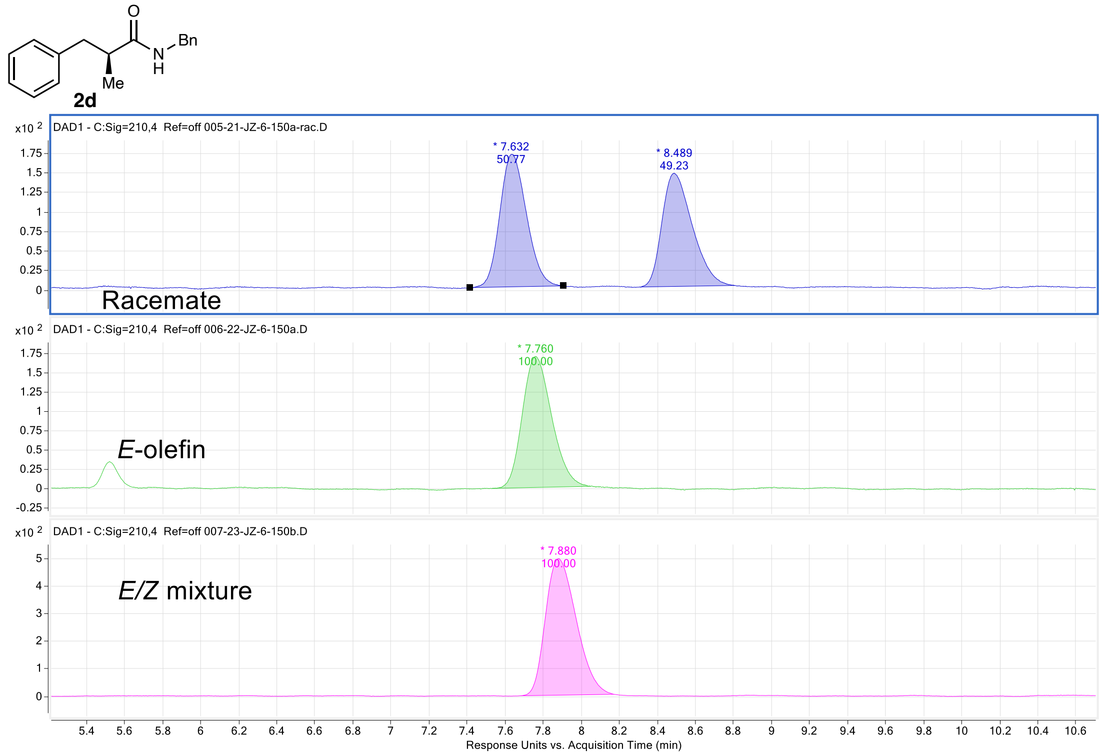

Supplementary Figure 103. SFC spectra of **2d**.

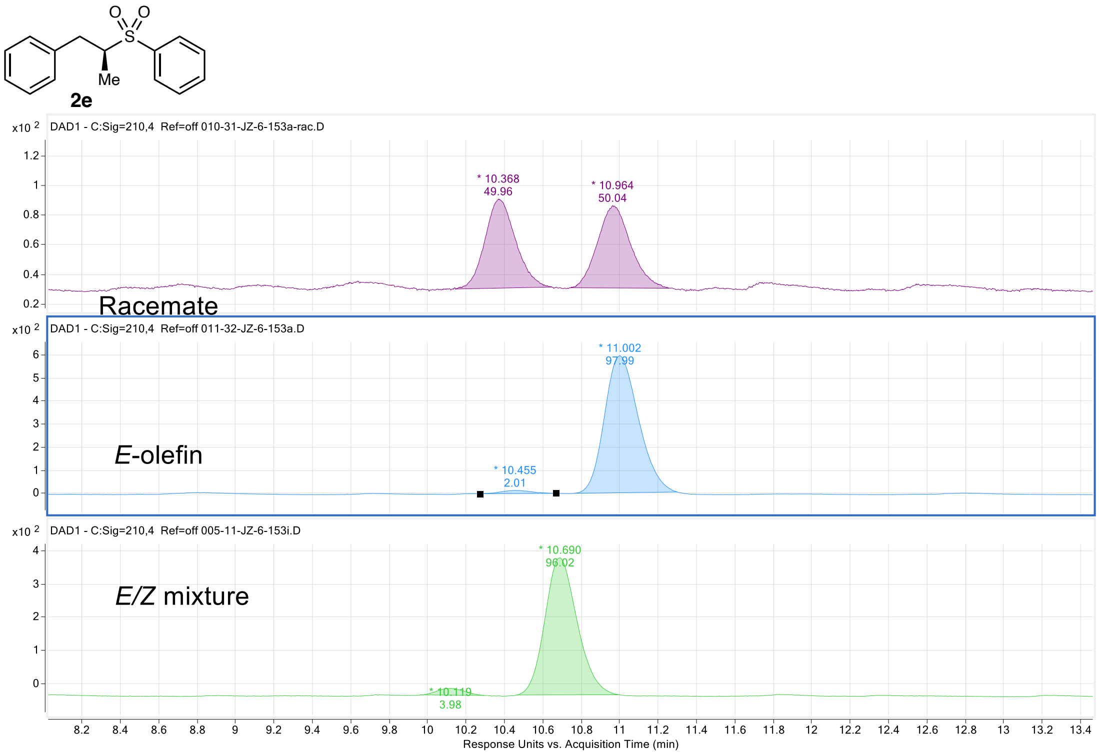

Supplementary Figure 104. SFC spectra of **2e**.

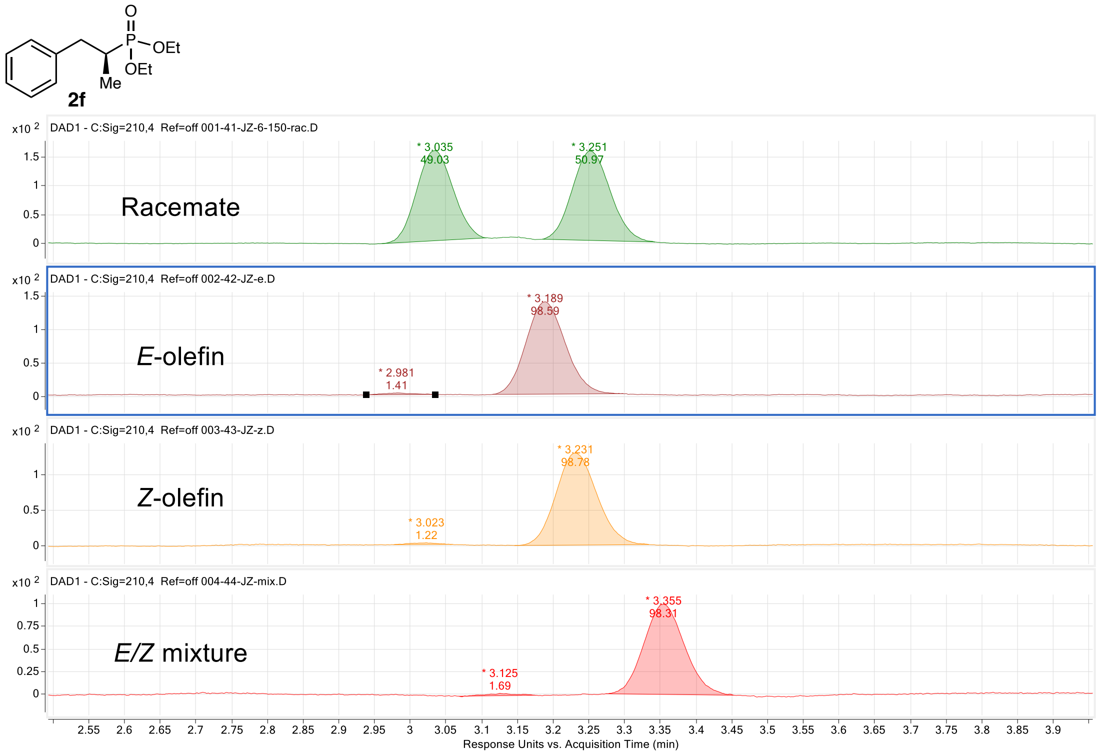

Supplementary Figure 105. SFC spectra of **2f**.

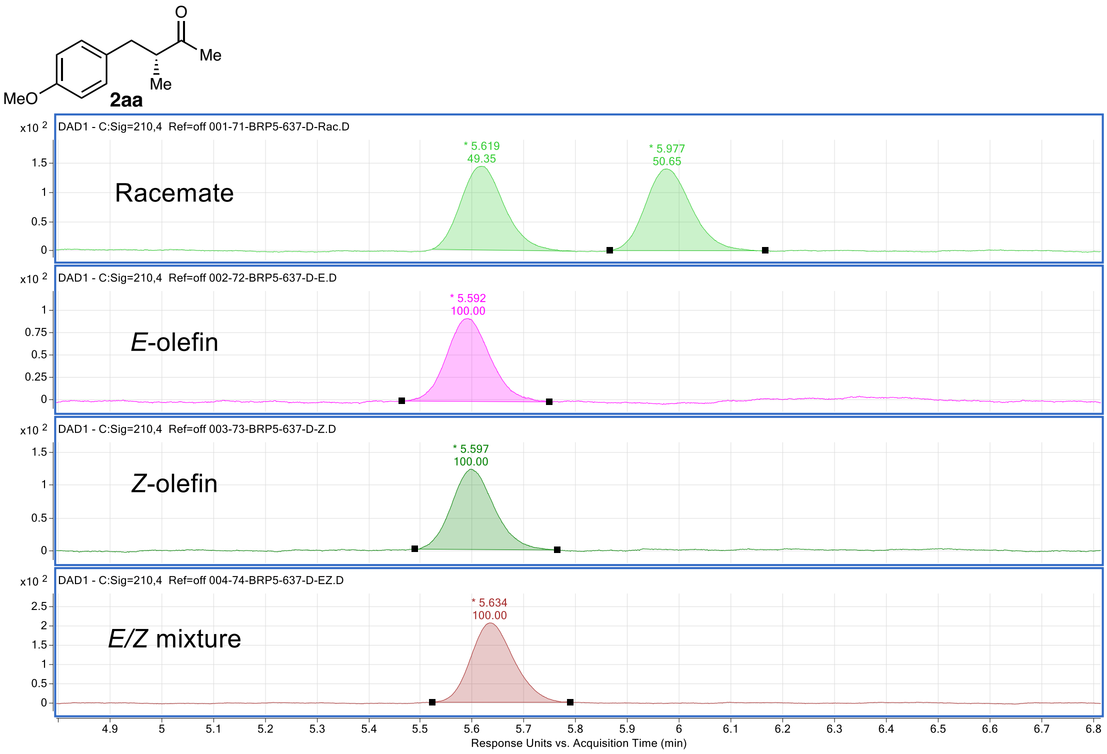

Supplementary Figure 106. SFC spectra of **2aa**.

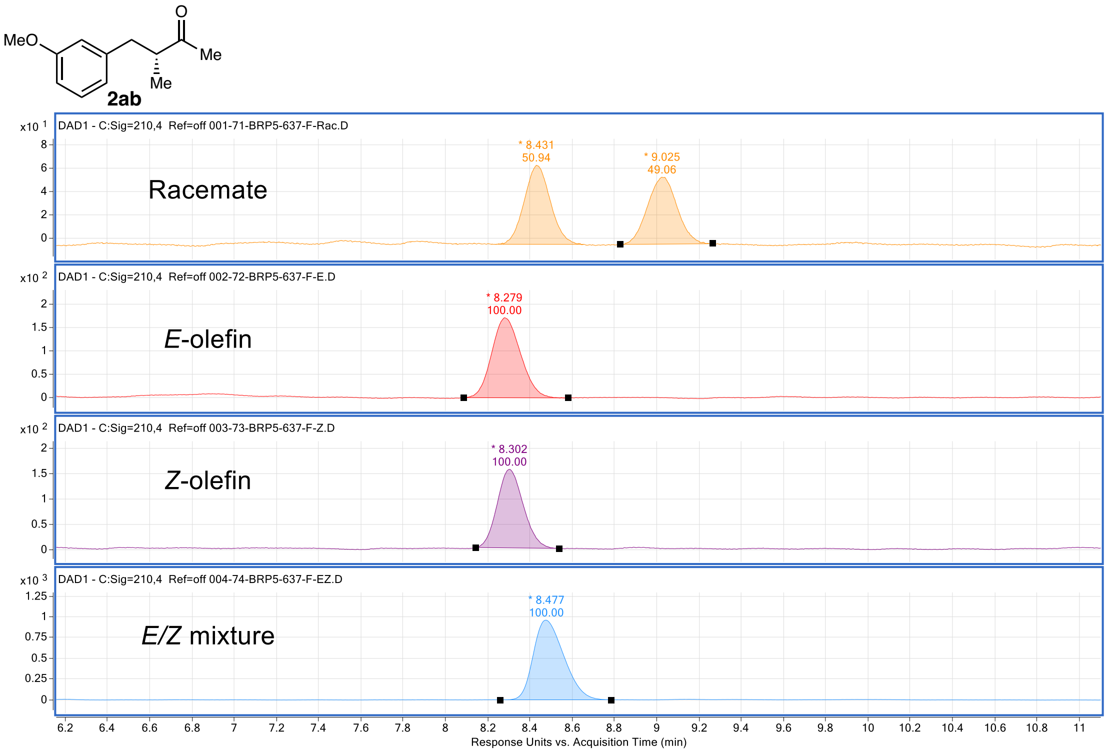

Supplementary Figure 107. SFC spectra of **2ab**.

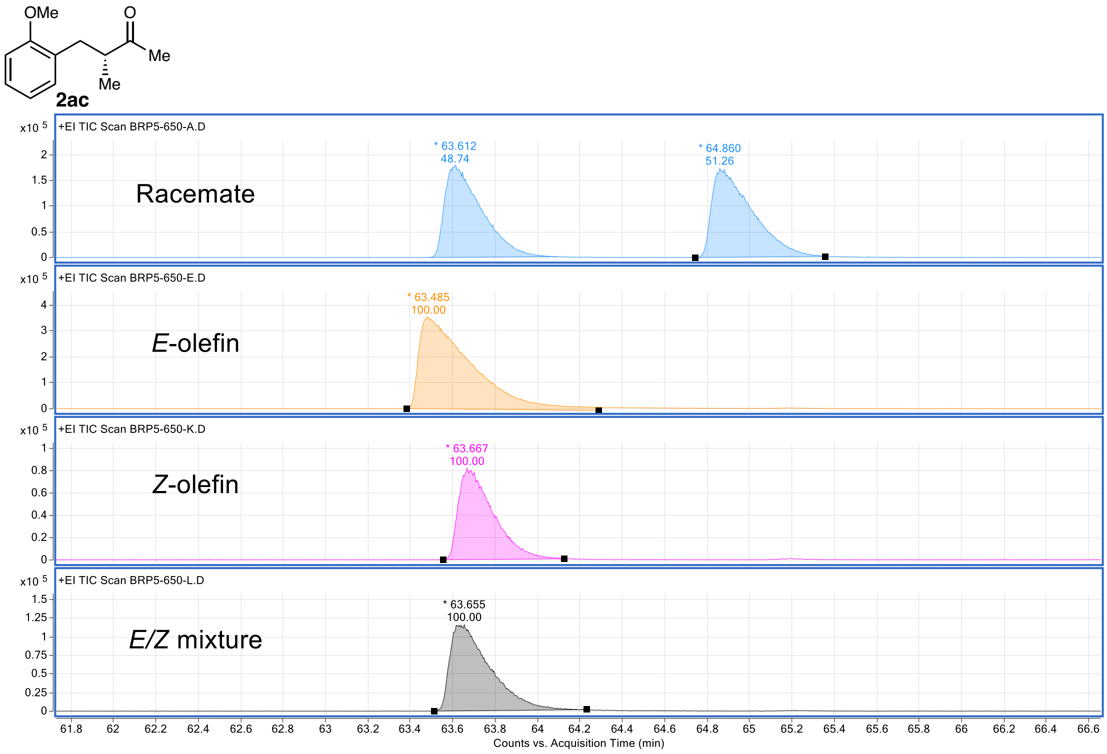

Supplementary Figure 108. GC spectra of **2ac**.

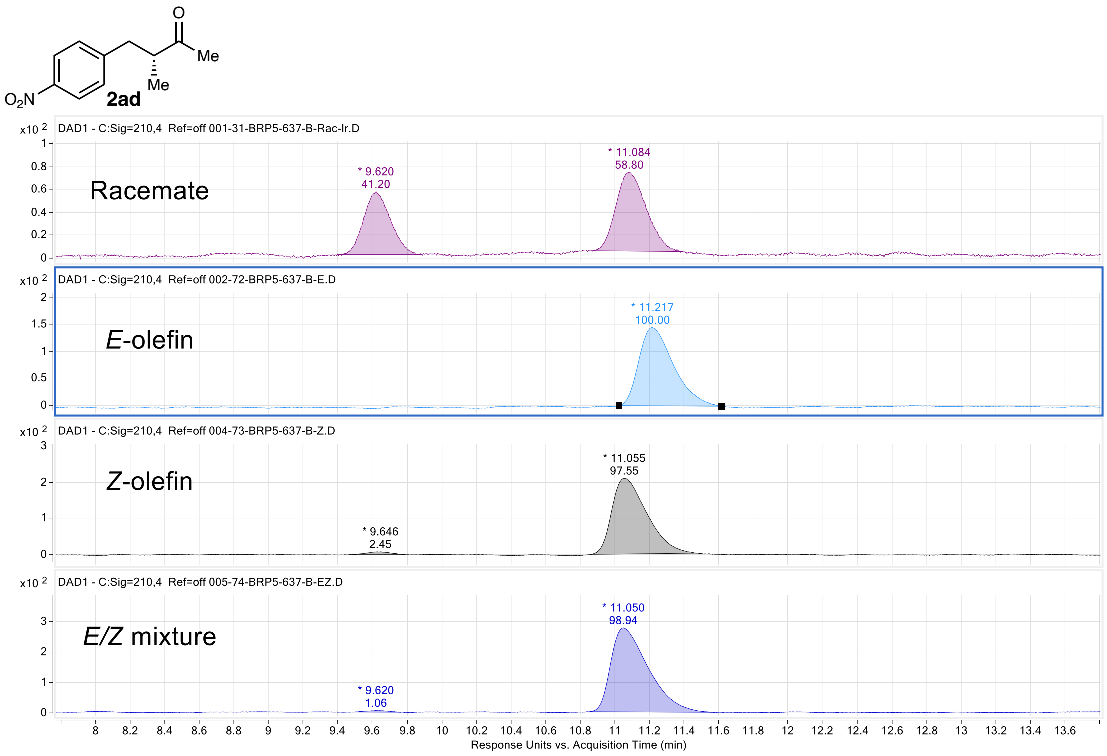

Supplementary Figure 109. SFC spectra of **2ad**.

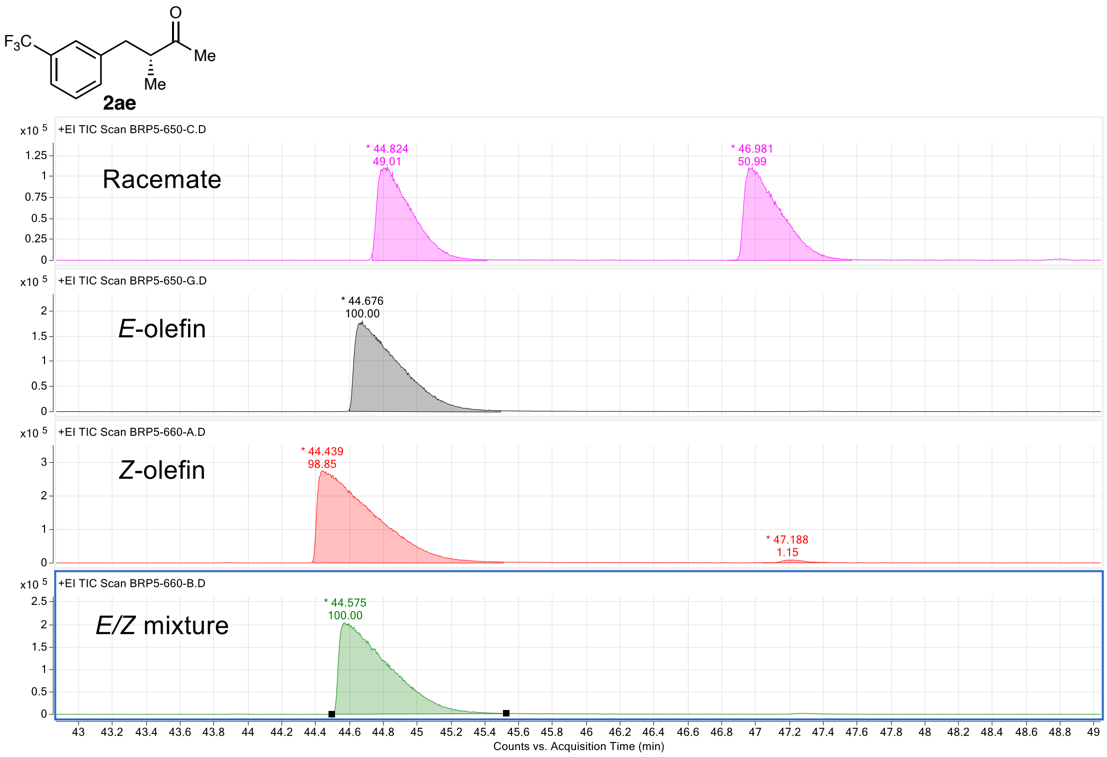

Supplementary Figure 110. GC spectra of **2ae**.

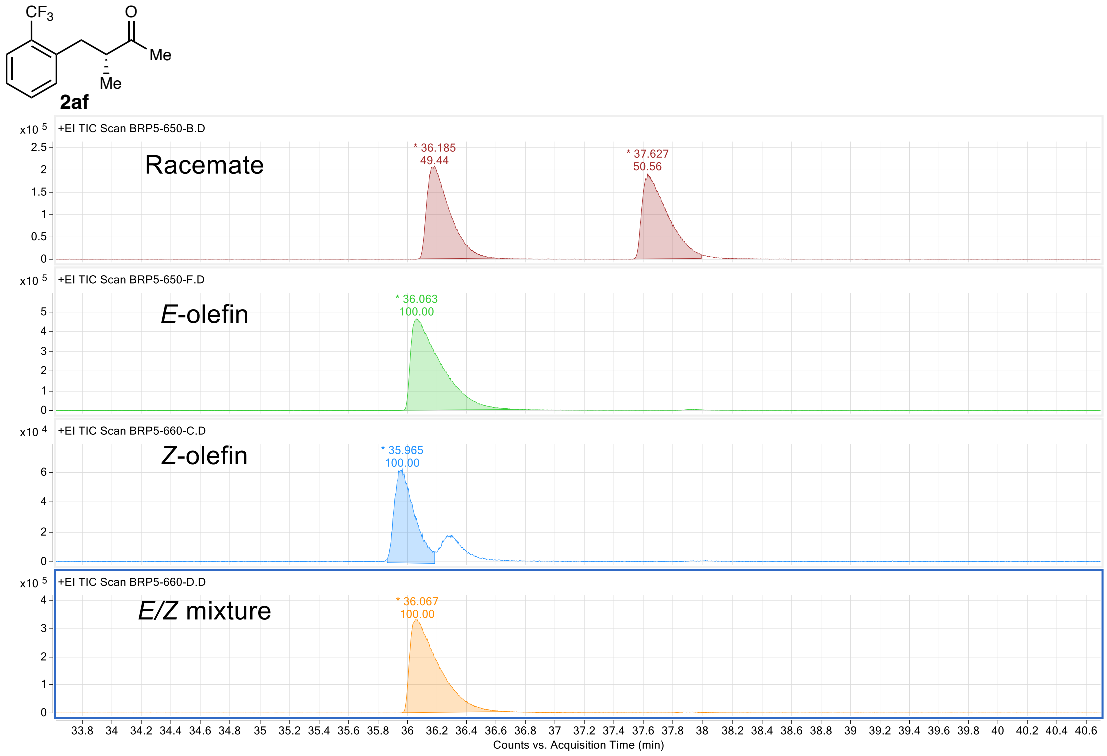

Supplementary Figure 111. GC spectra of **2af**.

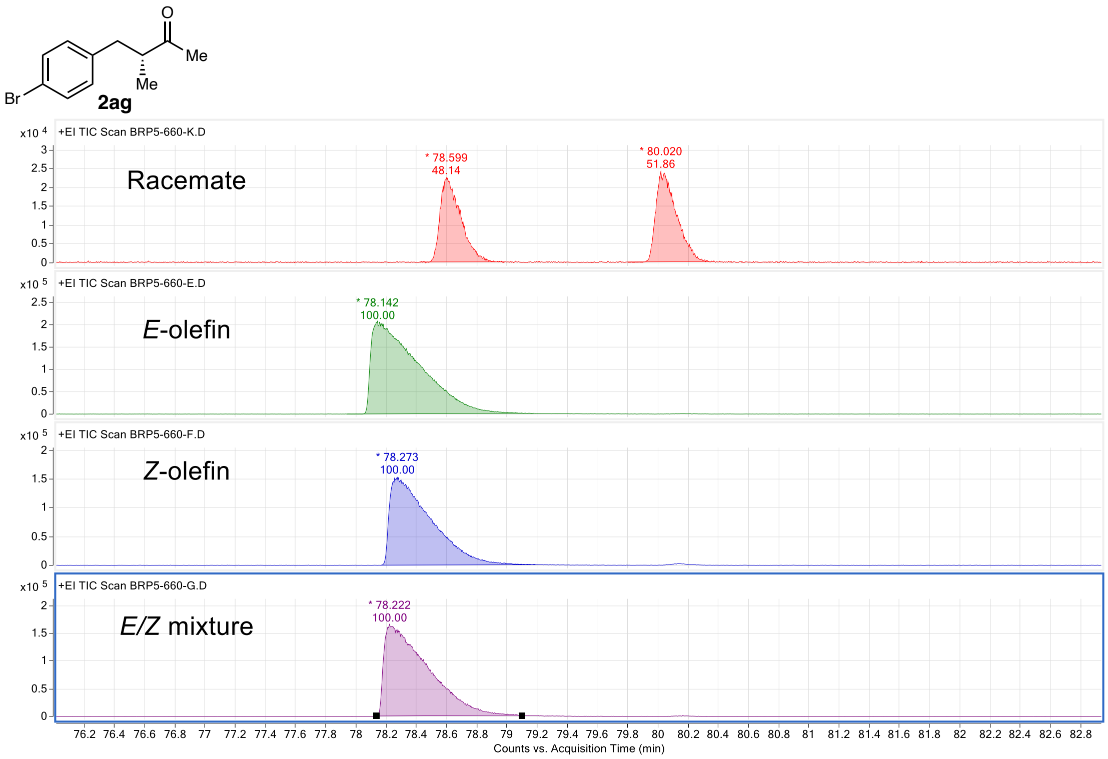

Supplementary Figure 112. GC spectra of **2ag**.

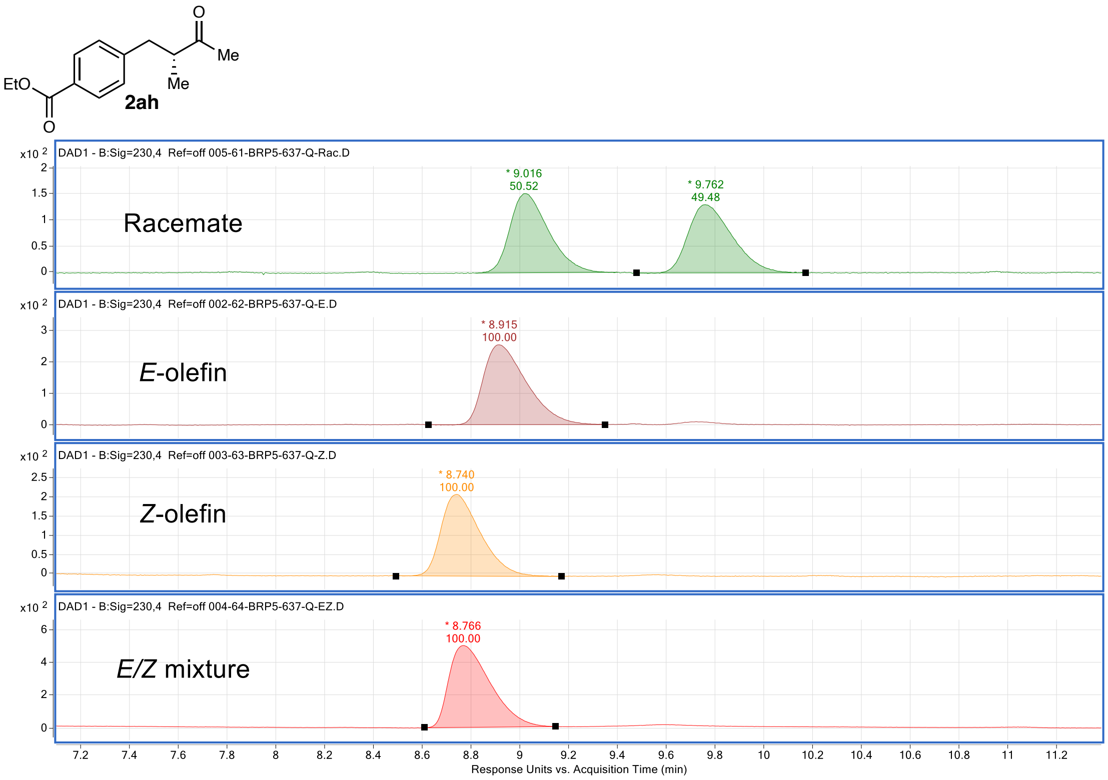

Supplementary Figure 113. SFC spectra of **2ah**.

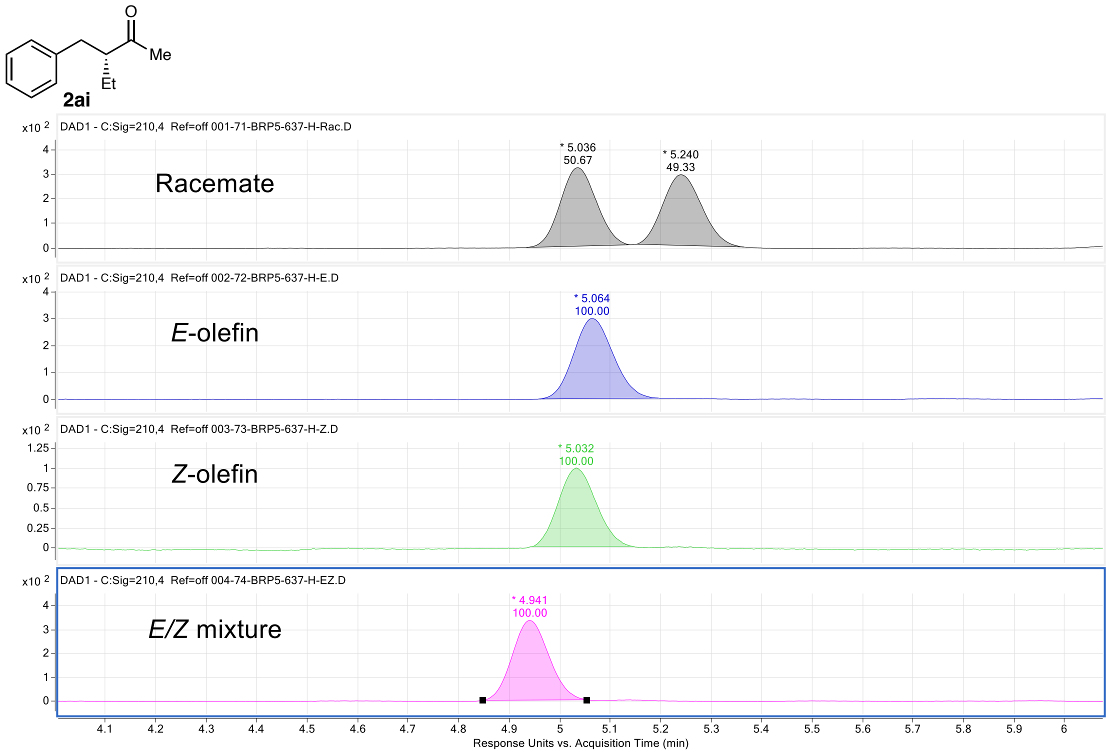

Supplementary Figure 114. SFC spectra of **2ai**.

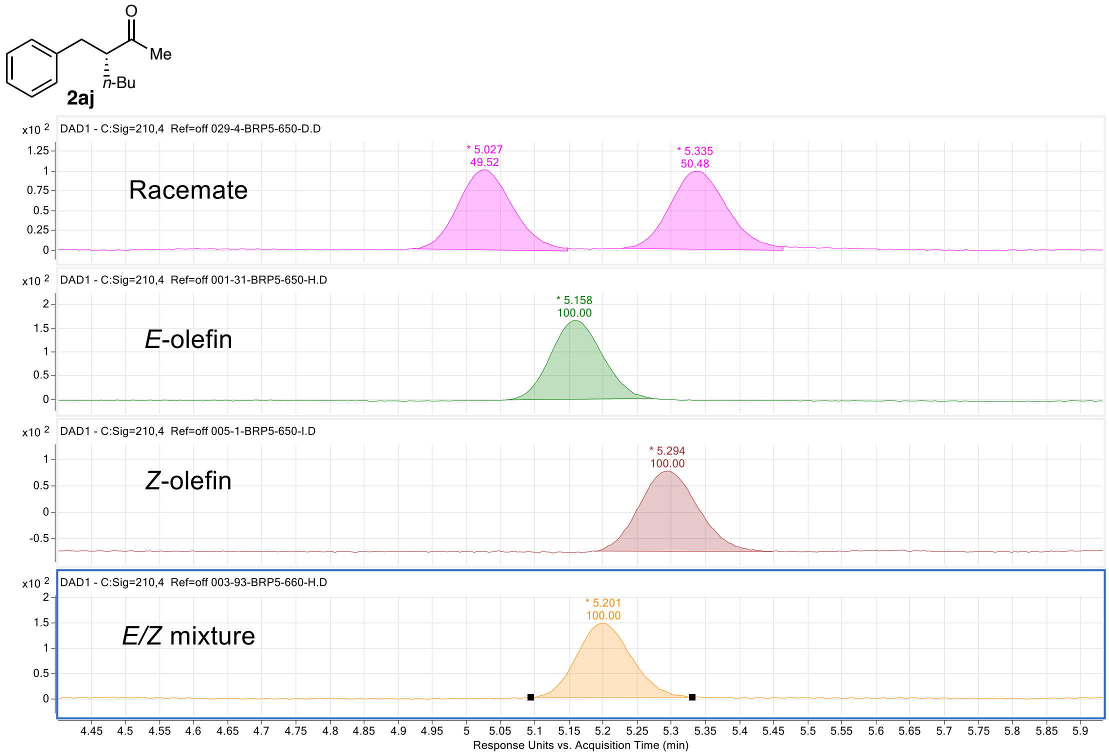

Supplementary Figure 115. SFC spectra of **2aj**.

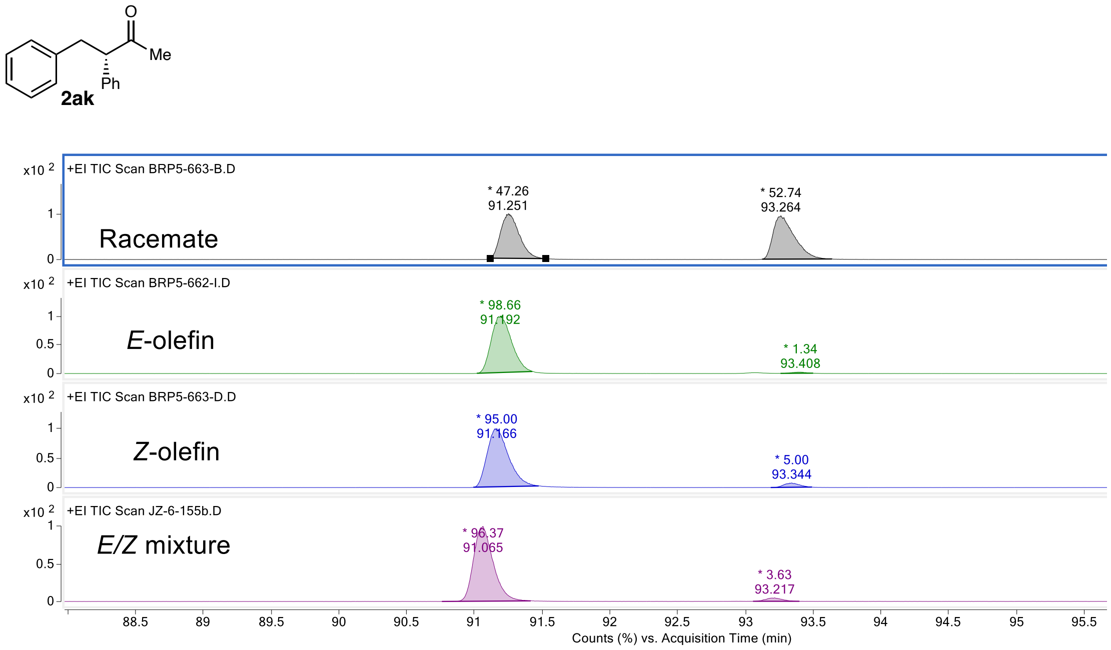

Supplementary Figure 116. GC spectra of **2ak**.

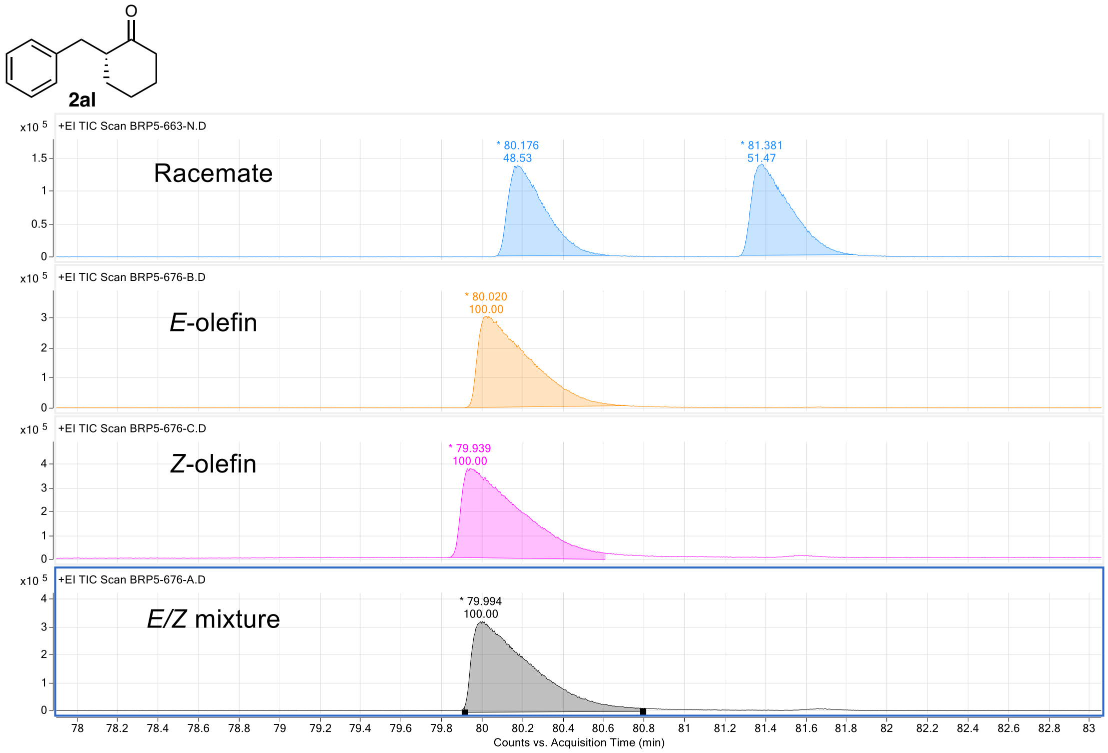

Supplementary Figure 117. GC spectra of **2al**.

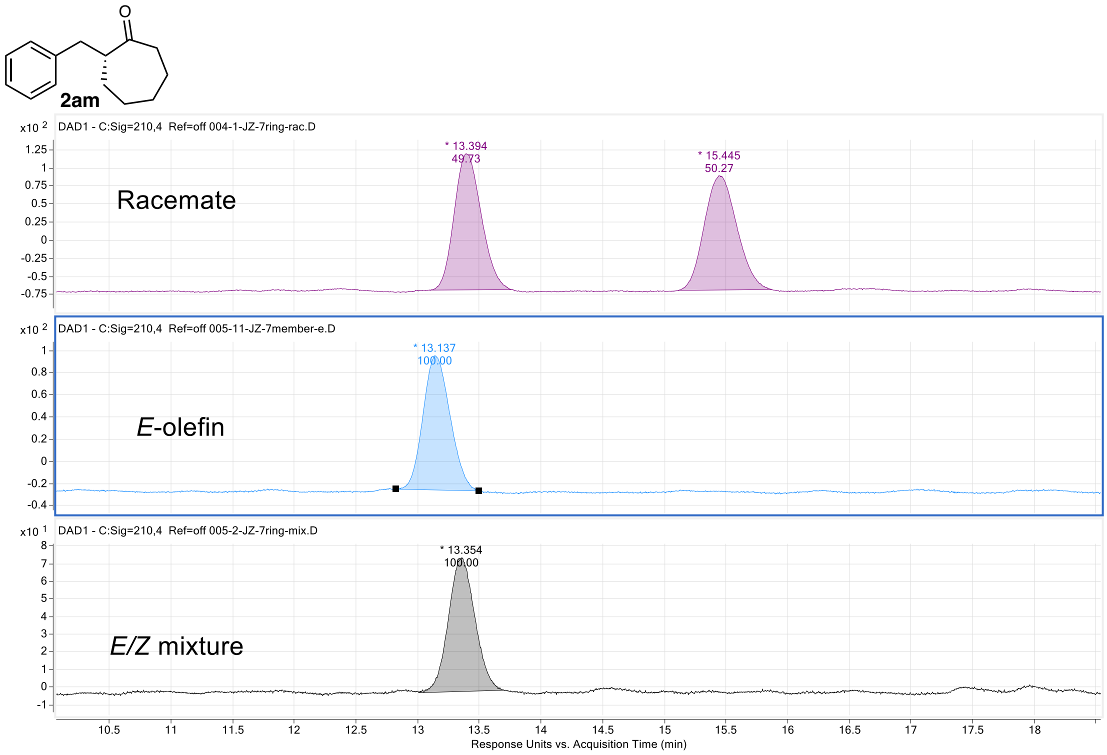

Supplementary Figure 118. SFC spectra of **2am**.

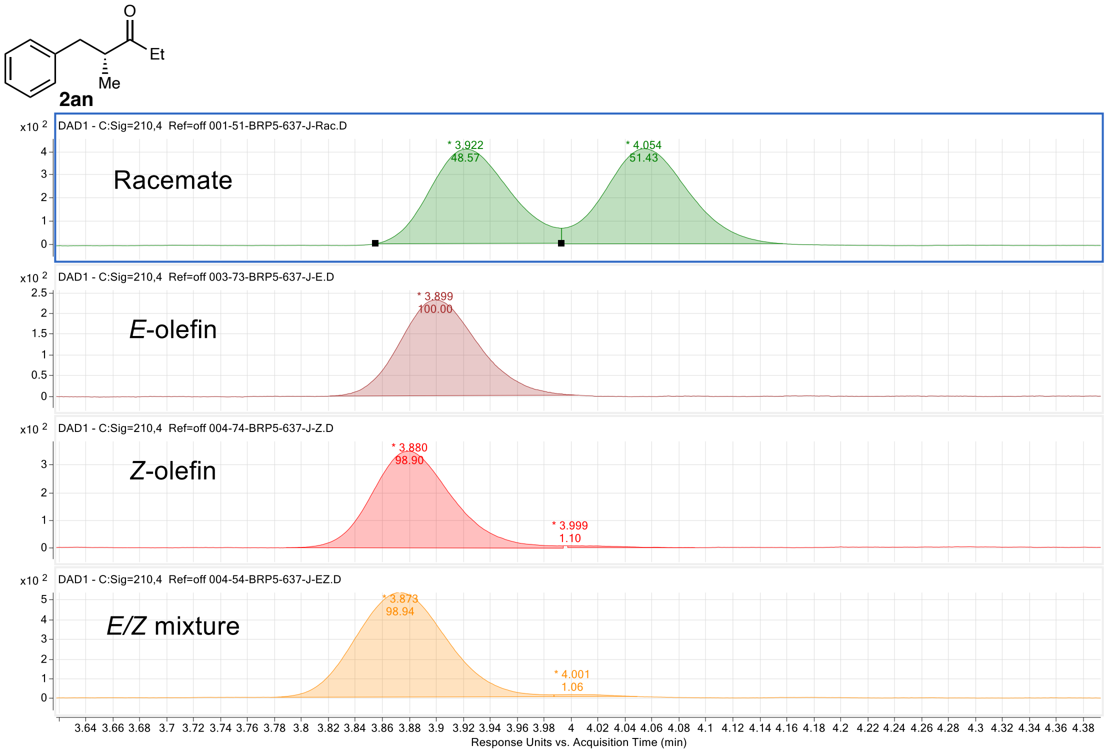

Supplementary Figure 119. SFC spectra of **2an**.

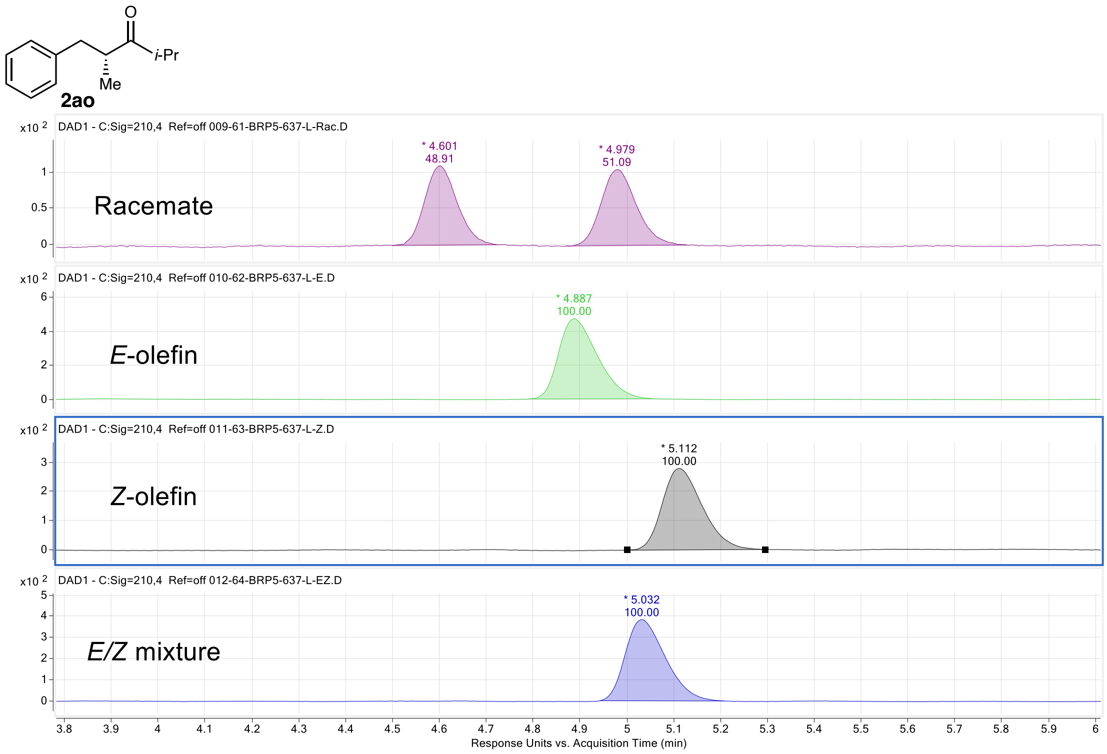

Supplementary Figure 120. SFC spectra of **2ao**.

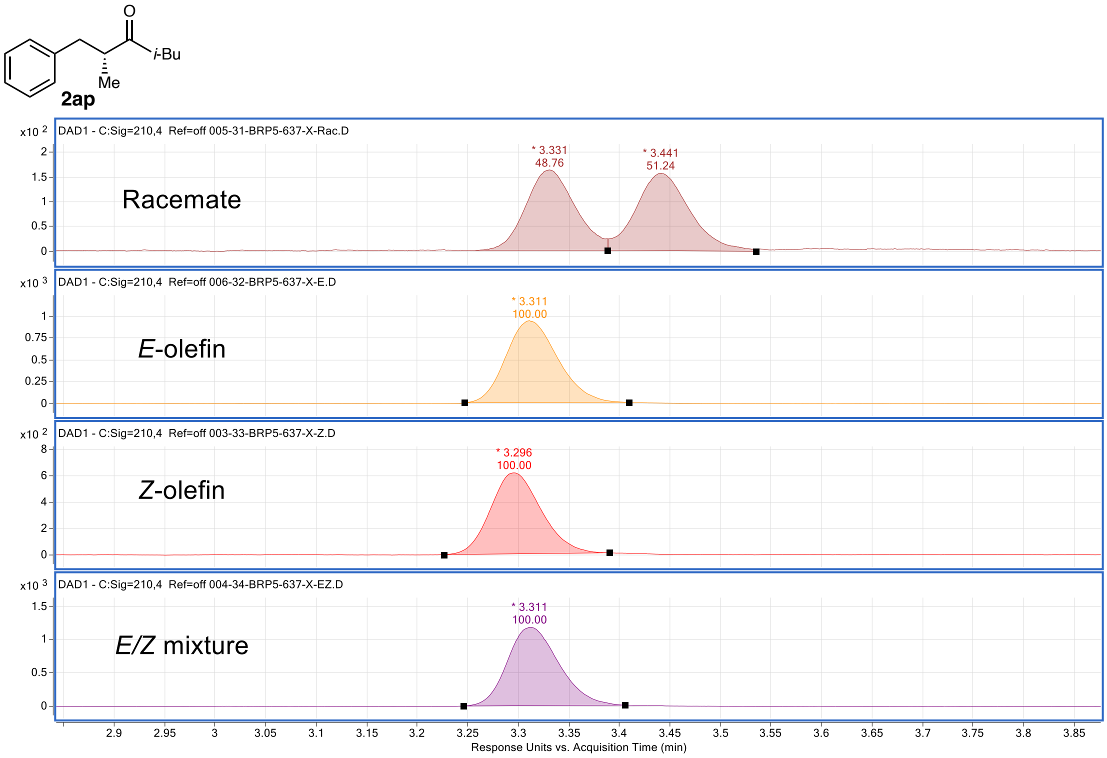

Supplementary Figure 121. SFC spectra of **2ap**.

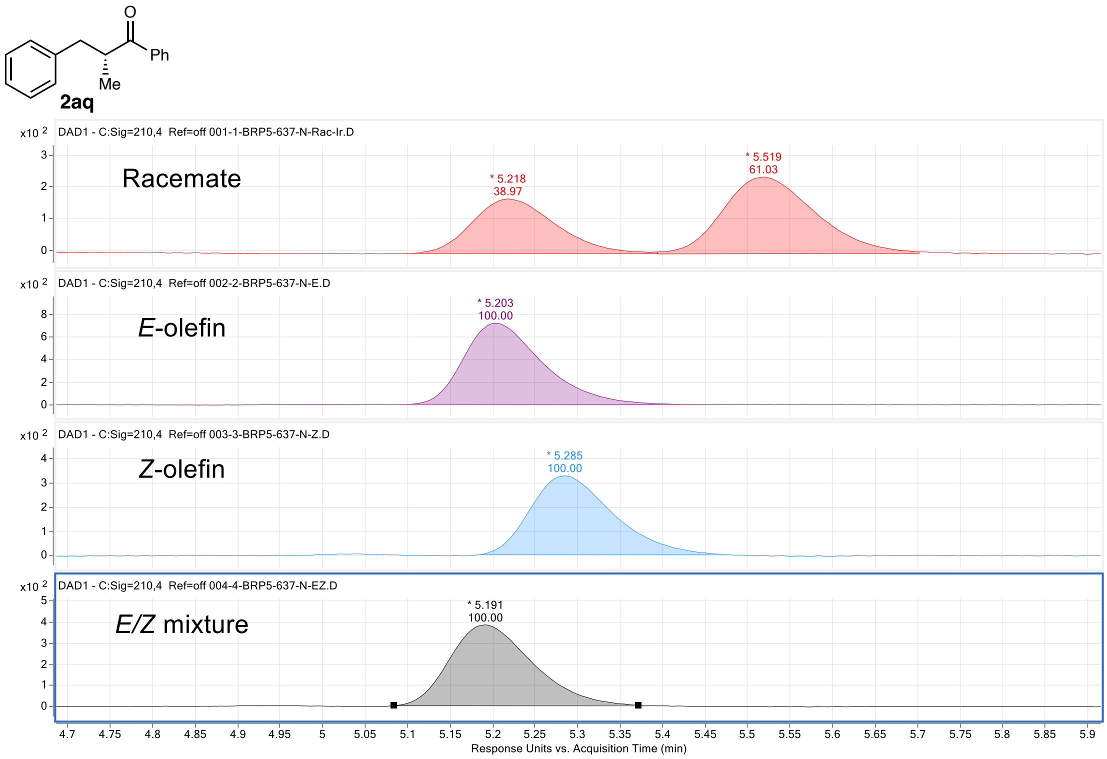

Supplementary Figure 122. SFC spectra of **2aq**.

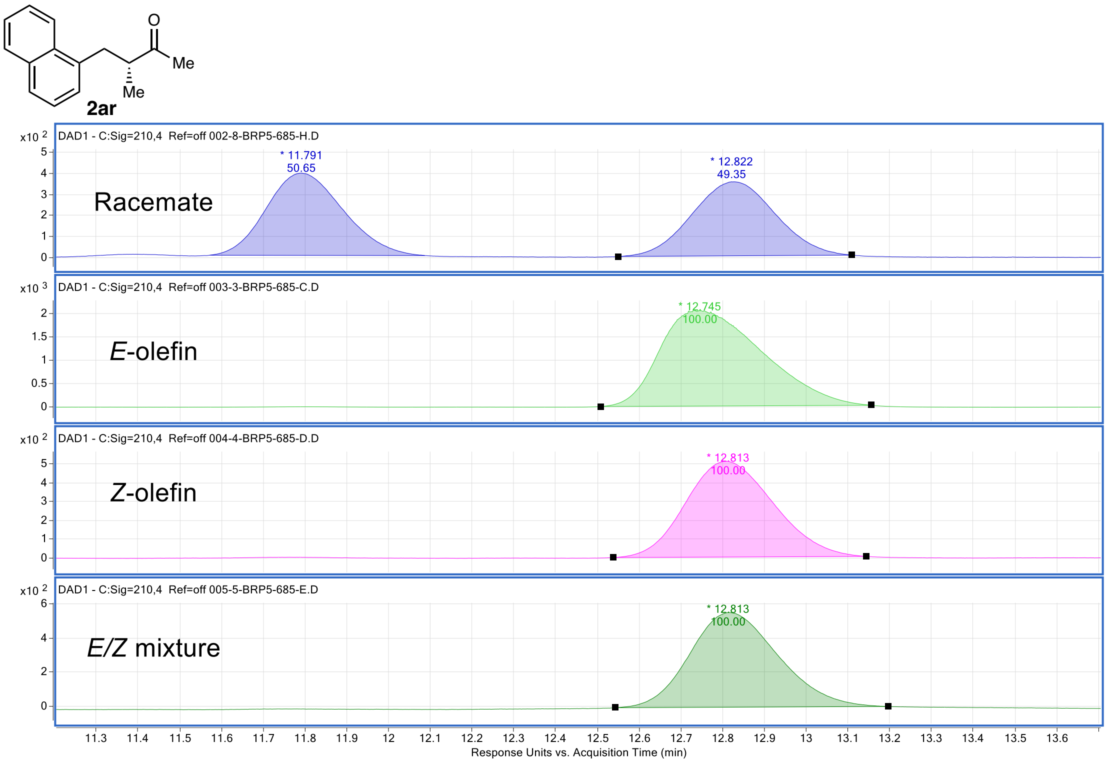

Supplementary Figure 123. SFC spectra of **2ar**.

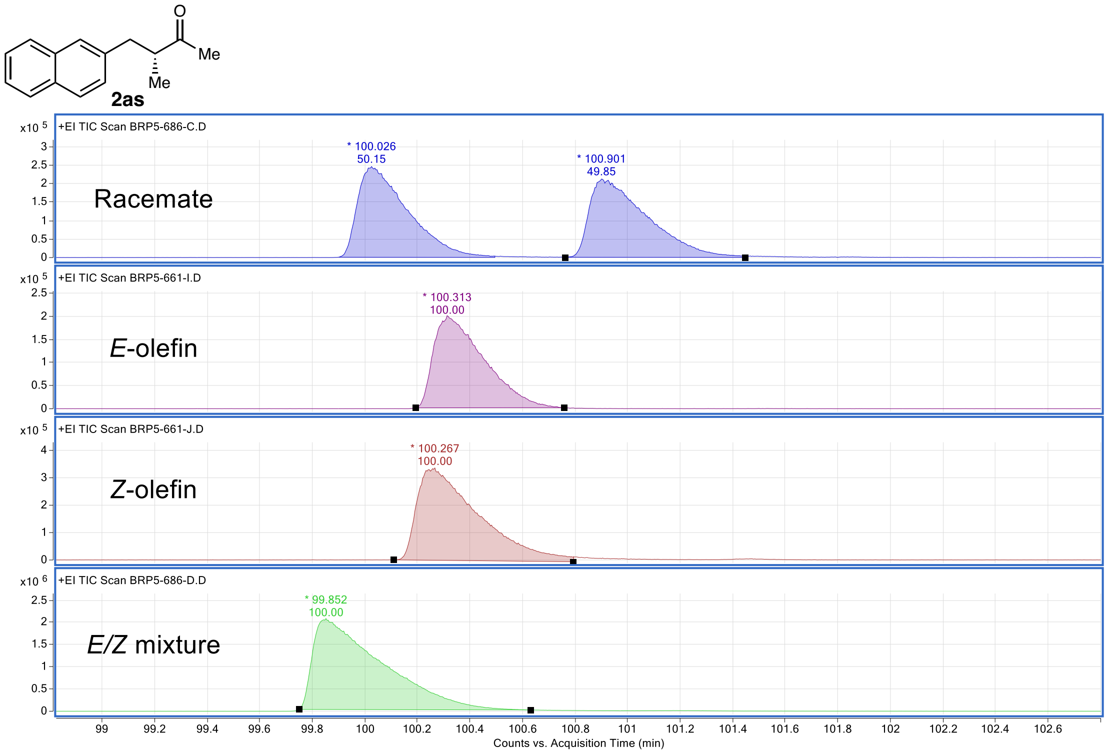

Supplementary Figure 124. GC spectra of **2as**.

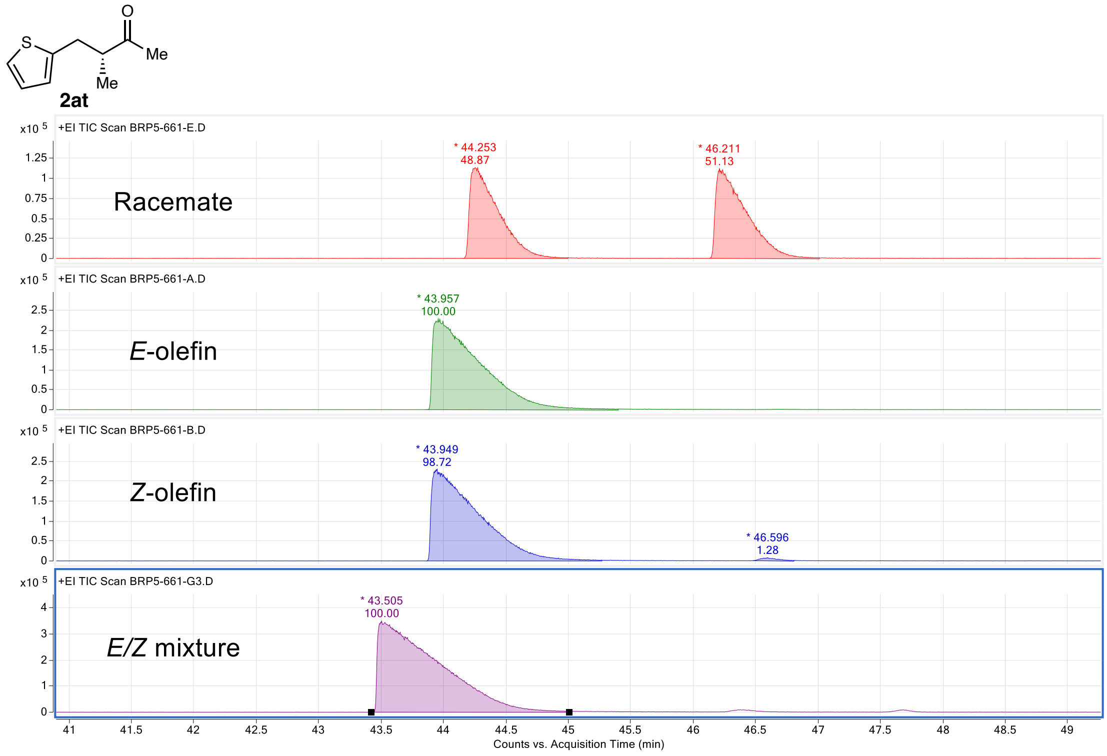

Supplementary Figure 125. GC spectra of **2at**.

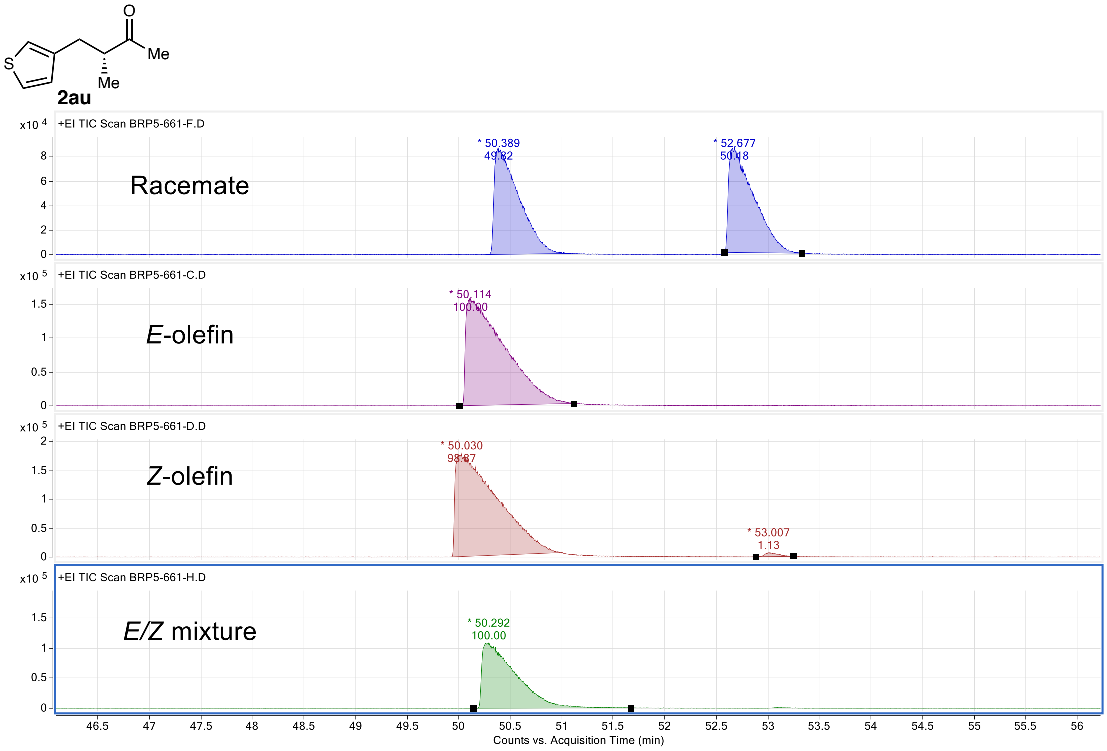

Supplementary Figure 126. GC spectra of **2au**.

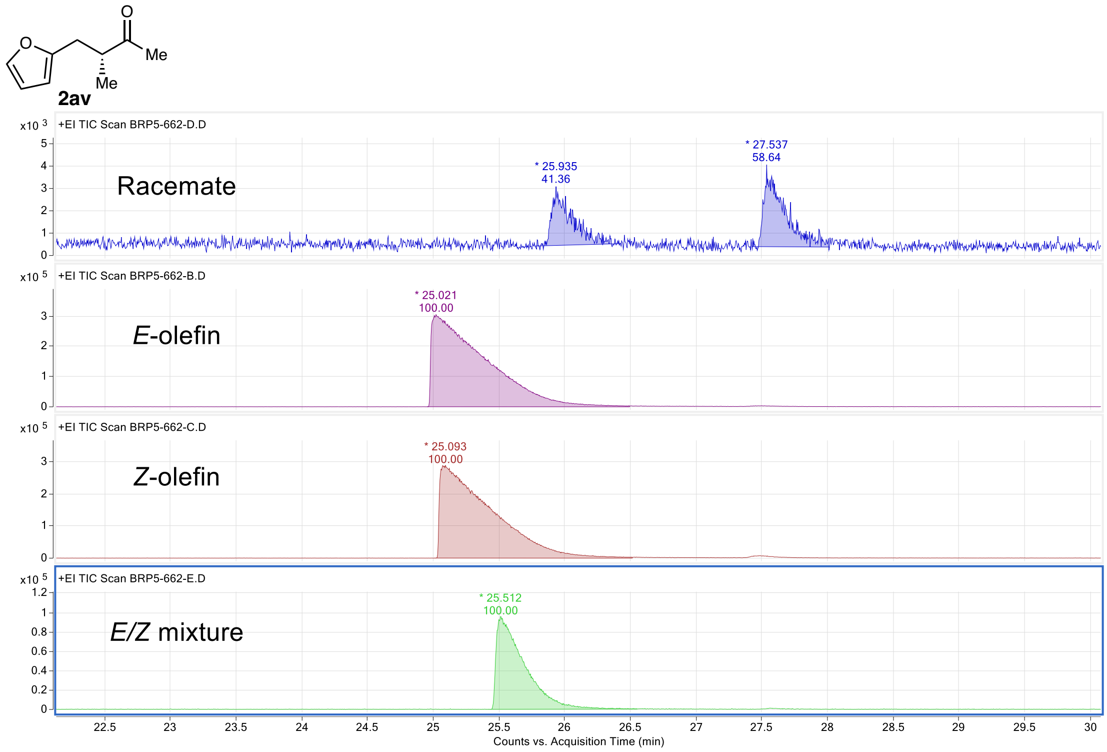

Supplementary Figure 127. GC spectra of **2av**.

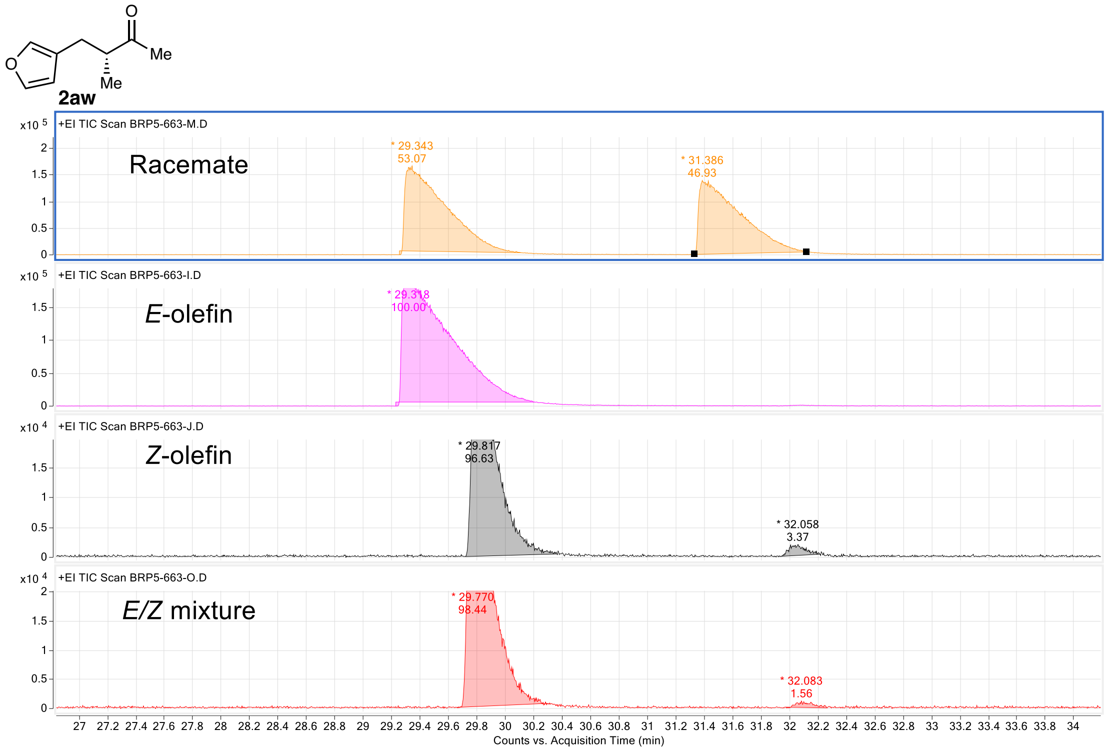

Supplementary Figure 128. GC spectra of **2aw**.

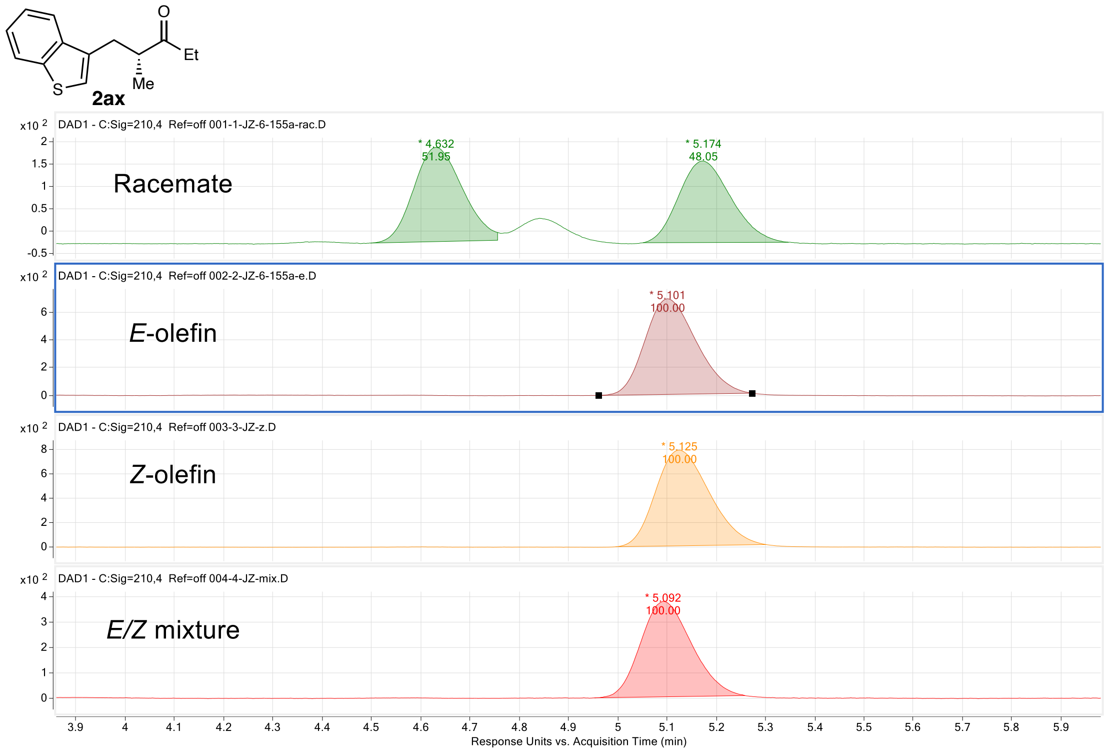

Supplementary Figure 129. SFC spectra of **2ax**.

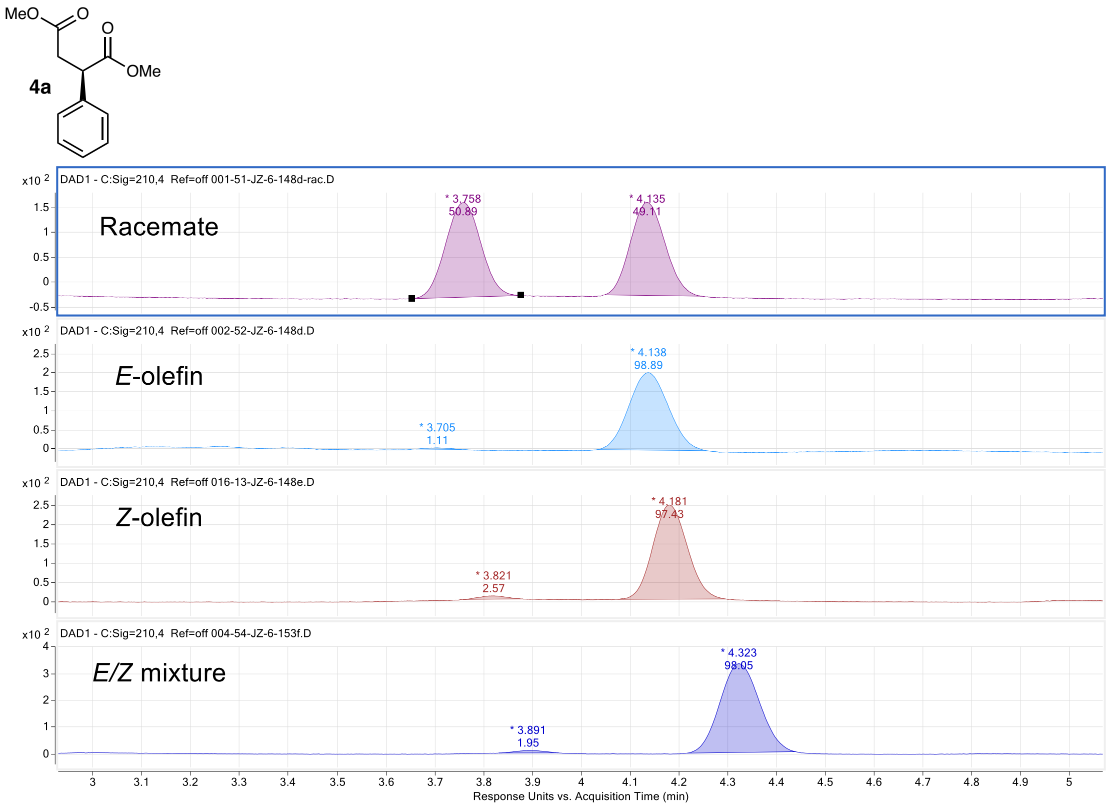

Supplementary Figure 130. SFC spectra of **4a**.

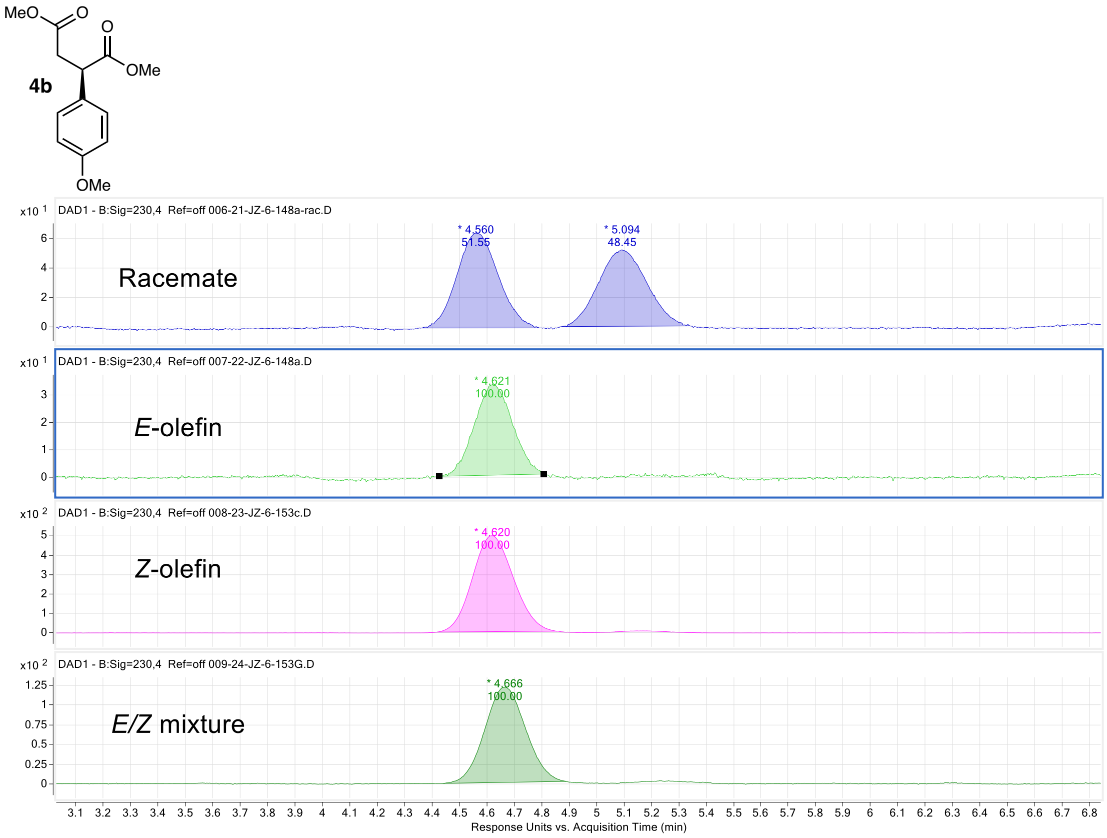

Supplementary Figure 131. SFC spectra of **4b**.

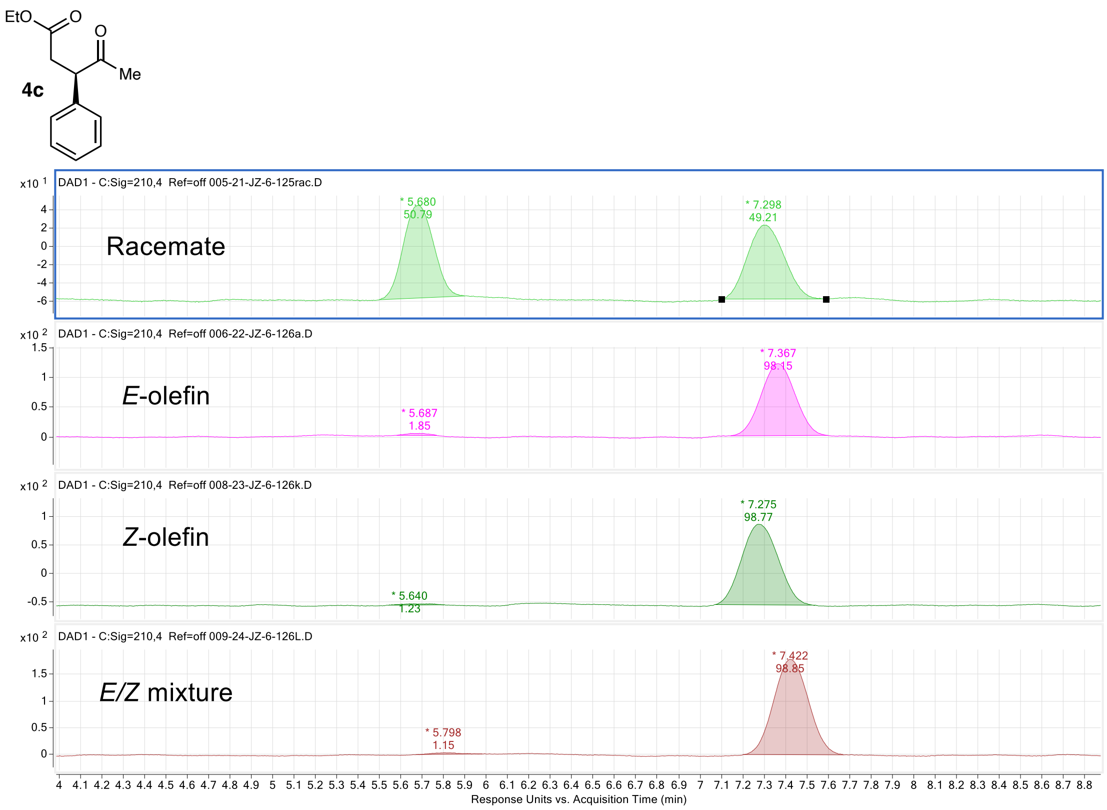

Supplementary Figure 132. SFC spectra of **4c**.

Supplementary References

(1) Peters, B. B. C.; Jongcharoenkamol, J.; Krajangsri, S.; Andersson, P. G. *Org. Lett.* **2020**, *23*, 242-246.
(2) O'Brien, C. J.; Nixon, Z. S.; Holohan, A. J.; Kunkel, S. R.; Tellez, J. L.; Doonan, B. J.; Coyle, E. E.; Lavigne, F.; Kang, L. J.; Przeworski, K. C. *Chem. Eur. J.* **2013**, *19*, 15281-15289.
(3) Bressin, R. K.; Driscoll, J. L.; Wang, Y.; Koide, K. *Org. Process Res. Dev.* **2019**, *23*, 274-277.
(4) Niwa , T.; Ochiai, H.; Isoda , M.; Hosoya, T. *Chem. Lett.* **2017**, *46*, 1315-1318
(5) Pelletier, G.; Bechara, W. S.; Charette, A. B. *J. Am. Chem. Soc.* **2010**, *132*, 12817-12819.
(6) Yan, Q.; Xiao, G.; Wang, Y.; Zi, G.; Zhang, Z.; Hou, G. *J. Am. Chem. Soc.* **2019**, *141*, 1749-1756.
(7) Aboujaoude, E. E.; Liétjé, S.; Collignon, N.; Teulade, M. P.; Savignac, P. *Synthesis* **1986**, *11*, 934-937
(8) Kabalka, G. W.; Guchhait, S. K. *Org. Lett.* **2003**, *5*, 729-731
(9) Akula, P. S.; Hong, B.-C.; Lee, G.-H. *Org. Lett.* **2018**, *20*, 7835-7839.
(10) Li, S.; Huang, K.; Zhang, X. *Chem. Commun.* **2014**, *50*, 8878-8881.
(11) Dumez, E.; Faure R.; Dulcère, J.-P. *Eur. J. Org. Chem.* **2001**, *13*, 2577-2588.
(12) Seashore-Ludlow, B.; Somfai, P. *Org. Lett.* **2012**, *14*, 3858-3861.
(13) Lu, S.-M.; Bolm, C. *Angew. Chem. Int. Ed.* **2008**, *47*, 8920 –8923.
(14) Falconnet, A.; Magre, M.; Maity, B.; Cavallo, L.; Rueping, M. *Angew. Chem. Int. Ed.* **2019**, *58*, 17567-17571.
(15) Silva, R. M.; Okano, L. T.; Rodrigues, J. A. R.; Clososki, G. C. *Tetrahedron: Asymmetry* **2017**, *28*, 939-944.
(16) Brenna, E.; Cosi, S. L.; Ferrandi, E. E.; Gatti, F. G.; Monti, D.; Parmeggiani, F.; Sacchettia, A. *Org. & Biomol. Chem.* **2013**, *11*, 2988-2996.
(17) Sai, M.; Matsubara, S. *Adv. Synth. Catal.* **2019**, *361*, 39-43.
(18) Panteleev, J.; Huang, R. Y.; Lui, E. K. J.; Lautens, M. *Org. Lett.* **2011**, *13*, 5314-5317.
(19) Bernasconi, M.; Müller, M.-A.; Pfaltz, A. *Angew. Chem. Int.Ed.* **2014**, *53*, 5385-5388.
(20) Litman, Z. C.; Wang, Y.; Zhao, H.; Hartwig, J. F. *Nature* **2018**, *560*, 355–359.
(21) Hedberg, C.; Källström, K.; Brandt, P.; Hansen, L. K.; Andersson, P. G. *J. Am. Chem. Soc.* **2006**, *128*, 2995-3001.
(22) Metallinos, C.; Belle, L. V. *J. Organometal. Chem.* **2010**, *696*, 141-149.
(23) Fang, Y.; Yuan M.; Jin, X.; Zhang, L.; Li, R.; Yang, S.; Fang, M. *Tetrahedron let.* **2016**, *57*, 1368-1371.
(24) Masllorens, J.; Bouquillon, S.; Roglans, A.; Henin, F.; Muzart, J. *J. Organometal. Chem.* **2005**, *690*, 3822-3826.
(25) Torregrosa, R. R. P.; Ariyarathna, Y.; Chattopadhyay, K.; Tunge, J. A. *J. Am. Chem. Soc.* **2010**, *132*, 9280–9282.
(26) Mannathana, S.; Cheng, C.-H. *Adv. Synth. Catal.* **2014**, *356*, 2239 – 2246.
